# Solvation Effects on the Structure and Stability of Alkali Metal Carbenoids

**DOI:** 10.1002/anie.202011278

**Published:** 2020-11-03

**Authors:** Katharina Dilchert, Michelle Schmidt, Angela Großjohann, Kai‐Stephan Feichtner, Robert E. Mulvey, Viktoria H. Gessner

**Affiliations:** ^1^ Chair of Inorganic Chemistry II Faculty of Chemistry and Biochemistry Ruhr-University Bochum Universitätsstraße 150 44801 Bochum Germany; ^2^ WestCHEM Department of Pure and Applied Chemistry University of Strathclyde Glasgow G1 1XL UK

**Keywords:** alkali metals, carbenoids, lithium, NMR spectroscopy, structure–reactivity relationship

## Abstract

s‐Block metal carbenoids are carbene synthons and applied in a myriad of organic transformations. They exhibit a strong structure–activity relationship, but this is only poorly understood due to the challenging high reactivity and sensitivity of these reagents. Here, we report on systematic VT and DOSY NMR studies, XRD analyses as well as DFT calculations on a sulfoximinoyl‐substituted model system to explain the pronounced solvent dependency of the carbenoid stability. While the sodium and potassium chloride carbenoids showed high stabilities independent of the solvent, the lithium carbenoid was stable at room temperature in THF but decomposed at −10 °C in toluene. These divergent stabilities could be explained by the different structures formed in solution. In contrast to simple organolithium reagents, the monomeric THF‐solvate was found to be more stable than the dimer in toluene, since the latter more readily forms direct Li/Cl interactions which facilitate decomposition via α‐elimination.

## Graphical abstract

Whereas in conventional organolithium chemistry unsolvated dimers are generally more stable than solvated monomers and their sodium and potassium analogues are less stable still, curiously this trend seems to be reversed in carbenoid systems. DOSY‐NMR, SC‐XRD analyses and computational studies on a chiral test system show dramatic changes in the stability depending on the solvent and the formed structures.

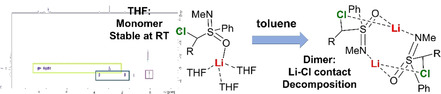

## Introduction

Polar organometallics, particularly organolithium or Grignard reagents are amongst the most important organometallic compounds used in synthetic chemistry.^[1]^ They are also routinely applied as strong bases and metalation agents in large scale processes.^[2]^ A key property of organolithium and s‐block metal compounds in general is the strong relationship between their structure and reactivity.^[3]^ Thus, intense research efforts have focussed on the elucidation of the structures and structure formation principles of these compounds to better understand and control the activity of these typically highly reactive compounds. This has led to remarkable advances in this chemistry by the design of reagents with defined structures and properties.^[4]^ Hence, s‐block metal compounds are no longer predominately used just as strong bases, but also as highly engineered reagents that allow for new applications or unprecedented selectivities.^[5]^

Carbenoids are a subclass of polar organometallic reagents that unlike the highly nucleophilic, simple organometallic compounds exhibit an advantageous ambiphilic character due to a leaving group bound directly to the metallated carbon atom.^[6]^ This makes them highly attractive carbene synthons with a broad range of applications, such as in cyclopropanation^[7]^ or homologation reactions.^[8]^ However, the presence of both a metal and a leaving group at a single carbon atom also results in an inherent thermal lability. In the case of lithium and magnesium carbenoids decomposition reactions via metal salt elimination often occur at low temperatures, thus impeding a controlled handling and isolation so that many transformations are still performed with in situ prepared reagents. Despite the synthetic advances^[9]^ and progresses in the stabilization^[10]^ and handling^[11]^ of carbenoids made in past years, only little information has been accrued on the impact of the structures on the reactivity. While the solid‐state structures of several s‐block metal carbenoids have been elucidated by single‐crystal X‐ray diffraction analysis (XRD),^[12]^ their solution chemistry is almost unexplored. This is surprising, since reactions are usually carried out in solution and strong solvent effects have frequently been observed. For example, Köbrich already noted in the 1960s that coordinating solvents, in particular THF stabilize carbenoids and thus lead to higher selectivities.^[13]^ This observation was repeatedly confirmed^[14]^ but no structure elucidations in solution have been performed to explain the role of the solvent and the origin of the different stabilities.

Herein, we describe the first detailed study on the impact of the solvent on the structure formation and the thermal stabilities of carbenoids. Based on sulfoximinoyl‐substituted alkali metal carbenoids we could show using a combination of VT‐DOSY NMR experiments together with XRD analyses and computational studies that the carbenoids form monomers in coordinating solvents (THF), while they aggregate to dimers in toluene. Unexpectedly and in contrast to the situation with conventional alkali metal reagents, this aggregation leads to a higher reactivity/reduced thermal stability due to the more facile formation of metal‐chlorine interactions. This phenomenon is most pronounced for lithium, while the heavier alkali metal compounds appear to be less “solvent‐sensitive” and hence offer a convenient alternative for nucleophilic carbenoids.

## Results and Discussion

**Scheme 1:**
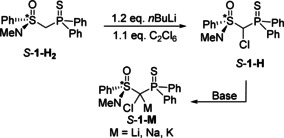
Preparation of the congeneric alkali metal carbenoids **1‐M** (Base=MeLi, NaH or KH in THF).

In order to shed light on the structure activity relationships of s‐block metal carbenoids in solution, we set out to gain a more profound understanding of the structure formation in solution. To this end, we chose the chiral thiophosphoryl‐substituted sulfoximine **1‐M** as test system. The sulfoximinoyl moiety was selected due to three reasons: i) sulfoximine carbenoids have successfully been used in carbenoid chemistry such as for asymmetric cyclopropanations and are thus also of synthetic interest.^[15]^ ii) The sulfoximinoyl group together with the thiophosphoryl unit promised sufficient stabilization of the carbenoid to facilitate its handling. iii) The stereocentre at sulfur also offered a further spectroscopic probe which might provide further information about the structure formation in solution. The chlorinated precursor **1‐H** was synthesised from the CH_2_‐containing compound **1‐H_2_**, which is accessible by a multi‐step procedure that involves racemic resolution with camphorsulfonic acid (see the Supporting Information).^[16]^ Chlorination was accomplished by lithiation with *n*‐BuLi and treatment with hexachloroethane, thus giving **1‐H** as a mixture of two diastereoisomers in 54 % yield (Scheme 1). The two isomers give rise to singlets in the ^31^P{^1^H} NMR spectrum at *δ*_p_=45.1 and *δ*_p_=46.5 ppm, respectively, and a doublet at *δ*_H_=5.79 and 5.88 in the ^1^H NMR spectrum for the central hydrogen atom. The chlorinated carbon appears at *δ*_C_=69.1 and 75.7 ppm with a coupling constant of ^1^ *J*_PC_=32.4 and 36.0 Hz, respectively. The metalation of **1‐H** was achieved with different metal bases (MeLi, NaH or KH) in THF at low temperatures thus giving the corresponding lithium, sodium and potassium carbenoid **1‐M** in high yields of 88–93 %.

The carbenoids **1‐M** are characterized by a signal at approx. 44 ppm in the ^31^P{^1^H} NMR spectrum and doublet at approx. 50 ppm in the ^13^C{^1^H} NMR spectrum, which is high‐field shifted compared to the protonated precursors (Table 1). Such high‐field shifted signals are typical for stabilized carbenoids,^[10]^ while non‐stabilized carbenoids often showed a distinct down‐field shift.^[17]^ The fact that all carbenoids **1‐M** feature only one set of signals in the NMR spectra indicates that the stereoinformation at the central carbon atom is lost presumably due to a change in hybridization from sp^3^ to sp^2^. This is also in line with the increase of the ^1^ *J*_PC_ coupling constant, which confirms a higher s‐character in the P−C bond. Consequently, it can be assumed that no or at least no strong C−M interactions are present in solution.

**Table 1:** NMR spectroscopic and crystallographic properties of *rac*‐**1** and *rac*‐**1‐M** in [D_8_]THF, decomposition temperatures in THF (D_THF_) and toluene (D_Tol_).

	**1‐H**	**1‐Li**	**1‐Na**	**1‐K**
*δ*_P_ [ppm]	45.1; 46.5	42.2	43.1	44.1
*δ*_C_ [ppm] (^1^ *J*_PC_ [Hz])	69.1; 75.7 (32.4, 36.0)	49.2 (83.1)	50.4 (88.5)	49.8 (86.3)
C−Cl (Å)	1.7737(19)	1.773(1)	1.778(4)^[a]^	1.780(6)^[a]^
^1^ *J*_PC_ [Hz]	32.4; 36.0	83.1	88.8	86.3
D_THF_ [°C]	–	>50	50	40
D_Tol_ [°C]	–	‐10	50	50

[a] Parameters taken from the structures with crown ether (see SI).

To probe the thermal stability of the carbenoids and the impact of the solvent on the stability, VT‐NMR studies of **1‐M** in THF as coordinating and toluene as non (weakly) coordinating solvents were performed using the racemic mixture of **1‐M**. To this end, the carbenoids were at first prepared at low temperatures in THF to facilitate the handling of the compounds. After filtration and confirmation of the successful preparation, the solvent was removed in vacuo and the carbenoid dissolved in precooled [D_8_]THF or [D_8_]toluene. The obtained solutions were warmed from −30 to 50 °C in 10 °C increments and kept at each temperature for 1 h, after which ^1^H and ^31^P{^1^H} NMR spectra were recorded. The thus obtained decomposition temperatures (Table 1, see SI for NMR spectra) revealed remarkable differences between the different alkali metals and solvents. In THF, all carbenoids revealed to be stable even above room temperature. Surprisingly, **1‐Li** seemed to be slightly more stable than the sodium and potassium congeners, which is in contrast to previous observations made in our group.^[18]^ However, in toluene a distinct higher stability was found for **1‐Na** and **1‐K**. Here, **1‐Li** is the by far least stable species already decomposing at −10 °C. Approx. 15 % of the carbenoid was decomposed after 1 h at that low temperature. In contrast, **1‐Na** and **1‐K** were stable at room temperature independent of the solvent.

To understand the different stabilities in the different solvents we turned our attention towards structure elucidation. To gain first insights into the structures of **1‐M** we initially attempted the structure determination in the solid state. Pleasingly, single crystals of *rac*‐**1‐Li** could be obtained from a THF solution of the carbenoid. Surprisingly, *rac*‐**1‐Li** crystallizes as monomer in which the lithium ion is coordinated solely by the oxygen of the sulfoximine unit and three additional THF molecules. This is in contrast to the dimeric structures of sulfones reported in the literature.^[19]^ No coordination of the metal to the carbon atom, the chlorine or the thiophosphoryl group is observed. The missing C‐Li contact is in line with NMR spectroscopic observations, that is, the large coupling constant and the lost stereoinformation at C1. The C−Cl bond of 1.773(1) Å is identical to the one found in the protonated precursor **1‐H** and thus is in line with a strong C−Cl bond and the high stability of **1‐Li** in THF.

Single‐crystals could also be grown for *rac*‐**1‐Na** and *rac*‐**1‐K,** yet only as crown ether adducts, which form ligand‐separated ion pairs and therefore showed no contact between the metal and **1** (see the SI). Nonetheless, the obtained structure parameters were similar to those found for [(*rac*‐**1‐Li**)⋅3THF]. Any further attempts to obtain crystals from *rac‐* or (*R*)‐**1‐M** failed. To gain insights into the solution structure of **1‐M** we addressed DOSY (Diffusion Ordered SpectroscopY) NMR studies, which have successfully been used for structure elucidations of assorted s‐block metal organyls but hitherto never been applied for carbenoids.[^20^, ^21^] We particularly focussed on the question, “how are the structures of the carbenoids **1‐M** influenced by the different solvents and by temperature?” To answer these questions DOSY NMR spectra were recorded at −30, −10 and 27 °C in toluene and THF, respectively. The carbenoid solutions were prepared in the same way as those in the decomposition studies except for the addition of the DOSY NMR standards (TMS and adamantane, respectively). Figure 2 exemplarily depicts the DOSY NMR spectrum of *rac*‐**1‐Li** in THF with TMS as internal standard, while all other results are summarized in Table 2 and the SI.

**Figure 1:**
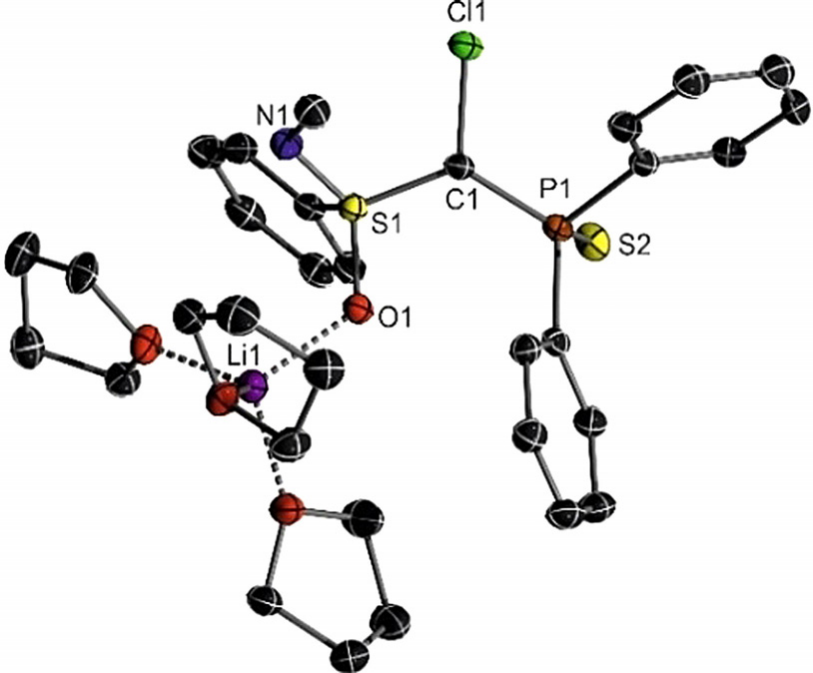
Molecular structure of the THF solvated Li/Cl carbenoid *rac*‐**1‐Li**. Ellipsoids at the 50 % probability level, hydrogens omitted for clarity. Selected bond lengths [Å] and angles [°]: P1‐C1 1.7586(15), S1‐C1 1.6933(15), Cl1‐C1 1.7734(14), O1‐Li 1.935(3), S1‐C1‐Cl1 111.29(8), P1‐C1‐S1 126.80(8), P1‐C1‐Cl1 111.59(8).

**Figure 2:**
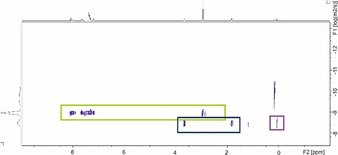
2D DOSY NMR spectrum of *rac*‐**1‐Li** in [D_8_]THF with TMS as internal standard measured at 27 °C; green frame: signal of *rac*‐**1‐Li**, blue frame: THF, purple frame: TMS (internal standard).

**Table 2:** Molecular weights determined by DOSY NMR (MW_det_)^[a]^ for *rac*‐**1‐M** in [D_8_]THF and [D_8_]toluene at different temperatures, suggested structures and the difference to the calculated molecular weight (MW_det_).^[b]^

Compound	MW_det_ [g mol^−1^]	Structure	MW_Dif_ [%]		MW_det_ [g mol^−1^]	Structure	MW_Dif_ [%]
	**[D_8_]THF**				**[D_8_]toluene^[c]^**		
**1‐Li** 27 °C	464	monomer + 1THF	−6.8		904	dimer dimer + 1THF	+6.1 −2.1
							
**1‐Na** 27 °C	471	monomer monomer+1THF	6.6 −8.3		1330	dimer + 6THF or Trimer	+1.0 +0.3
							
**1‐K** 27 °C	521	monomer + 1THF	−1.7		1135	dimer + 3THF	+0.2
							
**1‐Li** −10 °C	554	monomer + 2THF	−2.8		1051	dimer + 2 or 3THF	+5.5 −1.6
							
**1‐Li** −30 °C	600	monomer+ 2 or + 3THF	+5.3 −6.6		1025	dimer + 2 or 3THF	+2.9 −4.0

[a] determined by the ECC‐*MW* estimation software by Stalke and co‐workers. See ref. [21] for details. [b] TMS was used as internal standard in THF, adamantane in toluene. [c] In the case of the toluene experiments, THF is suggested to coordinate to the metal since for solubility and stability reasons the synthesis had to be done in THF and complete removal of all solvent molecules could not be guaranteed even after thoroughly drying in vacuo.

The obtained results allow two important conclusions: firstly, independent of the metal and temperature, monomers are formed in THF, while dimers (or trimers) are formed in toluene. This trend is similar to that of simple organoalkali metal compounds, which tend to form larger aggregates in non‐coordinating solvents to stabilize the charges of the polar M−C bond.^[3]^ Secondly, the size of the aggregates increases at lower temperatures. In line with entropic effects more solvent molecules coordinate to the metal at lower temperatures. For example, while **1‐Li** forms a monomer with only one (or no) coordinating THF at 27 °C, two or three solvent molecules, respectively, are binding to the carbenoid at −10 and −30 °C. This corroborates nicely with the tri‐solvated structure found in the solid state for *rac*‐**1‐Li** (Figure 1).

**Scheme 2:**
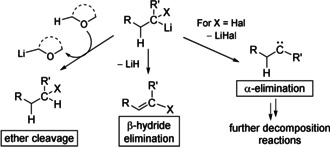
Decomposition pathways of simple organolithium compounds and carbenoids.

The DOSY NMR studies together with the observed stabilities of **1‐M** (Table 1) suggest that the carbenoids are stabilized by lower aggregation states (monomer versus dimer) as well as by the coordination of more solvent molecules to the metal (high versus low temperatures). The latter can probably be explained by the fact that solvent coordination to the metal weakens or totally destroys the metal halogen interaction and thus hampers MCl formation. The higher stability of monomeric relative to dimeric **1‐Li** contrasts with simple organolithium reagents which typically form larger aggregates to decrease their reactivity.[^3^, ^22^] This divergent behaviour may be explained by different decomposition pathways (see Scheme 2). Carbenoids in general decompose via MX elimination, while for simple s‐block metal organyls β‐hydride elimination and/or solvent decomposition are the most dominant decomposition pathways. Of course, carbenoids may also attack the solvent or undergo β‐hydride elimination. However, these processes usually exhibit higher activation barriers and are thus less critical than the α‐elimination process. From the DOSY NMR experiments one may now draw the conclusion that dimers are less stable than monomers because they facilitate or strengthen M‐X interaction and hence concomitantly increase the tendency for MX elimination. This is in agreement with the higher yields and selectivities obtained with lithium carbenoids in THF compared to reactions performed in toluene.[^13^, ^14^]

To further probe this relationship between the structure and the stability of the carbenoids we addressed computational studies (PBE0‐D3/def2tzvp//PBE0‐D3/defsvp). To this end, a series of monomeric and dimeric structures of all carbenoids **1‐M** were calculated, in which the metal is coordinated by combinations of the S=O, S=N and P=S linkages, the Cl atom as well as additional solvent molecules. The energies were calculated in both toluene and THF using a polarizable continuum model. THF molecules binding to the metal were explicitly calculated. All structures and the corresponding energies are given in the Supporting Information. Overall, the calculations confirm that monomers preferentially form in THF, while dimeric structures are preferred in toluene. In the case of lithium, which showed the most pronounced solvent effects, the S=O coordinated monomer observed in the solid state (Figure 1) was found to be the energetically most favored structure in THF. This structure is more than 25 kJ mol^−1^ lower in energy than any other complex in which lithium is coordinated by the Cl, P=S, S=N donors or any combination of those. The analogous structures but with 4 instead of 3 THF molecules are the most favoured structure for the larger Na and K cations.

**Figure 3:**
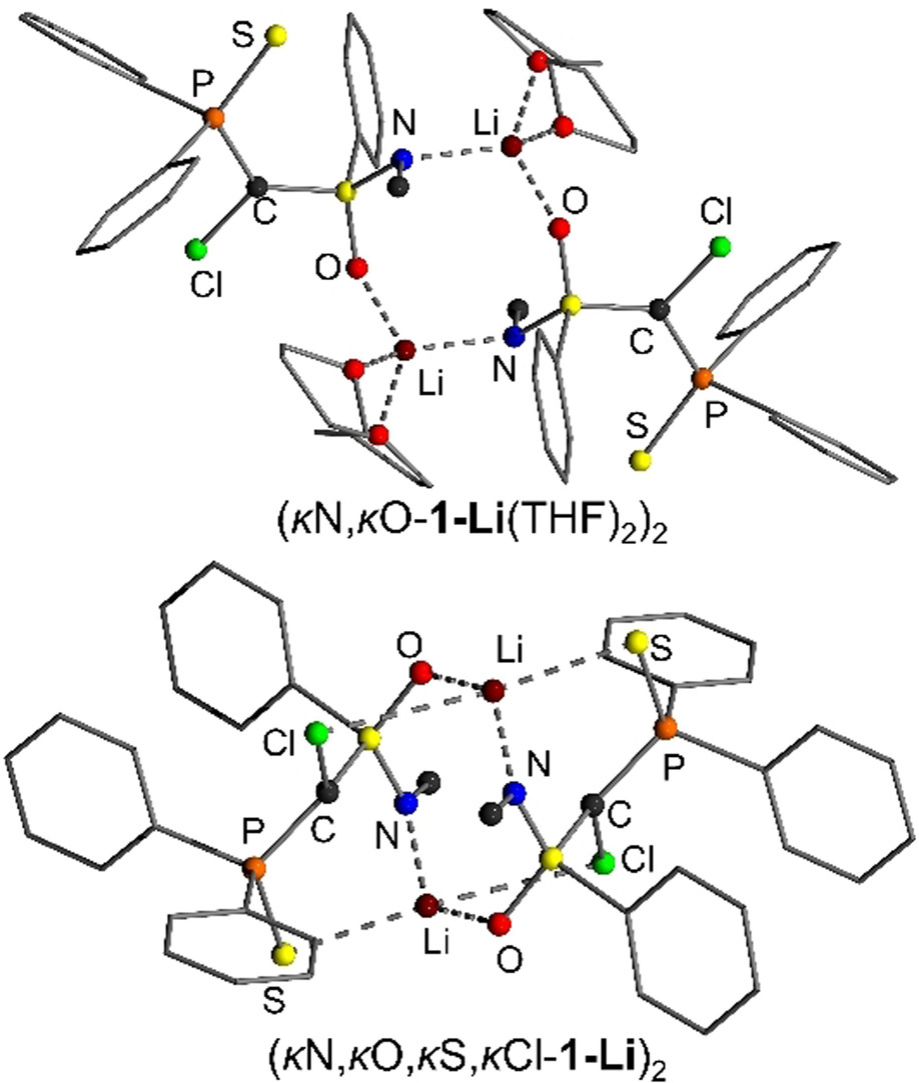
Calculated model structures of the two energetically most favoured dimeric structures (top) with and (bottom) without coordinating THF molecules.

In toluene, the dimeric structure (*κ*N,*κ*O‐**1‐Li**(THF)_2_)_2_ is favoured by 15 kJ mol^−1^ over the energetically most favoured monomer (Figure 3). In this dimer, the lithium has an NOOO coordination via the S=N and S=O groups, and two THF molecules. An analogous structure was reported by Zehnder and co‐workers for a simple *N*‐methyl‐*S*‐phenylsulfoximine^[23]^ as well as by Gais and others for a series of different sulfones.^[19]^ In the dimer, again there is no direct contact between the lithium and the chlorine atom. However, when fully excluding the coordination of any solvent molecule to the carbenoid—as was found to be the case for lithium at room temperature by the DOSY experiments (see Table 2)—the dimeric structure (*κ*N,*κ*O,*κ*S,*κ*Cl‐**1‐Li**)_2_ with coordination of the metal by all donor atoms including Cl is the most favoured species. This structure is easily formed from the THF‐coordinated dimer (*κ*N,*κ*O‐**1‐Li**(THF)_2_)_2_ by replacement of the two THF molecules with contacts to the P=S group and Cl atom. (*κ*N,*κ*O,*κ*S,*κ*Cl‐**1‐Li**)_2_ is more than 50 kJ mol^−1^ lower in energy than any other THF‐free dimer and only 25.3 kJ mol^−1^ higher in energy than (*κ*N,*κ*O‐**1‐Li**(THF)_2_)_2_, when assuming that 1 equiv THF is present per carbenoid. Since solvent coordination is fluxional, it is likely that in toluene a series of dimeric structures based on (*κ*N,*κ*O,*κ*S,*κ*Cl‐**1‐Li**)_2_ with varying numbers of coordinating THF are present. According to the DOSY NMR experiments the number of coordinating THF molecules decreases at higher temperature. Thus, THF‐free (*κ*N,*κ*O,*κ*S,*κ*Cl‐**1‐Li**)_2_ and its analogue with one coordinating THF molecule are the most favoured structures at room temperature. This nicely explains, the observed decomposition of **1‐Li** in toluene. While at lower temperatures the dimeric structure with no direct Li−Cl bond is favoured, Li−Cl contacts start to form when sufficient THF molecules dissociate from the dimer. These Li−Cl interactions initiate the α‐elimination and hence the carbenoid decomposition.

Note, that monomeric **1‐Li** also loses coordinating solvents upon warming. At room temperature a structure with only one THF molecule is formed according to the DOSY experiments. The calculations suggest that this monomer is either [*κ*O,*κ*S‐**1‐Li**(THF)] or [*κ*N,*κ*S,*κ*Cl‐**1‐Li**(THF)] (these are within only 3.5 kJ mol^−1^ of each other with preference for the monomer with a Li−Cl contact), thus indicating that here also Li−Cl contacts should start to form and that the carbenoid should slowly decompose. This contradicts the observed stability of **1‐Li** at room temperature and suggests that in fact no Li−Cl is formed. We speculate that this may be due to an appreciable activation barrier to form the Li−Cl contact in solution which is only overcome at higher temperatures.

Overall, the observation made with the model carbenoid **1‐Li** clearly demonstrates the importance of the coordination of donor groups/ligands to the metal to stabilize the carbenoid by weakening or preventing a direct contact between the metal and the leaving group. Thus, coordinating solvents such as THF impart a stabilizing effect on the carbenoid, while non or only weakly coordinating solvents such as toluene cannot compete with the donation of the halide and hence lead to lower stabilities. In our experiments, only small amounts of THF resulted in a remarkable increase in stability. While it is possible to handle the carbenoid in toluene solution when preformed in THF (that is, with only few THF molecules present per carbenoid), it was impossible to prepare samples of the carbenoid in pure toluene without observing decomposition already at temperatures even as low as −50 °C. Until now, it is unclear why sodium and potassium do not show such a distinct solvent dependency compared to that of lithium. One explanation might be the weaker M−Cl interaction, which lowers the tendency to form the corresponding metal salt even when a M−Cl interaction is present in the molecule. This suggests in accordance with previous observations,^[18]^ that sodium and potassium carbenoids might in general be better suited in reactions which only make use of the nucleophilic character of the carbenoid.

## Conclusion

In conclusion, by uncovering the first structures of challenging s‐block metal carbenoids in solution we have provided insights into the strong solvent dependencies of reactions described in the literature. Systematic studies revealed a considerably lower stability of the lithium compound in toluene compared to that in THF. DOSY NMR experiments supported by computational studies showed that this solvent dependency can be correlated with different structures formed in the respective solvents. While monomers with no direct Li−Cl contact are formed in THF, dimers with Li⋅⋅⋅Cl interactions are present in toluene, thus leading to the more facile salt elimination and carbenoid decomposition. These studies clearly demonstrate that coordinating solvents stabilize carbenoids and so allow for more selective transformations. The fact, that also stoichiometric amounts of coordinating solvents lead to a higher stability suggest that a further control of reactivity should be possible by using strong polydentate donor ligands such as crown ethers as additives. Such strategies should be employed systematically in future to open up further possibilities for selective application of these usually highly reactive reagents.

## Conflict of interest

The authors declare no conflict of interest.

## Supporting information

As a service to our authors and readers, this journal provides supporting information supplied by the authors. Such materials are peer reviewed and may be re‐organized for online delivery, but are not copy‐edited or typeset. Technical support issues arising from supporting information (other than missing files) should be addressed to the authors.

Supplementary
