# Supplementary material for: Solvation Effects on the Structure and Stability of Alkali Metal Carbenoids
Source: Angew Chem Int Ed Engl. 2020 Nov 3;60(1):493–8. doi: 10.1002/anie.202011278 (PMC7821203; doi:10.1002/anie.202011278)
Supplement: Supplementary file 1 — Supplementary [file ANIE-60-493-s001.pdf]

Angewandte Chemie
Eine Zeitschrift der Gesellschaft Deutscher Chemiker

Supporting Information

Solvation Effects on the Structure and Stability of Alkali Metal Carbenoids

Katharina Dilchert, Michelle Schmidt, Angela Großjohann, Kai-Stephan Feichtner, Robert E. Mulvey, and Viktoria H. Gessner**

anie_202011278_sm_miscellaneous_information.pdf

Index

1. Experimental Details 2

1.1 General methods 2

1.2 Synthesis of the chiral carbenoids 3

2. NMR spectra 5

2.1 NMR spectra of the isolated compounds 5

2.2 VT $^{31}P\{^{1}H\}$ NMR spectroscopy 13

3. DOSY NMR spectroscopy 17

3.1 General procedure 17

3.2 DOSY NMR data of rac-1-Li 18

3.3 DOSY NMR Data of rac-1-Na 22

3.4 DOSY NMR data of rac-1-K 26

4. Determination of ee 30

5. Crystal Structure Determination 31

5.1 General information 31

5.2 Further Details to the Crystal Structures 33

6. Computational Details 52

6.1 General 52

6.2 Structures of the energy-optimized compounds 53

6.3 Energies of the optimized compounds 56

6.4 Coordinates 59

6.4.1 Coordinates of the monomeric structures of 1-Li 59

6.4.2 Coordinates of the dimeric structures of 1-Li 74

6.4.3 Coordinates of the monomeric structures of 1-Na 93

6.4.4 Coordinates of the dimeric structures of 1-Na 108

6.4.5 Coordinates of the monomeric structures of 1-K 118

6.4.6 Coordinates of the dimeric structures of 1-K 132

7. References 141

1. Experimental Details

1.1 General methods

All experiments were carried out under a dry, oxygen-free argon atmosphere using standard Schlenk techniques. Involved solvents were dried using an MBraun SPS-800 (THF, toluene, Et_2O, DCM, pentane, hexane) or dried in accordance with standard procedures. H_2O is distilled water. ^{1}H, $^{13}C\{^{1}H\}$, $^{31}P\{^{1}H\}$ NMR spectra were recorded on Avance-400 or AVIII-400 spectrometers at 25 °C if not stated otherwise. All values of the chemical shift are in ppm regarding the δ-scale. All spin-spin coupling constants (*J*) are printed in Hertz (Hz). To display multiplicities and signal forms correctly the following abbreviations were used: s = singlet, d = doublet, t = triplet, m = multiplet, br = broad signal. Signal assignment was supported by DEPT, APT, HSQC and HMBC experiments and by literature studies on similar compounds.

Elemental analyses were performed on an Elementar vario MICRO-cube elemental analyzer.

For column chromatography silica gel 60M purchased from Machery-Nagel was used. The solvent mixtures are given as volume fractions (v/v). Pre-coated TLC sheets (ALUGRAM ALOX N/UV_{254}) with fluorescence indicator purchased from Machery-Nagel were used. The detection was done by means of UV light (λ = 254 nm).

For automated Column Chromatography a Reveleris X2 Flash Instrument by Büchi was used. Solid loaders with a screw cap and Flash cartridges (FlashPure Silica) by Büchi were used. The detection was done by means of integrated UV detection (λ_1 = 254 nm, λ_2 = 265 nm, λ_3 = 280 nm) and ELSD.

For the determination of the *ee* an analytical Knauer Azura HPLC system equipped with a Dr. Maisch Reprosil Chiral-NR 8µm, 250•4.6 mm was used. A mixture of hexane and isopropanol (85:15) were used with a flow rate of 1.5 mL/min.

All other reagents were purchased from Sigma-Aldrich, ABCR, Rockwood Lithium or Acros Organics and used without further purification.

1-H_2 was synthesized according to literature.[1]

1.2 Synthesis of the chiral carbenoids

Synthesis of *R*-1

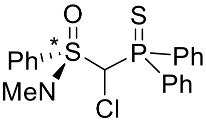

901 mg (2.337 mmol) of *R*-**2** were dissolved in 50 mL THF and cooled to –80 °C. 1.64 mL (1.71 M in Hex, 2.805 mmol) ^{n}BuLi were added dropwise. The yellow solution was stirred between –80 °C and –30° C for 1 h. 719.24 mg of hexachloroethane were dissolved in 50 mL THF. The yellow reaction mixture was transferred to hexachloroethane *via* temperature gradient and warmed to RT slowly overnight. 25 mL of H_2O were added, and the phases were separated. The aqueous phase was extracted with 25 mL of Et_2O three times. The combined organic phases were dried over Na_2SO_4. After filtration the solvent was removed *in vacuo*. The crude product was purified by column chromatography (THF/Hex 1:2.5, r_f = 0.4). The product was obtained as a white solid in 54 % yield (525 mg, 1.25 mmol). The same procedure was also used for racemic mixtures of the sulfoximine. Crystals suitable for X-ray diffraction analysis were obtained by slow evaporation of hexane from a solution of the rac-**1** at RT.

^{1}H NMR (400.33Hz, $CDCl_3$): δ_H = 2.77 + 2.88 (s + s, 3H; NCH_3), 5.71 + 5.78 (d + d, 1H, $^{2}J_{HP}$ = 4.49 Hz + 4.81 Hz; *C*HCl), 7.29-7.35 (m, 4H; C$H_{Ph,meta/para/ortho}$), 7.37-7.51 (m, 5H, C*H*), 7.73-7.93 (m, 6H; C$H_{Ph,ortho/meta/para}$) **^{13}C{^{1}H} NMR** (100.67 Hz, $CDCl_3$): δ_c = 29.6 (s, NCH_3), 31.0 (s, NCH_3), 69.2 (s, *C*Cl) 75.9 (s, *C*Cl), 128.5 (s, P$C_{Ph, para}$) 128.5 (s, P$C_{Ph, para}$), 128.6 (bs, P$C_{Ph, para}$), 129.0 (s, $S_{Ph. meta}$), 129.2 (s, $S_{Ph. meta}$), 130.3 (s, P$C_{Ph, ipso}$), 130.6 (s, P$C_{Ph, para}$), 130.6 (s, P$C_{Ph, para}$), 130.9 (s, S$C_{Ph, ortho}$), 131.6 (s, P$C_{Ph, para}$), 132.1 (s, P$C_{Ph, ortho/meta}$), 132.1 (s, P$C_{Ph, ortho/meta}$), 132.2 (s, P$C_{Ph, ortho/meta}$), 132.2 (s, P$C_{Ph, ortho/meta}$), 132.2 (s, P$C_{Ph, ortho/meta}$), 132.3 (s, P$C_{Ph, ortho/meta}$), 132.4 (s, P$C_{Ph, ortho/meta}$), 132.5 (s, P$C_{Ph, ortho/meta}$), 133.5 (s, S$C_{Ph, para}$), 134.0 (s, S$C_{Ph, para}$), 135.6 (s, S$C_{Ph, ipso}$), 139.0 (s, S$C_{Ph, ipso}$) **^{31}P{^{1}H} NMR** (162.1 MHz, $CDCl_3$): δ_P = 45.1 (s), 46.5 (s). **Anal. Calcd**. for $C_{20}H_{19}ClNOPS_2$: C, 57.21; H,4.56; N,3.34; S,15.27. Found: C, 57.32; H, 4.55; N, 3.28; S, 15.30.

Preparation of *R*-1-Li·2THF

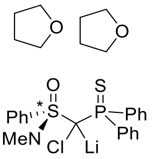

12.4 mg (0.0295 mmol) of *R*-**1** were dissolved in 1 mL of THF and cooled to 0 °C. 0.02 mL (0.0295 mmol, 1.6M in Et_2O) MeLi were added slowly. The resulting yellow solution was stirred for 1 h at 0 °C. The solvent was removed, and the product was obtained as a yellow solid in 93 % yield. *R*-**1** is coordinated by two THF molecules. (15.6 mg, 0.0274 mmol). NMR spectroscopic characterization was performed at –30 °C. Single crystals suitable for X-ray diffraction analysis were obtained from a concentrated THF solution at -30 °C.

^{1}H NMR (400.33 MHz, d_8-THF): δ_H = 2.91 (s, 3H; NCH_3), 7.13-7.19(m, 3H; SC$H_{Ph,meta,para}$ + PC$H_{Ph,meta,para}$),7.27–7.29 (m, 6H; SC$H_{Ph,meta,para}$ + PC$H_{Ph,meta,para}$), 7.70-7.75 (m, 2H; PC$H_{Ph,ortho}$), 8.11-8.13 (m, 4H; PC$H_{Ph,ortho}$ + SC$H_{Ph,ortho}$) **^{13}C{^{1}H} NMR** (100.67 MHz, d_8-THF):δ_C = 29.8 (s, NCH_3), 49.2 (d, $^{1}J_{P\text{-}C}$ = 83.1 Hz, *C*Cl), 127.8 – 128.1 (d+d, $^{3}J_{P\text{-}C}$ = 11.9Hz; P$C_{Ph,meta}$); 128.0 (s, S$C_{Ph, meta}$); 129.0 (S$C_{Ph, ortho}$); 130.0 (d, $^{4}J_{CP}$ = 2.5 Hz; $C_{Ph,para}$); 130.2 (d, $^{4}J_{CP}$ = 4.4 Hz; P$C_{Ph,para}$); 130.2 (s, $_{2}J_{CP}$ = 10.3 Hz; S$C_{Ph,para}$); 133.1 (d, $_{2}J_{CP}$ = 9.8 Hz; P$C_{Ph,ortho}$); 133.2 (d, $_{2}J_{CP}$ = 10.1 Hz; P$C_{Ph,ortho}$); 139.1 + 140.7 (d + d, $^{1}J_{CP}$ = 88.8 Hz + 96.2 Hz; P$C_{Ph,ipso}$); 145.8 (s, S$C_{Ph,ipso}$) **^{31}P{^{1}H} NMR** (162.1 MHz, d_8-THF): δ_P = 42.2 (s). **Anal. Calc.** $C_{28}H_{34}ClNLiO_3PS_2$: C 58.99, H 6.01, N 2.46, S 11.25. Found: C 58.76, H 5.81, N 2.74, S 11.64.

Preparation of *R*-1-Na·THF

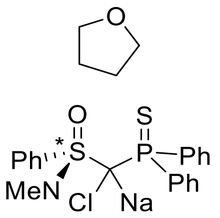

13.4 mg (0.0319 mmol) of *R*-**1** were dissolved in 1 mL of THF and cooled to 0 °C. Subsequently, the solution was added to 2.3 mg (0.1821 mmol) of NaH. The reaction solution was stirred for 1 h at 0 °C and subsequently filtered through a cannula. The solvent was removed *in vacuo*. The product was obtained as a pale-yellow solid in 92 % yield (15.1 mg, 0.0294 mmol). Suitable crystals for X-ray diffraction analysis were obtained by slow diffusion of pentane into a solution of 1eq. of 18-C-6 and *rac*-**1-Na·THF** in THF at -30 °C.

^{1}H NMR (400.3 MHz, d_8-THF): δ_H = 2.84 (s, 3H; NCH_3), 7.13-7.31 (m, 9H; SC$H_{Ph,meta,para}$ + PC$H_{Ph,meta,para}$), 7.85-7.89 (m, 2H; PC$H_{Ph,ortho}$), 7.98-8.10 (m, 4H; PC$H_{Ph,ortho}$ + SC$H_{Ph,ortho}$) **^{13}C{^{1}H} NMR** (100.7 MHz, d_8-THF): δ_C = 30.2 (NCH$_3$), 50.4 (d, $^{1}J_{CP}$ = 88.5 Hz; *C*Cl), 127.7 + 127.9 (d + d, $^{3}J_{CP}$ = 12.1 Hz; P$C_{Ph,meta}$), 128.1 (S$C_{Ph,meta}$), 128.4 (S$C_{Ph,ortho}$), 129.8 (S$C_{Ph,para}$), 130.0 (d, $^{4}J_{CP}$ = 2.2 Hz; P$C_{Ph,para}$), 130.2 (d, $^{4}J_{CP}$ = 2.9 Hz; P$C_{Ph,para}$), 133.3 (d, $_{2}J_{CP}$ = 10.3 Hz; P$C_{Ph,ortho}$), 139.2 + 140.5 (d + d, $^{1}J_{CP}$ = 87.9 Hz + 95.6 Hz; P$C_{Ph,ipso}$), 147.3 (s, S$C_{Ph,ipso}$) **^{31}P{^{1}H} NMR** (162.1 MHz, d_8-THF): δ_P = 43.1. **^{7}Li NMR** (155.6 MHz, THF): 0.53. **Anal Cald.** For $C_{24}H_{26}ClNNaO_2PS_2$: C 56.08, H 5.10, N 2.73, S 12.47. Found: C 55.80, H 5.41, N 2.73, S 12.22.

Preparation of *R*-1-K

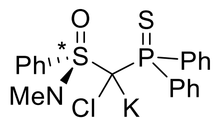

22.2 mg (0.0528 mmol) of *R*-**1** were dissolved in 1 mL of THF and cooled to 0 °C. Subsequently, the solution was added to 6.4 mg (0.16 mmol) KH and stirred at 0 °C for 1 h. The reaction solution was filtered through a cannula, and the solvent was removed *in vacuo*. The product was obtained as a yellow solid in 88 % yield (21.3 mg, 0.046 mmol). Crystals suitable for X-ray diffraction analysis were obtained from a saturated solution of *rac*-**1-K** and 2 eq. of 15-C-5 in THF at –30 °C.

^{1}H NMR (400.3 MHz, d_8-THF): δ_H = 2.68 (s, 3H; NCH_3), 7.13-7.31 (m, 9H; SC$H_{Ph,meta,para}$ + PC$H_{Ph,meta,para}$), 7.85-7.89 (m, 2H, $^{3}J_{HP}$ = 12.9 Hz; PC$H_{Ph,ortho}$), 7.98-8.00 (m, 2H; SC$H_{Ph,ortho}$), 8.05-8.10 (m, 2H; PC$H_{Ph,ortho}$) **^{13}C{^{1}H} NMR** (100.7 MHz, d_8-THF): δ_C = 29.6 (s, NCH$_3$), 49.8 (d, $^{1}J_{CP}$ = 86.3 Hz; *C*Cl), 127.6 + 127.7 (d + d, $^{3}J_{CP}$ = 13.9 Hz; P$C_{Ph,meta}$), 128.2 (S$C_{Ph,ortho}$), 128.5 (s, S$C_{Ph,meta}$), 129.9 (dd, $^{4}J_{CP}$ = 4.13 Hz; P$C_{Ph,para}$ + S$C_{Ph,para}$), 133.1 + 133.2 (d + d, $^{2}J_{CP}$ = 10.2 Hz + 9.7 Hz; P$C_{Ph,ortho}$), 139.5 (d, $^{1}J_{CP}$ = 85.9 Hz; P$C_{Ph,ipso}$), 140.4 (d, $^{1}J_{CP}$ = 90.0 Hz; P$C_{Ph,ipso}$), 146.5 (s, S$C_{Ph,ipso}$) **^{31}P{^{1}H} NMR** (162.1 MHz, d_8-THF): δ_P = 44.1 (s). **Anal. Calcd.** for $C_{20}H_{18}ClKNOPS_2$: C, 52.45; H, 3.96; N, 3,06; S, 14.00. Found: C, 52.4, H, 4.36, N, 3.05, S, 13.78.

2. NMR spectra

2.1 NMR spectra of the isolated compounds

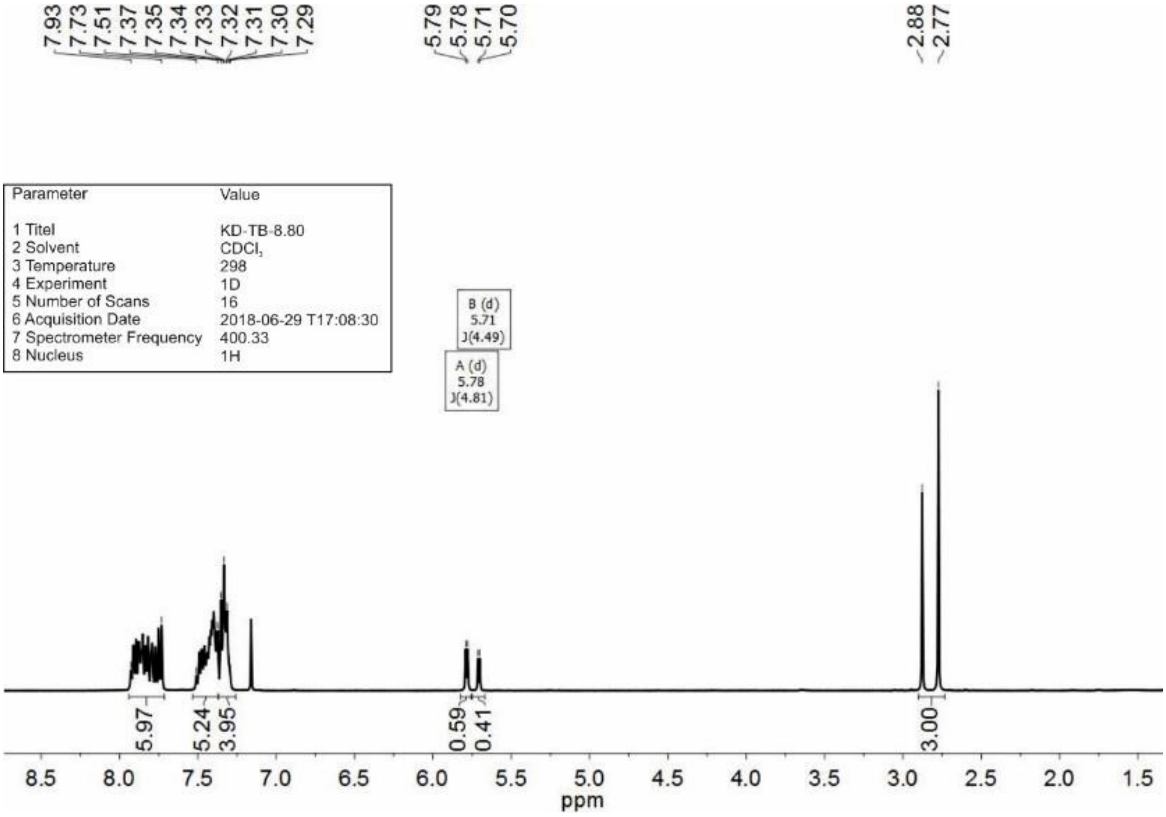

Figure S1. ^{1}H NMR spectrum of *R*-**1** in $CDCl_3$ recorded at room temperature.

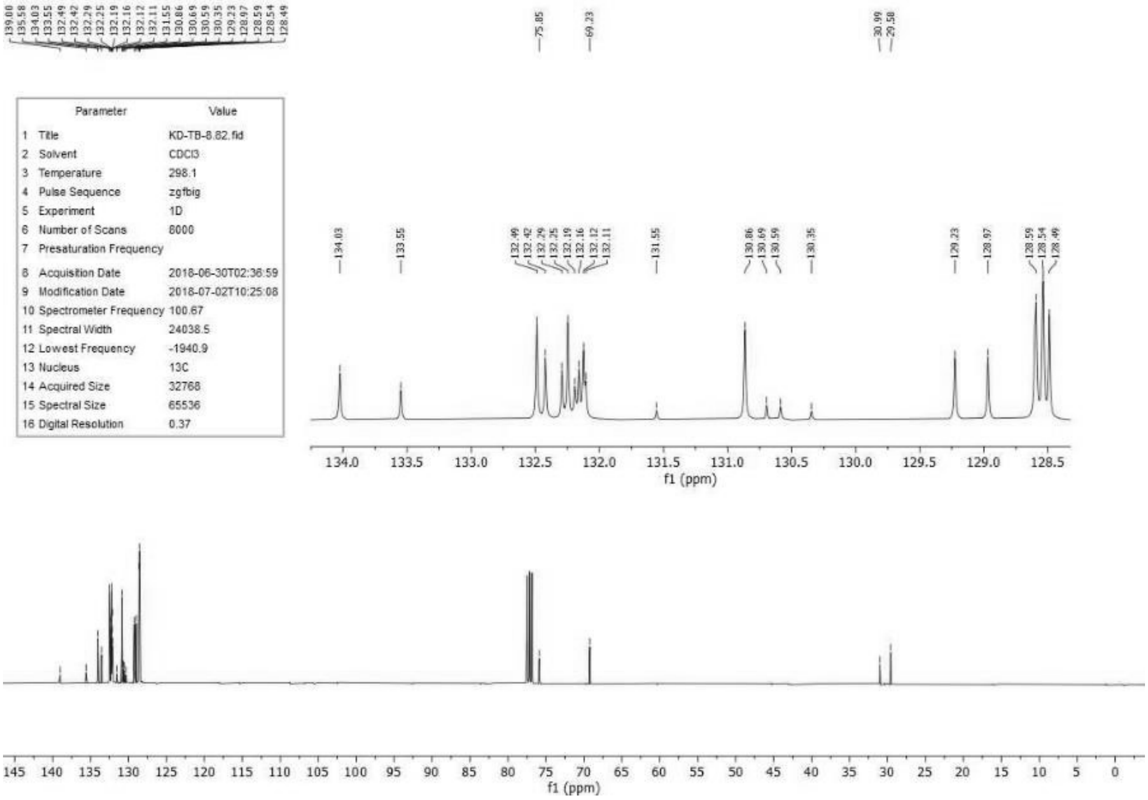

Figure S2. $^{13}C\{^{1}H\}\{^{31}P\}$ NMR spectrum of *R*-**1** in $CDCl_3$ recorded at room temperature.

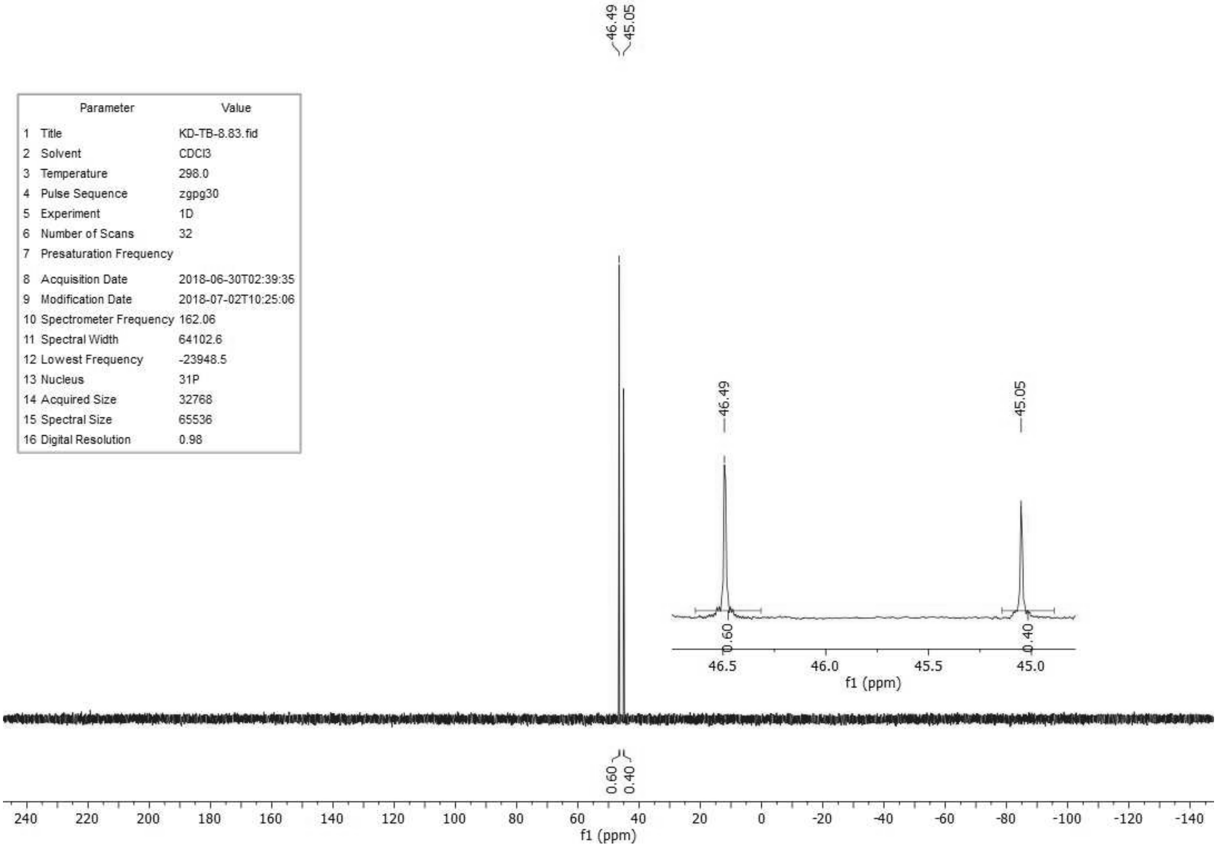

Figure S3. $^{31}P\{^{1}H\}$ NMR spectrum of *R*-**1** in $CDCl_3$ recorded at room temperature.

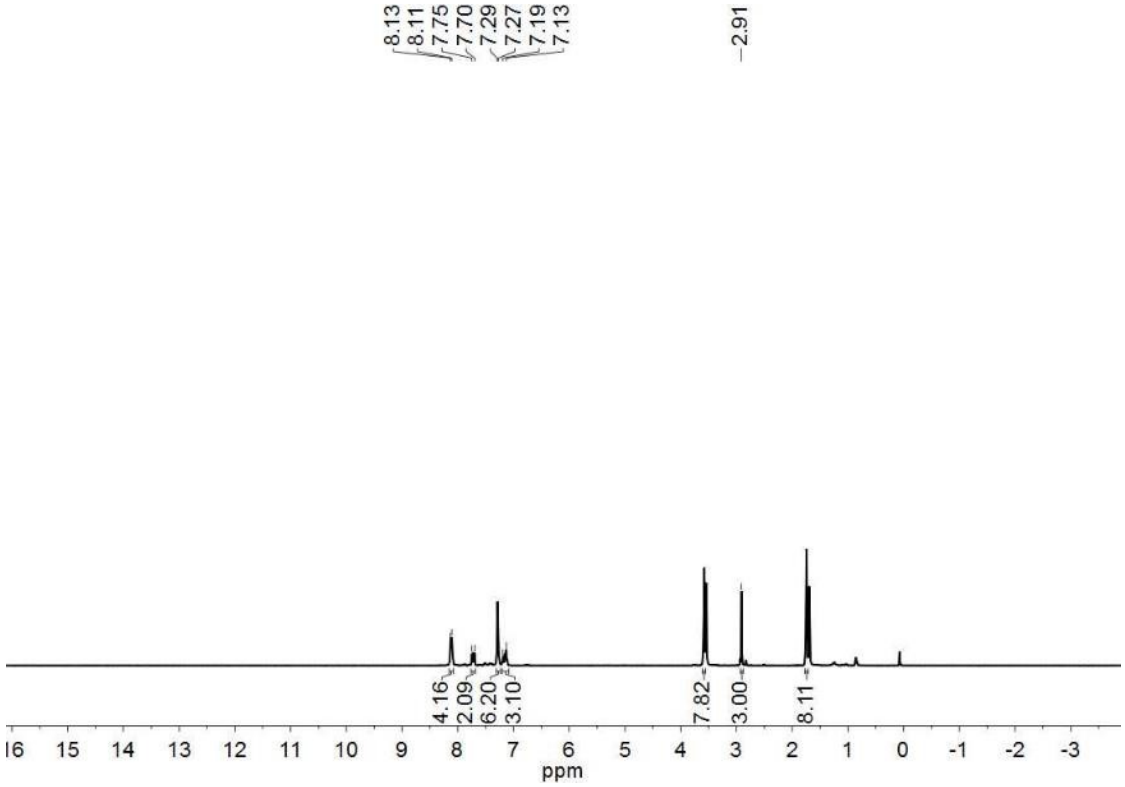

Figure S4. ^{1}H NMR spectrum of *R*-**1-Li·2THF** in d_8-THF recorded at 243 K.

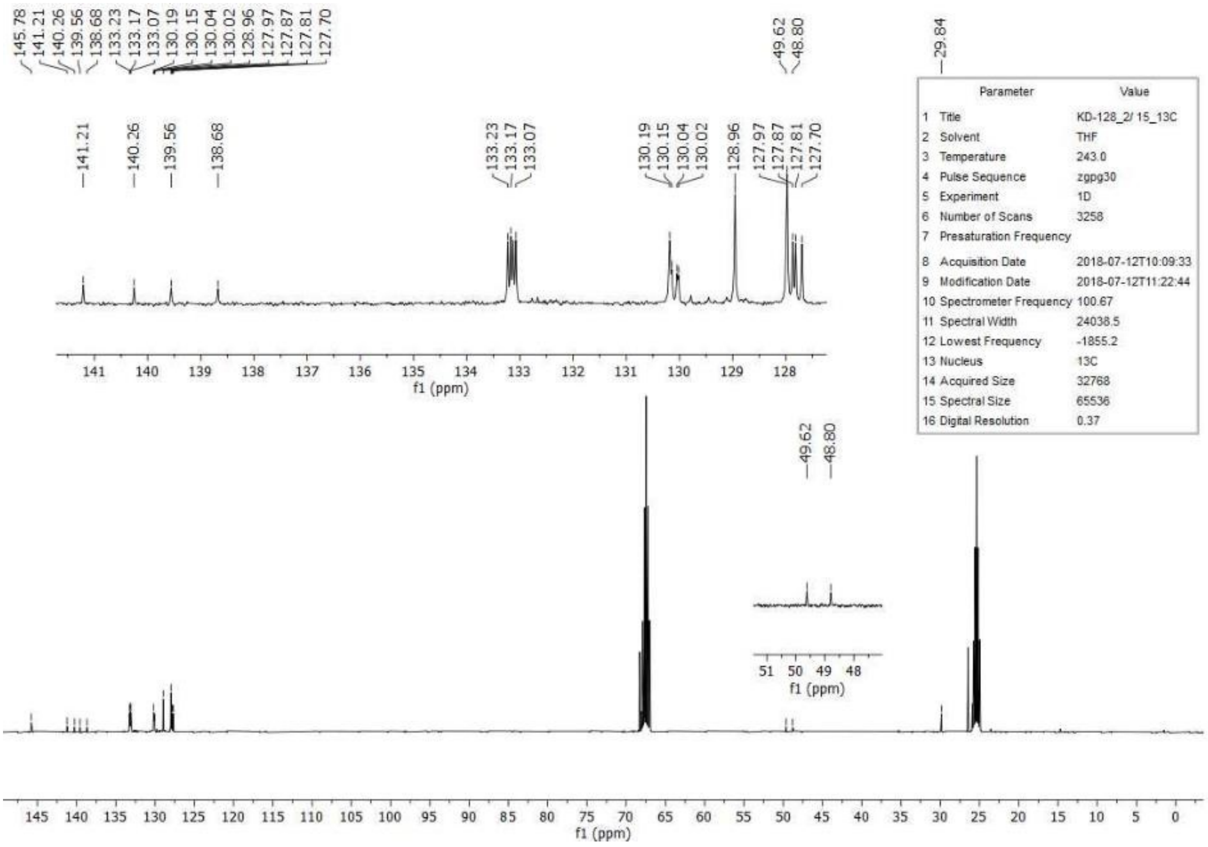

Figure S5. $^{13}C\{^1H\}$ NMR spectrum of *R*-**1-Li·2THF** in d_8-THF recorded at 243 K.

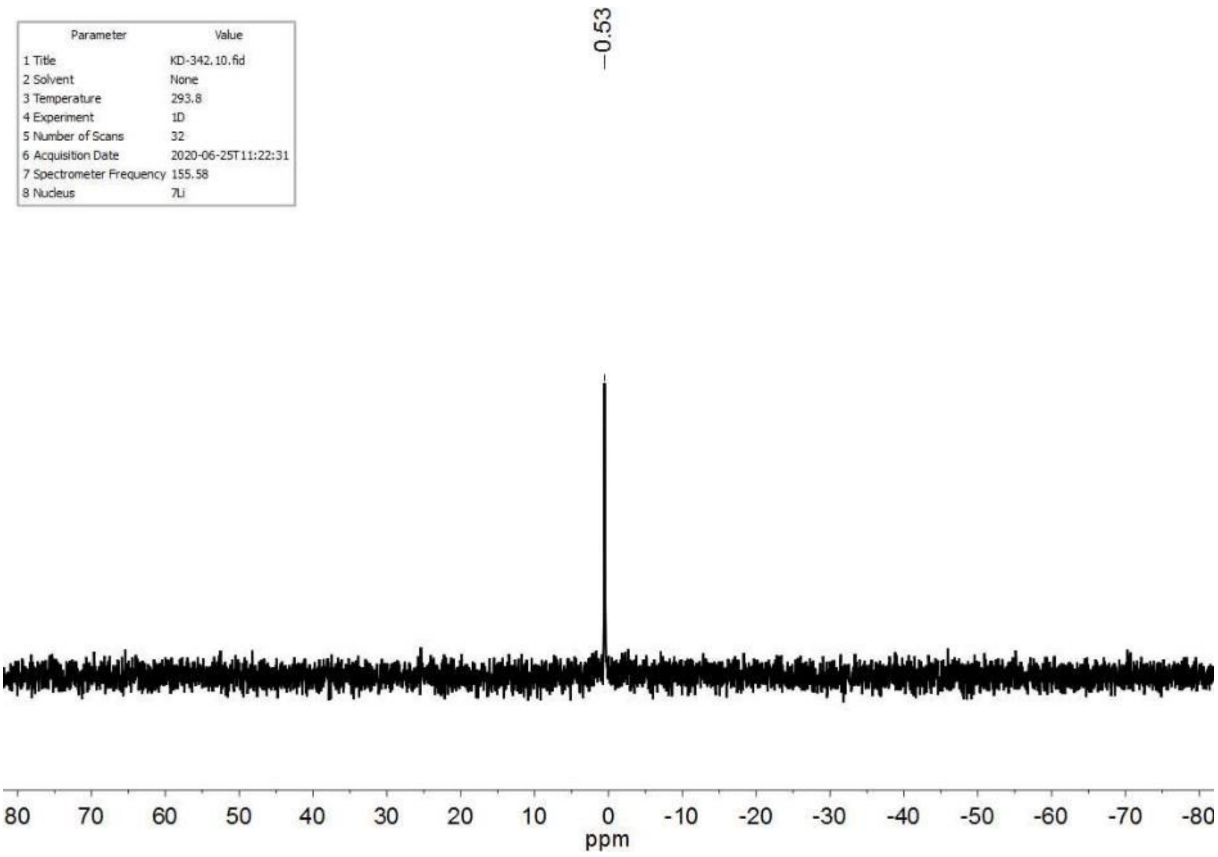

Figure S6. ^7Li NMR spectrum of *R*-**1-Li•2THF** in THF recorded at RT.

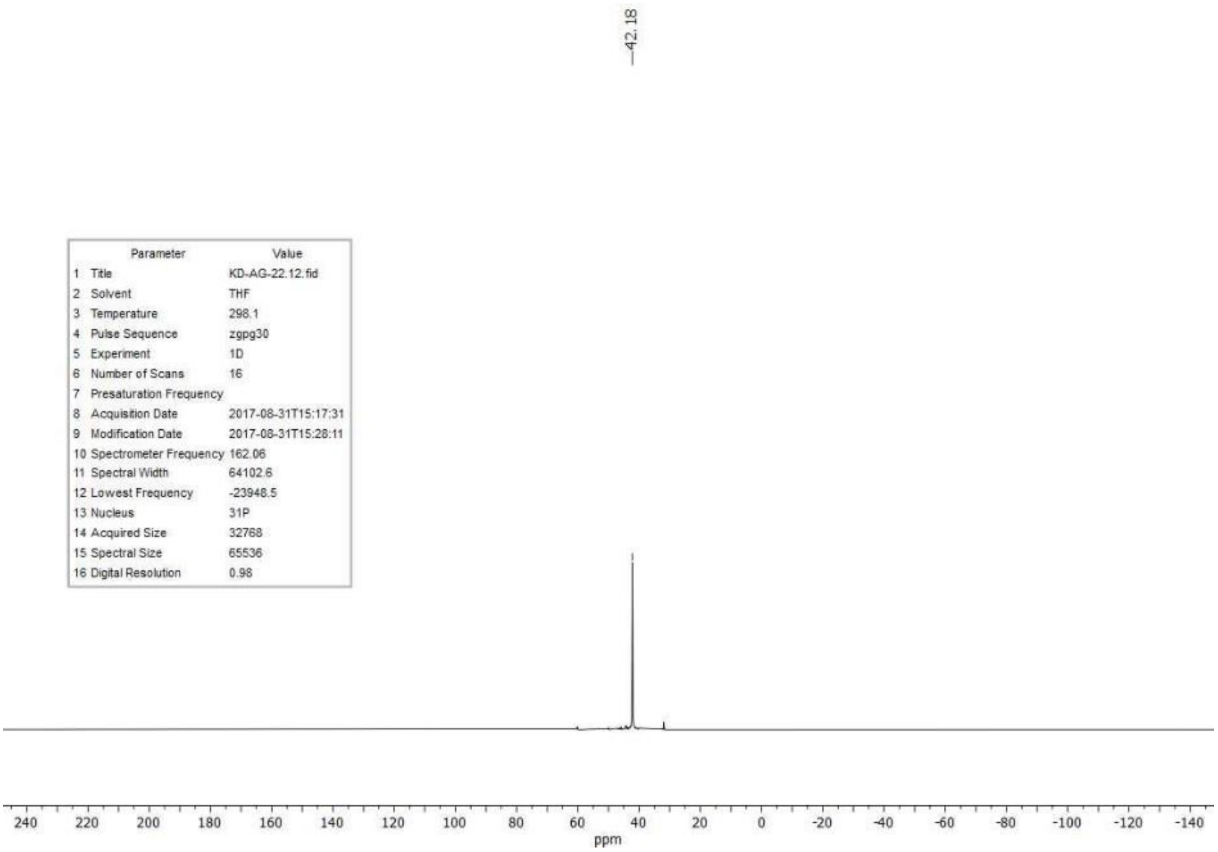

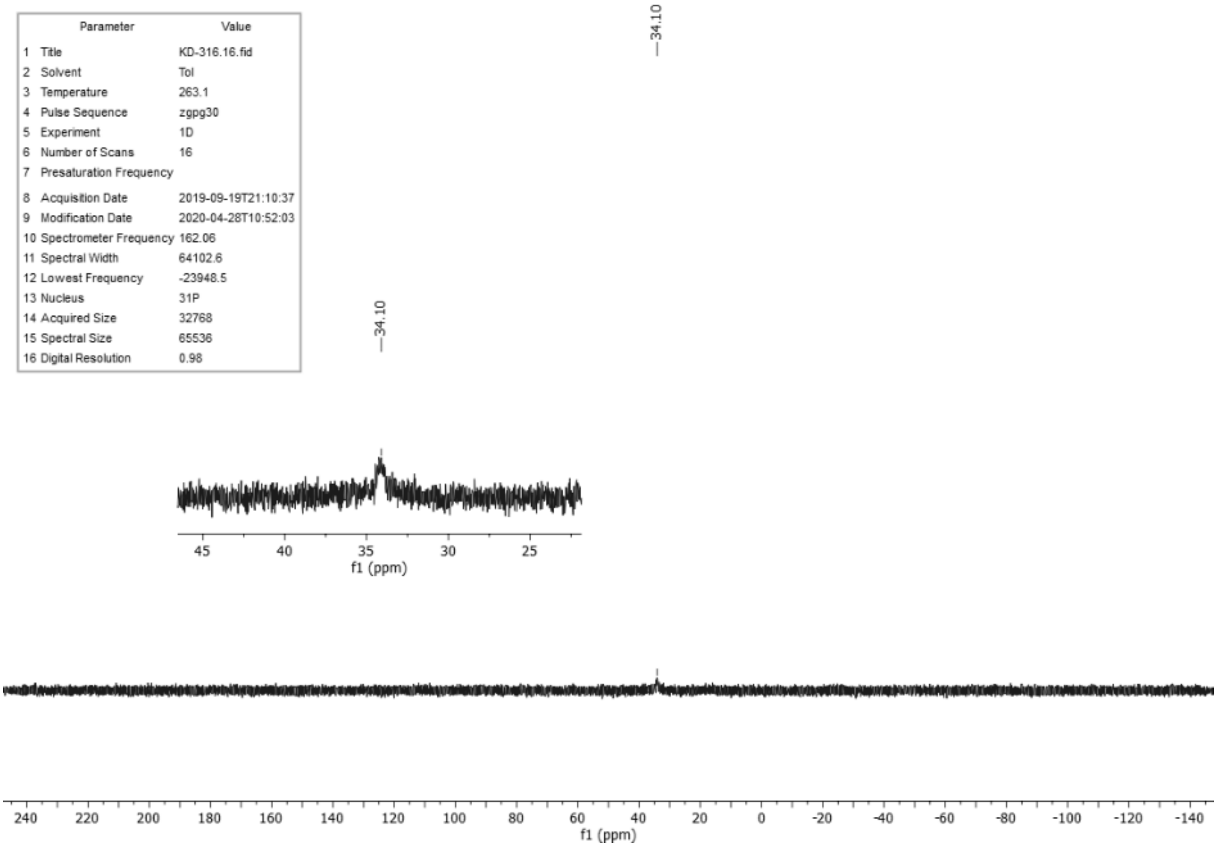

Figure S7. $^{31}P\{^{1}H\}$ NMR spectra of *R*-**1-Li•2THF** in THF recorded at RT (top) and in toluene recorded at 263K (bottom).

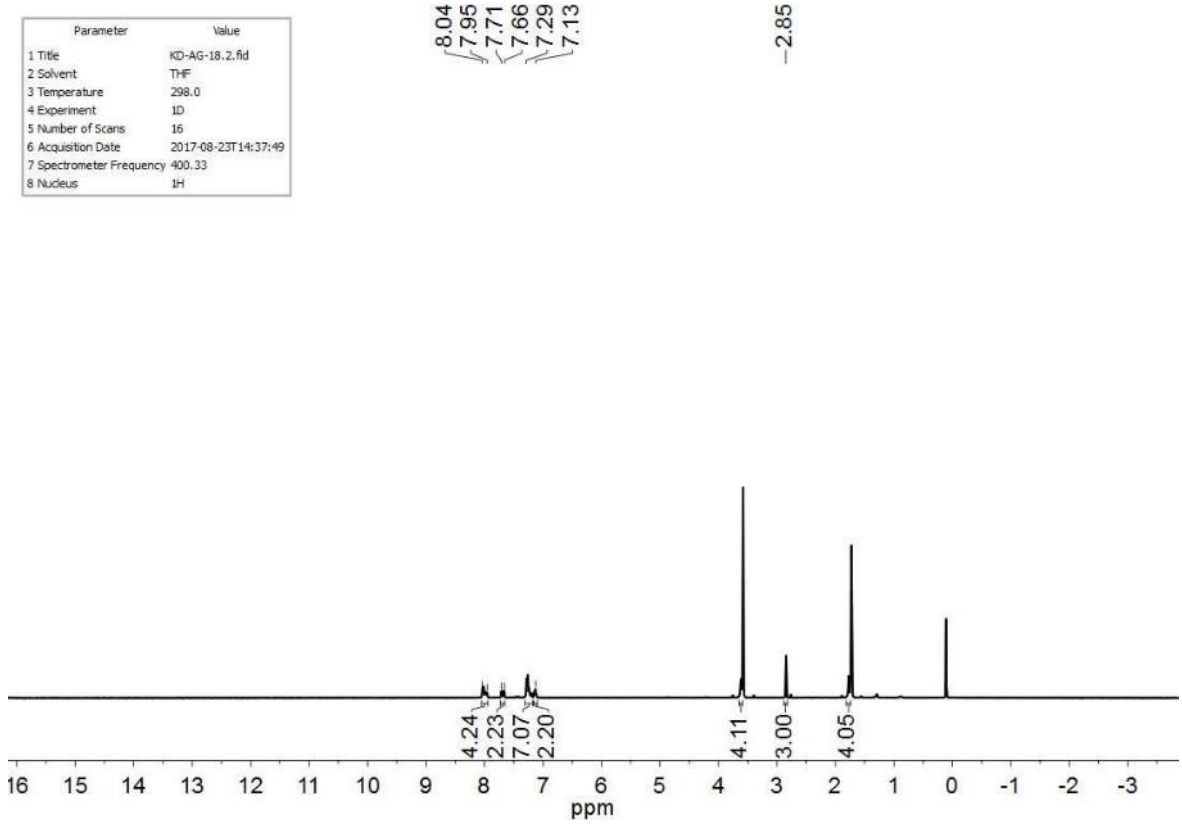

Figure S8. ^{1}H NMR spectrum of *R*-**1-Na·1THF** in d_8-THF recorded at room temperature.

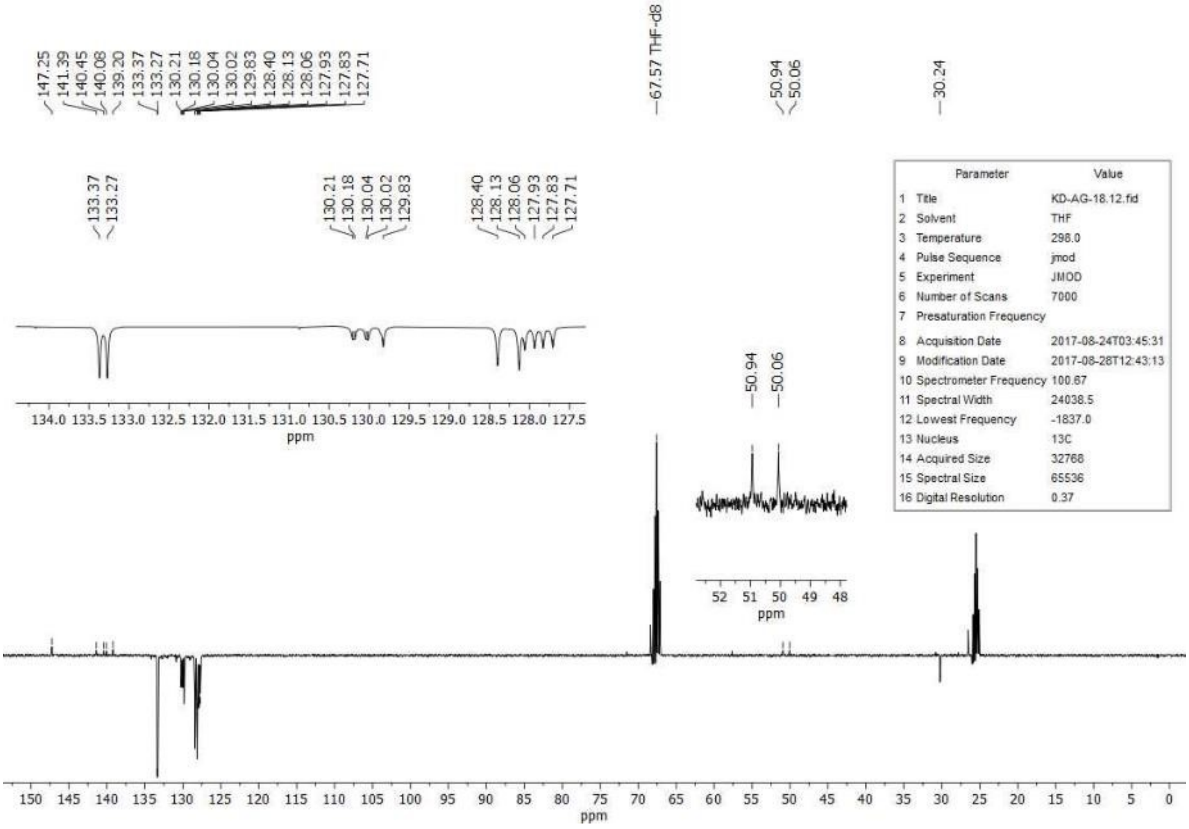

Figure S9. $^{13}C\{^{1}H\}$ NMR spectrum of *R*-**1-Na·1THF** in d_8-THF recorded at room temperature.

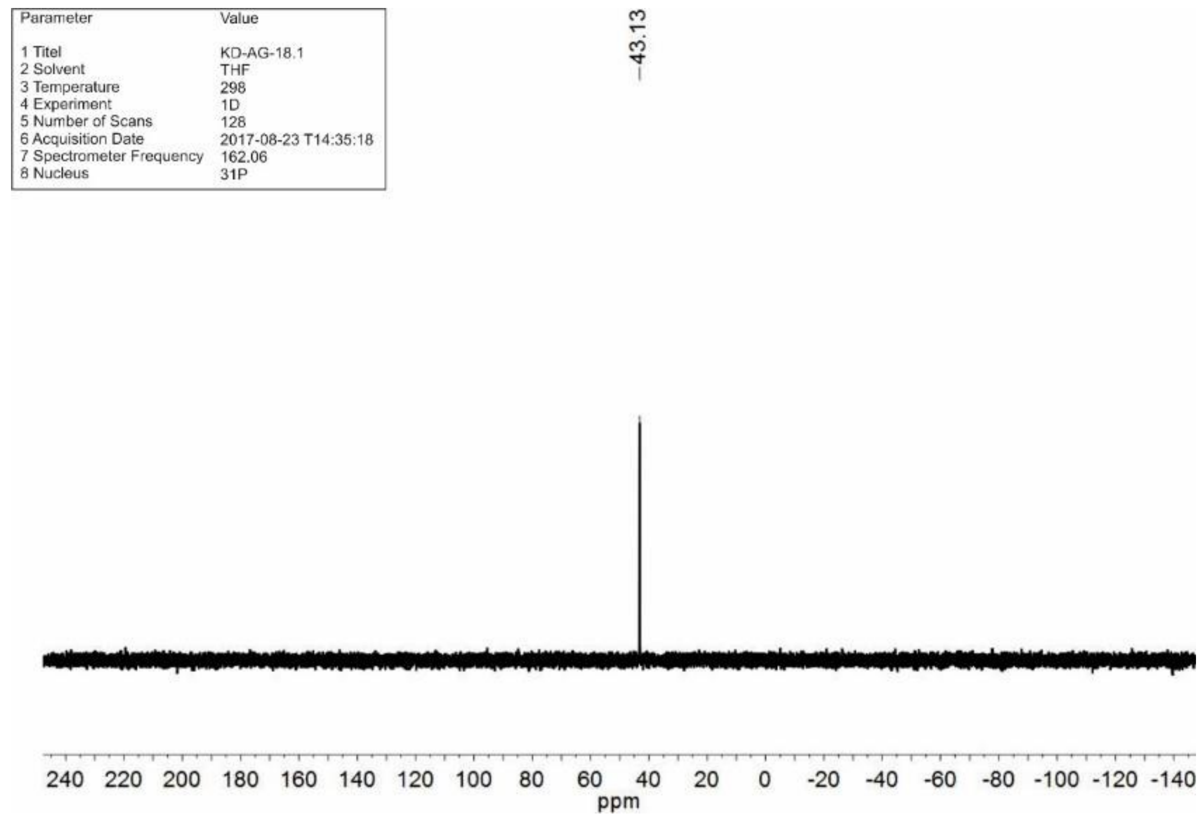

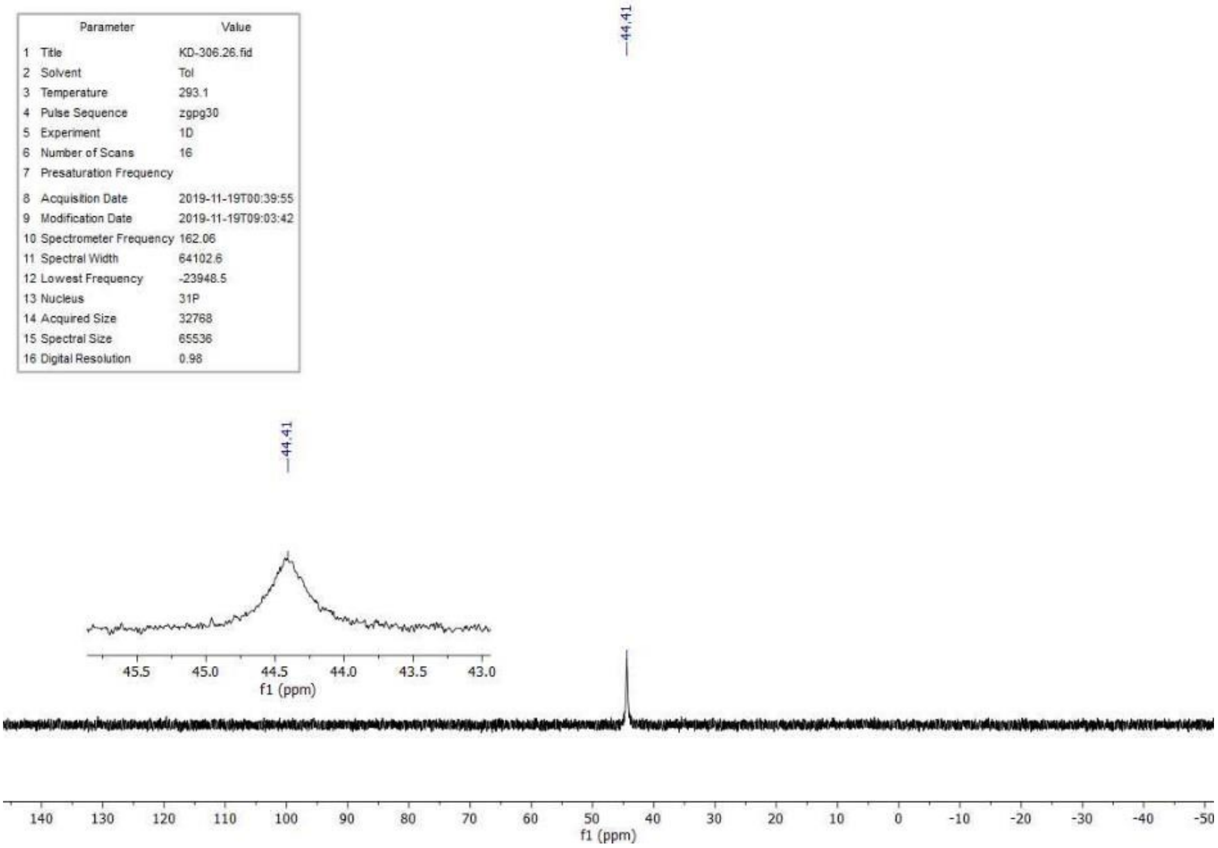

Figure S10. $^{31}P\{^{1}H\}$ NMR spectra of *R*-**1-Na•1THF** in d_8-THF recorded at room temperature (top) and in d^8-toluene at room temperature (bottom).

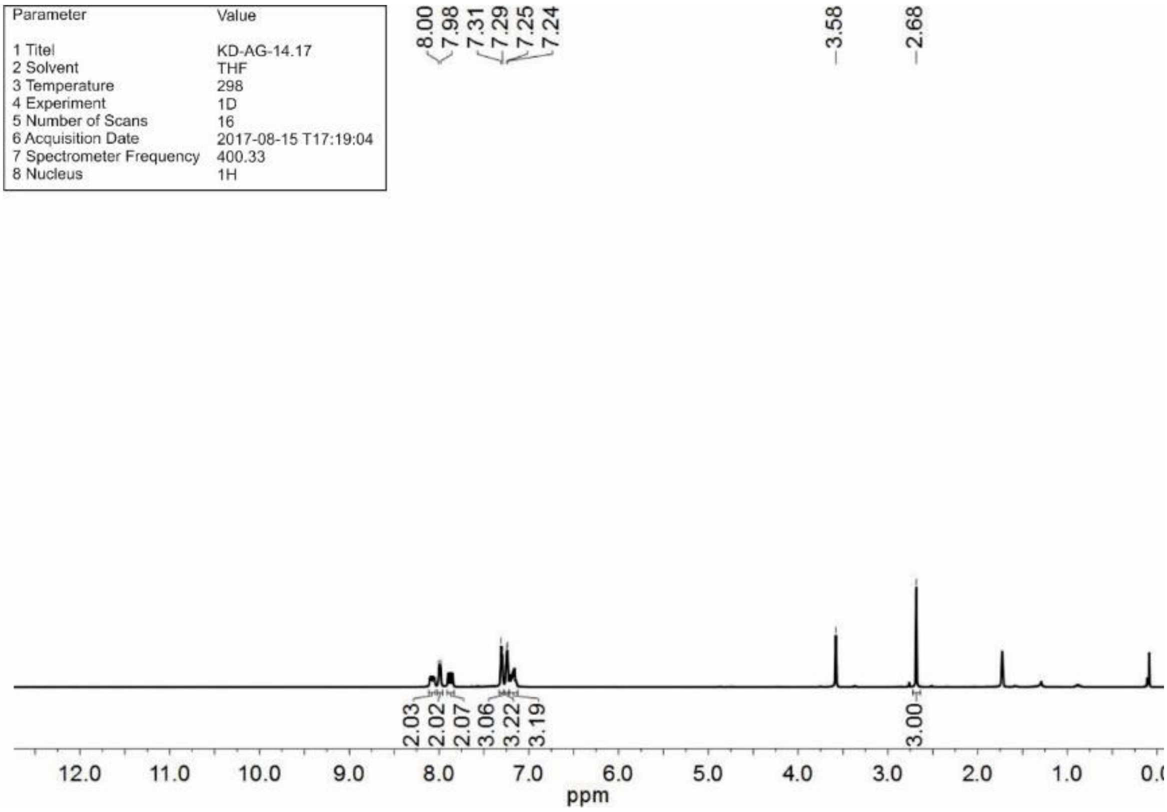

Figure S11. ^{1}H NMR spectra of *R*-**1-K** in d$_8$-THF recorded at room temperature.

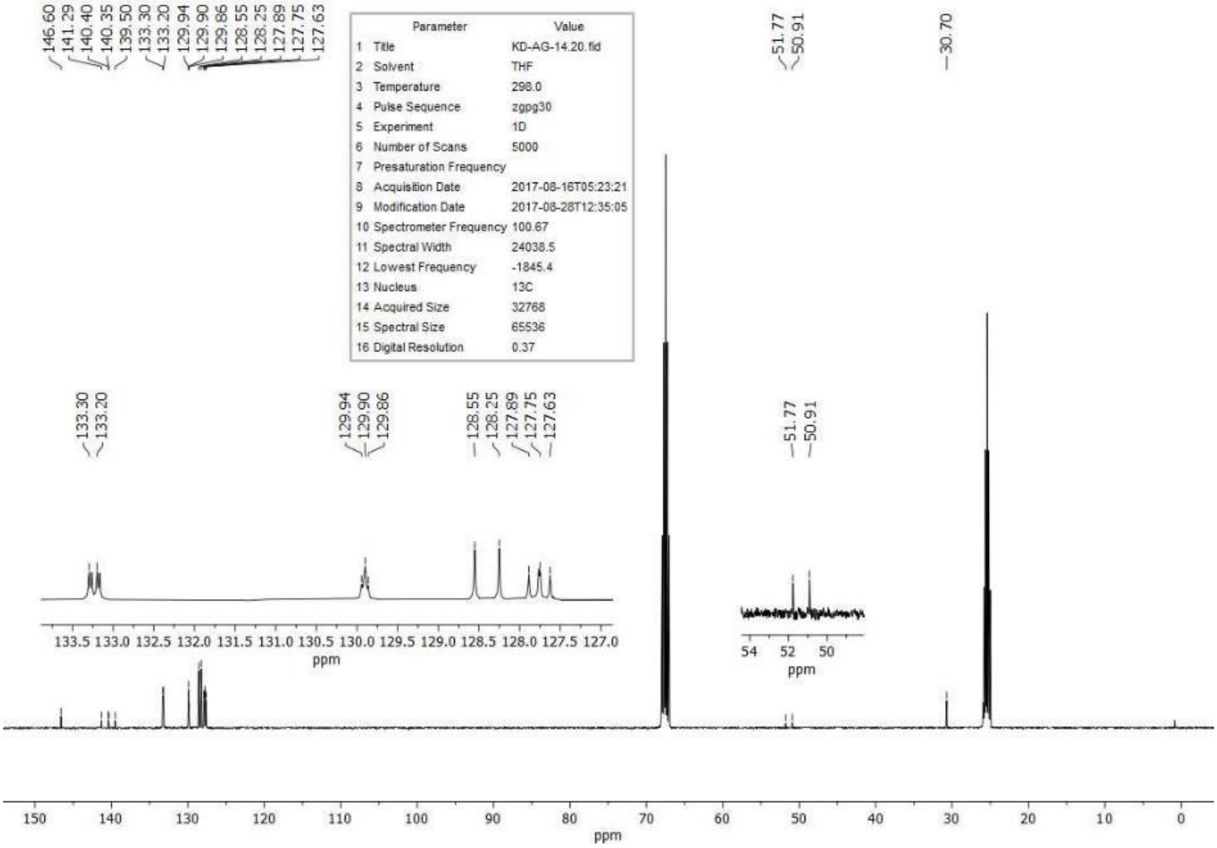

Figure S12. ^{13}C{^{1}H} NMR spectrmum of *R*-**1-K** in d$_8$-THF recorded at room temperature.

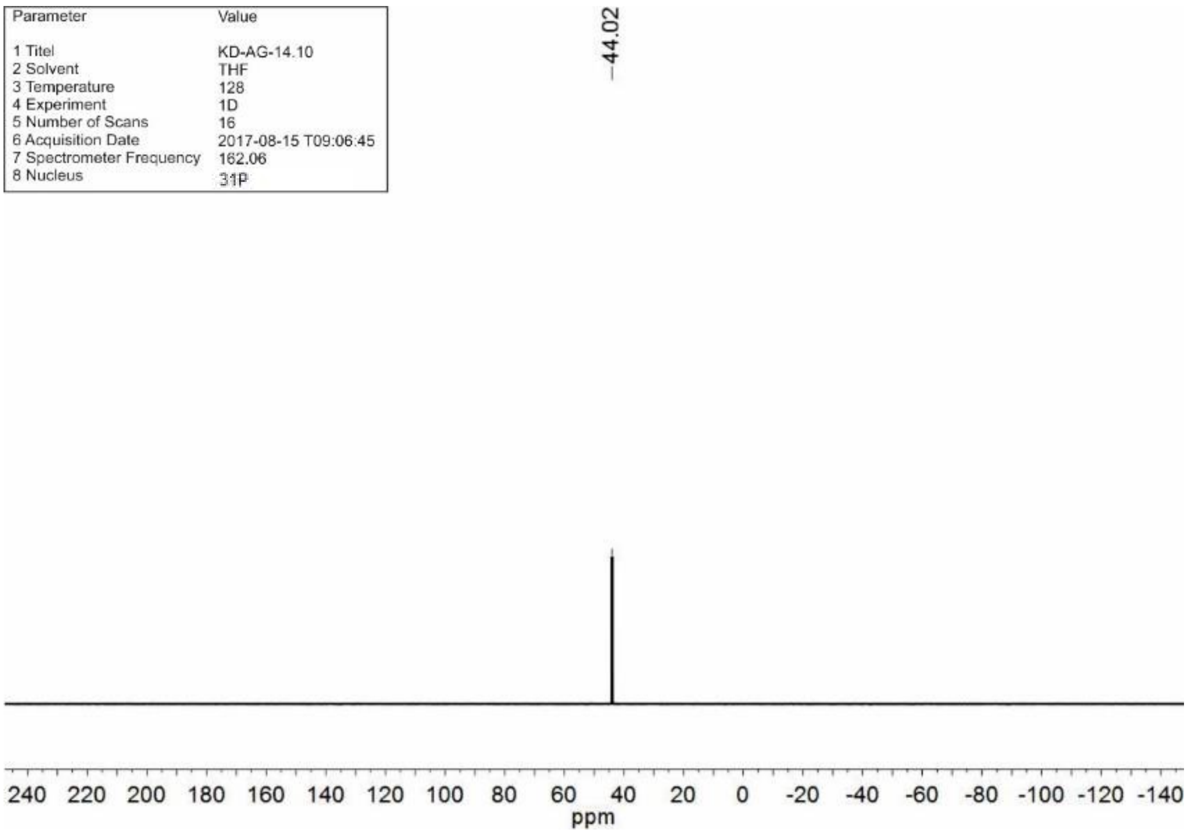

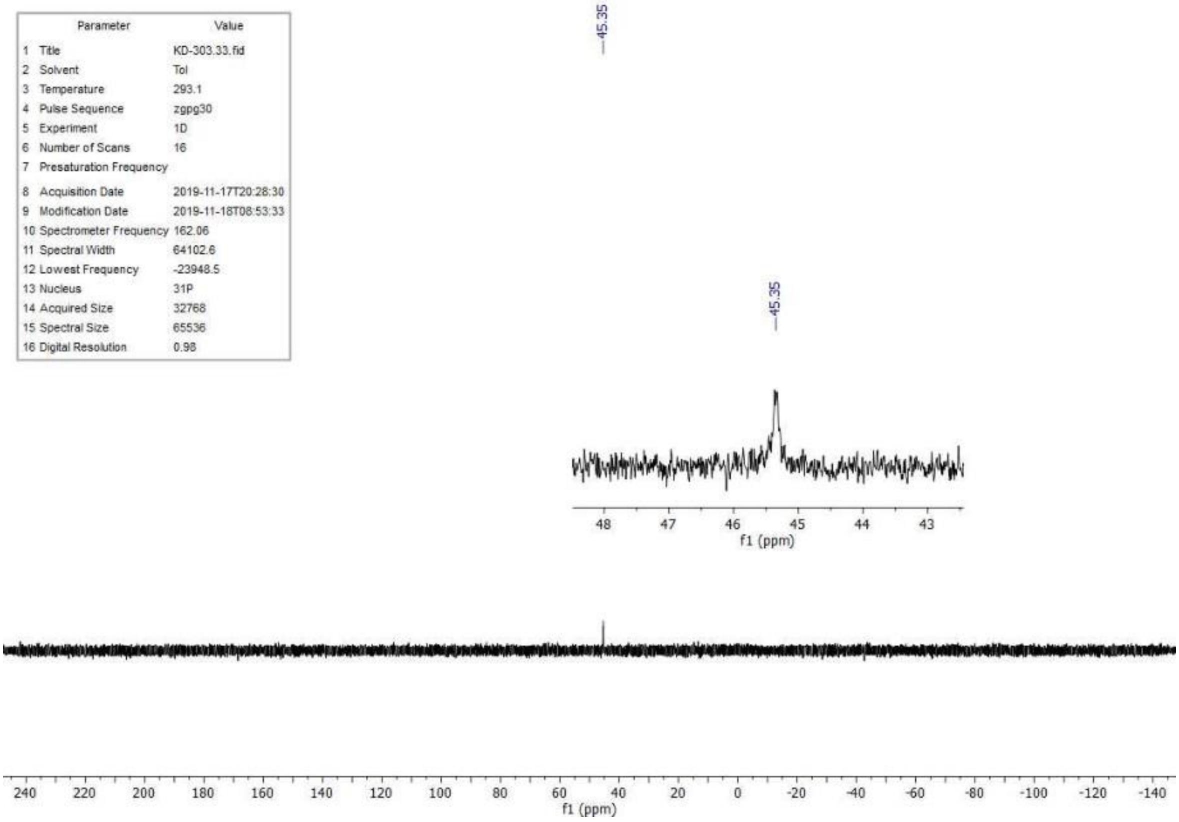

Figure S13. $^{31}P\{^1H\}$ NMR spectra of *R*-**1-K** in d_8-THF recorded at room temperature (top) and in d^8-toluene at room temperature (bottom).

2.2 VT $^{31}P\{^{1}H\}$ NMR spectroscopy

The carbenoids were prepared according to the synthesis stated above. A J. Young NMR tube was set under vacuum and flushed with Ar three times and cooled to –40 °C. The deuterated solvent (d_8-THF or d_8-Tol) was cooled to –40 °C. 0.6 mL of cooled deuterated solvent was added to the Schlenk flask which was also cooled to –40 °C and the carbenoids were dissolved and subsequently, transferred *via* a syringe to the J. Young NMR tube. The NMR tube was cooled while transporting to the NMR instrument

NMR instrument: The NMR instrument is cooled to –30 °C prior to the injection of the sample to prevent sample decomposition. The VT NMR studies are performed in automation starting at –30 °C and incrementing the temperature by 10 °C steps with a start delay of 3515 sec. until a temperature of 50 °C is reached.

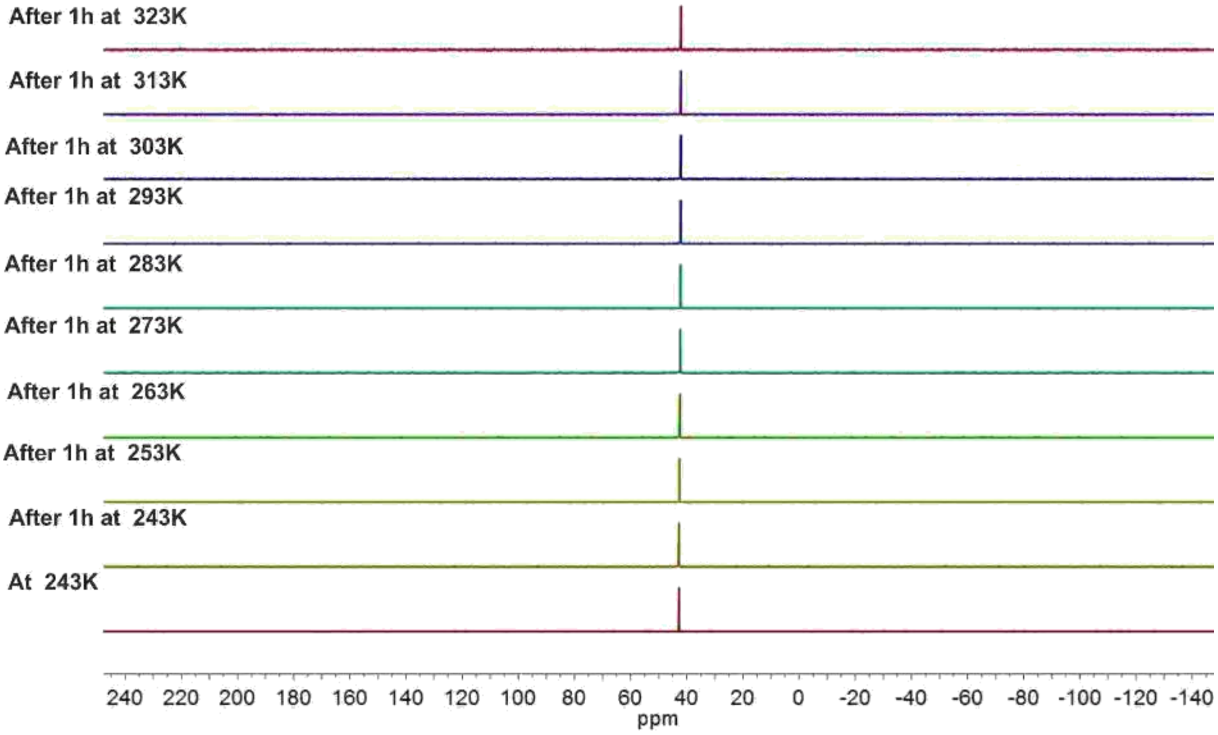

Figure S14. VT $^{31}P\{^{1}H\}$ NMR Spectra of *rac*-**1-Li** in d_8-THF.

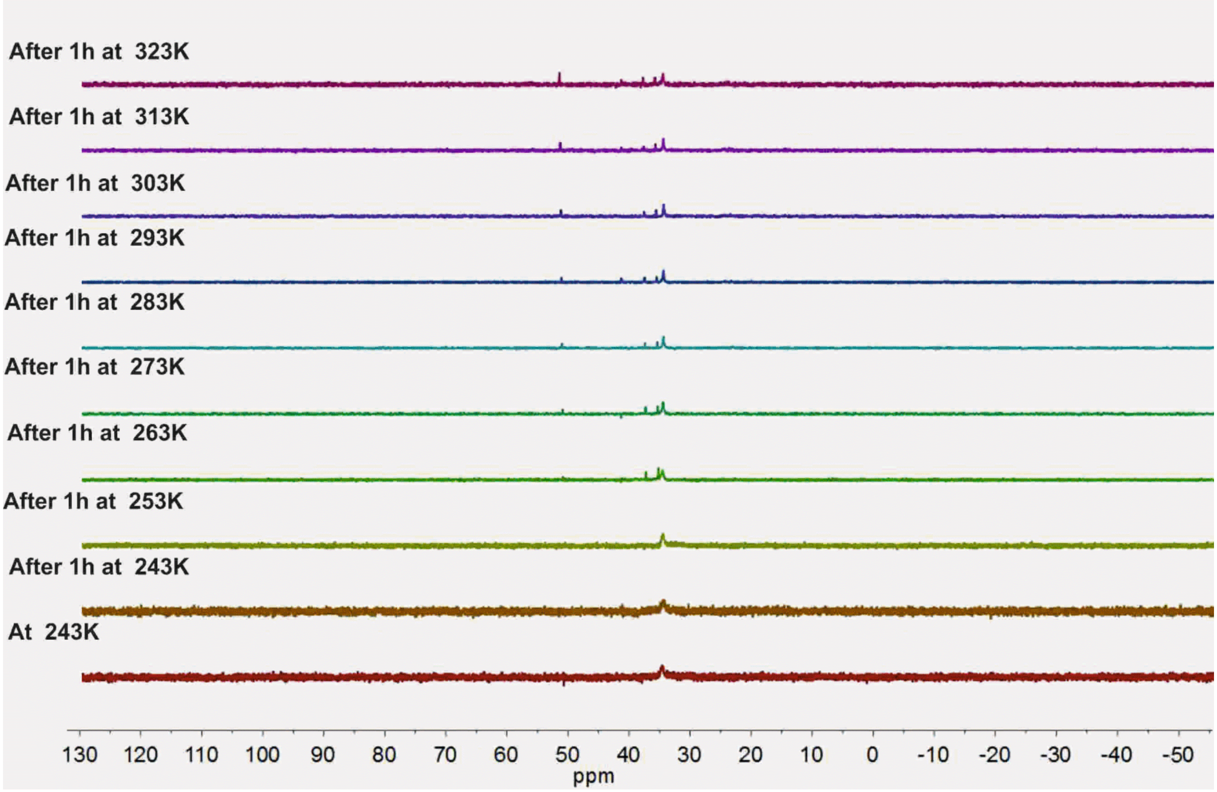

Figure S15. VT $^{31}P\{^{1}H\}$ NMR Spectra of *rac*-**1-Li** in d_8-Tol.

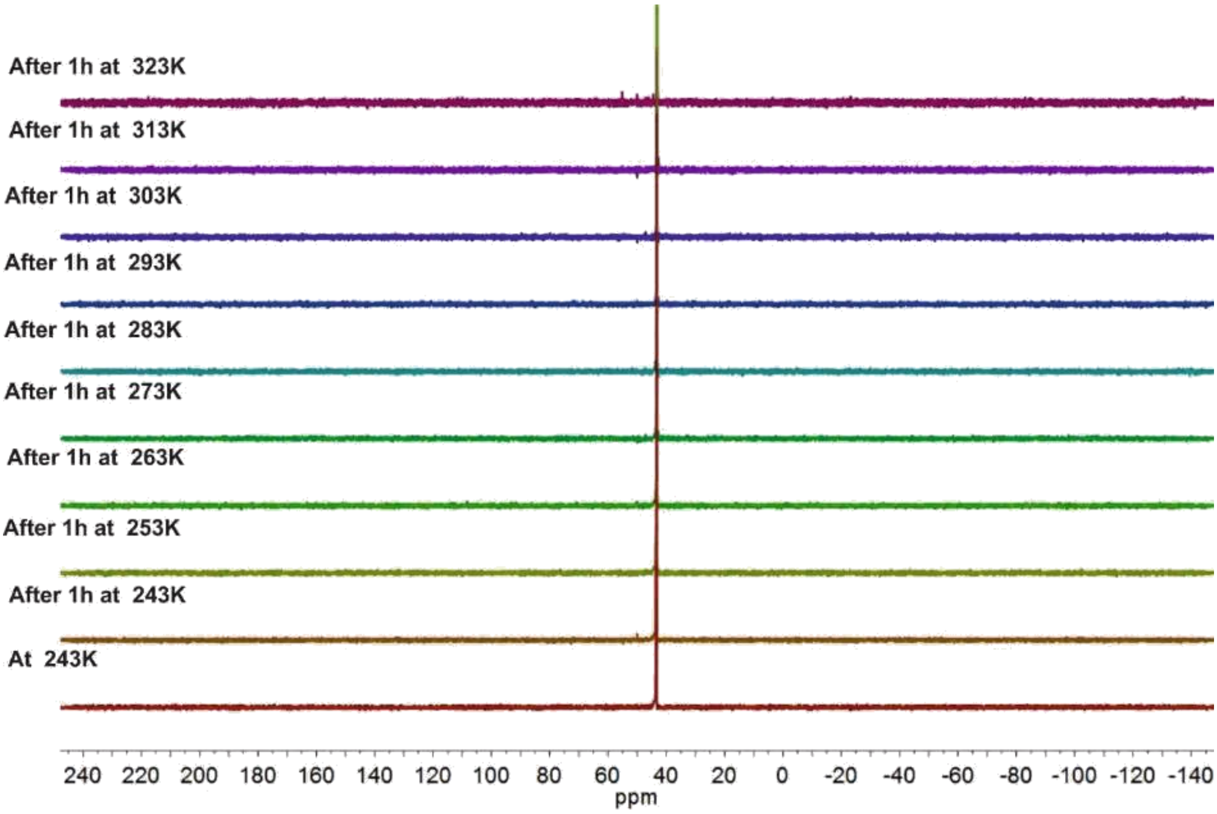

Figure S16. VT $^{31}P\{^{1}H\}$ NMR Spectra of *rac*-**1-Na** in d_8-THF.

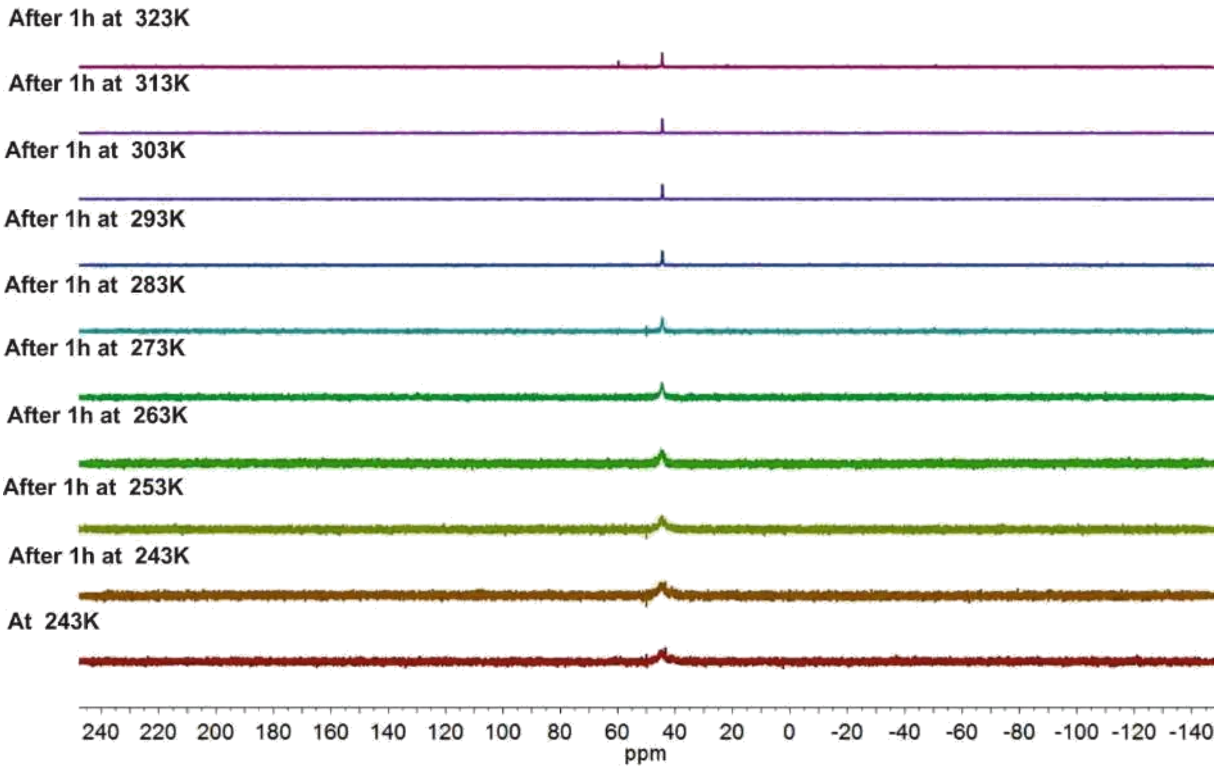

Figure S17. VT $^{31}P\{^1H\}$ NMR Spectra of *rac*-**1-Na** in d_8-Tol.

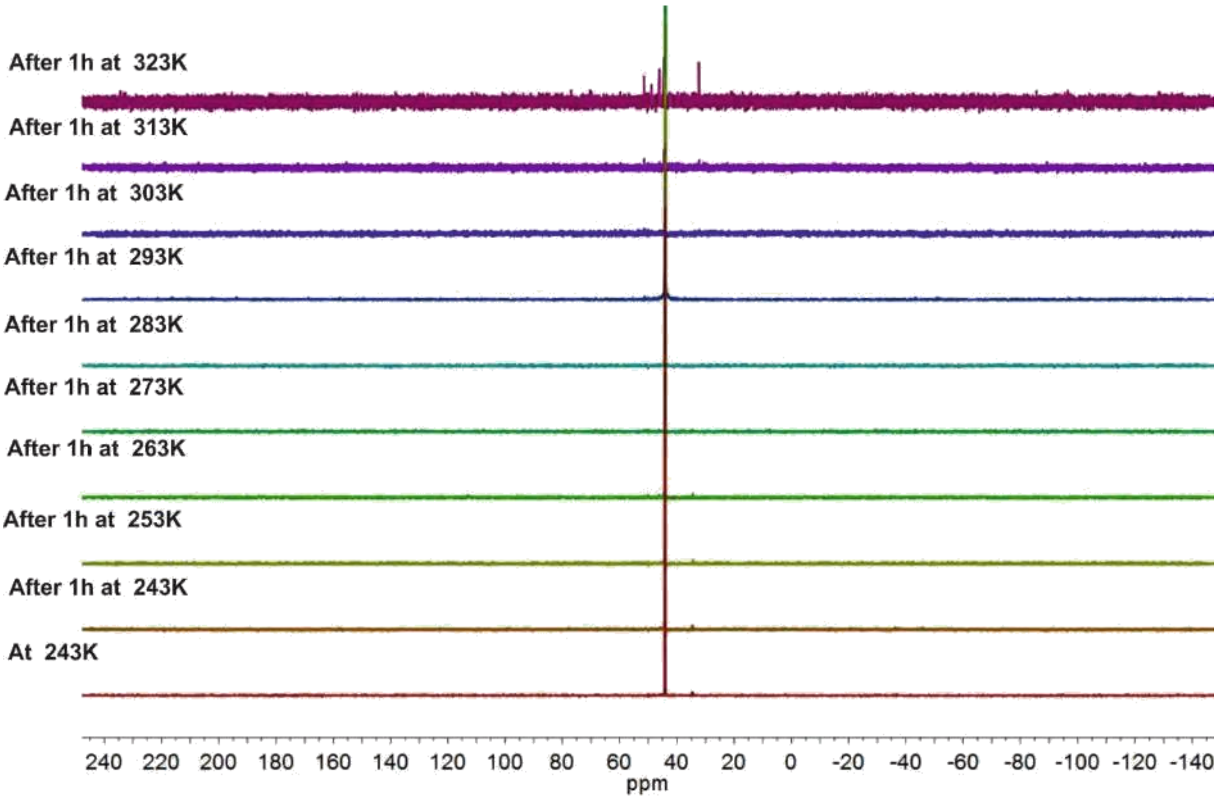

Figure S18. VT $^{31}P\{^1H\}$ NMR Spectra of *rac*-**1-K** in d_8-THF.

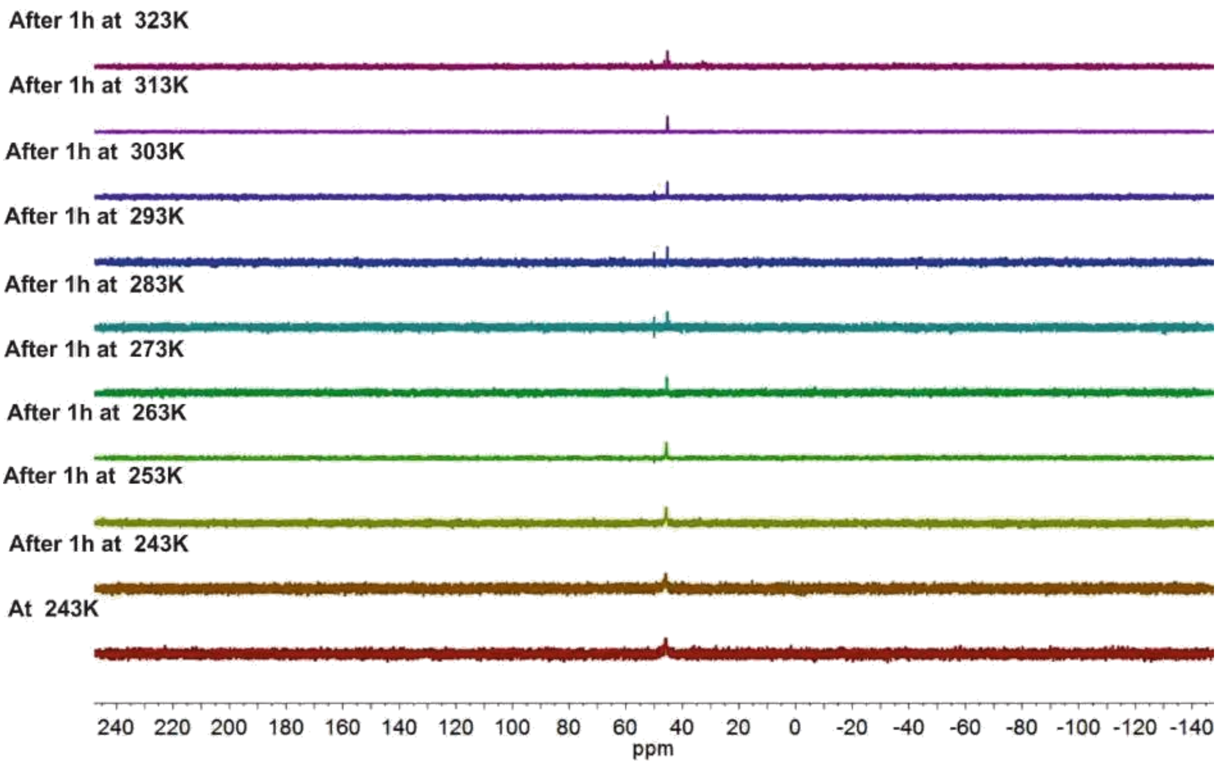

Figure S19. VT $^{31}P\{^1H\}$ NMR Spectra of *rac*-**1-K** in d_8-Tol.

3. DOSY NMR spectroscopy

3.1 General procedure

General procedure of the sample preparation for DOSY NMR spectroscopic measurements.

DOSY measurements were recorded on an AV 400 MHz spectrometer operating at 400.13 MHz. A double stimulated echo sequence (dstebpg3s) was used and the pulse gradients (g) were incremented from 2 to 95% of the maximum gradient strength in a linear ramp. The Stejskal-Tanner diffusion delay (d20) was set to 0.2 s and the eddy-current delay (d21) to 5 ms. After Fourier transformation and baseline correction, the diffusion dimension was processed with the Topspin 3.6.1 software (BrukerBiospin). Diffusion coefficients were calculated by exponential fits with the T1/T2 software of Topspin. Tetramethylsilane and adamantane have been used as references in DOSY measurements in THF and toluene, respectively. The molecular masses were calculated by the ECC-MW estimation software by Stalke *et al*.[143]

All carbenoids were prepared according to the synthetic procedure stated above with 0.075 mmol of the protonated precursor: The corresponding metal base was added to a THF solution of the protonated precursor. After stirring for 1h at that temperature, the solvent was removed in vacuo. The obtained solid was subsequently dissolved in 0.6 mL of cooled deuterated solvent and transferred via a syringe into a J. Young NMR tube with the corresponding internal standard and used for DOSY NMR spectroscopy measurements. The THF used for synthesis could not be removed completely in vacuo and residual THF was still present during the DOSY measurements (see above).

Measurements in d_8-toluene:

4 mg (0.03 mmol) adamantane were added to a J. Young NMR tube and the NMR tube was evaporated and flushed with Ar three times. 0.6 mL cooled (–40 °C) d_8-Tol was added to the cooled (–40 °C) Schlenk flask containing the respective carbenoid. The carbenoid was prepared as stated above, dissolved in d_8-Tol and subsequently transferred to the J. Young NMR tube *via* a syringe under Ar. The J. Young NMR tube was cooled to –40 °C and the sample was kept at that temperature while transferring it to the NMR instrument.

Measurements in d_8-THF:

A J. Young NMR tube was evaporated and flushed with Ar three times. 0.6 mL d_8-THF cooled to –40 °C were added to the cooled (–40 °C) Schlenk flask containing the carbenoid. The J. Young NMR tube is placed in a cooling bath at –40 °C and the d_8-THF solution of the carbenoid (prepared as stated above) is transferred to the J. Young NMR tube. Then, 4 µL TMS (0.03 mmol) were added to the J. Young tube under Ar. The sample was kept at –40 °C while transferring it to the NMR instrument.

NMR instrument:

The NMR instrument is cooled to –30 °C prior to the injection of the sample to prevent sample decomposition. The DOSY NMR spectra are recorded at -30 °C -10 °C and 27 °C, respectively. The instrument is warmed up to the respective temperature manually.

3.2 DOSY NMR data of *rac*-1-Li

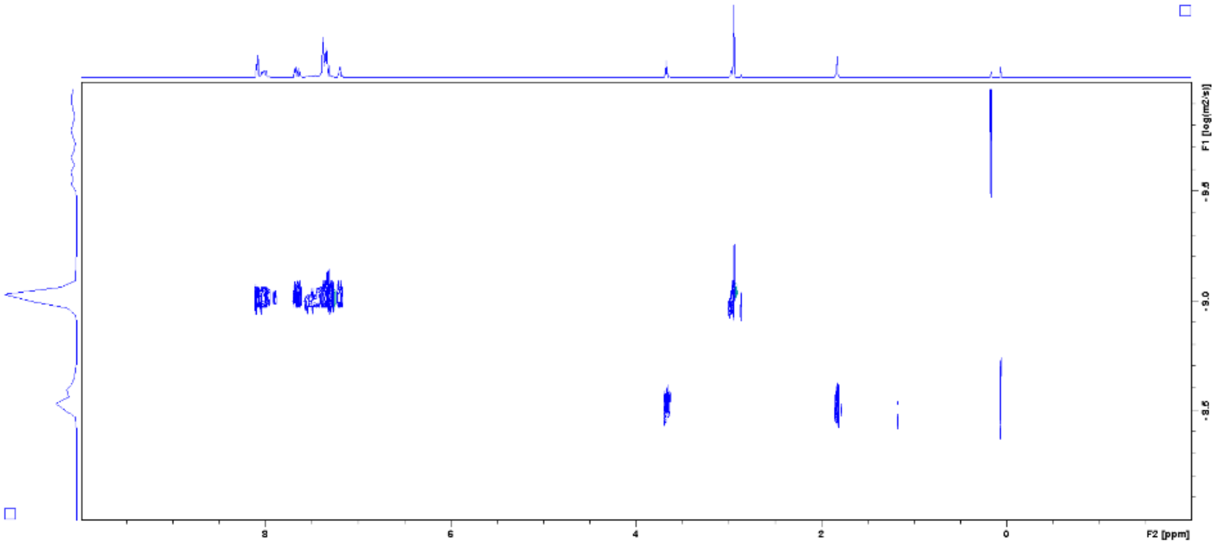

Figure S20. ^{1}H DOSY NMR spectrum for *rac*-**1-Li** in d_8-THF at 27 °C.

Table S1. ^{1}H DOSY NMR data for *rac*-**1-Li** at 27 °C in d_8-THF.

Solvent	Int. reference	D_{ref} [m^2s^{-1}]	$logD_{ref}$	Ø $logD_{Carb}$	MW_{DOSY} [g/mol]	MW_{calc} [g/mol]	Species	Error [%]
THF	TMS	2.21E-9	-8.67	-9.05	464	497.96	Monomer +1THF	-6.8

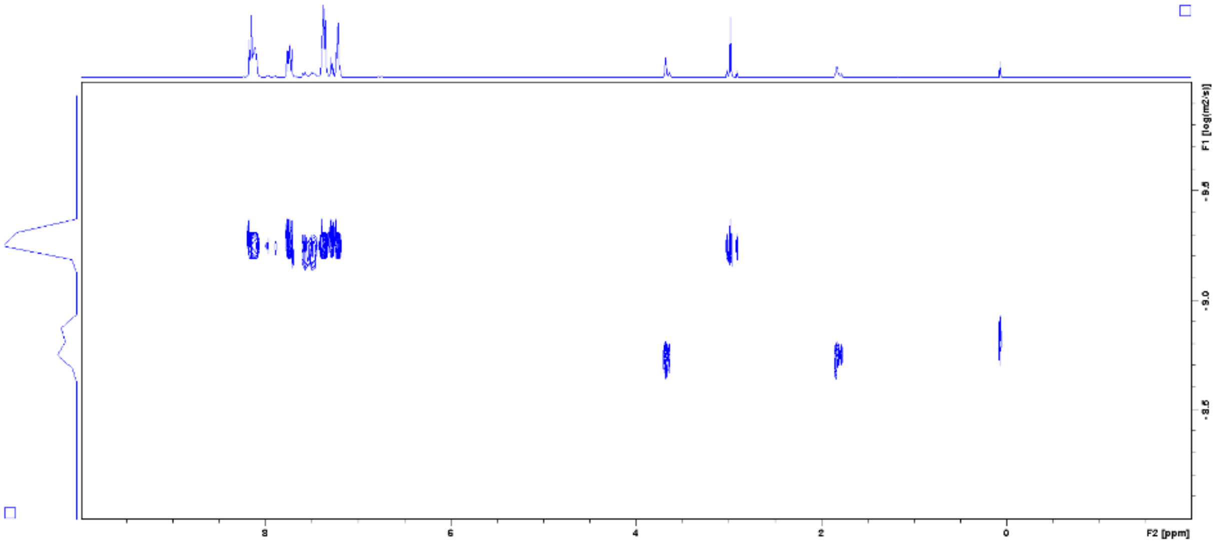

Figure S21. ^{1}H DOSY NMR spectrum for *rac*-**1-Li** in d_8-THF at –10 °C.

Table S2. ^{1}H DOSY NMR data for *rac*-**1-Li** at –10 °C in d_8-THF.

Solvent	Int. reference	D_{ref} [m^2s^{-1}]	$logD_{ref}$	Ø $logD_{Carb}$	MW_{DOSY} [g/mol]	MW_{calc} [g/mol]	Species	Error [%]
THF	TMS	1.28E-9	-8.89	-9.33	554	570.07	Monomer + 2 THF	-2.8

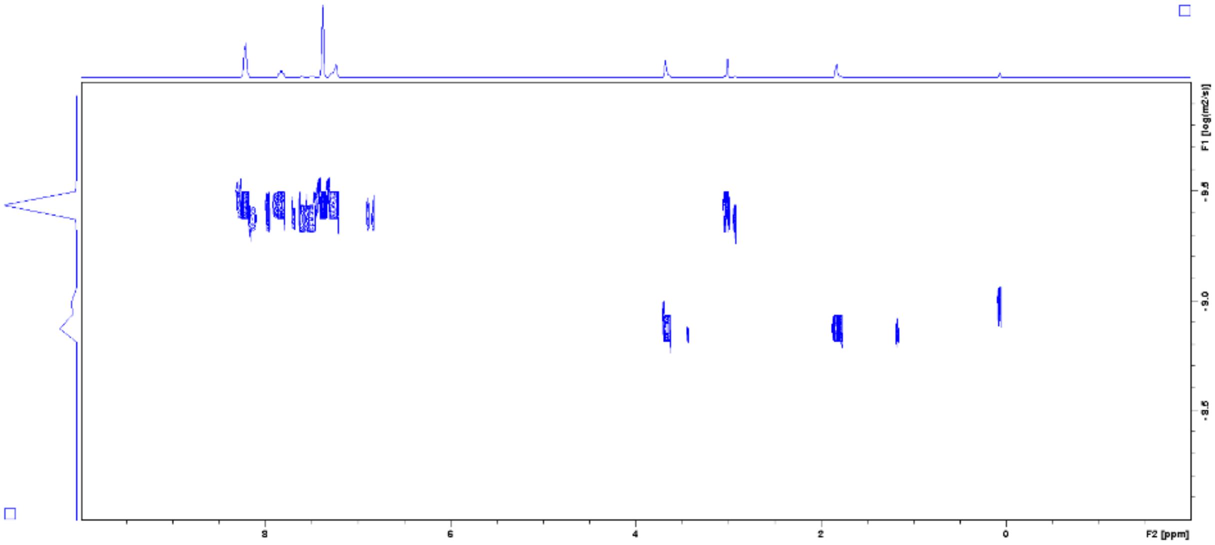

Figure S22. ^{1}H DOSY NMR spectrum for *rac*-**1-Li** in d_8-THF at –10 °C.

Table S3. ^{1}H DOSY NMR data for *rac*-**1-Li** at –30 °C in d_8-THF.

Solvent	Int. reference	D_{ref} [m^2s^{-1}]	$logD_{ref}$	Ø $logD_{Carb}$	MW_{DOSY} [g/mol]	MW_{calc} [g/mol]	Species	Error [%]
THF	TMS	8.75E-10	-9.06	-9.51	600	570.07	Monomer + 2 THF	+ 5.3
						642.17	Monomer + 3 THF	+ 6.6

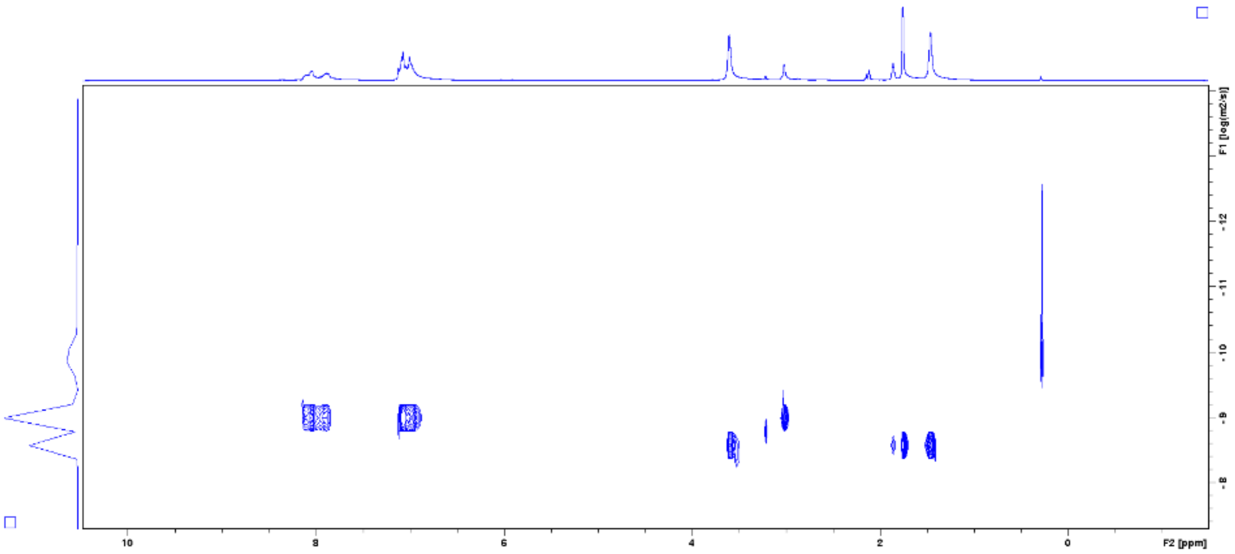

Figure S23. ^{1}H DOSY NMR spectrum for *rac*-**1-Li** in d_8-Tol at 27 °C.

Table S4. ^{1}H DOSY NMR data for *rac*-**1-Li** at 27 °C in d$_8$-Tol.

Solvent	Int. reference	ØD_{ref} [m^{2}s^{-1}]	ØlogD_{ref}	Ø logD_{Carb}	MW_{DOSY} [g/mol]	MW_{calc} [g/mol]	Species	Error [%]
Tol	Adam	1.69E-9	-8.77	-9.25	904	851.71	Dimer + 0 THF	+6.1
						923.81	Dimer + 1 THF	-2.1

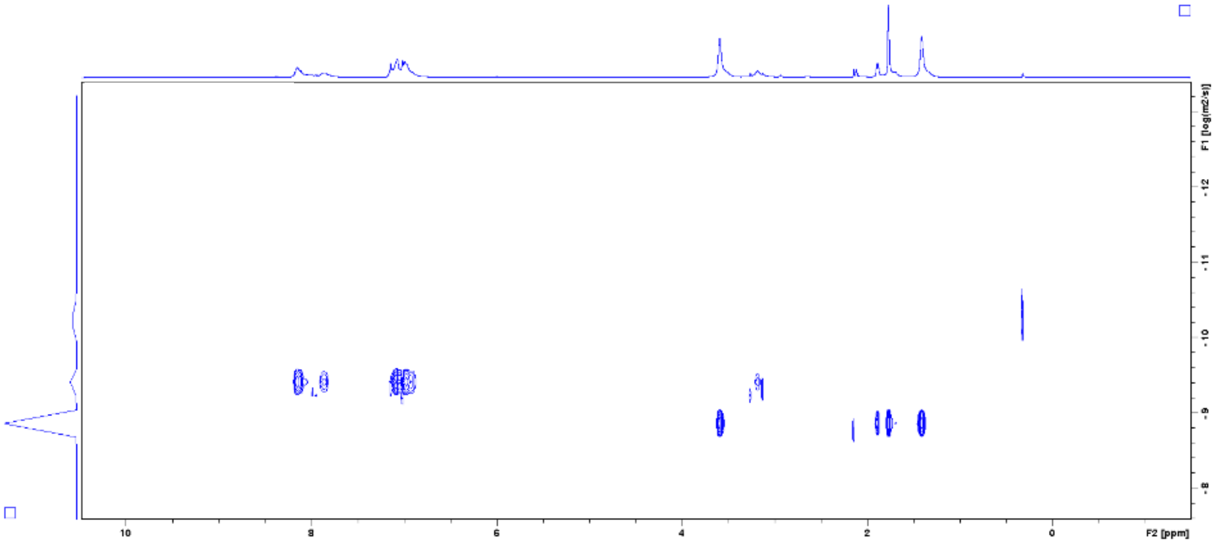

Figure S24. ^{1}H DOSY NMR spectrum for *rac*-**1-Li** in d$_8$-Tol at –10 °C.

Table S5. ^{1}H DOSY NMR data for *rac*-**1-Li** at –10 °C in d$_8$-Tol.

Solvent	Int. reference	ØD_{ref} [m^{2}s^{-1}]	ØlogD_{ref}	Ø logD_{Carb}	MW_{DOSY} [g/mol]	MW_{calc} [g/mol]	Species	Error [%]
Tol	Adam	8.62E-10	-9.06	-9.58	1051	995.92	Dimer + 2 THF	+5.5
						1068.03	Dimer + 3 THF	-1.6

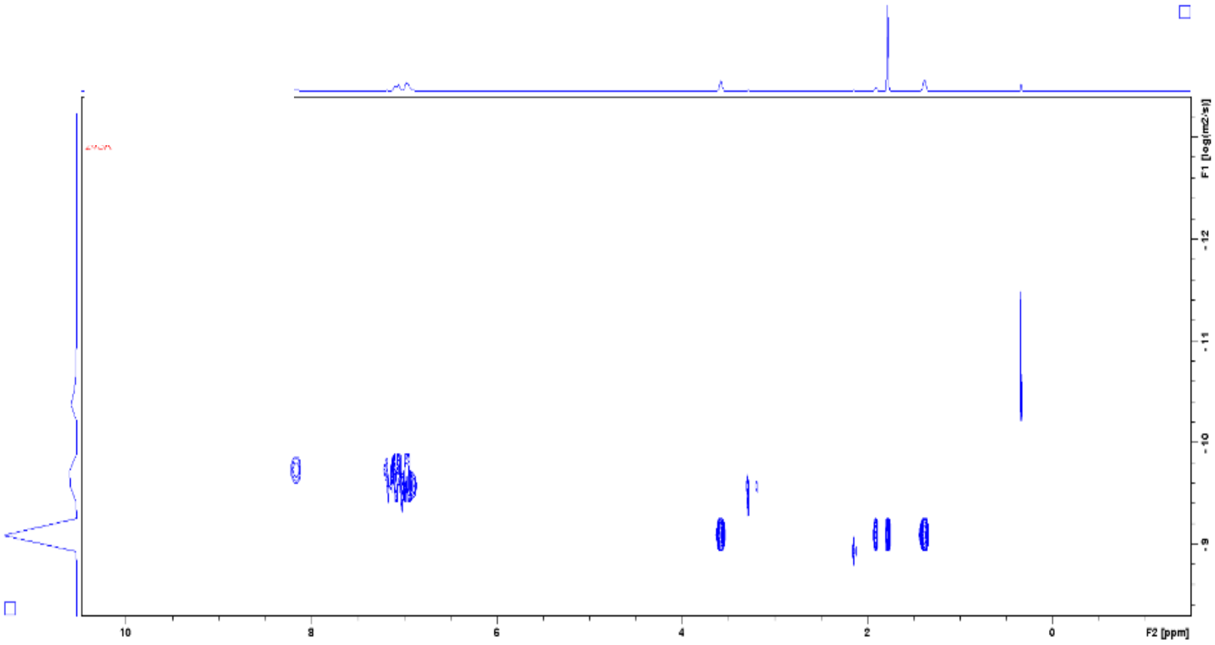

Figure S25. ^{1}H DOSY NMR spectrum for *rac*-**1-Li** in d_8-Tol at –30 °C.

Table S6. ^{1}H DOSY NMR data for *rac*-**1-Li** at –30 °C in d_8-Tol.

Solvent	Int. reference	ØD_{ref} [m^2s^{-1}]	Ø$logD_{ref}$	Ø $logD_{Carb}$	MW_{DOSY} [g/mol]	MW_{calc} [g/mol]	Species	Error [%]
Tol	Adam	5.36E-10	-9.27	-9.78	1025	995.92	Dimer + 2 THF	2.9
						1068.03	Dimer + 2 THF	-4.0

3.3 DOSY NMR Data of *rac*-1-Na

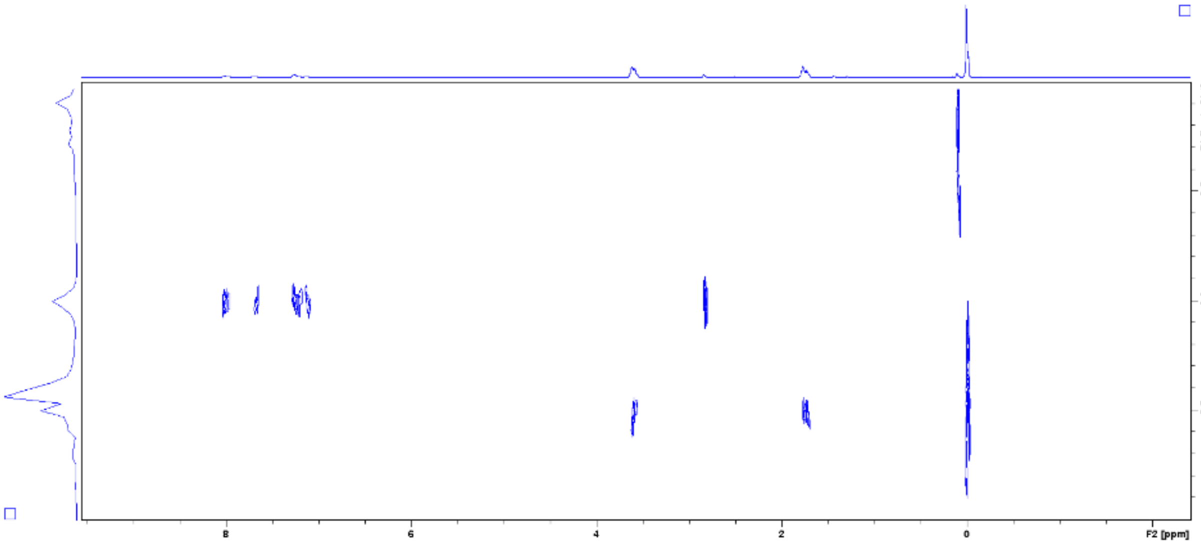

Figure S26. ^{1}H DOSY NMR spectrum for *rac*-**1-Na** in d_8-THF at 27 °C.

Table S7. ^{1}H DOSY NMR data for *rac*-**1-Na** at 27 °C in d_8-THF.

Solvent	Int. reference	D_{ref} [m^2s^{-1}]	$logD_{ref}$	Ø $logD_{Carb}$	MW_{DOSY} [g/mol]	MW_{calc} [g/mol]	Species	Error [%]
THF	TMS	2.33E-9	-8.63	-9.03	471	441.90	Monomer + 0 THF	6.6
						514.01	Monomer + 1 THF	-8.3

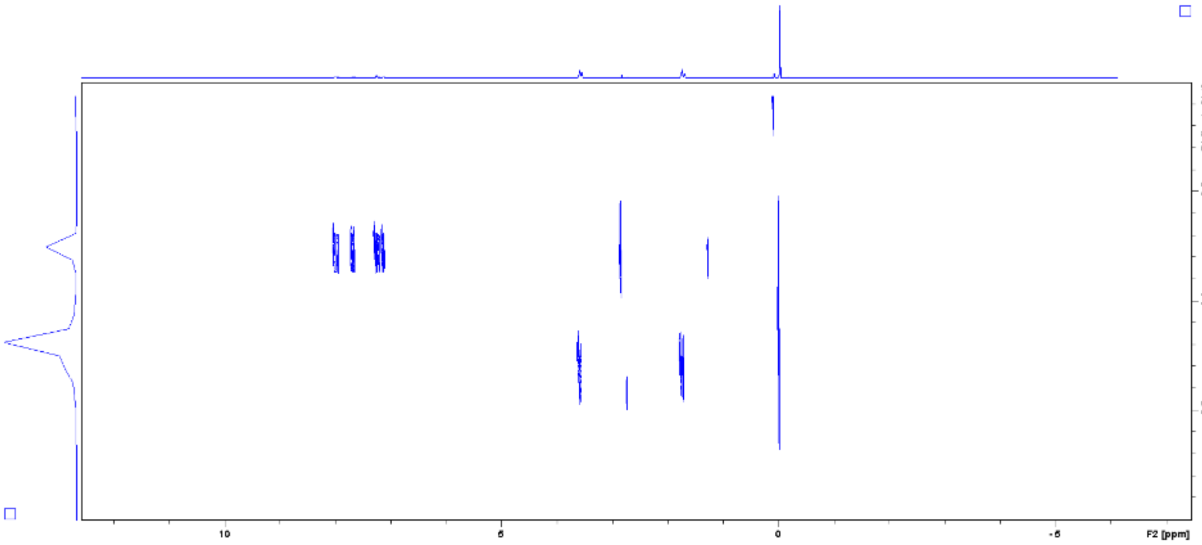

Figure S27. ^{1}H DOSY NMR spectrum for *rac*-**1-Na** in d_8-THF at –10 °C.

Table S8. ^{1}H DOSY NMR data for *rac*-**1-Na** at –10 °C in d_8-THF.

Solvent	Int. reference	D_{ref} [m^2s^{-1}]	$logD_{ref}$	Ø $logD_{Carb}$	MW_{DOSY} [g/mol]	MW_{calc} [g/mol]	Species	Error [%]
THF	TMS	1.44E-9	-8.84	-9.30	595	586.12	Monomer + 2THF	+1.5

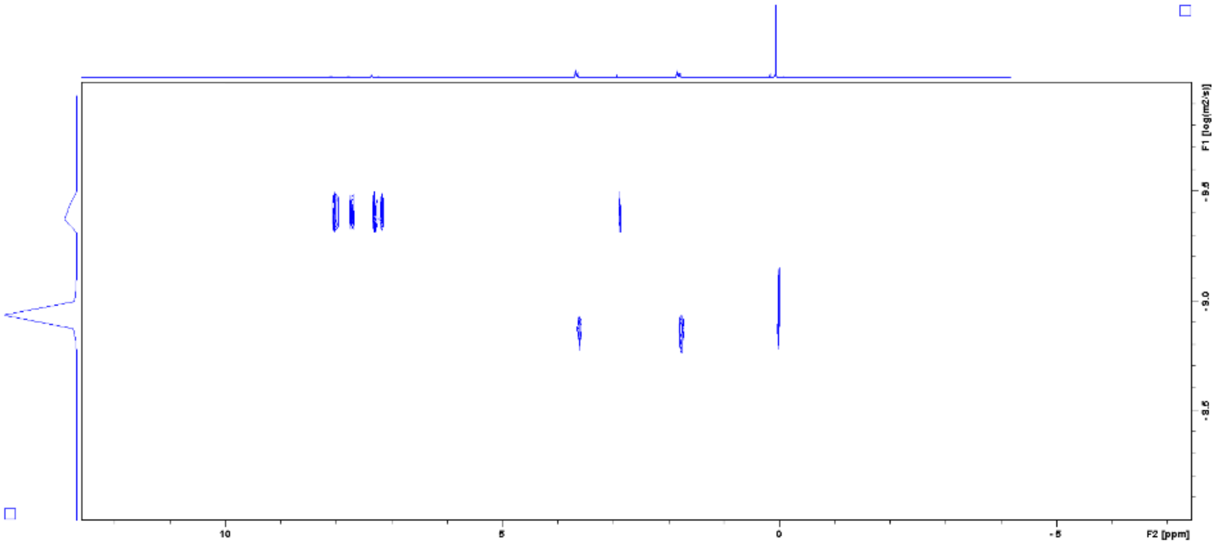

Figure S28. ^{1}H DOSY NMR spectrum for *rac*-**1-Na** in d_8-THF at –30 °C.

Table S9. ^{1}H DOSY NMR data for *rac*-**1-Na** at –30 °C in d_8-THF.

Solvent	Int. reference	D_{ref} [m^2s^{-1}]	$logD_{ref}$	Ø $logD_{Carb}$	MW_{DOSY} [g/mol]	MW_{calc} [g/mol]	Species	Error [%]
THF	TMS	1.01E-9	-8.99	-9.41	683	658.23	Monomer + 3THF	+3.8

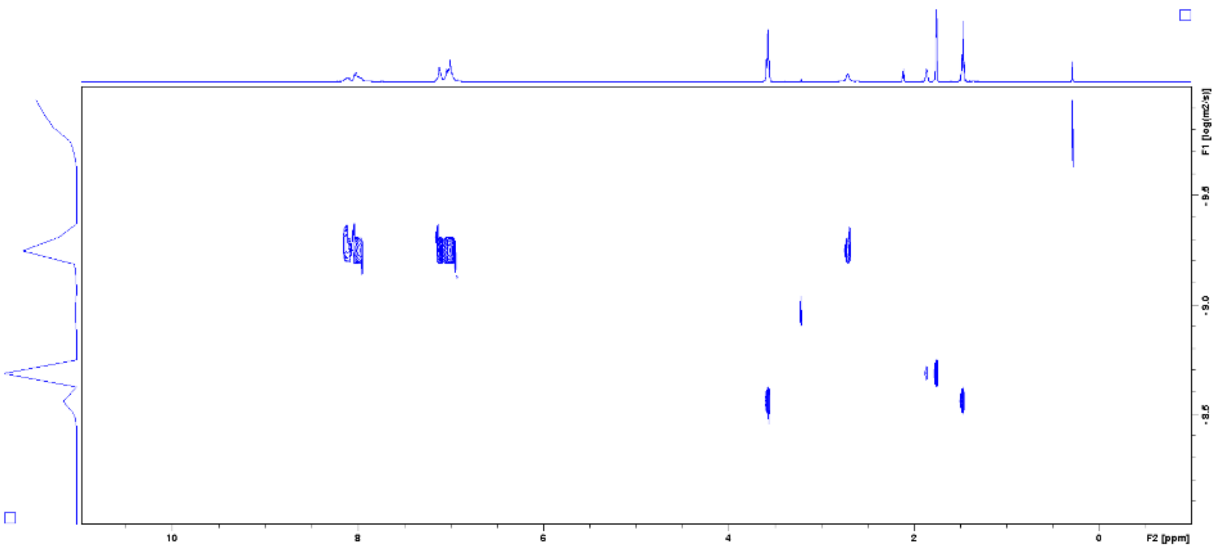

Figure S29. ^{1}H DOSY NMR spectrum for *rac*-**1-Na** in d_8-Tol at 27 °C.

Table S10. ^{1}H DOSY NMR data for *rac*-**1-Na** at 27 °C in d_8-Tol.

Solvent	Int. reference	ØD_{ref} [m^2s^{-1}]	ØlogD_{ref}	Ø logD_{Carb}	MW_{DOSY} [g/mol]	MW_{calc} [g/mol]	Species	Error [%]
Tol	Adam	1.80E-9	-8.75	-9.33	1330	1316.46	Dimer + 6THF	+1.0
						1325.70	Trimer	+0.3

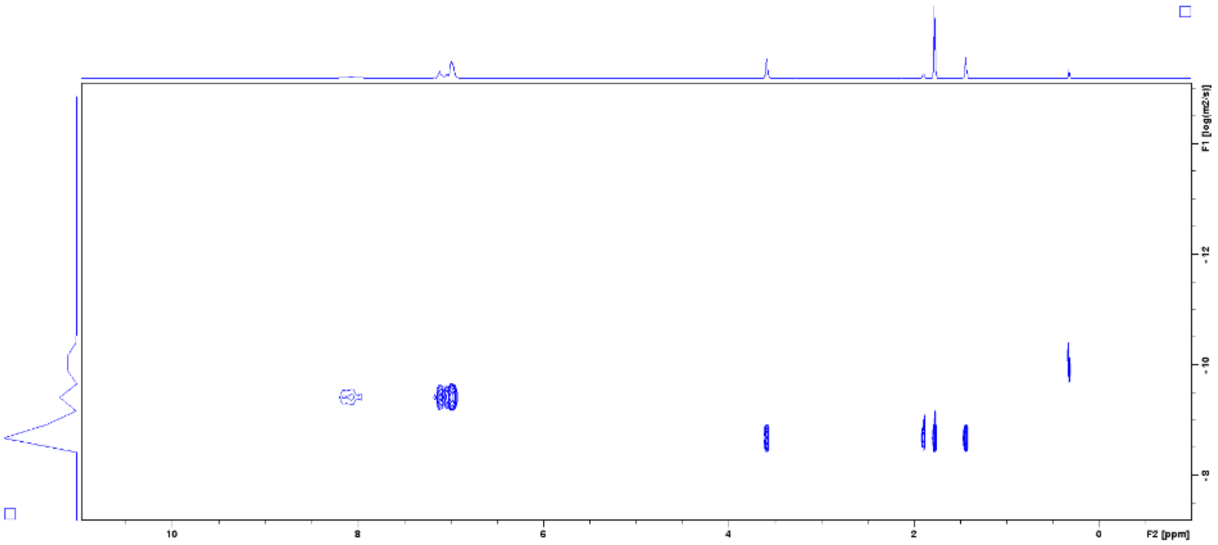

Figure S30. ^{1}H DOSY NMR spectrum for *rac*-**1-Na** in d_8-Tol at –10 °C.

Table S11. ^{1}H DOSY NMR data for *rac*-**1-Na** at –10 °C in d_8-Tol.

Solvent	Int. reference	ØD_{ref} [m^2s^{-1}]	ØlogD_{ref}	Ø logD_{Carb}	MW_{DOSY} [g/mol]	MW_{calc} [g/mol]	Species	Error [%]
Tol	Adam	9.14E-10	-9.04	-9.62	1312	1316.46	Dimer + 6THF	-0.3
						1325.70	Trimer	-1.0

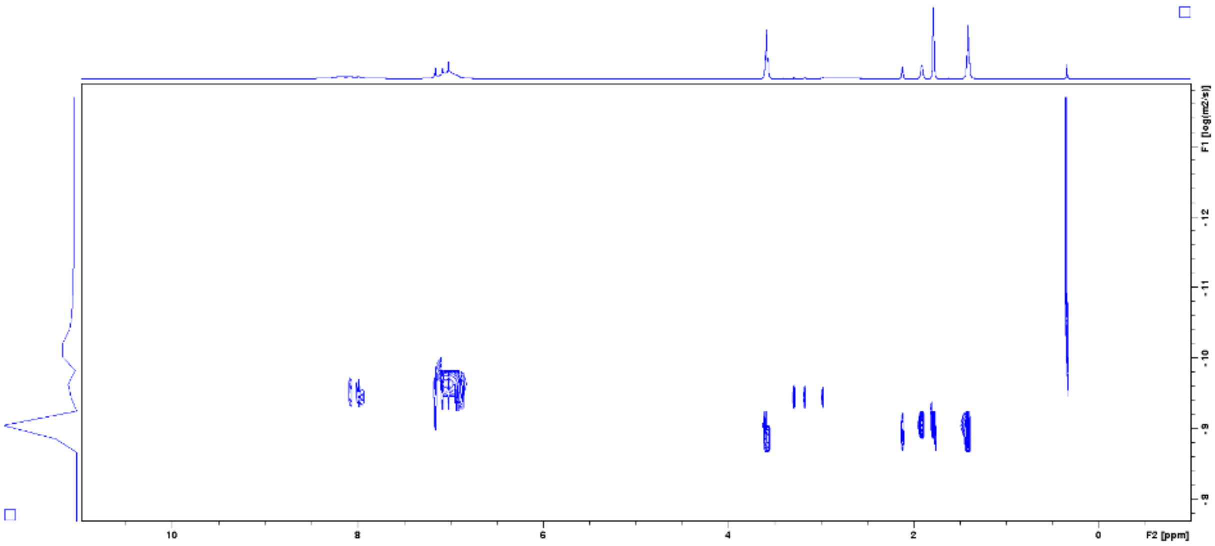

Figure S31. ^{1}H DOSY NMR spectrum for *rac*-**1-Na** in d_8-Tol at –30 °C.

Table S12. ^{1}H DOSY NMR data for *rac*-**1-Na** at –30 °C in d_8-Tol.

Solvent	Int. reference	$ØD_{ref}$ $[m^2s^{-1}]$	$ØlogD_{ref}$	Ø $logD_{Carb}$	MW_{DOSY} [g/mol]	MW_{calc} [g/mol]	Species	Error [%]
Tol	Adam	9.53E-10	-9.26	-9.81	1195	1172.24	Dimer + 4THF	+1.9

3.4 DOSY NMR data of *rac*-1-K

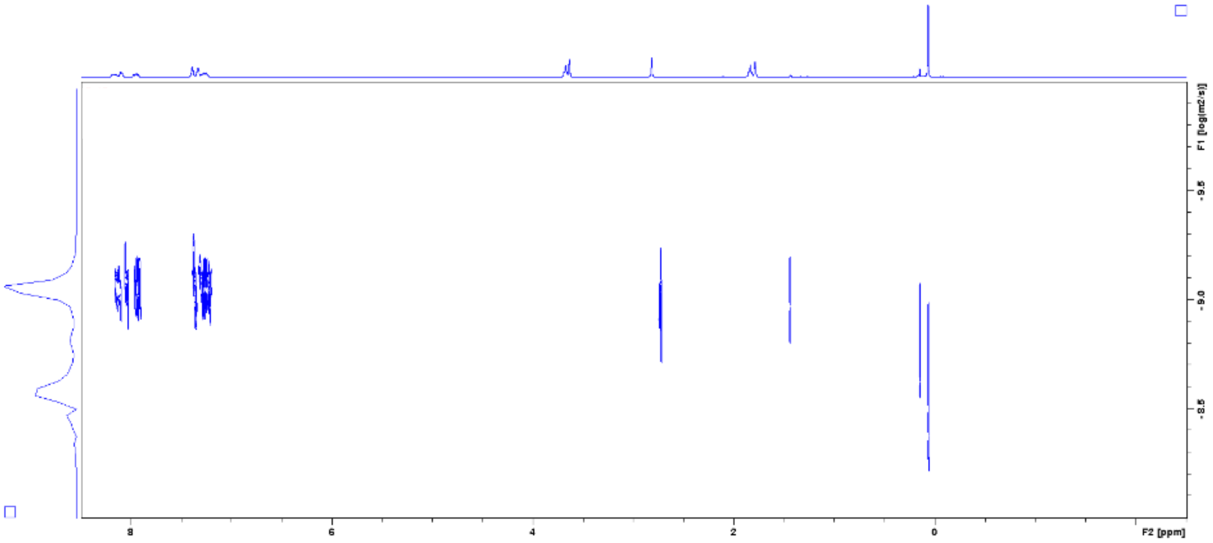

Figure S32. ^{1}H DOSY NMR spectrum for *rac*-**1-K** in d_8-THF at 27 °C.

Table S13. ^{1}H DOSY NMR data for *rac*-**1-K** at 27 °C in d_8-THF.

Solvent	Int. reference	D_{ref} [m^2s^{-1}]	$logD_{ref}$	Ø $logD_{Carb}$	MW_{DOSY} [g/mol]	MW_{calc} [g/mol]	Species	Error [%]
THF	TMS	2.17E-9	-8.66	-9.083	521	530.12	Monomer + 1THF	-1.7

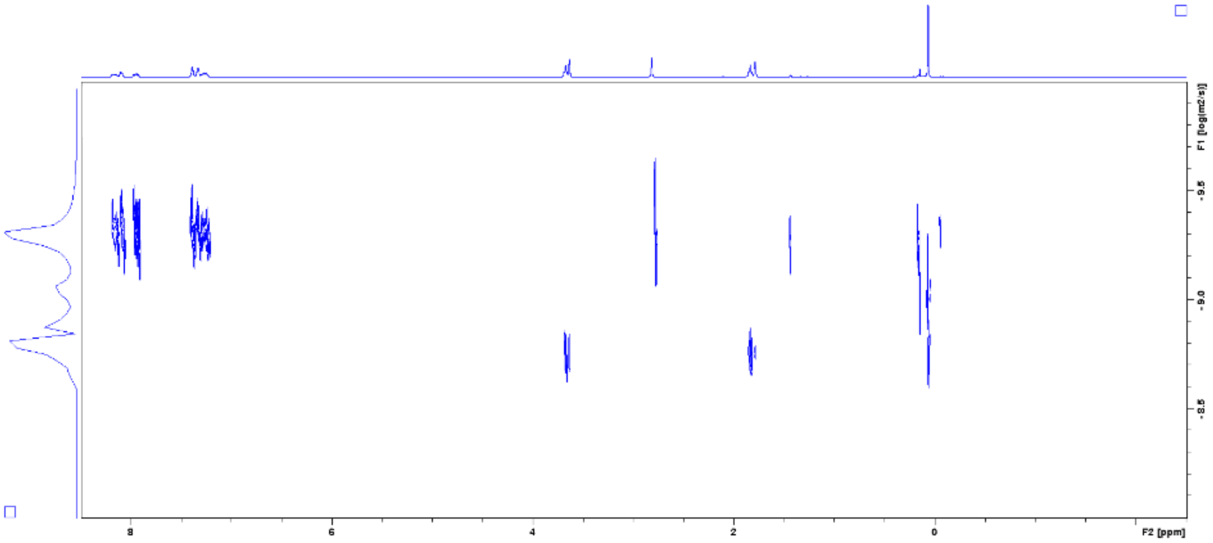

Figure S33. ^{1}H DOSY NMR spectrum for *rac*-**1-K** in d_8-THF at –10 °C.

Table S14. ^{1}H DOSY NMR data for *rac*-**1-K** at –10 °C in d_8-THF.

Solvent	Int. reference	D_{ref} [m^2s^{-1}]	$logD_{ref}$	Ø $logD_{Carb}$	MW_{DOSY} [g/mol]	MW_{calc} [g/mol]	Species	Error [%]
THF	TMS	1.24E-9	-8.91	-9.34	555	530.12	Monomer + 1THF	+4.7
						602.23	Monomer + 2THF	-7.8

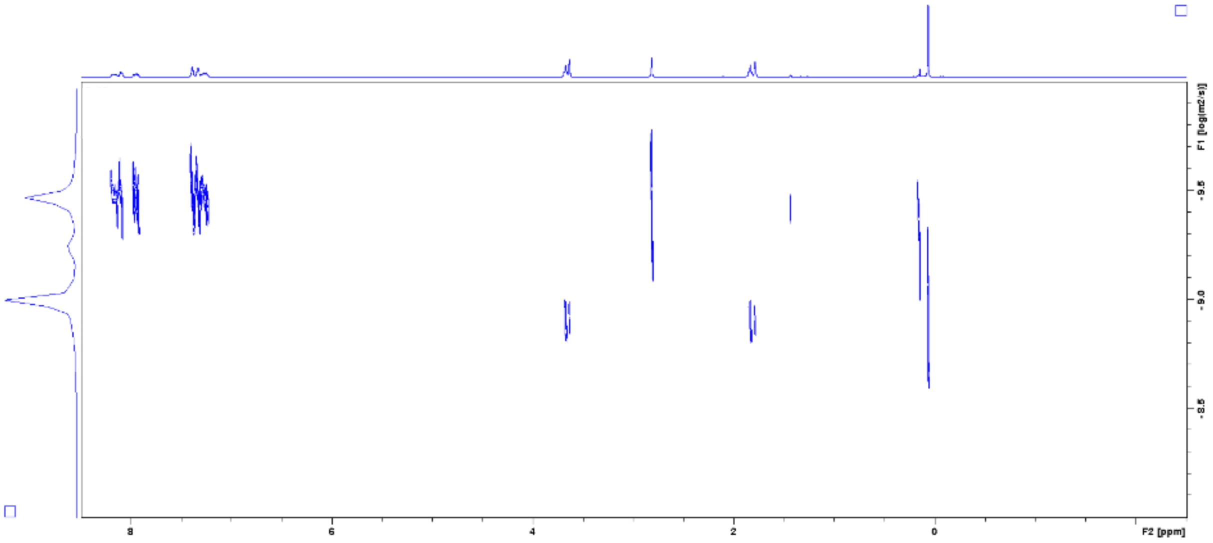

Figure S34. ^{1}H DOSY NMR spectrum for *rac*-**1-K** in d_8-THF at –30 °C.

Table S15. ^{1}H DOSY NMR data for *rac*-**1-K** at –30 °C in d_8-THF.

Solvent	Int. reference	D_{ref} [m^2s^{-1}]	$logD_{ref}$	Ø $logD_{Carb}$	MW_{DOSY} [g/mol]	MW_{calc} [g/mol]	Species	Error [%]
THF	TMS	9.16E-10	-9.04	-9.51	633	602.23	Monomer + 2THF	+5.1
						674.33	Monomer + 3THF	-6.1

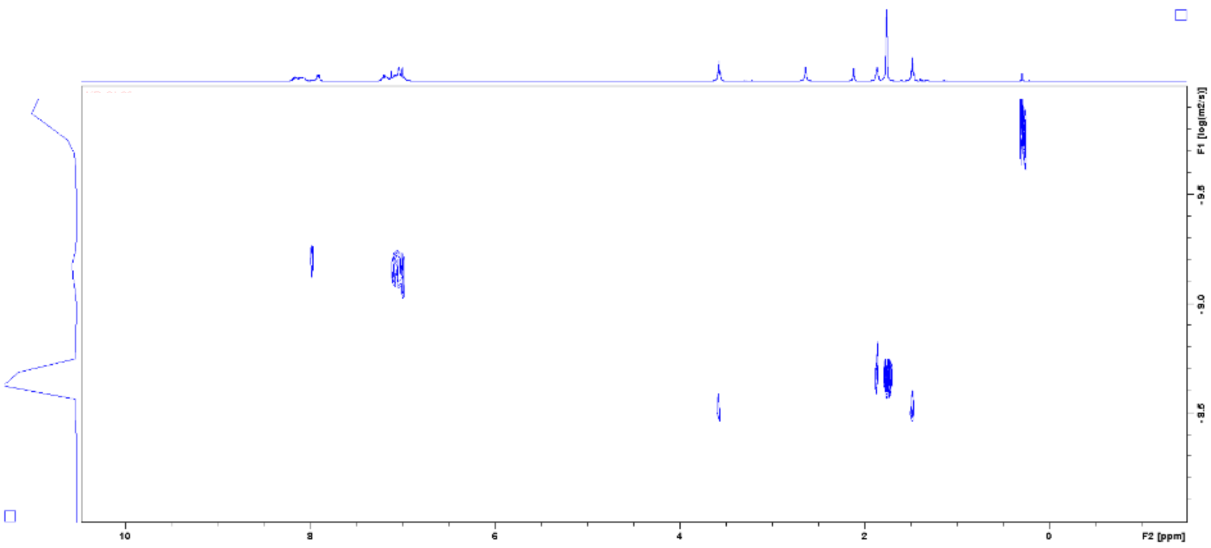

Figure S35. ^{1}H DOSY NMR spectrum for *rac*-**1-K** in d_8-Tol at 27 °C.

Table S16. ^{1}H DOSY NMR data for *rac*-**1-K** at 27 °C in d_8-Tol.

Solvent	Int. reference	ØD_{ref} [m^2s^{-1}]	Ø$logD_{ref}$	Ø $logD_{Carb}$	MW_{DOSY} [g/mol]	MW_{calc} [g/mol]	Species	Error [%]
Tol	Adam	1.71E-9	-8.77	-9.31	1135	1132.35	Dimer + 3THF	+0.2

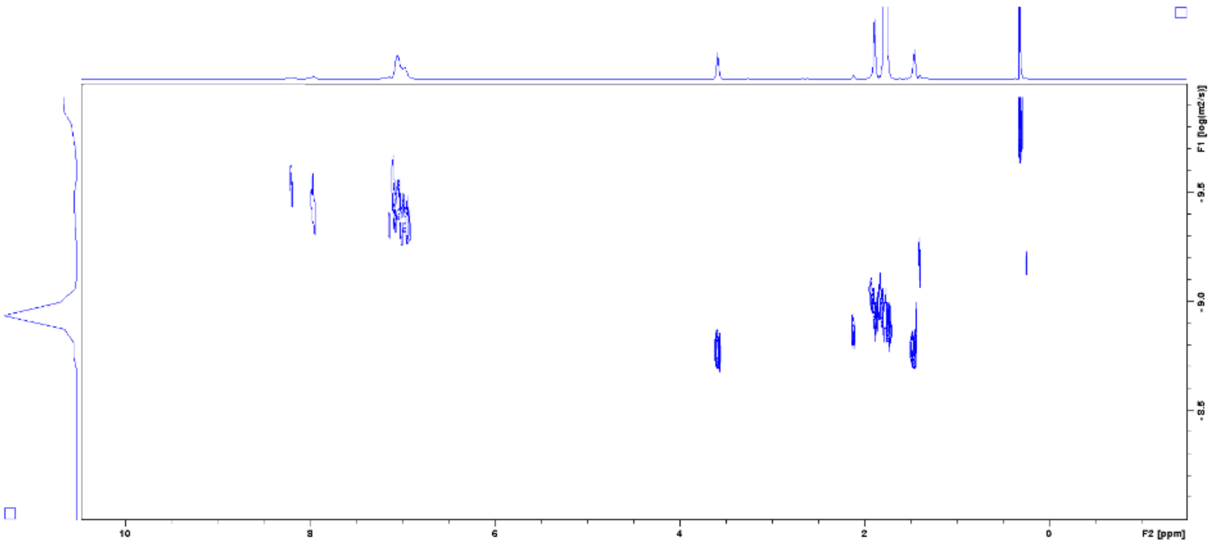

Figure S36. ^{1}H DOSY NMR spectrum for *rac*-**1-K** in d_8-Tol at –10 °C.

Table S17. ^{1}H DOSY NMR data for *rac*-**1-K** at –10 °C in d_8-Tol.

Solvent	Int. reference	ØD_{ref} [m^2s^{-1}]	Ø$logD_{ref}$	Ø $logD_{Carb}$	MW_{DOSY} [g/mol]	MW_{calc} [g/mol]	Species	Error [%]
Tol	Adam	9.51E-9	-9.02	-9.53	1012	1060.24	Dimer + 2THF	-4.5

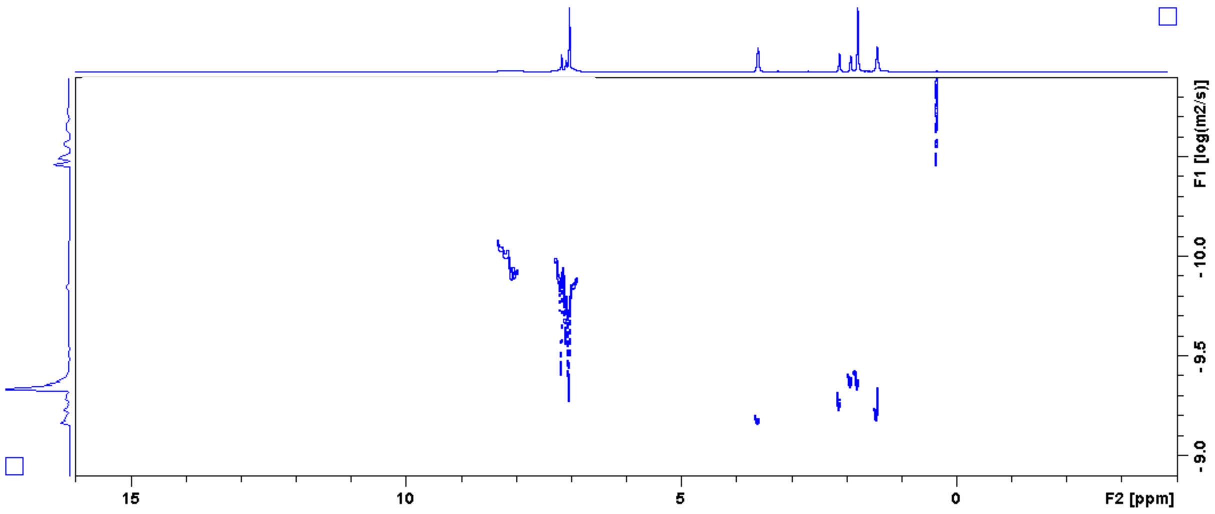

Figure S37. ¹H DOSY NMR spectrum for *rac*-**1-K** in d_8-Tol at –30 °C.

Table S18. ¹H DOSY NMR data for *rac*-**1-K** at –30 °C in d_8-Tol.

Solvent	Int. reference	$ØD_{ref}$ [m^2s^{-1}]	$ØlogD_{ref}$	Ø $logD_{Carb}$	MW_{DOSY} [g/mol]	MW_{calc} [g/mol]	Species	Error [%]
Tol	Adam	4.70E-10	-9.33	-9.89	1252	1251.46	Dimer + 6THF	0.0

4. Determination of *ee*

	Reten. Time [min]	Area [mAU.s]	Height [mAU]	Area [%]	W05 [min]	PDA Peak Purity	Compound Name	PDA Best Match Name
1	14,233	43683,380	1317,071	100,0	0,53	891		
	Total	43683,380	1317,071	100,0				

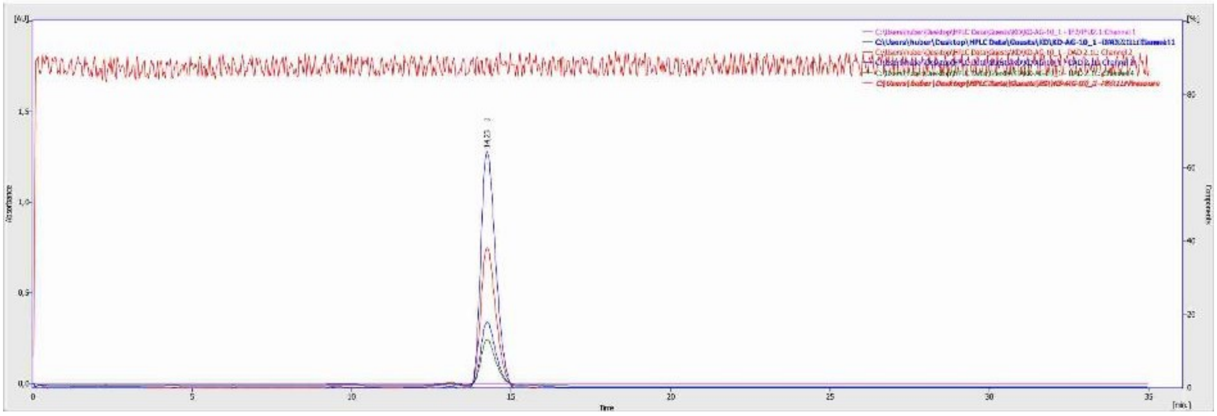

Figure S38. HPLC chromatogram of *R*-**2** in hexane:isopropanol (85:15).

	Reten. Time [min]	Area [mAU.s]	Height [mAU]	Area [%]	W05 [min]	PDA Peak Purity	Compound Name	PDA Best Match Name
1	14,217	25766,366	830,776	50,0	0,52	925		
2	15,467	25803,410	742,596	50,0	0,55	907		
	Total	51569,775	1573,372	100,0				

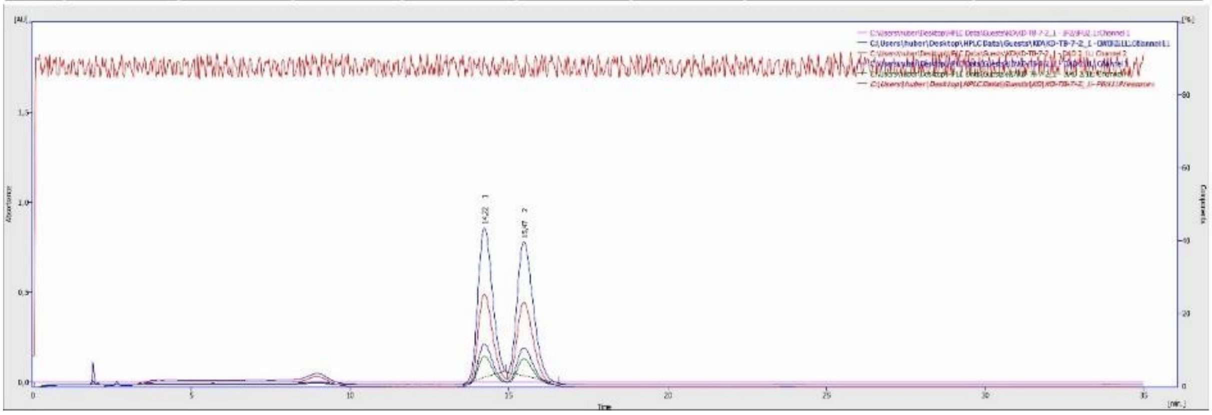

Figure S39. HPLC chromatogram of ***rac*-2** in hexane:isopropanol (85:15).

5. Crystal Structure Determination

5.1 General information

Data collection of all compounds was conducted with an Oxford Synergy. The structures were solved using direct methods, refined with the Shelx software package[2] and expanded using Fourier techniques. The crystals of all compounds were mounted in an inert oil (perfluoropolyalkylether). Crystal structure determinations were affected at 100 K. Crystallographic data (including structure factors) have been deposited with the Cambridge Crystallographic Data Centre as supplementary publication no. CCDC-2022984-2022987. Copies of the data can be obtained free of charge on application to Cambridge Crystallographic Data Centre, 12 Union Road, Cambridge CB2 1EZ, UK; [fax: (+44) 1223-336-033; email: deposit@ccdc.cam.ac.uk].

The structure of ***rac*-1-Na(18crown6)** has been solved using the DELU and SIMU commands to prevent distortion of an ellipsoid due to residue electron density from a minor disorder.

The structure of ***rac*-1-K(18crown6)** contained a twin with the occupancy 0.59:0.41 and was solved with the TWIN and BASF commands (Twin law: -1 0 0 0 1 0 0 0 -1)

Table S19. Data collection and structure refinement details for compound *S,S*-**1-H** and *rac*-**1-Li.**

**Parameter**	***S,S*-1-H**	***rac*-1-Li**
CCDC No.	CCDC-2022984	CCDC-2022985
Emperical formula	$C_{20}H_{19}ClNOPS_2$	$C_{32}H_{42}ClLiNO_4PS_2$
Formula weight	419.90 g/mol	642.14 g/mol
Temperature	100(2) K	100(2) K
Wavelength	0.71073 Å	1.54184 Å
Crystal system	Monoclinic	Monoclinic
Space group	$P2_1$	$P2_1/c$
Unit cell dimensions	a = 9.3549(5) Å	a = 8.9364(4) Å
	b = 9.4299(5) Å	b = 19.994(3) Å
	c = 10.8508(6) Å	c = 18.1879(14) Å
	α= 90°	α = 90°
	β= 92.165(2)°	β= 97.652(6)°
	γ = 90°	γ = 90°
Volume	956.53(9) Å^3	3220.8(5) Å^3
Formula unit per cell	2	4
Density (calculated)	1.458 Mg/m^3	1.324 Mg/m^3
Absorption coefficient	0.511 mm^{-1}	3.025 mm^{-1}
F(000)	436	1360
Crystal size	0.30 x 0.25 x 0.24 mm^3	0.409 x 0.164 x 0.116 mm^3
Θ range for data collection	1.878 to 26.395°°	3.301 to 72.483°

Index ranges	-11 ≤ h ≤ 11, -11 ≤ k ≤ 11, -13 ≤ l ≤ 13	-11 ≤ h ≤ 11, -21 ≤ k ≤ 24, -22 ≤ l ≤ 21
Reflections collected	17758	25932
Independent reflections	3907 [*R*(int) = 0.0188]	6357 [*R*(int) = 0.0302]
Refinement method	Full-matrix least-squares on F^2	Full-matrix least-squares on F^2
Data / restraints / parameters	3907 / 1 / 236	6357 / 0 / 505
Goodness-of-fit on F^2	1.054	1.028
Final *R* indices [I>2σ(I)]	*R*1 = 0.0178, w*R*2 = 0.0484	*R*1 = 0.0343, w*R*2 = 0.0895
R indices (all data)	*R*1 = 0.0180, w*R*2 = 0.0486	*R*1 = 0.0379, w*R*2 = 0.0928
Largest diff. peak and hole	0.294 and -0.157 e.Å^{-3}	0.513 and -0.367 e.Å^{-3}

Table S20. Data collection and structure refinement details for compound *rac*-**1**-Na(18crown6) and *rac*-1-K(18crown6).

**Parameter**	***rac*-1-Na(18crown6)**	***rac*-1-K(18crown6)**
CCDC No.	CCDC-2022986	CCDC-2022987
Emperical formula	$C_{80}H_{116}Cl_2N_2Na_2O_{18}P_2S_4$	$C_{40}H_{58}ClKNO_{11}PS_2$
Formula weight	1700.80 g/mol	898.51 g/mol
Temperature	100(2) K	100(2) K
Wavelength	1.54184 Å	1.54184 Å
Crystal system	Triclinic	Triclinic
Space group	$P\bar{1}$	$P\bar{1}$
Unit cell dimensions	a = 9.2135(3) Å	a = 9.1916(3) Å
	b = 20.4163(6) Å	b = 20.6188(8) Å
	c = 23.8781(8) Å	c = 23.3493(5) Å
	α = 75.754(3)°	α = 89.975(2)°
	β = 80.345(3)°	β = 84.739(2)°
	γ = 87.399(3)°	γ = 89.983(3)°
Volume	4291.7(2) Å^3	4406.5(2) Å^3
Formula unit per cell	2	4
Density (calculated)	1.316 Mg/m^3	1.354 Mg/m^3
Absorption coefficient	2.586 mm^{-1}	3.323 mm^{-1}
F(000)	1808	1904
Crystal size	0.129 x 0.059 x 0.059 mm^3	0.295 x 0.036 x 0.017 mm^3
Θ range for data collection	3.290 to 67.080°	2.865 to 67.499°
Index ranges	-11 ≤ h ≤ 9, -24 ≤ k ≤ 20, -28 ≤ l ≤ 28	-10 ≤ h ≤ 11, -24 ≤ k ≤ 24, -26 ≤ l ≤ 27
Reflections collected	29548	79386
Independent reflections	15283 [*R*(int) = 0.0564]	15421 [*R*(int) = 0.1498]

Refinement method	Full-matrix least-squares on F^2	Full-matrix least-squares on F^2
Data / restraints / parameters	15283 / 7 / 997	15421 / 0 / 1049
Goodness-of-fit on F^2	1.024	1.040
Final *R* indices [I>2σ(I)]	*R*1 = 0.0519, w*R*2 = 0.1281	*R*1 = 0.0799, w*R*2 = 0.2051
R indices (all data)	*R*1 = 0.0798, w*R*2 = 0.1432	*R*1 = 0.0855, w*R*2 = 0.2103
Largest diff. peak and hole	1.573 and -0.597 e.Å^{-3}	1.422 and -1.312 e.Å^{-3}

5.2 Further Details to the Crystal Structures

Crystal Structure Determination of (*S,S*)-1-H

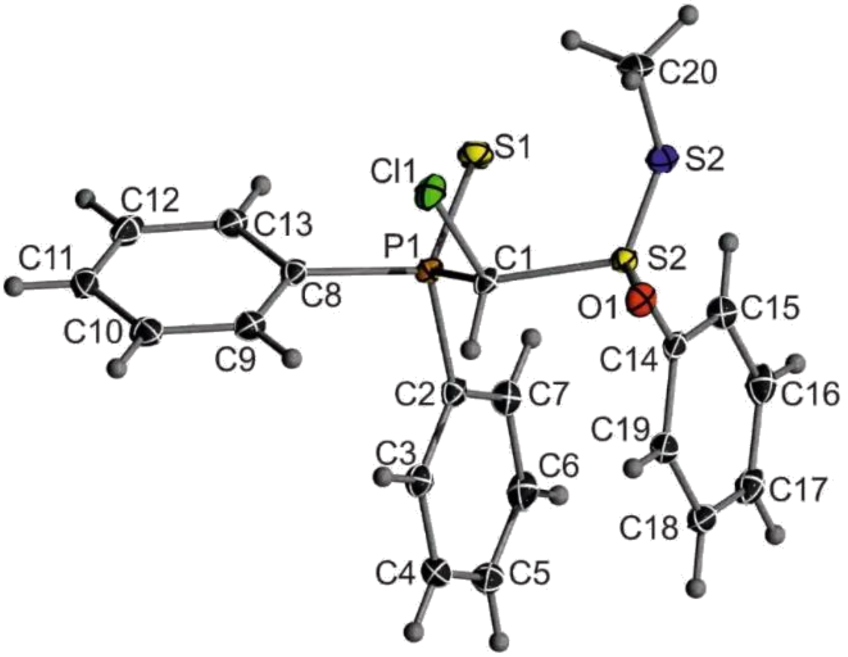

Figure S40. ORTEP of compound *S,S*-1-H. Ellipsoids are drawn at the 50% probability level.

Table S21. Atomic coordinates (x 10^4) and equivalent isotropic displacement parameters (Å^{2}x 10^3) for *S,S*-**1**-**H**. U(eq) is defined as one third of the trace of the orthogonalized U^{ij} tensor.

	x	y	z	U(eq)
Cl(1)	8242(1)	11120(1)	5139(1)	18(1)
S(1)	8418(1)	9172(1)	1977(1)	18(1)
S(2)	6077(1)	8978(1)	4718(1)	13(1)
P(1)	7297(1)	10780(1)	2499(1)	11(1)
O(1)	5467(2)	9451(2)	5862(1)	17(1)
N(1)	7085(2)	7745(2)	4670(2)	17(1)
C(1)	6767(2)	10695(2)	4130(2)	12(1)
C(2)	5613(2)	11039(2)	1649(2)	13(1)
C(3)	4751(2)	12213(2)	1895(2)	16(1)

C(4)	3489(2)	12413(2)	1224(2)	19(1)
C(5)	3073(2)	11475(2)	295(2)	21(1)
C(6)	3918(2)	10311(2)	45(2)	20(1)
C(7)	5188(2)	10086(2)	724(2)	16(1)
C(8)	8194(2)	12484(2)	2376(2)	14(1)
C(9)	7819(2)	13671(2)	3063(2)	16(1)
C(10)	8456(2)	14971(2)	2859(2)	19(1)
C(11)	9481(2)	15091(2)	1965(2)	21(1)
C(12)	9853(2)	13930(3)	1279(2)	21(1)
C(13)	9229(2)	12614(2)	1489(2)	17(1)
C(14)	4673(2)	8571(2)	3647(2)	15(1)
C(15)	4767(2)	7443(2)	2838(2)	18(1)
C(16)	3614(2)	7184(2)	2027(2)	21(1)
C(17)	2424(2)	8058(2)	2019(2)	21(1)
C(18)	2342(2)	9184(2)	2839(2)	20(1)
C(19)	3472(2)	9445(2)	3670(2)	17(1)
C(20)	8416(2)	7676(3)	5437(2)	23(1)

Table S22. Anisotropic displacement parameters ($Å^2$x 10^3) for *S,S*-1-H. The anisotropic displacement factor exponent takes the form: $-2p^2[h^2 a^{*2}U^{11} + ... + 2 h k a^* b^* U^{12}]$

	U^{11}	U^{22}	U^{33}	U^{23}	U^{13}	U^{12}
Cl(1)	18(1)	22(1)	15(1)	0(1)	-4(1)	-5(1)
S(1)	16(1)	17(1)	20(1)	-4(1)	3(1)	3(1)
S(2)	11(1)	14(1)	14(1)	2(1)	-1(1)	-1(1)
P(1)	11(1)	13(1)	11(1)	-1(1)	1(1)	0(1)
O(1)	18(1)	20(1)	13(1)	1(1)	2(1)	-1(1)
N(1)	15(1)	17(1)	20(1)	0(1)	-3(1)	1(1)
C(1)	11(1)	14(1)	11(1)	-1(1)	-1(1)	-2(1)
C(2)	13(1)	16(1)	11(1)	2(1)	0(1)	-2(1)
C(3)	17(1)	16(1)	15(1)	-1(1)	0(1)	-2(1)
C(4)	17(1)	16(1)	23(1)	4(1)	1(1)	1(1)
C(5)	18(1)	23(1)	20(1)	7(1)	-6(1)	-3(1)
C(6)	24(1)	21(1)	16(1)	0(1)	-3(1)	-6(1)
C(7)	18(1)	17(1)	14(1)	0(1)	1(1)	-2(1)
C(8)	11(1)	16(1)	15(1)	2(1)	-2(1)	-2(1)
C(9)	14(1)	19(1)	16(1)	0(1)	0(1)	0(1)
C(10)	18(1)	17(1)	23(1)	-1(1)	-5(1)	0(1)
C(11)	16(1)	20(1)	28(1)	9(1)	-7(1)	-5(1)
C(12)	14(1)	30(1)	20(1)	9(1)	1(1)	-2(1)
C(13)	14(1)	21(1)	16(1)	2(1)	1(1)	1(1)
C(14)	12(1)	16(1)	16(1)	4(1)	-1(1)	-4(1)
C(15)	17(1)	16(1)	20(1)	2(1)	1(1)	-1(1)
C(16)	25(1)	20(1)	20(1)	-1(1)	-2(1)	-6(1)
C(17)	19(1)	25(1)	19(1)	5(1)	-5(1)	-8(1)
C(18)	13(1)	22(1)	24(1)	6(1)	-1(1)	-1(1)
C(19)	16(1)	16(1)	18(1)	2(1)	1(1)	-2(1)
C(20)	14(1)	23(1)	31(1)	3(1)	-7(1)	4(1)

Crystal Structure Determination of *rac*-1-Li

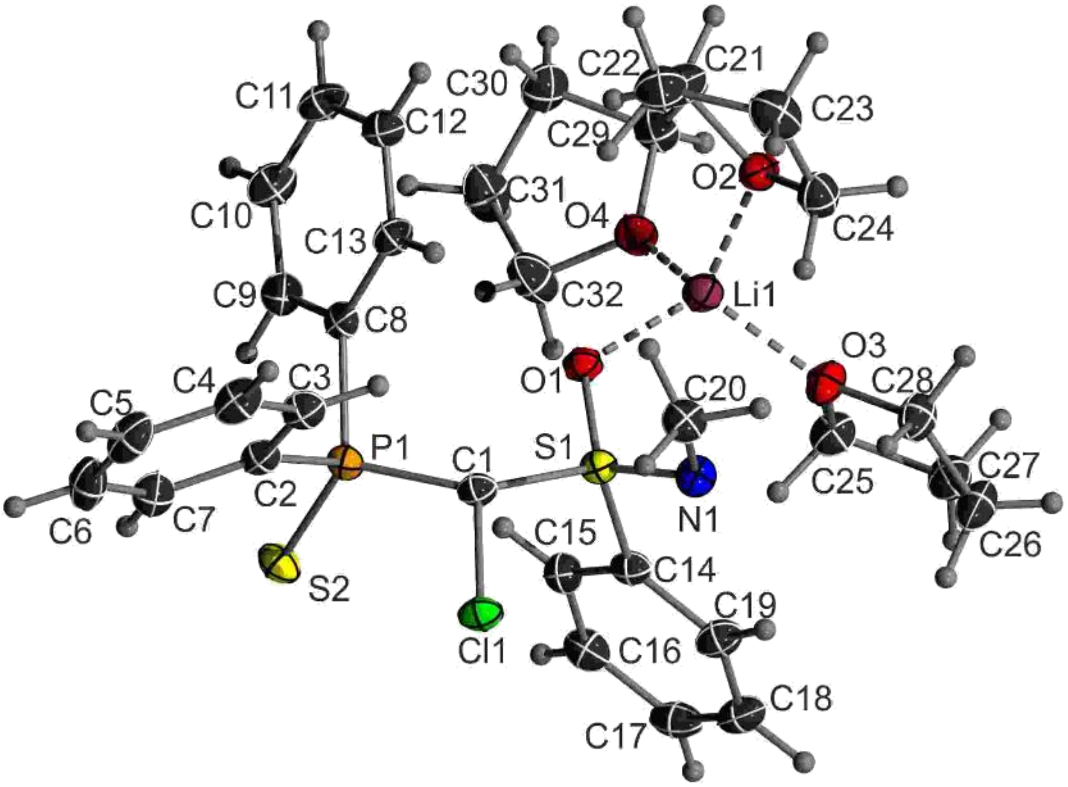

Figure S41. ORTEP of compound *rac*-**1-Li**. Ellipsoids are drawn at the 50% probability level.

Table S23. Atomic coordinates (x 10^4) and equivalent isotropic displacement parameters (Å^{2}x 10^3) for *rac*-**1-Li**. U(eq) is defined as one third of the trace of the orthogonalized U^{ij} tensor.

	x	y	z	U(eq)
Li(1)	3025(3)	5815(1)	2182(1)	24(1)
Cl(1)	7063(1)	6836(1)	4726(1)	23(1)
S(1)	5020(1)	6278(1)	3538(1)	18(1)
S(2)	4178(1)	8238(1)	4248(1)	25(1)
P(1)	5592(1)	7799(1)	3658(1)	18(1)
N(1)	5930(1)	5632(1)	3632(1)	22(1)
O(1)	4221(1)	6458(1)	2803(1)	21(1)
O(2)	4174(1)	5459(1)	1439(1)	26(1)
O(3)	2257(1)	5067(1)	2676(1)	25(1)
O(4)	1346(1)	6334(1)	1695(1)	28(1)
C(1)	6080(2)	6955(1)	3822(1)	19(1)
C(2)	7437(2)	8218(1)	3797(1)	21(1)
C(3)	8700(2)	7909(1)	3563(1)	25(1)

C(4)	10076(2)	8240(1)	3634(1)	30(1)
C(5)	10206(2)	8880(1)	3932(1)	33(1)
C(6)	8963(2)	9187(1)	4161(1)	32(1)
C(7)	7577(2)	8854(1)	4099(1)	27(1)
C(8)	5005(2)	7904(1)	2667(1)	19(1)
C(9)	3838(2)	8345(1)	2424(1)	24(1)
C(10)	3452(2)	8468(1)	1671(1)	31(1)
C(11)	4214(2)	8148(1)	1157(1)	29(1)
C(12)	5372(2)	7704(1)	1397(1)	26(1)
C(13)	5773(2)	7585(1)	2146(1)	22(1)
C(14)	3547(2)	6118(1)	4088(1)	20(1)
C(15)	2388(2)	6588(1)	4062(1)	23(1)
C(16)	1206(2)	6475(1)	4473(1)	26(1)
C(17)	1186(2)	5904(1)	4902(1)	29(1)
C(18)	2356(2)	5444(1)	4931(1)	31(1)
C(19)	3547(2)	5552(1)	4523(1)	26(1)
C(20)	7429(2)	5645(1)	3382(1)	26(1)
C(21)	4621(2)	5950(1)	928(1)	32(1)
C(22)	6331(2)	5978(1)	1085(1)	34(1)
C(23)	6709(2)	5247(1)	1247(1)	38(1)
C(24)	5443(2)	5019(1)	1666(1)	31(1)
C(25)	770(2)	5080(1)	2898(1)	27(1)
C(26)	449(2)	4369(1)	3118(1)	31(1)
C(27)	2012(2)	4109(1)	3416(1)	35(1)
C(28)	3023(2)	4455(1)	2924(1)	29(1)
C(29)	656(2)	6284(1)	936(1)	28(1)
C(30)	416(2)	6997(1)	677(1)	35(1)
C(31)	2(2)	7338(1)	1368(1)	39(1)
C(32)	946(2)	6968(1)	1994(1)	39(1)

Table S24. Anisotropic displacement parameters (Å^{2}x 10^3) for *rac*-**1-Li**. The anisotropic displacement factor exponent takes the form: -2p^2[h^2 a*^{2}U^{11} + ... + 2 h k a* b* U^{12}]

	U^{11}	U^{22}	U^{33}	U^{23}	U^{13}	U^{12}
Li(1)	23(1)	24(1)	23(1)	1(1)	1(1)	-1(1)
Cl(1)	21(1)	29(1)	17(1)	2(1)	-2(1)	-1(1)
S(1)	16(1)	20(1)	17(1)	1(1)	1(1)	-1(1)
S(2)	22(1)	28(1)	25(1)	-6(1)	6(1)	1(1)
P(1)	15(1)	20(1)	17(1)	-1(1)	1(1)	-1(1)
N(1)	19(1)	23(1)	25(1)	1(1)	4(1)	1(1)

O(1)	21(1)	24(1)	18(1)	1(1)	0(1)	-3(1)
O(2)	28(1)	26(1)	23(1)	2(1)	5(1)	1(1)
O(3)	22(1)	24(1)	32(1)	4(1)	9(1)	0(1)
O(4)	29(1)	26(1)	25(1)	-1(1)	-4(1)	5(1)
C(1)	18(1)	23(1)	16(1)	1(1)	-1(1)	0(1)
C(2)	18(1)	24(1)	20(1)	4(1)	-1(1)	-3(1)
C(3)	22(1)	29(1)	23(1)	2(1)	0(1)	-2(1)
C(4)	20(1)	36(1)	32(1)	8(1)	2(1)	-2(1)
C(5)	24(1)	36(1)	38(1)	11(1)	-5(1)	-11(1)
C(6)	31(1)	24(1)	39(1)	3(1)	-8(1)	-8(1)
C(7)	24(1)	26(1)	29(1)	2(1)	-4(1)	0(1)
C(8)	16(1)	21(1)	20(1)	2(1)	-1(1)	-3(1)
C(9)	21(1)	25(1)	27(1)	1(1)	0(1)	1(1)
C(10)	25(1)	35(1)	30(1)	7(1)	-3(1)	5(1)
C(11)	28(1)	37(1)	21(1)	8(1)	-2(1)	-2(1)
C(12)	27(1)	30(1)	22(1)	2(1)	5(1)	-2(1)
C(13)	20(1)	24(1)	23(1)	3(1)	2(1)	0(1)
C(14)	17(1)	26(1)	17(1)	0(1)	0(1)	-2(1)
C(15)	21(1)	25(1)	24(1)	0(1)	2(1)	-2(1)
C(16)	20(1)	34(1)	26(1)	-6(1)	3(1)	-1(1)
C(17)	23(1)	45(1)	21(1)	-3(1)	5(1)	-8(1)
C(18)	30(1)	40(1)	22(1)	8(1)	2(1)	-6(1)
C(19)	22(1)	32(1)	23(1)	5(1)	0(1)	-1(1)
C(20)	20(1)	29(1)	29(1)	0(1)	4(1)	2(1)
C(21)	28(1)	40(1)	29(1)	11(1)	4(1)	1(1)
C(22)	29(1)	40(1)	32(1)	2(1)	8(1)	0(1)
C(23)	31(1)	43(1)	39(1)	-5(1)	4(1)	8(1)
C(24)	33(1)	26(1)	31(1)	-1(1)	-2(1)	2(1)
C(25)	20(1)	31(1)	30(1)	3(1)	5(1)	1(1)
C(26)	29(1)	31(1)	34(1)	-1(1)	10(1)	-6(1)
C(27)	37(1)	25(1)	45(1)	8(1)	13(1)	2(1)
C(28)	29(1)	22(1)	38(1)	2(1)	10(1)	4(1)
C(29)	24(1)	30(1)	27(1)	-2(1)	-5(1)	1(1)
C(30)	34(1)	33(1)	34(1)	6(1)	-6(1)	2(1)
C(31)	34(1)	34(1)	47(1)	-6(1)	-8(1)	10(1)
C(32)	42(1)	37(1)	36(1)	-9(1)	-5(1)	12(1)

Crystal Structure Determination of *rac*-1-Na

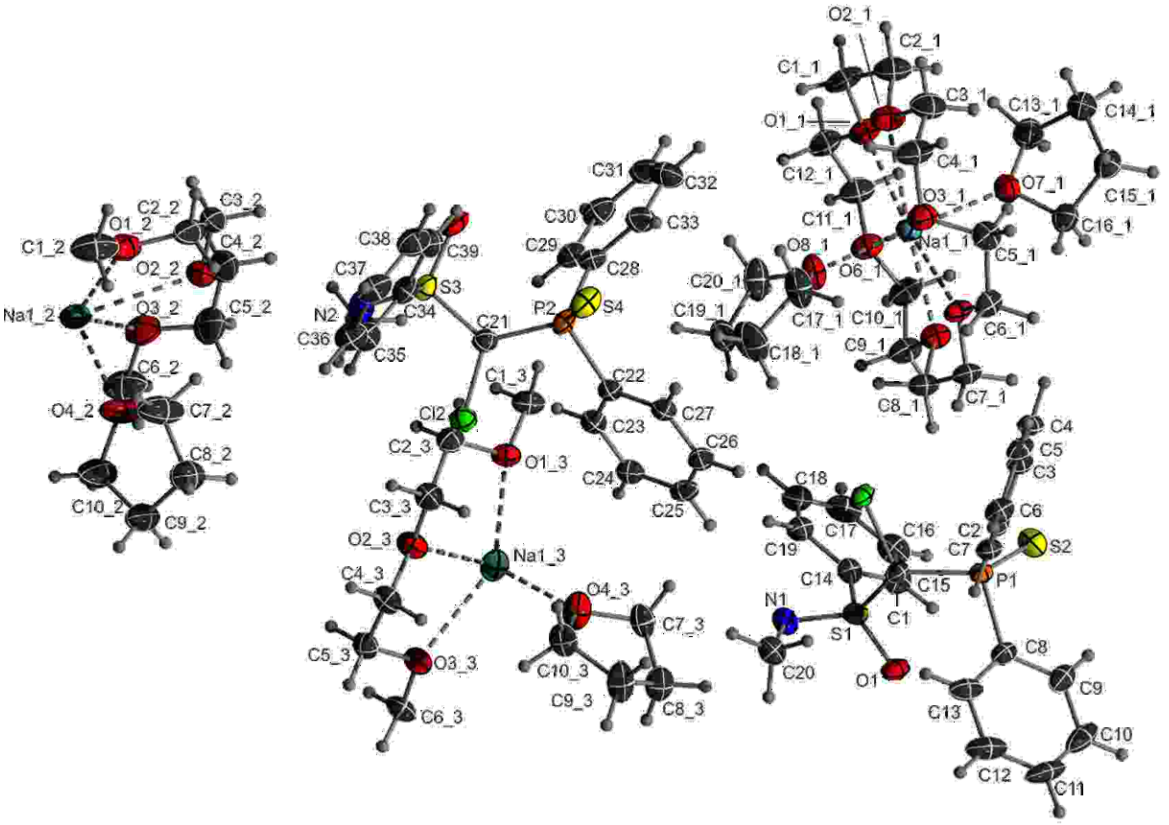

Figure S42. ORTEP of compound *rac*-**1-Na**. Ellipsoids are drawn at the 50% probability level.

Table S25. Atomic coordinates (x 10^4) and equivalent isotropic displacement parameters (Å^2 x 10^3) for *rac*-**1-Na**. U(eq) is defined as one third of the trace of the orthogonalized U^{ij} tensor.

	x	y	z	U(eq)
Cl(1)	5297(1)	1768(1)	3051(1)	27(1)
S(1)	6898(1)	1295(1)	2077(1)	24(1)
P(1)	5741(1)	281(1)	3199(1)	21(1)
O(1)	7227(2)	655(1)	1907(1)	29(1)
N(1)	6416(3)	1916(1)	1645(1)	29(1)
C(1)	5677(3)	1081(1)	2711(1)	23(1)
Cl(2)	3175(1)	5885(1)	1360(1)	30(1)
O(2)	3626(3)	6907(1)	2613(1)	33(1)
S(2)	7494(1)	-31(1)	3571(1)	33(1)
P(2)	3322(1)	5396(1)	2621(1)	25(1)
N(2)	3799(3)	7396(1)	1468(1)	35(1)
C(2)	4145(3)	312(1)	3762(1)	23(1)
S(3)	3242(1)	6876(1)	2032(1)	30(1)
C(3)	4340(3)	413(2)	4297(1)	26(1)

S(4)	1287(1)	5249(1)	3047(1)	34(1)
C(4)	3134(4)	479(2)	4716(1)	27(1)
C(5)	1711(4)	436(2)	4605(1)	29(1)
C(6)	1502(4)	329(2)	4077(2)	31(1)
C(7)	2714(3)	270(2)	3656(1)	26(1)
C(8)	5213(4)	-332(2)	2825(1)	28(1)
C(9)	5733(4)	-993(2)	2961(2)	39(1)
C(10)	5214(5)	-1474(2)	2716(2)	52(1)
C(11)	4209(5)	-1306(2)	2339(2)	54(1)
C(12)	3724(4)	-646(2)	2192(2)	48(1)
C(13)	4231(4)	-163(2)	2432(2)	35(1)
C(14)	8590(3)	1584(2)	2198(1)	26(1)
C(15)	9674(4)	1104(2)	2333(2)	34(1)
C(16)	11020(4)	1309(2)	2417(2)	39(1)
C(17)	11269(4)	1983(2)	2373(2)	40(1)
C(18)	10172(4)	2459(2)	2255(2)	35(1)
C(19)	8816(3)	2257(2)	2171(1)	29(1)
C(20)	4964(4)	1873(2)	1482(2)	34(1)
C(21)	3724(4)	6062(2)	1986(1)	28(1)
C(22)	4017(3)	4643(2)	2373(1)	23(1)
C(23)	5285(3)	4670(2)	1958(1)	28(1)
C(24)	5833(4)	4091(2)	1789(1)	30(1)
C(25)	5127(4)	3476(2)	2039(1)	30(1)
C(26)	3870(4)	3449(2)	2456(1)	29(1)
C(27)	3306(3)	4029(2)	2620(1)	25(1)
C(28)	4620(3)	5446(2)	3112(1)	26(1)
C(29)	6042(4)	5693(2)	2904(2)	33(1)
C(30)	7044(4)	5657(2)	3283(2)	41(1)
C(31)	6599(5)	5393(2)	3877(2)	47(1)
C(32)	5182(5)	5158(2)	4090(2)	51(1)
C(33)	4200(4)	5176(2)	3707(2)	39(1)
C(34)	1292(4)	7024(2)	2051(2)	33(1)
C(35)	674(4)	7154(2)	1545(2)	37(1)
C(36)	-848(4)	7273(2)	1583(2)	39(1)
C(37)	-1694(4)	7264(2)	2120(2)	40(1)
C(38)	-1059(4)	7132(2)	2617(2)	41(1)
C(39)	443(4)	7005(2)	2585(2)	37(1)
C(40)	5368(4)	7393(2)	1235(2)	41(1)
Na11	9081(1)	2667(1)	4848(1)	28(1)
O11	10339(3)	3581(1)	5159(1)	34(1)
C11	9450(4)	4011(2)	5459(2)	43(1)

O21	7260(3)	3377(1)	5628(1)	39(1)
C21	8315(4)	3579(2)	5915(2)	45(1)
O31	5284(3)	2539(1)	5321(1)	40(1)
C31	6072(4)	3029(2)	6053(2)	45(1)
O41	7332(2)	1781(1)	4727(1)	27(1)
C41	4874(5)	2921(2)	5748(2)	48(1)
O51	10201(2)	1923(1)	4188(1)	29(1)
C51	5533(4)	1843(2)	5555(2)	37(1)
O61	11677(2)	3028(1)	4275(1)	32(1)
C61	5989(4)	1508(2)	5067(2)	31(1)
O71	9978(3)	1952(1)	5645(1)	39(1)
C71	7883(4)	1406(2)	4304(1)	30(1)
O81	8176(2)	3351(1)	4064(1)	33(1)
C81	9102(4)	1783(2)	3873(2)	32(1)
C91	11518(4)	2160(2)	3804(2)	37(1)
C101	12463(4)	2459(2)	4132(2)	34(1)
C111	12505(4)	3403(2)	4533(2)	38(1)
C121	11505(4)	3921(2)	4747(2)	37(1)
C131	10811(5)	2026(2)	6080(2)	45(1)
C141	9953(5)	1633(2)	6660(2)	46(1)
C151	9144(4)	1100(2)	6482(2)	35(1)
C161	9603(4)	1252(2)	5821(2)	36(1)
C171	6664(4)	3390(2)	3988(2)	45(1)
C181	6688(6)	3587(3)	3335(2)	66(1)
C191	8180(5)	3904(2)	3085(2)	50(1)
C201	8914(4)	3870(2)	3610(2)	48(1)
Na12	0	10000	0	37(1)
O12	-2102(3)	9835(1)	934(1)	46(1)
C12	-3541(5)	10087(3)	871(2)	58(1)
O22	281(4)	9020(1)	1022(1)	50(1)
C22	-2099(6)	9289(2)	1431(2)	56(1)
O32	2662(3)	9340(2)	98(1)	51(1)
C32	-589(6)	9146(2)	1534(2)	57(1)
O42	-845(3)	9107(2)	-314(1)	52(1)
C42	1768(6)	8863(2)	1091(2)	62(1)
C52	2580(6)	8726(2)	535(2)	63(1)
C62	3417(5)	9253(3)	-446(2)	59(1)
C72	-2056(6)	8706(3)	37(2)	70(2)
C82	-2223(5)	8133(2)	-243(2)	52(1)
C92	-1433(5)	8377(2)	-860(2)	48(1)
C102	-757(7)	9025(3)	-879(2)	68(2)

Na13	10000	5000	0	34(1)
O13	8360(2)	5391(1)	956(1)	28(1)
C13	9217(4)	5445(2)	1384(2)	34(1)
O23	7590(2)	5836(1)	-159(1)	30(1)
C23	7503(4)	5979(2)	798(2)	32(1)
O33	8914(2)	5249(1)	-1025(1)	28(1)
C33	6603(4)	5891(2)	353(1)	32(1)
O43	8724(3)	3979(1)	286(1)	43(1)
C43	6827(4)	5796(2)	-619(2)	32(1)
C53	7936(4)	5801(2)	-1153(2)	31(1)
C63	9919(4)	5195(2)	-1536(1)	32(1)
C73	9138(4)	3316(2)	576(2)	38(1)
C83	8435(4)	2844(2)	302(2)	44(1)
C93	6981(4)	3196(2)	200(2)	50(1)
C103	7310(4)	3941(2)	119(2)	38(1)

Table S26. Anisotropic displacement parameters (Å^2 x 10^3) for *rac*-**1-Na**. The anisotropic displacement factor exponent takes the form: -2□^{2}[h^2 $a^{*2}U^{11}$ + ... + 2 h k a* b* U^{12}]

	U^{11}	U^{22}	U^{33}	U^{23}	U^{13}	U^{12}
Cl(1)	29(1)	21(1)	29(1)	-10(1)	4(1)	-1(1)
S(1)	21(1)	26(1)	23(1)	-8(1)	2(1)	-3(1)
P(1)	20(1)	22(1)	21(1)	-7(1)	2(1)	-2(1)
O(1)	30(1)	30(1)	28(1)	-14(1)	6(1)	-4(1)
N(1)	24(1)	34(1)	26(1)	-5(1)	-1(1)	-6(1)
C(1)	25(2)	19(1)	26(2)	-10(1)	1(1)	0(1)
Cl(2)	29(1)	32(1)	32(1)	-10(1)	-8(1)	0(1)
O(2)	42(1)	24(1)	38(1)	-16(1)	-5(1)	-2(1)
S(2)	24(1)	42(1)	29(1)	-8(1)	-3(1)	7(1)
P(2)	20(1)	21(1)	32(1)	-7(1)	0(1)	-3(1)
N(2)	25(1)	32(2)	47(2)	-3(1)	-9(1)	1(1)
C(2)	25(2)	16(1)	25(1)	-5(1)	2(1)	-3(1)
S(3)	30(1)	23(1)	38(1)	-10(1)	-8(1)	-1(1)
C(3)	24(2)	26(2)	30(2)	-11(1)	-2(1)	-4(1)
S(4)	23(1)	31(1)	47(1)	-13(1)	6(1)	-5(1)
C(4)	31(2)	29(2)	23(1)	-12(1)	3(1)	-4(1)
C(5)	28(2)	24(2)	32(2)	-12(1)	9(1)	-4(1)
C(6)	23(2)	31(2)	39(2)	-14(1)	1(1)	-3(1)
C(7)	25(2)	28(2)	27(2)	-12(1)	-1(1)	-1(1)
C(8)	28(2)	25(2)	29(2)	-11(1)	8(1)	-6(1)

C(9)	49(2)	26(2)	34(2)	-8(1)	19(2)	-5(2)
C(10)	71(3)	26(2)	49(2)	-18(2)	33(2)	-13(2)
C(11)	64(3)	50(2)	53(2)	-38(2)	23(2)	-30(2)
C(12)	42(2)	66(3)	45(2)	-35(2)	10(2)	-23(2)
C(13)	29(2)	44(2)	33(2)	-21(2)	8(1)	-12(1)
C(14)	19(2)	30(2)	24(2)	-5(1)	6(1)	-5(1)
C(15)	27(2)	36(2)	36(2)	-8(1)	4(1)	0(1)
C(16)	24(2)	55(2)	35(2)	-11(2)	-1(1)	5(2)
C(17)	26(2)	61(2)	34(2)	-18(2)	3(1)	-11(2)
C(18)	31(2)	42(2)	31(2)	-12(1)	3(1)	-16(2)
C(19)	23(2)	32(2)	29(2)	-10(1)	3(1)	-4(1)
C(20)	28(2)	41(2)	32(2)	-6(1)	-3(1)	-2(1)
C(21)	29(2)	29(2)	29(2)	-9(1)	-6(1)	-2(1)
C(22)	18(1)	25(2)	26(1)	-7(1)	-6(1)	-1(1)
C(23)	24(2)	33(2)	28(2)	-7(1)	-6(1)	-5(1)
C(24)	26(2)	36(2)	31(2)	-13(1)	-6(1)	3(1)
C(25)	29(2)	34(2)	35(2)	-17(1)	-14(1)	5(1)
C(26)	31(2)	29(2)	31(2)	-9(1)	-10(1)	-7(1)
C(27)	21(2)	29(2)	26(2)	-7(1)	-6(1)	-3(1)
C(28)	26(2)	27(2)	30(2)	-13(1)	-4(1)	-1(1)
C(29)	29(2)	31(2)	41(2)	-14(1)	-4(1)	1(1)
C(30)	36(2)	37(2)	57(2)	-20(2)	-13(2)	0(2)
C(31)	59(3)	46(2)	50(2)	-23(2)	-30(2)	3(2)
C(32)	65(3)	57(3)	37(2)	-17(2)	-18(2)	-6(2)
C(33)	43(2)	41(2)	34(2)	-12(2)	-5(2)	-9(2)
C(34)	30(2)	27(2)	43(2)	-10(1)	-5(2)	4(1)
C(35)	32(2)	36(2)	41(2)	-11(2)	-4(2)	0(1)
C(36)	30(2)	42(2)	50(2)	-17(2)	-10(2)	-1(2)
C(37)	27(2)	40(2)	56(2)	-23(2)	-3(2)	0(1)
C(38)	36(2)	42(2)	47(2)	-18(2)	2(2)	-1(2)
C(39)	38(2)	38(2)	38(2)	-10(2)	-9(2)	4(2)
C(40)	29(2)	33(2)	53(2)	-1(2)	-2(2)	-2(1)
Na11	30(1)	27(1)	30(1)	-8(1)	-11(1)	0(1)
O11	30(1)	34(1)	41(1)	-20(1)	1(1)	-3(1)
C11	35(2)	40(2)	62(2)	-34(2)	2(2)	-3(2)
O21	35(1)	44(1)	40(1)	-20(1)	-1(1)	-7(1)
C21	41(2)	56(2)	45(2)	-29(2)	-2(2)	0(2)
O31	45(2)	39(1)	39(1)	-17(1)	-2(1)	-1(1)
C31	45(2)	57(2)	36(2)	-22(2)	4(2)	-6(2)
O41	23(1)	28(1)	30(1)	-13(1)	2(1)	-7(1)
C41	45(2)	55(2)	49(2)	-30(2)	5(2)	-8(2)

O51	20(1)	33(1)	34(1)	-14(1)	3(1)	-3(1)
C51	29(2)	41(2)	36(2)	-9(2)	7(1)	-5(1)
O61	22(1)	35(1)	41(1)	-18(1)	-4(1)	-1(1)
C61	24(2)	30(2)	39(2)	-9(1)	3(1)	-10(1)
O71	47(2)	33(1)	39(1)	-6(1)	-17(1)	1(1)
C71	29(2)	31(2)	33(2)	-17(1)	0(1)	-5(1)
O81	22(1)	37(1)	33(1)	7(1)	-7(1)	-5(1)
C81	30(2)	39(2)	32(2)	-18(1)	-1(1)	-4(1)
C91	29(2)	39(2)	43(2)	-19(2)	11(2)	-9(1)
C101	22(2)	37(2)	45(2)	-17(2)	5(1)	-1(1)
C111	24(2)	45(2)	50(2)	-24(2)	-5(2)	-7(1)
C121	27(2)	35(2)	52(2)	-19(2)	-1(2)	-12(1)
C131	47(2)	40(2)	50(2)	-9(2)	-19(2)	-4(2)
C141	59(3)	41(2)	39(2)	-10(2)	-13(2)	-2(2)
C151	32(2)	34(2)	39(2)	-9(2)	-3(1)	3(1)
C161	37(2)	31(2)	40(2)	-10(2)	-9(2)	6(1)
C171	24(2)	51(2)	50(2)	10(2)	-11(2)	-5(2)
C181	62(3)	79(3)	58(3)	-4(2)	-37(2)	-1(3)
C191	70(3)	42(2)	30(2)	-1(2)	1(2)	12(2)
C201	28(2)	44(2)	57(2)	13(2)	0(2)	-4(2)
Na12	41(1)	42(1)	33(1)	-18(1)	0(1)	-7(1)
O12	53(2)	45(2)	37(1)	-17(1)	12(1)	-12(1)
C12	33(2)	85(3)	66(3)	-46(3)	10(2)	-8(2)
O22	82(2)	39(2)	34(1)	-12(1)	-20(1)	2(1)
C22	90(4)	41(2)	33(2)	-14(2)	16(2)	-26(2)
O32	50(2)	52(2)	58(2)	-26(1)	-16(1)	16(1)
C32	107(4)	35(2)	27(2)	-5(2)	-9(2)	-16(2)
O42	52(2)	72(2)	38(1)	-31(1)	8(1)	-26(1)
C42	94(4)	44(2)	60(3)	-11(2)	-48(3)	14(2)
C52	73(3)	48(3)	73(3)	-15(2)	-33(3)	23(2)
C62	44(2)	74(3)	69(3)	-41(3)	-6(2)	17(2)
C72	73(3)	91(4)	50(3)	-37(3)	18(2)	-41(3)
C82	55(3)	54(2)	47(2)	-16(2)	4(2)	-15(2)
C92	51(2)	50(2)	44(2)	-19(2)	0(2)	-11(2)
C102	95(4)	63(3)	43(2)	-22(2)	18(2)	-35(3)
Na13	26(1)	34(1)	42(1)	-6(1)	-5(1)	-7(1)
O13	22(1)	35(1)	30(1)	-13(1)	-5(1)	-1(1)
C13	25(2)	46(2)	34(2)	-18(2)	-2(1)	-4(1)
O23	19(1)	44(1)	28(1)	-11(1)	-4(1)	-1(1)
C23	30(2)	34(2)	32(2)	-11(1)	1(1)	0(1)
O33	20(1)	37(1)	24(1)	-5(1)	-3(1)	-1(1)

C33	23(2)	39(2)	32(2)	-9(1)	-1(1)	5(1)
O43	28(1)	36(1)	61(2)	1(1)	-19(1)	-6(1)
C43	23(2)	41(2)	35(2)	-8(1)	-11(1)	2(1)
C53	29(2)	34(2)	32(2)	-5(1)	-12(1)	1(1)
C63	22(2)	50(2)	23(2)	-7(1)	-1(1)	-6(1)
C73	31(2)	39(2)	38(2)	4(2)	-8(2)	-5(1)
C83	37(2)	40(2)	53(2)	-3(2)	-12(2)	-6(2)
C93	35(2)	44(2)	64(3)	10(2)	-19(2)	-15(2)
C103	26(2)	43(2)	43(2)	-3(2)	-12(2)	-3(2)

Crystal Structure Determination of *rac*-1-K

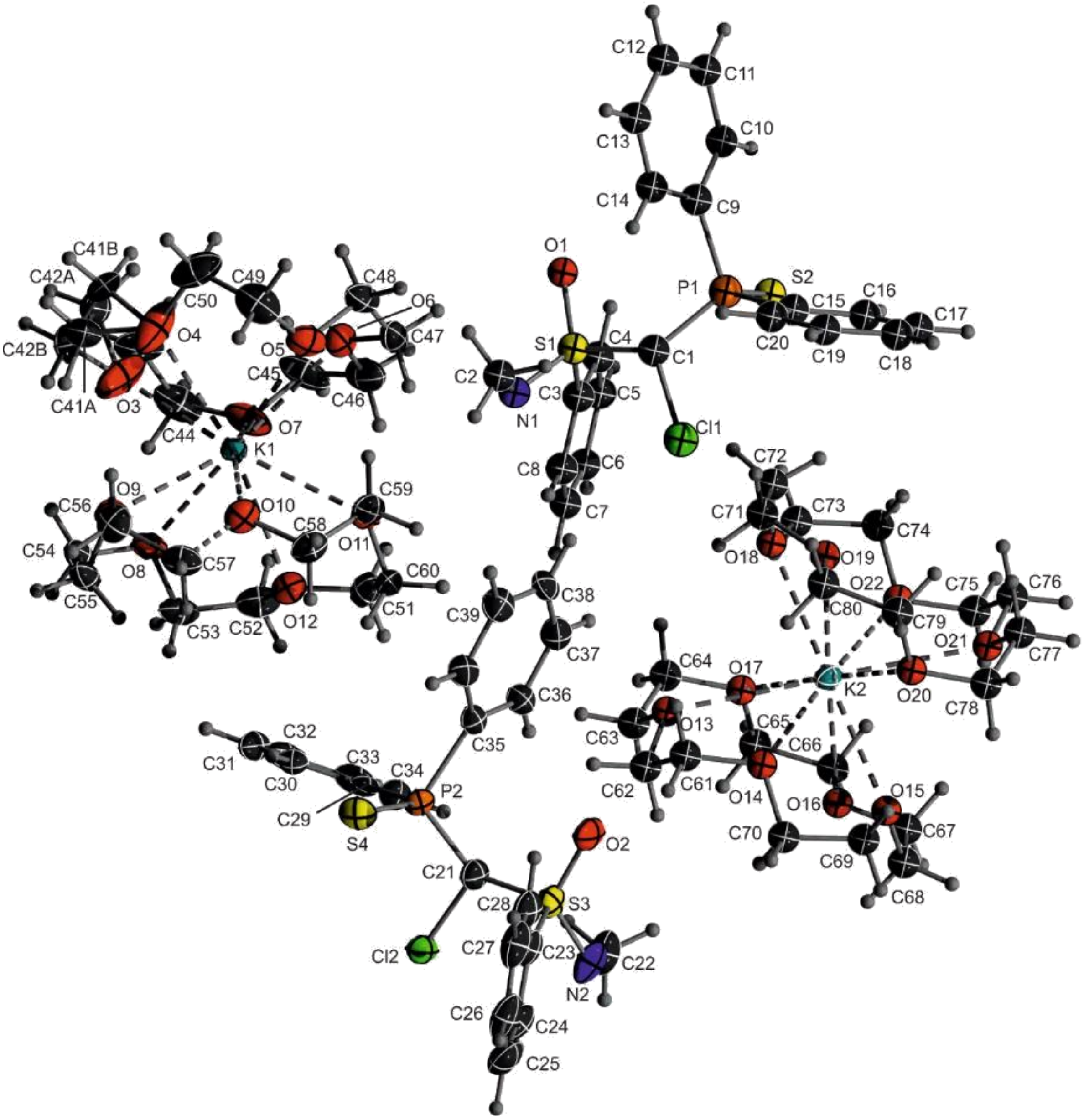

Figure S43. ORTEP of compound *rac*-**1-K**. Ellipsoids are drawn at the 50% probability level.

Table S27. Atomic coordinates (x 10^4) and equivalent isotropic displacement parameters ($Å^2$x 10^3) for *rac*-**1-K** U(eq) is defined as one third of the trace of the orthogonalized U^{ij} tensor.

	x	y	z	U(eq)
K(1)	611(1)	2885(1)	5268(1)	23(1)
K(2)	844(1)	2086(1)	240(1)	26(1)
Cl(1)	5143(1)	3909(1)	1892(1)	29(1)
Cl(2)	4361(2)	-1071(1)	2976(1)	33(1)
S(1)	3370(2)	4472(1)	2859(1)	29(1)

S(2)	2343(2)	5332(1)	1408(1)	38(1)
S(3)	6150(2)	-379(1)	2092(1)	31(1)
S(4)	6968(2)	15(1)	3768(1)	36(1)
P(1)	4159(1)	5281(1)	1794(1)	23(1)
P(2)	5394(1)	220(1)	3279(1)	24(1)
O(1)	2901(5)	5113(2)	3072(2)	42(1)
O(2)	6544(5)	290(2)	1925(2)	42(1)
O(3)	-1213(6)	3280(3)	6248(3)	68(2)
O(4)	1613(6)	3800(4)	6123(2)	63(2)
O(5)	2488(5)	3998(2)	4935(2)	42(1)
O(6)	-402(4)	4046(2)	4615(2)	36(1)
O(7)	-2420(5)	3151(2)	5156(3)	50(1)
O(8)	-1317(5)	1859(2)	5720(2)	36(1)
O(9)	1523(5)	2086(2)	6121(2)	39(1)
O(10)	3516(4)	2348(2)	5138(2)	35(1)
O(11)	1994(4)	2592(2)	4149(2)	32(1)
O(12)	-222(4)	1780(2)	4577(2)	32(1)
O(13)	613(6)	1464(3)	1431(2)	61(2)
O(14)	3140(6)	1213(3)	641(2)	57(1)
O(15)	1944(5)	1046(3)	-433(2)	47(1)
O(16)	-1043(5)	991(3)	-94(2)	45(1)
O(17)	-1938(5)	1777(2)	843(2)	44(1)
O(18)	-365(5)	3139(2)	901(2)	41(1)
O(19)	2635(5)	3160(3)	515(2)	43(1)
O(20)	3745(5)	2341(2)	-343(2)	40(1)
O(21)	1229(5)	2683(2)	-936(2)	38(1)
O(22)	-1430(4)	2885(2)	-207(2)	36(1)
N(1)	4016(5)	3978(3)	3251(2)	34(1)
N(2)	5509(5)	-837(3)	1667(2)	42(1)
C(1)	4483(6)	4627(3)	2249(2)	27(1)
C(2)	5475(7)	4109(4)	3425(3)	46(2)
C(3)	1754(6)	4051(3)	2703(2)	30(1)
C(4)	540(7)	4423(4)	2598(3)	37(1)
C(5)	-758(7)	4101(4)	2493(3)	44(2)
C(6)	-821(7)	3447(4)	2486(3)	45(2)
C(7)	395(8)	3081(4)	2586(3)	46(2)
C(8)	1684(6)	3385(3)	2701(3)	35(1)
C(9)	4458(6)	6029(3)	2189(2)	26(1)
C(10)	3709(7)	6584(3)	2082(3)	37(1)
C(11)	4059(9)	7175(3)	2345(3)	46(2)

C(12)	5121(9)	7190(3)	2712(3)	47(2)
C(13)	5875(8)	6633(3)	2836(3)	43(2)
C(14)	5534(6)	6058(3)	2578(3)	36(1)
C(15)	5763(5)	5272(3)	1263(2)	25(1)
C(16)	5583(7)	5363(3)	689(2)	35(1)
C(17)	6802(8)	5369(3)	280(3)	47(2)
C(18)	8152(7)	5283(3)	456(3)	44(2)
C(19)	8349(7)	5185(3)	1029(4)	49(2)
C(20)	7143(6)	5182(3)	1442(3)	35(1)
C(21)	5052(6)	-305(3)	2714(2)	32(1)
C(22)	3991(7)	-737(4)	1540(3)	47(2)
C(23)	7800(6)	-814(3)	2205(2)	34(1)
C(24)	7856(7)	-1482(4)	2171(3)	41(2)
C(25)	9164(9)	-1806(4)	2225(3)	49(2)
C(26)	10410(7)	-1438(4)	2315(3)	48(2)
C(27)	10308(7)	-789(4)	2367(3)	48(2)
C(28)	8999(7)	-463(4)	2313(3)	38(1)
C(29)	3621(5)	242(3)	3707(2)	25(1)
C(30)	3539(6)	308(3)	4297(2)	30(1)
C(31)	2149(7)	352(3)	4606(3)	40(2)
C(32)	900(6)	317(3)	4325(3)	37(1)
C(33)	994(6)	248(3)	3738(3)	40(2)
C(34)	2341(6)	209(3)	3425(3)	33(1)
C(35)	5545(6)	1051(3)	3009(2)	26(1)
C(36)	4554(6)	1313(3)	2660(2)	31(1)
C(37)	4615(7)	1962(3)	2504(3)	37(1)
C(38)	5653(7)	2365(3)	2693(3)	36(1)
C(39)	6663(7)	2111(4)	3043(3)	39(1)
C(40)	6609(6)	1466(3)	3193(3)	32(1)
C(43)	-2547(9)	3569(4)	6134(4)	66(3)
C(44)	-3218(7)	3149(4)	5717(4)	56(2)
C(45)	-2815(8)	3666(4)	4790(4)	56(2)
C(46)	-1565(9)	3783(4)	4339(3)	53(2)
C(47)	844(8)	4194(3)	4230(3)	40(2)
C(48)	1966(7)	4479(3)	4568(3)	38(1)
C(49)	3226(8)	4234(4)	5389(4)	54(2)
C(50)	2233(9)	4396(4)	5912(4)	61(2)
C(51)	18(7)	1921(3)	3980(3)	38(1)
C(52)	-1688(7)	1585(3)	4760(3)	42(2)
C(53)	-1701(7)	1344(3)	5361(3)	39(2)

C(54)	-818(7)	1642(3)	6255(3)	40(2)
C(55)	772(7)	1485(3)	6201(3)	40(2)
C(56)	3080(7)	2026(4)	6111(3)	44(2)
C(57)	3786(7)	1842(3)	5531(3)	41(2)
C(58)	4177(6)	2221(3)	4571(3)	38(1)
C(59)	3514(7)	2665(3)	4162(3)	34(1)
C(60)	1599(7)	2040(3)	3838(2)	34(1)
C(61)	3120(8)	1139(5)	1249(3)	53(2)
C(62)	1649(9)	949(4)	1511(4)	57(2)
C(63)	-855(8)	1282(4)	1636(3)	49(2)
C(64)	-1893(9)	1754(4)	1455(3)	55(2)
C(65)	-2854(8)	1318(4)	617(4)	51(2)
C(66)	-2472(7)	1270(4)	-23(3)	40(1)
C(67)	-486(7)	906(4)	-666(3)	39(2)
C(68)	990(7)	621(4)	-681(3)	38(1)
C(69)	3266(7)	764(4)	-285(3)	44(2)
C(70)	3267(9)	624(4)	340(3)	52(2)
C(71)	2056(7)	3393(3)	1067(3)	38(1)
C(72)	584(7)	3659(4)	1015(3)	39(1)
C(73)	-1730(7)	3362(3)	717(3)	42(2)
C(74)	-1683(7)	3492(3)	78(3)	38(1)
C(75)	-1305(8)	2940(4)	-822(3)	45(2)
C(76)	194(8)	3151(3)	-1062(3)	42(2)
C(77)	2668(8)	2876(4)	-1139(3)	42(2)
C(78)	3715(8)	2373(4)	-951(3)	45(2)
C(79)	4627(7)	2826(4)	-117(3)	46(2)
C(80)	4109(7)	2926(4)	503(3)	50(2)
C(41A)	680(30)	3606(11)	6655(8)	56(7)
C(42A)	-800(30)	3838(12)	6574(9)	66(8)
C(41B)	504(11)	4050(7)	6569(5)	31(4)
C(42B)	-372(13)	3463(8)	6739(5)	34(4)

Table S28. Anisotropic displacement parameters (Å^{2}x 10^3) for *rac*-**1-K**. The anisotropic displacement factor exponent takes the form: -2□^{2}[h^2 a*^{2}U^{11} + ... + 2 h k a* b* U^{12}]

	U^{11}	U^{22}	U^{33}	U^{23}	U^{13}	U^{12}
K(1)	23(1)	20(1)	24(1)	1(1)	4(1)	5(1)
K(2)	26(1)	24(1)	26(1)	-4(1)	3(1)	-1(1)
Cl(1)	31(1)	24(1)	31(1)	0(1)	1(1)	2(1)
Cl(2)	35(1)	27(1)	37(1)	-3(1)	5(1)	-2(1)

S(1)	33(1)	29(1)	23(1)	2(1)	2(1)	-2(1)
S(2)	26(1)	48(1)	41(1)	12(1)	-9(1)	-6(1)
S(3)	30(1)	36(1)	26(1)	0(1)	6(1)	2(1)
S(4)	27(1)	36(1)	46(1)	-1(1)	-9(1)	7(1)
P(1)	21(1)	23(1)	24(1)	3(1)	0(1)	-1(1)
P(2)	18(1)	26(1)	28(1)	-3(1)	0(1)	3(1)
O(1)	54(3)	36(3)	32(2)	4(2)	16(2)	0(2)
O(2)	50(3)	38(2)	34(2)	-7(2)	20(2)	-8(2)
O(3)	45(3)	82(4)	69(4)	-39(3)	27(3)	-19(3)
O(4)	38(3)	102(5)	46(3)	-26(3)	5(2)	-18(3)
O(5)	35(2)	35(2)	54(3)	-1(2)	5(2)	6(2)
O(6)	32(2)	38(2)	37(2)	2(2)	1(2)	3(2)
O(7)	39(2)	34(3)	79(4)	11(2)	-11(2)	4(2)
O(8)	36(2)	24(2)	46(2)	-1(2)	11(2)	2(2)
O(9)	43(2)	36(2)	37(2)	1(2)	1(2)	11(2)
O(10)	26(2)	41(2)	39(2)	-3(2)	-1(2)	4(2)
O(11)	33(2)	27(2)	34(2)	-1(2)	9(2)	4(2)
O(12)	25(2)	35(2)	36(2)	-4(2)	-6(2)	1(2)
O(13)	44(3)	90(5)	47(3)	11(3)	5(2)	-18(3)
O(14)	62(3)	62(4)	48(3)	-1(3)	-13(2)	16(3)
O(15)	36(2)	48(3)	57(3)	-13(2)	-8(2)	10(2)
O(16)	33(2)	53(3)	49(3)	-9(2)	3(2)	3(2)
O(17)	42(2)	41(3)	45(3)	-6(2)	19(2)	-10(2)
O(18)	44(2)	36(2)	43(2)	-3(2)	6(2)	-2(2)
O(19)	33(2)	54(3)	43(2)	-9(2)	-1(2)	-1(2)
O(20)	35(2)	35(2)	50(3)	-3(2)	5(2)	-5(2)
O(21)	47(2)	30(2)	37(2)	0(2)	1(2)	-1(2)
O(22)	30(2)	25(2)	51(3)	0(2)	-8(2)	1(2)
N(1)	33(2)	41(3)	28(2)	4(2)	-9(2)	-2(2)
N(2)	26(2)	67(4)	32(3)	-13(3)	-2(2)	4(2)
C(1)	40(3)	21(3)	19(2)	2(2)	1(2)	2(2)
C(2)	39(3)	64(5)	36(3)	1(3)	-13(3)	-3(3)
C(3)	23(2)	47(4)	19(2)	6(2)	8(2)	2(2)
C(4)	38(3)	41(4)	29(3)	7(3)	6(2)	13(3)
C(5)	26(3)	67(5)	36(3)	24(3)	10(2)	7(3)
C(6)	28(3)	67(5)	41(4)	24(3)	-3(2)	-13(3)
C(7)	44(4)	44(4)	48(4)	10(3)	1(3)	-6(3)
C(8)	30(3)	36(3)	39(3)	9(3)	-3(2)	2(2)
C(9)	27(2)	21(3)	28(3)	-4(2)	11(2)	-1(2)
C(10)	44(3)	35(3)	30(3)	3(3)	7(2)	11(3)

C(11)	66(4)	21(3)	48(4)	3(3)	5(3)	22(3)
C(12)	63(4)	29(4)	46(4)	-7(3)	10(3)	-5(3)
C(13)	44(4)	39(4)	45(4)	-7(3)	1(3)	-1(3)
C(14)	28(3)	35(3)	45(3)	-9(3)	-4(2)	2(2)
C(15)	21(2)	21(3)	33(3)	-3(2)	1(2)	-5(2)
C(16)	38(3)	41(4)	24(3)	-1(2)	7(2)	-12(3)
C(17)	61(4)	40(4)	35(3)	-4(3)	20(3)	-17(3)
C(18)	47(4)	27(3)	52(4)	-9(3)	31(3)	-5(3)
C(19)	27(3)	26(3)	91(6)	3(3)	9(3)	3(2)
C(20)	25(3)	28(3)	51(4)	4(3)	2(2)	0(2)
C(21)	30(3)	34(3)	30(3)	-2(2)	6(2)	-3(2)
C(22)	37(3)	71(5)	34(3)	-2(3)	-2(3)	1(3)
C(23)	27(3)	49(4)	22(3)	-6(2)	7(2)	4(2)
C(24)	30(3)	56(4)	37(3)	-14(3)	0(2)	-2(3)
C(25)	58(4)	52(4)	37(3)	-15(3)	0(3)	14(3)
C(26)	33(3)	80(6)	31(3)	-12(3)	1(2)	19(3)
C(27)	33(3)	81(6)	29(3)	-2(3)	0(2)	-3(3)
C(28)	33(3)	49(4)	30(3)	-1(3)	10(2)	3(3)
C(29)	23(2)	17(3)	34(3)	2(2)	5(2)	-2(2)
C(30)	35(3)	25(3)	30(3)	-2(2)	-3(2)	-5(2)
C(31)	47(4)	31(3)	38(3)	-2(3)	21(3)	-8(3)
C(32)	29(3)	25(3)	55(4)	4(3)	16(3)	-3(2)
C(33)	23(3)	28(3)	67(4)	8(3)	4(3)	1(2)
C(34)	26(3)	33(3)	38(3)	0(3)	1(2)	1(2)
C(35)	22(2)	30(3)	26(3)	2(2)	2(2)	1(2)
C(36)	29(3)	31(3)	33(3)	3(2)	-1(2)	1(2)
C(37)	34(3)	41(4)	34(3)	2(3)	-1(2)	6(3)
C(38)	45(3)	29(3)	32(3)	8(2)	9(3)	0(3)
C(39)	34(3)	44(4)	39(3)	1(3)	2(2)	-10(3)
C(40)	27(3)	35(3)	35(3)	2(2)	-2(2)	-6(2)
C(43)	56(5)	36(4)	94(6)	-10(4)	51(5)	1(3)
C(44)	27(3)	43(4)	94(6)	17(4)	12(3)	6(3)
C(45)	37(3)	33(4)	101(6)	18(4)	-27(4)	-1(3)
C(46)	69(5)	38(4)	58(4)	0(3)	-34(4)	6(3)
C(47)	50(4)	35(3)	32(3)	6(3)	13(3)	11(3)
C(48)	34(3)	30(3)	48(4)	3(3)	14(3)	7(2)
C(49)	34(3)	52(5)	76(5)	11(4)	-16(3)	-7(3)
C(50)	51(4)	62(5)	73(5)	-31(4)	-25(4)	11(4)
C(51)	41(3)	45(4)	30(3)	-4(3)	-12(2)	9(3)
C(52)	24(3)	37(4)	67(4)	-11(3)	-6(3)	-4(3)

C(53)	28(3)	26(3)	61(4)	-4(3)	13(3)	-3(2)
C(54)	46(3)	30(3)	39(3)	8(3)	18(3)	8(3)
C(55)	49(4)	29(3)	38(3)	7(3)	14(3)	14(3)
C(56)	37(3)	53(4)	43(4)	-1(3)	-13(3)	4(3)
C(57)	32(3)	33(3)	59(4)	-1(3)	-12(3)	7(3)
C(58)	27(3)	42(4)	44(3)	-9(3)	4(2)	4(3)
C(59)	32(3)	31(3)	37(3)	-2(2)	9(2)	2(2)
C(60)	49(3)	27(3)	25(3)	-1(2)	3(2)	6(3)
C(61)	49(4)	70(5)	42(4)	3(4)	-7(3)	2(4)
C(62)	51(4)	63(5)	60(5)	23(4)	-19(3)	-8(4)
C(63)	51(4)	57(5)	37(4)	1(3)	10(3)	-21(3)
C(64)	63(5)	53(5)	42(4)	-10(3)	20(3)	-13(4)
C(65)	35(3)	41(4)	73(5)	5(4)	12(3)	-8(3)
C(66)	26(3)	41(4)	53(4)	-3(3)	-1(3)	0(3)
C(67)	39(3)	50(4)	26(3)	-8(3)	1(2)	10(3)
C(68)	33(3)	51(4)	28(3)	-9(3)	6(2)	-3(3)
C(69)	37(3)	52(4)	44(4)	-4(3)	-4(3)	12(3)
C(70)	57(4)	40(4)	59(5)	7(3)	-4(3)	15(3)
C(71)	39(3)	39(4)	35(3)	-11(3)	-4(2)	-4(3)
C(72)	38(3)	45(4)	33(3)	-18(3)	0(2)	-5(3)
C(73)	31(3)	30(3)	64(4)	-11(3)	-3(3)	-1(2)
C(74)	27(3)	29(3)	56(4)	-1(3)	-1(3)	0(2)
C(75)	54(4)	37(4)	46(4)	8(3)	-19(3)	-1(3)
C(76)	58(4)	28(3)	40(3)	1(3)	-10(3)	2(3)
C(77)	51(4)	39(4)	35(3)	2(3)	8(3)	-13(3)
C(78)	46(4)	41(4)	44(4)	-9(3)	16(3)	-4(3)
C(79)	33(3)	38(4)	69(5)	-9(3)	-5(3)	0(3)
C(80)	25(3)	63(5)	64(4)	-18(4)	-7(3)	2(3)
C(41A)	86(18)	29(12)	52(10)	0(8)	-6(10)	4(10)
C(42A)	110(20)	45(12)	38(10)	0(9)	33(11)	0(12)
C(41B)	26(5)	37(10)	31(6)	-15(5)	-1(4)	1(5)
C(42B)	25(6)	46(9)	29(6)	9(5)	6(4)	-4(5)

6. Computational Details

6.1 General

All calculations were performed without symmetry restrictions. Starting coordinates for the corresponding structures were obtained from the crystal structure analyses or modelled with GaussView 6.0.16.[3] The geometry optimization was performed with the *Gaussian 16* (Revision C.01) program package.[4] The geometry optimization was performed using Density-Functional Theory (DFT)[5] with the PBE0 functional[6] and the MWB10 basis set and the corresponding Stuttgart-Dresden ECP for Potassium[7] and the def2svp basis set[8] for all other atoms including Grimme's D3 dispersion correction with Becke-Johnson damping[9]. Solvent corrections were included by using the polarizable continuum model (PCM) for THF. Harmonic vibrational frequency analysis was performed on the same levels of theory to determine the nature of the structure.[10] The vibrational frequency analysis showed no imaginary frequencies for all calculated structures. Coordinates of all energy-optimized structures are provided as cartesian coordinates in Angstrom in the Tables below.

Single point energies were obtained with the PBE0 functional[6] and the MWB10 basis set and the corresponding Stuttgart-Dresden ECP for Potassium[7] and the def2tzvp basis set for all other atoms[11] with Grimme's D3 dispersion correction with Becke-Johnson damping[9]. Solvent corrections were included by using the polarizable continuum model (PCM) for THF and toluene, which are also the solvents used in experiments.

The energies were corrected by conversion from the standard state (1 atm) to the solution state as follows:

$$\Delta G_M^0 = \Delta G_{gas}^0 + RT\ln\left(\frac{K_M^0}{K_{gas}^0}\right)$$

with R = 8.31447 J K^{-1} mol^{-1}, T = temperature in K = 298.15 K, cGas = p/RT = 0.0408 mol/L (ideal gas),
in THF: c_M(monomer) = 0.05 mol/l, c_M(dimer) = 0.025 mol/l, c_M(THF) = 12 mol/l,
in toluene: c_M(monomer) = 0.05 mol/l, c_M(dimer) = 0.025 mol/l, c_M(THF) = 0.05 mol/l.

Table S29. Energy corrections $\Delta G_{corr}^0 = RT\ln\left(\frac{K_M^0}{K_{gas}^0}\right)$ for different monomers and dimers in THF and toluene.

**THF**		**toluene**	
monomer$(THF)_3$ + 2 THF→ monomer$(THF)_5$	–28.18	monomer$(THF)_3$ + 2 THF→ monomer$(THF)_5$	–1.01
monomer$(THF)_3$ + THF→ monomer$(THF)_4$	–14.09	monomer$(THF)_3$ + THF→ monomer$(THF)_4$	–0.50
monomer$(THF)_3$ → monomer$(THF)_2$ + THF	14.09	monomer$(THF)_3$ → monomer$(THF)_2$ + THF	0.50
monomer$(THF)_3$ → monomer(THF) + 2 THF	28.18	monomer$(THF)_3$ → monomer(THF) + 2 THF	1.01
2 monomer$(THF)_3$ → dimer$(THF)_2$ + 4 THF	54.14	2 monomer$(THF)_3$ → dimer$(THF)_2$ + 4 THF	–0.21
2 monomer$(THF)_3$ → dimer$(THF)_4$ + 2 THF	25.96	2 monomer$(THF)_3$ → dimer$(THF)_4$ + 2 THF	–1.21
2 monomer$(THF)_3$ → dimer + 6 THF	82.32	2 monomer$(THF)_3$ → dimer + 6 THF	0.80

6.2 Structures of the energy-optimized compounds

Structures of monomeric 1-Li

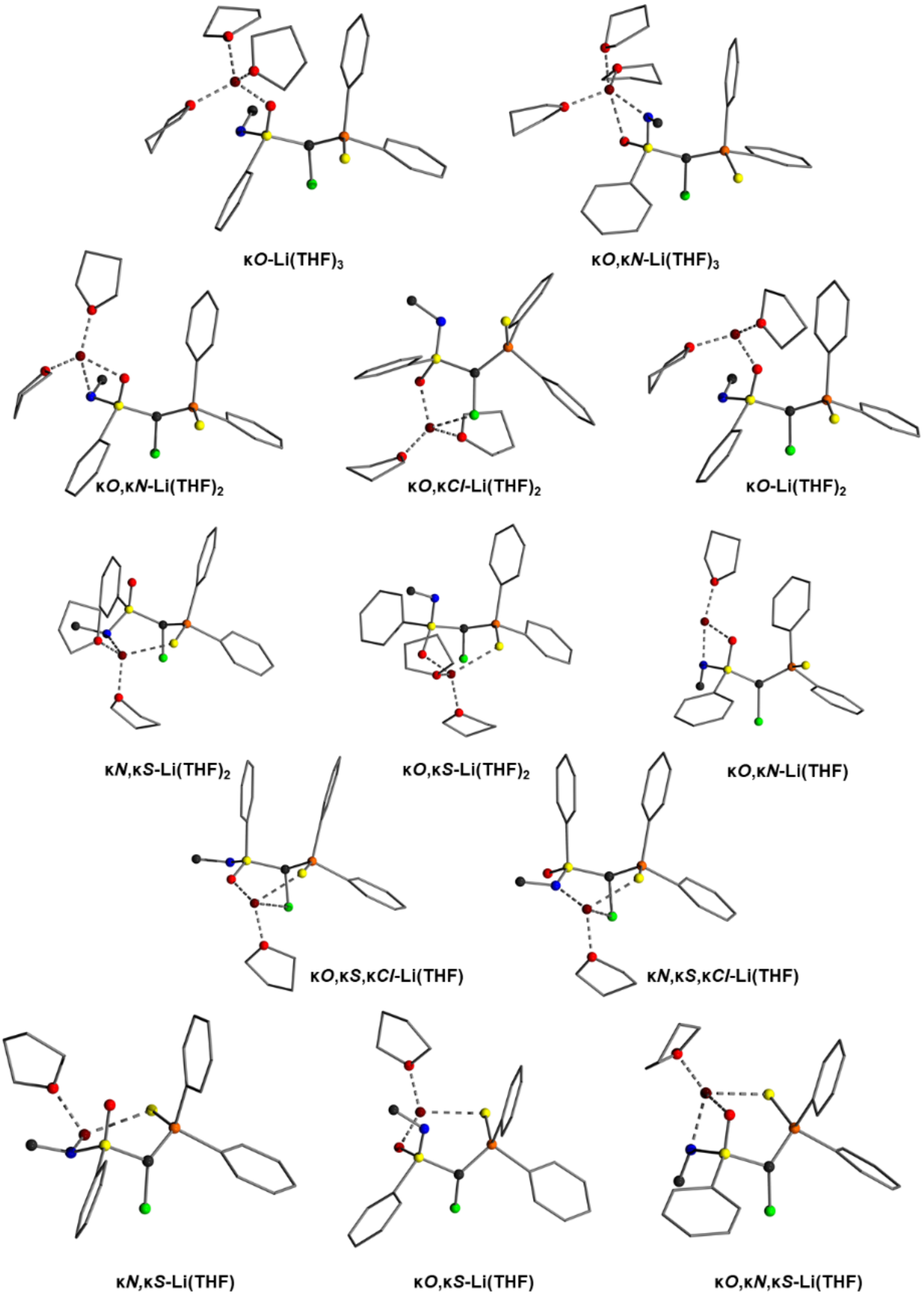

Figure S44. Li monomers.

Structures of dimeric 1-Li

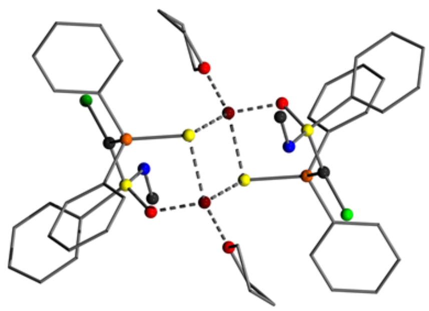

κ*O*,κ*S*-$Li_2(THF)_2$

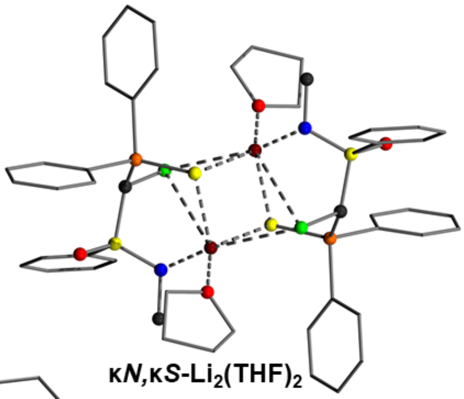

κ*N*,κ*S*-$Li_2(THF)_2$

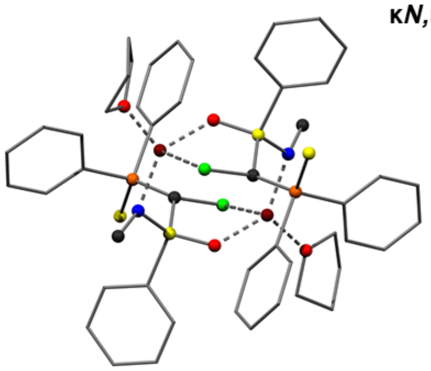

κ*O*,κ*N*,κ*Cl*-$Li_2(THF)_2$

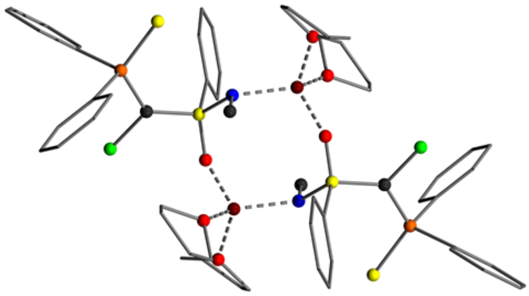

κ*O*,κ*N*-$Li_2(THF)_4$

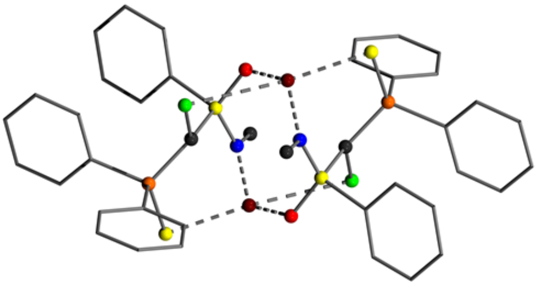

κ*O*,κ*N*,κ*S*,κ*Cl*-Li_2

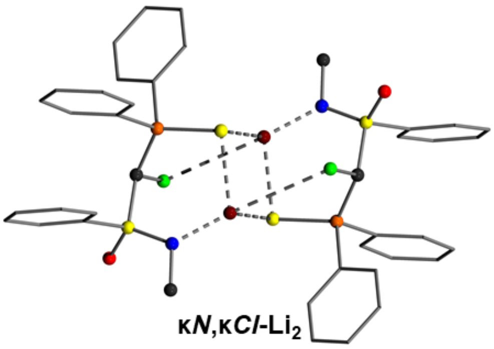

κ*N*,κ*Cl*-Li_2

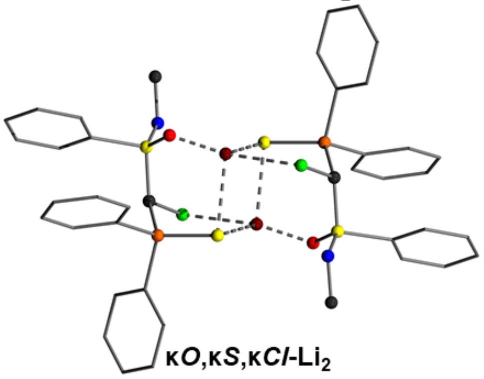

κ*O*,κ*S*,κ*Cl*-Li_2

Figure S45. Li dimers (Part I).

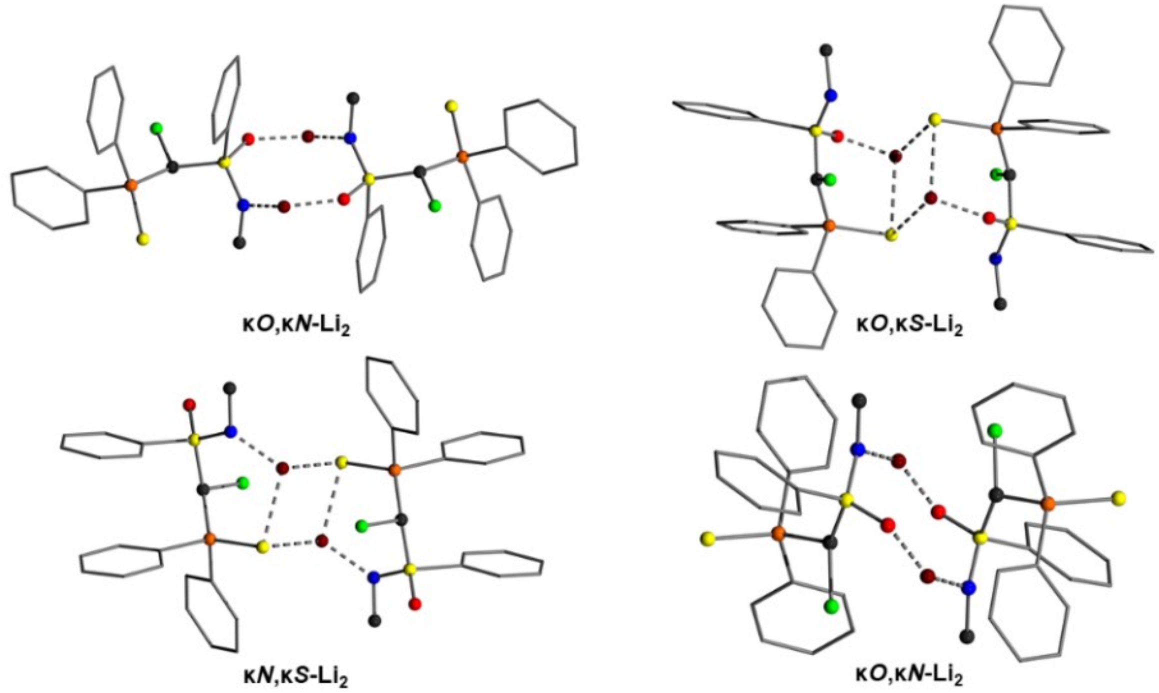

Figure S46. Li dimers (Part II).

6.3 Energies of the optimized compounds

Table S30. SCF energies and enthalpy/free energy corrections in Hartree for all calculated monomers and dimers.

Structure	Corr(G)	Corr(H)	E_{SCF}(THF)	E_{SCF}(toluene)
κ*O*-Li(THF)$_3$	0.611318	0.740854	–3204.14980656	–3204.14139252
κ*N*-Li(THF)$_3$	0.612755	0.741198	–3204.13862831	–3204.12969871
κ*O*,κ*N*-Li(THF)$_3$	0.499075	0.614954	–2971.84616503	–2971.83624384
κ*O*,κ*Cl*-Li(THF)$_3$	0.498869	0.615170	–2971.84374276	–2971.83278651
κ*O*-Li(THF)$_2$	0.499311	0.614947	–2971.85127750	–2971.83885075
κ*N*,κ*S*-Li(THF)$_2$	0.498936	0.615071	–2971.85326828	–2971.84694075
κ*O*,κ*S*-Li(THF)$_2$	0.498165	0.614769	–2971.84916164	–2971.84301703
κ*O*,κ*N*-Li(THF)	0.389890	0.489412	–2739.54907333	–2739.53598398
κ*O*,κ*S*,κ*Cl*-Li(THF)	0.388617	0.488997	–2739.55158286	–2739.54181955
κ*N*,κ*S*,κ*Cl*-Li(THF)	0.390717	0.489508	–2739.55833053	–2739.54956014
κ*N*,κ*S*-Li(THF)	0.390951	0.488434	–2739.55620864	–2739.54573801
κ*O*,κ*N*,κ*S*-Li(THF)	0.386978	0.489206	–2739.55054668	–2739.54303523
κ*O*,κ*S*-Li(THF)	0.386894	0.488934	–2739.55316844	–2739.54272824
κ*O*,κ*S*-Li$_2$(THF)$_2$	0.799234	0.981443	–5479.14610678	–5479.13728744
κ*N*,κ*S*-Li$_2$(THF)$_2$	0.805385	0.981212	–5479.14937283	–5479.13867604
κ*O*,κ*N*,κ*Cl*-Li$_2$(THF)$_2$	0.808277	0.982759	–5479.15020428	–5479.13550119
κ*O*,κ*N*-Li$_2$(THF)$_4$	1.026769	1.233798	–5943.74225062	–5943.73226938
κ*O*,κ*N*,κ*S*,κ*Cl*-Li$_2$	0.585839	0.730979	–5014.57443118	–5014.56302866
κ*N*,κ*S*,κ*Cl*-Li$_2$	0.588293	0.730560	–5014.55088939	–5014.53312623
κ*O*,κ*S*,κ*Cl*-Li$_2$	0.582736	0.729228	–5014.54493149	–5014.52972574
κ*O*,κ*N*-Li$_2$	0.584598	0.730825	–5014.54769161	–5014.52064536
κ*O*,κ*S*-Li$_2$	0.582668	0.728486	–5014.54537659	–5014.52702488
κ*S*,κ*Cl*-Li$_2$	0.588351	0.730557	–5014.55089132	–5014.53314863
κ*O*,κ*N*,κ*Cl*-Li$_2$	0.585758	0.730791	–5014.55606304	–5014.54056817
κ*O*-Na(THF)$_3$	0.605654	0.739556	–3358.82170887	–3358.81141961
κ*S*-Na(THF)$_5$	0.826338	0.991020	–3823.38603574	–3823.37738340
κ*O*-Na(THF)$_4$	0.716097	0.864732	–3591.10906578	–3591.09872543
κ*S*,κ*Cl*-Na(THF)$_4$	0.714107	0.864689	–3591.10150089	–3591.09358604
κ*O*,κ*N*-Na(THF)$_3$	0.606526	0.739755	–3358.81612601	–3358.80652647
κ*N*-Na(THF)$_3$	0.605838	0.739727	–3358.81680171	–3358.80579866
κ*S*,κ*Cl*-Na(THF)$_3$	0.602772	0.739384	–3358.81120355	–3358.80343449
κ*O*-Na(THF)$_2$	0.494900	0.613818	–3126.52827985	–3126.51409305
κ*S*,κ*Cl*-Na(THF)$_2$	0.494753	0.613917	–3126.52185991	–3126.50839103
κ*O*,κ*S*-Na(THF)$_2$	0.494581	0.613737	–3126.52723608	–3126.51962030
κ*O*,κ*N*-Na(THF)	0.383998	0.488412	–2894.22919197	–2894.21429946
κ*S*,κ*Cl*-Na$_2$(THF)$_2$	0.801258	0.979786	–5788.49203279	–5788.47734246
κ*N*,κ*S*,κ*Cl*-Na$_2$(THF)$_2$	0.800781	0.980535	–5788.48094950	–5788.49508176
κ*O*,κ*N*,κ*S*-Na$_2$(THF)$_2$	0.797536	0.979414	–5788.51446562	–5788.50544530
κ*O*,κ*S*,κ*Cl*-Na$_2$(THF)$_2$	0.797808	0.979125	–5788.50372250	–5788.49038243

**κ*O*,κ*N*-Na_2(THF)$_4$**	1.014671	1.231263	–6253.09435252	–6253.08172105
**κ*O*-K(THF)$_3$**	0.601677	0.739037	–3224.93559097	–3224.92429573
**κ*O*-K(THF)$_4$**	0.711753	0.864608	–3457.23124949	–3457.22389523
**κ*Ph*,κ*S*-K(THF)$_3$**	0.599953	0.739010	–3224.93366894	–3224.92210206
**κ*N*-K(THF)$_3$**	0.602761	0.739361	–3224.93163716	–3224.92117272
**κ*O*,κ*S*-K(THF)$_3$**	0.602122	0.738816	–3224.93442813	–3224.92736894
**κ*O*,κ*N*-K(THF)$_2$**	0.492052	0.613403	–2992.64304011	–2992.62906974
**κ*O*-K(THF)$_2$**	0.493058	0.613530	–2992.64651467	–2992.63311645
**κ*O*,κ*Cl*-K(THF)$_2$**	0.492265	0.613605	–2992.63903055	–2992.62436546
**κ*O*,κ*S*-K(THF)$_2$**	0.492313	0.613500	–2992.64457655	–2992.63653412
**κ*O*,κ*N*-K(THF)**	0.383678	0.488079	–2760.34854473	–2760.33396325
**κ*O*,κ*N*,κ*S*-K_2(THF)$_2$**	0.788897	0.978847	–5520.75133972	–5520.74234731
**κ*N*,κ*S*,κ*Cl*-K_2(THF)$_2$**	0.791505	0.978947	–5520.74331077	–5520.73126335
**κ*Ph*,κ*S*,κ*Cl*-K_2(THF)$_2$**	0.788904	0.978682	–5520.73154420	–5520.71673969
**κ*O*,κ*N*,κ*S*-K_2(THF)$_4$**	1.012345	1.228837	–5985.32646643	–5985.31777517

Table S31. Gibbs free energies ΔG_M (kJ/mol), enthalpies ΔH(kJ/mol) and entropies ΔS_M($JK^{-1}mol^{-1}$) relative to κ*O*-M(THF)$_3$ (M=Li,Na,K), respectively. ΔG_M and ΔS_M are the solution state correct energy and entropy.

	**THF**			**Toluol**		
**Structure**	**ΔG_M**	**ΔH**	**ΔS_M**	**ΔG_M**	**ΔH**	**ΔS_M**
**κ*O*-Li(THF)$_3$**						
**κ*N*-Li(THF)$_3$**	33.1	30.3	–9.6	34.5	28.5	–20.1
**κ*O*,κ*N*-Li(THF)$_3$**	44.1	75.9	106.8	34.5	76.8	141.9
**κ*O*,κ*Cl*-Li(THF)$_3$**	49.9	82.9	110.5	43.0	86.4	145.6
**κ*O*-Li(THF)$_2$**	31.3	62.5	104.6	28.2	69.9	139.8
**κ*N*,κ*S*-Li(THF)$_2$**	25.1	57.6	109.0	6.0	49.0	144.2
**κ*O*,κ*S*-Li(THF)$_2$**	33.8	67.6	113.2	14.3	58.5	148.3
**κ*O*,κ*N*-Li(THF)**	79.0	135.6	189.8	64.1	144.8	270.5
**κO,κS,κCl-Li(THF)**	69.1	127.9	197.3	46.0	–31.5	–260.1
**κN,κS,κCl-Li(THF)**	56.9	111.5	183.3	31.2	–50.5	–274.1
**κN,κS-Li(THF)**	63.1	114.3	171.8	41.9	-43.3	-285.6
**κO,κN,κS-Li(THF)**	67.5	131.2	193.5	50.7	-28.0	-263.9
**κO,κS-Li(THF)**	60.4	123.6	213.6	38.6	-34.2	-243.9
**κ*O*,κ*S*-Li_2(THF)$_2$**	81.0	152.2	238.8	6.8	132.3	420.9
**κ*N*,κ*S*-Li_2(THF)$_2$**	88.5	143.0	182.6	19.3	128.0	364.7
**κ*O*,κ*N*,κ*Cl*-Li_2(THF)$_2$**	94.0	144.9	170.7	51.4	31.3	–67.4
**κ*O*,κ*N*-Li_2(THF)$_4$**	30.0	31.0	3.2	–14.5	13.6	94.3
**κ*O*,κ*N*,κ*S*,κ*Cl*-Li_2**	104.8	214.0	366.4	10.8	201.5	202.3
**κ*N*,κ*S*,κ*Cl*-Li_2**	173.0	274.7	341.1	95.7	278.9	279.7
**κ*O*,κ*S*,κ*Cl*-Li_2**	174.1	286.9	378.3	90.1	284.3	285.1
**κ*O*,κ*N*-Li_2**	171.7	283.8	376.0	118.8	312.4	313.2
**κ*O*,κ*S*-Li_2**	172.7	283.8	372.4	97.0	289.5	290.3
**κ*S*,κ*Cl*-Li_2**	173.2	274.7	340.6	95.8	278.8	279.7

**κ*O*,κ*N*,κ*Cl*-Li$_2$**	152.8	261.8	365.5	69.6	260.0	260.8
**κ*O*-Na(THF)$_3$**						
**κ*S*-Na(THF)$_5$**	14.6	–40.0	–183.0	37.5	–44.3	–274.2
**κ*O*-Na(THF)$_4$**	–6.1	–35.1	–97.3	7.7	–34.9	–142.9
**κ*S*,κ*Cl*-Na(THF)$_4$**	8.6	–15.3	–80.2	15.9	–21.6	–125.7
**κ*O*,κ*N*-Na(THF)$_3$**	16.9	15.2	–5.9	15.1	13.4	–5.9
**κ*N*-Na(THF)$_3$**	13.4	13.3	–0.1	15.2	15.2	–0.1
**κ*S*,κ*Cl*-Na(THF)$_3$**	20.0	27.1	23.9	13.4	20.5	23.9
**κ*O*-Na(THF)$_2$**	21.2	49.5	95.1	17.8	59.8	140.7
**κ*S*,κ*Cl*-Na(THF)$_2$**	37.7	66.7	97.3	32.4	75.0	142.8
**κ*O*,κ*S*-Na(THF)$_2$**	23.1	52.1	97.2	2.5	45.0	142.8
**κ*O*,κ*N*-Na(THF)**	56.8	114.8	194.4	41.3	126.9	287.2
**κ*S*,κ*Cl*-Na$_2$(THF)$_2$**	110.5	149.0	129.4	41.8	134.7	311.6
**κ*N*,κ*S*,κ*Cl*-Na$_2$(THF)$_2$**	138.3	180.1	140.2	–6.0	90.1	322.4
**κ*O*,κ*N*,κ*S*-Na$_2$(THF)$_2$**	41.8	89.2	158.9	–41.7	60.0	341.1
**κ*O*,κ*S*,κ*Cl*-Na$_2$(THF)$_2$**	70.7	116.6	154.0	–1.5	98.8	336.2
**κ*O*,κ*N*-Na$_2$(THF)$_4$**	6.2	9.4	10.6	–41.2	–10.9	101.6
**κ*O*-K(THF)$_3$**						
**κ*O*-K(THF)$_4$**	–28.8	–55.8	–90.6	–25.6	–66.2	–136.2
**κ*Ph*,κ*S*-K(THF)$_3$**	0.5	5.0	14.9	1.2	5.7	14.9
**κ*N*-K(THF)$_3$**	13.2	11.2	–6.7	11.0	9.1	–6.7
**κ*O*,κ*S*-K(THF)$_3$**	4.2	2.5	–5.9	–6.9	–8.6	–5.9
**κ*O*,κ*N*-K(THF)$_2$**	21.8	47.5	86.1	15.3	54.5	131.6
**κ*O*-K(THF)$_2$**	15.4	38.7	78.3	7.3	44.2	123.9
**κ*O*,κ*Cl*-K(THF)$_2$**	32.9	58.6	86.0	28.2	67.4	131.5
**κ*O*,κ*S*-K(THF)$_2$**	18.5	43.7	84.6	–3.6	35.2	130.2
**κ*O*,κ*N*-K(THF)**	52.1	100.9	163.8	33.0	109.6	256.7
**κ*O*,κ*N*,κ*S*-K$_2$(THF)$_2$**	16.1	66.5	169.1	–72.8	31.9	351.3
**κ*N*,κ*S*,κ*Cl*-K$_2$(THF)$_2$**	44.0	87.8	147.0	–36.8	61.3	329.2
**κ*Ph*,κ*S*,κ*Cl*-K$_2$(THF)$_2$**	68.1	118.0	167.6	–5.5	98.7	349.8
**κ*O*,κ*N*,κ*S*-K$_2$(THF)$_4$**	9.6	–5.7	–51.2	–53.5	–41.6	39.9

6.4 Coordinates

6.4.1 Coordinates of the monomeric structures of 1-Li

κ*O*-Li(THF)$_3$

E = –3204.14980656

Li 2.311501 0.881552 -0.058066
O 0.598002 0.161150 0.234416
O 2.545079 2.293360 1.243068
O 3.798702 -0.327115 0.027859
O 2.317650 1.726652 -1.785287
S 0.215719 -1.110369 0.954055
C 1.453183 3.214598 1.333621
C 3.002354 1.924570 2.547068
C 4.512301 -0.675742 -1.161407
C 4.110369 -1.232665 1.090690
C 2.787309 3.051537 -1.995738
C 1.302175 1.412646 -2.748936
N 0.725359 -1.389494 2.382955
C -1.478976 -1.158993 0.800520
C 0.972891 -2.436505 0.030056
H 0.654179 2.884636 0.654423
H 1.801085 4.213653 1.016490
C 1.036127 3.204695 2.793115
C 2.357421 2.918476 3.497710
H 4.102209 1.949877 2.555731
H 2.674379 0.892488 2.762949
H 5.020362 0.219535 -1.550618
H 3.787496 -1.018945 -1.918339
C 5.466341 -1.788942 -0.755522
C 4.722185 -2.439237 0.406015
H 3.183220 -1.448596 1.643312
H 4.830901 -0.750038 1.776115
H 3.627849 3.046465 -2.715150
H 3.153186 3.437148 -1.033814
C 1.585397 3.783840 -2.559282
C 0.991732 2.720257 -3.480345
H 0.435082 1.004536 -2.208211
H 1.678911 0.633139 -3.431599
C 0.065057 -0.732938 3.489040
Cl -2.156707 -2.696707 1.334819
P -2.260832 -0.346310 -0.560390
C 1.237611 -2.233758 -1.323785
C 1.182279 -3.677164 0.632207
H 0.319609 2.387954 2.971290
H 0.563283 4.146212 3.103928
H 2.963715 3.835579 3.567810
H 2.236555 2.511569 4.511474
H 6.425126 -1.369641 -0.410918
H 5.676543 -2.479541 -1.584206
H 3.927510 -3.102820 0.032731
H 5.372113 -3.020948 1.074449
H 1.852114 4.714210 -3.079934
H 0.882302 4.029089 -1.747201
H 1.491020 2.746440 -4.461097
H -0.085560 2.850616 -3.652001
H -1.004305 -0.527065 3.296940
H 0.545206 0.224950 3.761852
H 0.123463 -1.383383 4.375754
S -1.813923 -0.818767 -2.442951

C -4.046699 -0.616239 -0.232692
C -2.084599 1.457457 -0.274781
H 1.026872 -1.264883 -1.775579
C 1.748375 -3.287642 -2.078812
C 1.687767 -4.725320 -0.134525
H 0.963935 -3.794430 1.693914
C -4.569861 -0.440620 1.054997
C -4.898287 -0.951713 -1.285409
C -2.061517 2.323491 -1.369282
C -2.075093 1.979753 1.022682
H 1.965703 -3.136153 -3.138691
C 1.973175 -4.531397 -1.487262
H 1.862526 -5.699156 0.328665
H -3.903688 -0.195570 1.884789
C -5.934875 -0.595317 1.279095
C -6.266228 -1.107263 -1.058166
H -4.466834 -1.090541 -2.281026
H -2.069354 1.893790 -2.375009
C -2.024375 3.703771 -1.170782
C -2.055293 3.358826 1.218741
H -2.064595 1.295370 1.873790
H 2.371625 -5.355290 -2.084025
H -6.338084 -0.460464 2.285726
C -6.785911 -0.927571 0.222443
H -6.927560 -1.371642 -1.886965
H -2.000364 4.375736 -2.032389
C -2.026681 4.223965 0.123408
H -2.050346 3.761724 2.234244
H -7.857050 -1.049713 0.401531
H -2.003980 5.305207 0.280100

κ*N*-Li(THF)$_3$

E = –3204.13862831

O 1.885583 1.907612 1.894087
O 3.947915 -0.251153 0.532550
O 3.111294 2.319444 -0.820445
S 0.296341 -1.094494 0.244675
C 0.545092 2.378231 1.732331
C 2.098632 1.468357 3.238989
C 4.881907 -0.457615 -0.528472
C 4.417989 -0.840346 1.744793
C 3.063593 3.677265 -0.406414
C 3.001527 2.358695 -2.234075
C -1.381731 -1.103979 0.485770
C 0.794855 -2.709861 -0.379321
H 0.168605 2.007821 0.768670
H 0.546519 3.484093 1.713362
C -0.216518 1.839301 2.927786
C 0.855056 1.882235 4.012065
H 3.024785 1.926688 3.619322
H 2.212470 0.374155 3.230822
H 5.049562 0.501632 -1.039295
H 4.444116 -1.168574 -1.251619
C 6.129220 -1.024485 0.131094
C 5.542583 -1.770761 1.324697
H 3.570410 -1.344335 2.232031
H 4.785038 -0.045319 2.417861
H 4.046161 4.147124 -0.598005
H 2.866301 3.690514 0.672221
C 1.972922 4.313386 -1.270760
C 1.923471 3.404614 -2.515989

H 2.763021 1.349067 -2.590894
H 3.973785 2.661981 -2.665667
Cl -1.878235 -2.474959 1.496454
P -2.624817 -0.560259 -0.644494
C -0.065549 -3.458262 -1.178378
C 2.100054 -3.129552 -0.124046
H -0.518712 0.800960 2.723294
H -1.109019 2.434356 3.168012
H 0.962917 2.905600 4.406368
H 0.650103 1.212017 4.858593
H 6.785591 -0.209659 0.475772
H 6.709959 -1.664466 -0.547451
H 5.139453 -2.746693 1.008894
H 6.265647 -1.949871 2.132311
H 2.208256 5.359254 -1.512562
H 1.005242 4.301151 -0.749115
H 2.118518 3.945870 -3.452204
H 0.935324 2.930611 -2.603833
S -3.085016 -1.674142 -2.234173
C -4.089394 -0.339132 0.435700
C -2.240740 1.159850 -1.153856
H -1.086220 -3.108652 -1.370814
C 0.395904 -4.654484 -1.727928
C 2.551885 -4.323499 -0.680834
H 2.743476 -2.513671 0.503757
C -3.948311 0.036767 1.777342
C -5.365231 -0.481648 -0.114389
C -2.133922 1.477577 -2.508698
C -2.161051 2.177768 -0.197291
H -0.271615 -5.252797 -2.352559
C 1.699249 -5.086486 -1.481662
H 3.572136 -4.662976 -0.485742
H -2.949043 0.118080 2.211209
C -5.078775 0.280185 2.553842
C -6.494563 -0.235852 0.665650
H -5.456880 -0.795806 -1.157707
H -2.240627 0.676995 -3.245420
C -1.909130 2.796280 -2.903486
C -1.946312 3.495166 -0.592780
H -2.273551 1.940114 0.861342
H 2.053620 -6.025038 -1.914635
H -4.964417 0.567748 3.601808
C -6.352757 0.147835 1.998842
H -7.490144 -0.348535 0.229677
H -1.818074 3.036038 -3.965624
C -1.811249 3.806000 -1.947116
H -1.885130 4.284302 0.160628
H -7.237906 0.337122 2.611136
H -1.639263 4.839746 -2.256673
N 0.763633 0.006132 -0.767972
C 0.623553 -0.267452 -2.188280
H -0.314801 -0.781358 -2.459723
H 0.617574 0.689155 -2.728573
H 1.458350 -0.877692 -2.583038
O 1.017725 -0.937775 1.532809
Li 2.346803 0.835029 0.292261

κ*O*,κ*N*-Li(THF)$_3$

E = –2971.84616503

Li -2.409307 1.054184 -0.609377
O -0.847967 0.308114 0.474695

O -2.160148 2.924890 -0.555529
O -4.000417 0.193637 -0.016215
S -0.588937 -0.681153 -0.631807
C -2.609623 3.923958 -1.471945
C -1.607116 3.525230 0.625320
C -4.238041 -0.015977 1.380748
C -4.705427 -0.771608 -0.802668
N -1.308783 -0.339799 -1.973812
C 1.084987 -0.886171 -0.733322
C -1.382164 -2.197807 -0.128528
H -3.594600 3.628475 -1.863878
H -1.901285 3.981698 -2.317252
C -2.630530 5.215959 -0.673907
C -1.469531 4.999016 0.290588
H -0.656907 3.024729 0.856948
H -2.306002 3.362715 1.464256
H -4.495497 0.948468 1.844367
H -3.307705 -0.388927 1.840177
C -5.355928 -1.043020 1.452016
C -5.125606 -1.849982 0.178666
H -4.027484 -1.127644 -1.592522
H -5.578640 -0.284251 -1.271482
C -0.592034 0.489029 -2.929998
Cl 1.544388 -2.236856 -1.760394
P 2.088836 -0.453696 0.655872
C -1.428695 -2.480466 1.237044
C -1.830926 -3.110693 -1.081079
H -3.578954 5.309894 -0.121206
H -2.516778 6.104967 -1.309534
H -0.509577 5.184856 -0.215530
H -1.511237 5.637691 1.183597
H -6.339333 -0.547216 1.423332
H -5.306584 -1.648345 2.367797
H -4.305739 -2.569059 0.323750
H -6.013345 -2.403268 -0.157749
H 0.431452 0.123338 -3.122611
H -0.513564 1.551802 -2.630554
H -1.140894 0.459311 -3.883044
S 1.826063 -1.324234 2.422890
C 3.790997 -0.730895 0.029965
C 1.995472 1.374455 0.781817
H -1.027074 -1.757629 1.949727
C -1.964500 -3.697212 1.652840
C -2.361920 -4.325835 -0.651458
H -1.773352 -2.847576 -2.137970
C 4.168753 -0.283816 -1.242882
C 4.731101 -1.353005 0.851995
C 2.129223 1.984485 2.029715
C 1.876490 2.163792 -0.367769
H -2.013726 -3.929912 2.718980
C -2.430978 -4.617427 0.711843
H -2.725666 -5.048260 -1.385688
H 3.432990 0.188872 -1.896487
C 5.478747 -0.455939 -1.680882
C 6.043434 -1.524681 0.410465
H 4.413132 -1.699205 1.839661
H 2.204413 1.350579 2.917652
C 2.155982 3.375949 2.128965
C 1.915708 3.552303 -0.267423
H 1.734854 1.681321 -1.336910
H -2.849802 -5.570297 1.043826
H 5.768044 -0.108254 -2.675556

C 6.418789 -1.075585 -0.854361
H 6.774638 -2.013184 1.059122
H 2.257314 3.848101 3.109232
C 2.059335 4.161440 0.980855
H 1.825830 4.163467 -1.169054
H 7.446329 -1.210299 -1.201193
H 2.085245 5.251154 1.058586

κ*O*,κ*Cl*-Li(THF)$_3$

E = –2971.84374276

Li -1.874214 1.763449 0.732799
O -0.602426 3.155749 1.090779
O -3.603034 1.800325 -0.125053
S -0.724469 -0.940789 1.082538
C 0.132798 3.117973 2.317927
C 0.114921 3.892591 0.092643
C -4.772024 2.100046 0.640456
C -3.958721 1.162071 -1.360435
C 0.303520 -0.356285 -0.126227
C -2.103065 -1.833743 0.339859
H 0.046232 2.103295 2.733383
H -0.314561 3.833560 3.030404
C 1.546464 3.520985 1.943632
C 1.297354 4.517380 0.816507
H -0.562475 4.633884 -0.357881
H 0.438035 3.189699 -0.692988
H -5.006372 3.175041 0.544198
H -4.562562 1.883091 1.698816
C -5.867837 1.237396 0.044044
C -5.476672 1.222743 -1.429699
H -3.588085 0.124859 -1.336081
H -3.462661 1.689565 -2.188826
Cl -0.565564 0.633736 -1.298775
P 1.784185 -1.168191 -0.635841
C -1.830689 -2.708490 -0.710816
C -3.390973 -1.692419 0.854417
H 2.104445 2.647891 1.572032
H 2.104239 3.943819 2.790411
H 1.024550 5.501291 1.229638
H 2.163908 4.656529 0.155013
H -6.872259 1.642091 0.229565
H -5.822998 0.220457 0.463047
H -5.806842 2.150894 -1.922297
H -5.899191 0.374303 -1.985485
S 1.745525 -2.988033 -1.440074
C 2.453113 0.054483 -1.831918
C 2.980531 -1.072076 0.743962
H -0.804482 -2.810420 -1.085096
C -2.879518 -3.432825 -1.273097
C -4.430508 -2.432867 0.291998
H -3.568498 -0.996449 1.675779
C 2.820144 1.334352 -1.399416
C 2.579912 -0.285006 -3.178428
C 4.137500 -1.853233 0.682246
C 2.797829 -0.184336 1.807867
H -2.681530 -4.110536 -2.106918
C -4.176889 -3.296186 -0.774151
H -5.444396 -2.333582 0.687361
H 2.728223 1.600068 -0.344242
C 3.303887 2.267372 -2.312308
C 3.062967 0.651822 -4.092771

H 2.299097 -1.294753 -3.491122
H 4.257350 -2.562645 -0.140873
C 5.114346 -1.732834 1.669949
C 3.775014 -0.065878 2.793523
H 1.870820 0.388199 1.862702
H -4.994991 -3.868368 -1.218135
H 3.592210 3.264255 -1.969623
C 3.423004 1.927813 -3.662044
H 3.159569 0.381521 -5.147155
H 6.017825 -2.345187 1.616824
C 4.936498 -0.836771 2.724284
H 3.624263 0.626435 3.625672
H 3.803115 2.660624 -4.378071
H 5.701525 -0.744840 3.499269
N 0.155610 -1.874282 1.922111
C -0.468413 -2.690276 2.936794
H -1.275971 -3.341854 2.547391
H 0.300155 -3.351217 3.363871
H -0.892915 -2.102483 3.772507
O -1.441221 0.219962 1.741565

κ*O*-Li(THF)$_2$

E = –2971.85127750

O 0.742740 0.128423 0.777312
O 2.098612 2.715756 -0.097633
O 3.817967 0.444926 1.165445
S 0.374432 -1.332132 0.868066
C 2.403851 4.090284 0.139986
C 1.636945 2.521153 -1.441065
C 4.661131 0.625591 0.023888
C 4.067896 -0.826497 1.781732
N 0.709698 -2.144606 2.135029
C -1.271935 -1.336077 0.453678
C 1.384660 -2.138382 -0.361414
H 3.364999 4.153910 0.673208
H 1.617532 4.528205 0.780068
C 2.425580 4.740008 -1.233043
C 1.381464 3.916781 -1.979729
H 0.744235 1.878200 -1.424324
H 2.429826 2.010319 -2.016520
H 5.094707 1.636382 0.058053
H 4.049873 0.539977 -0.891077
C 5.691184 -0.488160 0.100286
C 4.895156 -1.601478 0.772266
H 3.101923 -1.300524 2.016432
H 4.625554 -0.662028 2.720786
C -0.122455 -1.930176 3.300135
Cl -1.919212 -2.959230 0.236732
P -1.957978 -0.034192 -0.520587
C 1.767410 -1.397572 -1.479555
C 1.718406 -3.485613 -0.232356
H 3.416425 4.618904 -1.699440
H 2.196806 5.814047 -1.193346
H 0.366932 4.249111 -1.710229
H 1.477589 3.968623 -3.072885
H 6.541631 -0.185197 0.731885
H 6.082130 -0.763103 -0.889317
H 4.236754 -2.097959 0.042860
H 5.523764 -2.369108 1.244462
H -1.202802 -2.008234 3.078064
H 0.045481 -0.949682 3.785394

H 0.119641 -2.703499 4.044442
S -1.357220 0.262396 -2.397158
C -3.758025 -0.379098 -0.460371
C -1.809232 1.502959 0.472011
H 1.458018 -0.356501 -1.569356
C 2.530001 -2.013743 -2.469190
C 2.479303 -4.091458 -1.230596
H 1.398399 -4.027152 0.658133
C -4.369304 -0.721117 0.752655
C -4.530968 -0.250876 -1.614537
C -1.745606 2.733086 -0.183773
C -1.802069 1.461985 1.870592
H 2.842479 -1.440629 -3.345194
C 2.889080 -3.356866 -2.344521
H 2.756462 -5.143910 -1.135879
H -3.761510 -0.836858 1.652867
C -5.745133 -0.926213 0.804780
C -5.909829 -0.457489 -1.559671
H -4.031683 0.010253 -2.552112
H -1.745996 2.745294 -1.277360
C -1.659661 3.916142 0.551009
C -1.715686 2.643741 2.602505
H -1.833975 0.495028 2.376997
H 3.488668 -3.835338 -3.122499
H -6.218438 -1.195474 1.752159
C -6.517891 -0.793209 -0.351233
H -6.510515 -0.356611 -2.466956
H -1.606293 4.876109 0.031048
C -1.640708 3.873816 1.944649
H -1.701718 2.605221 3.694711
H -7.597751 -0.955512 -0.307847
H -1.569788 4.799741 2.520835
Li 2.092577 1.301281 1.193620

κ*N*,*κS*-Li(THF)$_2$

E = –2971.85326828

Cl 0.714492 -0.243982 -2.276262
S 0.052810 -1.973623 1.410860
O -2.486229 -2.456635 -1.174136
C 0.758105 0.447578 -0.655495
P 1.320538 -0.623566 0.624610
C -2.486089 -2.017942 -2.538123
C -1.923308 -3.761626 -1.111645
C 2.055031 0.410803 1.935999
C 2.753519 -1.471796 -0.130579
C -0.397539 2.904083 -1.315638
H -3.511153 -2.081125 -2.940499
H -2.165462 -0.966262 -2.531594
C -1.515989 -2.939852 -3.289577
C -0.842792 -3.740631 -2.173757
H -1.544114 -3.917687 -0.092134
H -2.699634 -4.519019 -1.330764
C 2.655341 1.634997 1.621394
C 2.074998 -0.058985 3.250564
C 3.733895 -0.724849 -0.795843
C 2.889659 -2.855529 -0.015797
C 0.110310 4.069338 -0.743816
C -0.743753 2.843996 -2.665949
H -0.793171 -2.370259 -3.889850
H -2.064267 -3.609376 -3.969565
H 0.033743 -3.200292 -1.783341

H -0.525266 -4.744823 -2.488117
H 2.606386 2.008582 0.596365
C 3.277640 2.378737 2.620399
C 2.700737 0.688640 4.247605
H 1.583527 -1.007078 3.484104
H 3.618409 0.356322 -0.899481
C 4.844419 -1.365231 -1.337441
C 4.004547 -3.493771 -0.560408
H 2.109748 -3.420899 0.501729
H 0.355213 4.076954 0.319832
C 0.286058 5.194569 -1.546481
C -0.560895 3.975290 -3.458818
H -1.152305 1.921896 -3.082697
H 3.738739 3.338272 2.373979
C 3.303794 1.906114 3.933912
H 2.712314 0.319068 5.275801
H 5.606683 -0.781151 -1.858500
C 4.981832 -2.750360 -1.219453
H 4.107454 -4.577684 -0.469030
H 0.681935 6.115032 -1.111152
C -0.045728 5.146560 -2.901486
H -0.826531 3.943122 -4.517930
H 3.790411 2.493392 4.716530
H 5.854643 -3.250294 -1.646496
H 0.093766 6.030943 -3.527594
S -0.571687 1.450185 -0.282420
O -0.423564 1.922806 1.118414
N -1.893103 0.712855 -0.679338
C -3.121211 1.487436 -0.639438
H -3.079760 2.400232 -1.262182
H -3.935060 0.862099 -1.036180
H -3.409608 1.798466 0.380917
Li -1.979905 -1.148428 0.162156
O -3.296987 -0.962503 1.557881
C -4.696483 -1.225418 1.496107
C -2.984434 -0.103454 2.660394
C -5.262137 -0.708862 2.809382
H -5.127943 -0.688309 0.632513
H -4.850715 -2.304052 1.341479
C -4.321812 0.452026 3.116844
H -2.499180 -0.700608 3.451619
H -2.268007 0.657953 2.316727
H -5.183867 -1.481326 3.591041
H -6.317582 -0.414083 2.726998
H -4.316624 0.746705 4.175357
H -4.595712 1.336259 2.518872

κ*O*,κ*S*-Li(THF)$_2$

E = –2971.84916164

Cl 0.529746 -0.222006 -2.322205
S 0.635299 -1.470142 1.711411
O -1.481565 -3.144549 -0.714861
C 0.489248 0.647016 -0.784427
P 1.433578 -0.033061 0.545071
C -1.789090 -2.923733 -2.095602
C -0.458860 -4.128338 -0.608311
C 2.051089 1.345298 1.565613
C 2.933272 -0.665389 -0.288925
C -1.697730 2.096926 -1.728632
H -2.783111 -3.344683 -2.320440
H -1.819512 -1.836010 -2.249996

C -0.676324 -3.605675 -2.899454
C 0.387760 -3.912438 -1.846105
H 0.075965 -3.952785 0.335882
H -0.907797 -5.139402 -0.591596
C 2.202100 2.620319 1.011901
C 2.438068 1.104664 2.885548
C 3.675957 0.182844 -1.120142
C 3.347127 -1.982626 -0.089827
C -1.168660 3.382715 -1.835914
C -2.661639 1.626466 -2.615391
H -0.299767 -2.961512 -3.706342
H -1.043667 -4.536869 -3.357288
H 1.041952 -3.040537 -1.693031
H 1.013312 -4.780498 -2.097585
H 1.866420 2.796558 -0.012394
C 2.747634 3.647479 1.777869
C 2.984398 2.136127 3.648396
H 2.294109 0.108516 3.311940
H 3.349656 1.212615 -1.282321
C 4.823842 -0.291827 -1.747552
C 4.498361 -2.455051 -0.721093
H 2.755064 -2.627312 0.565409
H -0.422992 3.719228 -1.111775
C -1.611430 4.210814 -2.863485
C -3.097133 2.463642 -3.642502
H -3.059460 0.618709 -2.487850
H 2.858613 4.645507 1.346851
C 3.141431 3.406926 3.095591
H 3.284882 1.946049 4.681627
H 5.400250 0.370934 -2.397287
C 5.236137 -1.611758 -1.549575
H 4.818438 -3.487765 -0.563285
H -1.207454 5.221069 -2.960275
C -2.572507 3.750095 -3.766735
H -3.850694 2.108580 -4.349260
H 3.566295 4.215557 3.695451
H 6.137505 -1.981736 -2.044294
H -2.916924 4.402305 -4.572751
S -1.112728 1.080565 -0.370247
Li -1.600204 -1.686955 0.554339
O -2.892775 -1.988847 1.980612
C -4.102762 -1.224854 1.922721
C -2.495675 -2.123268 3.340087
C -4.259996 -0.561577 3.298170
H -3.987919 -0.501178 1.102006
H -4.947654 -1.892688 1.686815
C -2.907875 -0.807719 3.967833
H -3.020496 -2.979066 3.804817
H -1.413582 -2.311969 3.360375
H -5.063729 -1.048118 3.871130
H -4.510851 0.505685 3.219651
H -2.966188 -0.849567 5.064391
H -2.178908 -0.030939 3.686831
O -2.091204 -0.075652 -0.381620
N -0.954304 1.884611 0.933211
C -2.164754 2.343563 1.576018
H -1.890440 2.789665 2.543375
H -2.696644 3.124576 0.996693
H -2.891994 1.537438 1.782343

κ*O*,κ*N*-Li(THF)

E = –2739.54907333

Li 2.318590 1.644395 -1.029824
O 0.956659 1.359231 0.426642
O 3.912270 0.834214 -0.379088
S -0.184321 1.589944 -0.515395
C 5.147449 0.561381 -1.037224
C 3.955022 0.385790 0.986279
N 0.476467 1.976479 -1.885710
C -1.217051 0.248218 -0.511091
C -1.117439 2.980983 0.119144
H 5.381555 1.401844 -1.707433
H 5.045116 -0.354957 -1.646196
C 6.152089 0.367876 0.082849
C 5.294113 -0.317917 1.141049
H 3.088663 -0.264447 1.170733
H 3.875624 1.268596 1.641664
C -0.289943 1.844209 -3.106254
Cl -2.839481 0.582550 -1.099312
P -0.999616 -1.042170 0.670643
C -1.887374 2.791874 1.267464
C -1.009068 4.223125 -0.499463
H 6.511655 1.344103 0.445694
H 7.024004 -0.223847 -0.228762
H 5.189371 -1.389247 0.911725
H 5.696837 -0.227080 2.159207
H -1.103670 2.587937 -3.200303
H -0.745107 0.842271 -3.216953
H 0.391230 1.996159 -3.956248
S -1.320503 -0.721030 2.606929
C -2.113133 -2.349887 0.028076
C 0.666766 -1.741824 0.366023
H -1.951755 1.798051 1.726277
C -2.560097 3.884761 1.805824
C -1.694413 5.308661 0.046323
H -0.390419 4.325388 -1.393685
C -2.063395 -2.713998 -1.323335
C -2.988070 -3.006037 0.893394
C 1.289040 -2.469973 1.383593
C 1.281937 -1.633898 -0.885931
H -3.165735 3.757777 2.706089
C -2.465588 5.138702 1.195918
H -1.624536 6.290109 -0.428194
H -1.387304 -2.189754 -2.002685
C -2.882577 -3.733816 -1.798654
C -3.809767 -4.026749 0.413710
H -3.011405 -2.701108 1.943574
H 0.807117 -2.524562 2.363523
C 2.508058 -3.103184 1.144955
C 2.505575 -2.258654 -1.117760
H 0.801156 -1.041585 -1.668148
H -3.001632 5.990652 1.620990
H -2.842827 -4.015508 -2.853649
C -3.756902 -4.391970 -0.930276
H -4.494651 -4.537665 1.094848
H 2.988071 -3.674653 1.942995
C 3.115399 -3.002790 -0.107286
H 2.985056 -2.165495 -2.095316
H -4.400252 -5.191430 -1.306077
H 4.070181 -3.500357 -0.294537

κ*O*,κ*S*,κ*Cl*-Li(THF)

E = -2739.55158286

C -0.176335 -0.365862 0.932914
Cl -1.807319 -0.411863 1.627372
S 0.376102 -1.968441 0.650273
P 0.079935 0.834852 -0.342257
S -0.348511 0.387553 -2.265878
O -0.328094 -2.678193 -0.492012
N 0.352351 -2.691909 2.010128
C 0.644272 -4.111721 1.962273
H 1.690240 -4.336822 1.670192
H 0.496883 -4.525497 2.971045
H -0.014421 -4.673551 1.274769
C 2.045098 -1.749244 0.029287
C 3.034480 -1.428466 0.957490
C 2.340936 -1.942847 -1.317689
C 4.343472 -1.261825 0.517423
H 2.763374 -1.305229 2.007968
C 3.656252 -1.778840 -1.746785
H 1.544060 -2.202309 -2.013593
C 4.651467 -1.431692 -0.833600
H 5.126529 -0.993421 1.229620
H 3.903591 -1.916941 -2.801702
H 5.679509 -1.293968 -1.176815
C 1.808910 1.419236 -0.211361
C 2.567941 1.655197 -1.357614
C 2.343862 1.706208 1.050694
C 3.854137 2.183548 -1.244420
H 2.141011 1.413502 -2.334322
C 3.622930 2.242338 1.158784
H 1.763476 1.481801 1.947459
C 4.379725 2.483689 0.010586
H 4.447773 2.359581 -2.144599
H 4.036514 2.462856 2.145690
H 5.386491 2.899689 0.097073
C -0.906368 2.278498 0.196798
C -0.900204 2.695415 1.534624
C -1.629546 3.002527 -0.752363
C -1.610533 3.831428 1.910536
H -0.358332 2.119781 2.287005
C -2.340248 4.140937 -0.370882
H -1.629660 2.658320 -1.790067
C -2.330147 4.557160 0.958741
H -1.607393 4.149513 2.955674
H -2.904499 4.702998 -1.118802
H -2.887518 5.447970 1.258378
Li -1.419673 -1.779552 -1.713285
O -3.272972 -1.576729 -1.317547
C -3.951943 -0.320052 -1.401925
C -3.915836 -2.436378 -0.367968
C -5.325761 -0.581392 -0.815315
H -3.395303 0.431394 -0.816423
H -3.965559 -0.002009 -2.454338
C -5.002440 -1.592209 0.280061
H -4.335353 -3.301880 -0.908605
H -3.160401 -2.794651 0.347394
H -5.990005 -1.027628 -1.572906
H -5.802494 0.334399 -0.438921
H -5.867067 -2.192177 0.595899
H -4.601125 -1.075152 1.164828

κ*N*,κ*S*,κ*Cl*-Li(THF)

E = -2739.55833053

C -0.136017 -0.323295 0.926378
Cl -1.678349 -0.376873 1.795746
S 0.518948 -1.909638 0.838663
P -0.039458 0.839505 -0.395810
S -0.631349 0.333299 -2.260926
C 2.238075 -1.680193 0.399818
C 3.192070 -1.714505 1.414790
C 2.589041 -1.473073 -0.932357
C 4.529760 -1.514370 1.086096
H 2.871153 -1.896390 2.441750
C 3.929894 -1.262751 -1.247482
H 1.814678 -1.452791 -1.703117
C 4.895328 -1.280695 -0.241176
H 5.291266 -1.536192 1.868980
H 4.220537 -1.079858 -2.284121
H 5.945018 -1.113218 -0.493537
C 1.669122 1.482604 -0.466148
C 2.166205 1.983869 -1.672358
C 2.458046 1.528702 0.687952
C 3.448006 2.526670 -1.724152
H 1.545627 1.925622 -2.570001
C 3.742230 2.064306 0.628563
H 2.069513 1.112346 1.619635
C 4.238128 2.565085 -0.575247
H 3.834529 2.914882 -2.669517
H 4.362676 2.081586 1.527505
H 5.247469 2.981418 -0.619284
C -1.028213 2.253721 0.205124
C -0.737692 2.826456 1.449745
C -2.055511 2.775370 -0.581092
C -1.478105 3.914940 1.900231
H 0.060065 2.413538 2.070887
C -2.797023 3.865426 -0.124023
H -2.262688 2.317823 -1.552186
C -2.509429 4.435140 1.114615
H -1.251771 4.359253 2.872258
H -3.602228 4.270367 -0.741567
H -3.089640 5.289253 1.472117
Li -1.427501 -1.861803 -1.550193
O -3.329085 -1.991548 -1.300185
C -4.187563 -0.928795 -1.703955
C -3.808391 -2.542490 -0.065957
C -4.633786 -0.304076 -0.397840
H -3.606121 -0.255823 -2.350155
H -5.042180 -1.333204 -2.277828
C -4.822670 -1.535310 0.489466
H -4.264214 -3.527258 -0.260061
H -2.935492 -2.678028 0.588763
H -5.544458 0.302553 -0.500345
H -3.828341 0.335279 -0.005056
H -5.847038 -1.925979 0.393888
H -4.644257 -1.319418 1.551968
N -0.187124 -2.721199 -0.309616
O 0.513941 -2.545583 2.173565
C 0.219991 -4.114545 -0.409025
H 1.293648 -4.237457 -0.651967
H 0.021772 -4.683201 0.516067
H -0.352762 -4.587733 -1.220322

κ*N*,κ*S*-Li(THF)

E = -2739.55620864

S -0.391256 -2.376835 -1.304385
C -0.577785 0.783527 -0.563470
P -1.239252 -0.831813 -0.330033
C -1.305754 -1.149778 1.464294
C -2.991110 -0.667520 -0.821507
C 1.076268 2.862418 0.282561
C -1.780362 -0.157112 2.329505
C -0.936726 -2.397660 1.965763
C -3.680541 0.530156 -0.596728
C -3.660671 -1.766896 -1.363629
C 0.900115 3.358759 1.572985
C 1.290611 3.710017 -0.804210
H -2.048134 0.825153 1.935000
C -1.881528 -0.418799 3.692113
C -1.038501 -2.654961 3.333164
H -0.563683 -3.157075 1.273378
H -3.144639 1.393449 -0.195529
C -5.035171 0.618821 -0.907644
C -5.017908 -1.674529 -1.669039
H -3.104166 -2.688962 -1.551490
H 0.749803 2.658901 2.396955
C 0.927940 4.736817 1.775226
C 1.314248 5.086791 -0.589389
H 1.439213 3.287972 -1.799588
H -2.246685 0.358244 4.367926
C -1.510766 -1.667862 4.196203
H -0.745278 -3.632082 3.724530
H -5.569676 1.556542 -0.738101
C -5.706083 -0.483426 -1.440164
H -5.538528 -2.536812 -2.092323
H 0.792935 5.140557 2.781318
C 1.131359 5.598663 0.695923
H 1.480756 5.763586 -1.430528
H -1.589246 -1.869290 5.267379
H -6.768816 -0.410336 -1.683735
H 1.152548 6.678759 0.858937
S 0.995692 1.089622 0.031306
O 1.143869 0.482040 1.377085
N 2.042820 0.697912 -1.067335
C 3.426255 1.043190 -0.781434
H 4.038314 0.785673 -1.658253
H 3.572629 2.122550 -0.590114
H 3.841052 0.496817 0.085045
Li 1.678668 -1.104526 -1.899148
O 3.075605 -2.212017 -1.142934
C 4.383376 -2.543502 -1.606441
C 2.958027 -2.451602 0.265936
C 5.050357 -3.274712 -0.452176
H 4.296889 -3.152552 -2.519307
H 4.923352 -1.613733 -1.859854
C 4.384370 -2.623283 0.754924
H 2.423493 -1.604111 0.720989
H 2.358653 -3.364551 0.423404
H 6.144268 -3.170961 -0.464434
H 4.809581 -4.349125 -0.487995
H 4.840272 -1.641960 0.963107
H 4.445049 -3.227090 1.670902
Cl -0.851989 1.429762 -2.186993

κ*O*,κ*N*,κ*S*-Li(THF)

E = -2739.55054668

S -1.185196 -1.539479 -2.073358
C -0.592307 1.051860 -0.164519
P -1.521730 -0.432768 -0.422448
C -1.339086 -1.458015 1.076581
C -3.253112 0.138362 -0.388740
C 1.891418 2.098589 0.531490
C -0.963787 -0.884809 2.295326
C -1.620625 -2.825167 1.005295
C -3.751215 0.763161 0.761348
C -4.075441 -0.034928 -1.502266
C 3.220324 2.387195 0.219172
C 1.235662 2.728607 1.588140
H -0.716315 0.178169 2.327483
C -0.877379 -1.677287 3.437970
C -1.536019 -3.613564 2.151590
H -1.888190 -3.264640 0.040896
H -3.107179 0.902057 1.632937
C -5.069456 1.208554 0.791573
C -5.395971 0.412977 -1.467455
H -3.668314 -0.526212 -2.390004
H 3.698617 1.880055 -0.621650
C 3.906890 3.322015 0.990259
C 1.930544 3.666825 2.347631
H 0.190695 2.484120 1.790084
H -0.574897 -1.228447 4.387172
C -1.165596 -3.040822 3.368810
H -1.755778 -4.682258 2.092332
H -5.457633 1.694739 1.689624
C -5.893010 1.033980 -0.322858
H -6.038474 0.275382 -2.340360
H 4.948281 3.556490 0.759196
C 3.262831 3.959828 2.051595
H 1.428816 4.172559 3.175537
H -1.094500 -3.660921 4.265773
H -6.927854 1.383869 -0.296249
H 3.803129 4.695908 2.651394
S 1.084441 0.823137 -0.422628
O 1.380007 -0.525932 0.145098
N 1.684396 0.798802 -1.868237
C 1.522917 1.917709 -2.773483
H 0.501946 1.995335 -3.184562
H 1.771149 2.892300 -2.312962
H 2.213224 1.772976 -3.618303
Li 1.348252 -1.251179 -1.954384
O 2.829328 -2.448865 -1.669778
C 4.067651 -1.768411 -1.442498
C 2.602051 -3.423601 -0.640809
C 4.370470 -1.987691 0.026825
H 4.848486 -2.205524 -2.090727
H 3.926299 -0.711589 -1.714467
C 3.849080 -3.406344 0.233399
H 1.705479 -3.124788 -0.072950
H 2.413380 -4.401069 -1.110461
H 3.787839 -1.274517 0.629036
H 5.437418 -1.872542 0.263883
H 3.622192 -3.640865 1.282728
H 4.585687 -4.141969 -0.127133
Cl -1.216864 2.550032 -0.867278

κ*O*,κ*S*-Li(THF)

E = -2739.55316844
S -0.664426 -1.806987 -1.873527
C -0.604872 1.052991 -0.307359
P -1.308591 -0.564767 -0.423436
C -1.203672 -1.357310 1.215362
C -3.095280 -0.243043 -0.632377
C 1.448509 2.842897 0.246473
C -1.391963 -0.597738 2.375314
C -1.003699 -2.734860 1.305753
C -3.701026 0.826055 0.039320
C -3.868860 -1.104707 -1.412368
C 1.099571 3.316183 1.510877
C 2.070471 3.657410 -0.694882
H -1.527139 0.482651 2.295637
C -1.373492 -1.219880 3.619514
C -0.983991 -3.354399 2.555895
H -0.853060 -3.309356 0.387963
H -3.087890 1.509578 0.631132
C -5.075044 1.023503 -0.069577
C -5.245282 -0.905321 -1.514816
H -3.378953 -1.924932 -1.943708
H 0.621023 2.641817 2.224881
C 1.376612 4.641586 1.833914
C 2.341741 4.984134 -0.361297
H 2.332238 3.243866 -1.669586
H -1.512532 -0.624892 4.525417
C -1.168263 -2.598776 3.712427
H -0.822574 -4.432962 2.624357
H -5.544586 1.862671 0.449174
C -5.849237 0.156987 -0.843597
H -5.847091 -1.581743 -2.126433
H 1.110865 5.028028 2.820420
C 1.994894 5.474108 0.897766
H 2.825866 5.638526 -1.089873
H -1.150895 -3.083509 4.691693
H -6.927188 0.315266 -0.927622
H 2.209412 6.514252 1.154198
S 1.098957 1.120484 -0.109836
Li 1.640775 -0.846358 -2.159760
O 2.801056 -2.212964 -1.456592
C 4.195642 -2.396669 -1.701006
C 2.375223 -2.989053 -0.326169
C 4.590322 -3.609901 -0.876497
H 4.352414 -2.527813 -2.782320
H 4.743597 -1.493688 -1.377064
C 3.656341 -3.483191 0.322233
H 1.757445 -2.349797 0.321136
H 1.750588 -3.823359 -0.687076
H 5.656132 -3.608795 -0.609019
H 4.378444 -4.536943 -1.432795
H 4.042227 -2.734174 1.031368
H 3.511718 -4.425221 0.869137
Cl -1.066366 2.089958 -1.676285
O 1.876919 0.880477 -1.386716
N 1.411276 0.206686 1.091255
C 2.808181 0.084001 1.451136
H 3.238961 1.024087 1.849728
H 2.890196 -0.671080 2.246990
H 3.453385 -0.244106 0.615193

6.4.2 Coordinates of the dimeric structures of 1-Li

κ*O*,κ*S*-$Li_2(THF)_2$

E = –5479.14610678

Cl -3.881936 1.720549 -1.969288
Cl 3.881650 -1.720805 1.969188
S -0.942092 1.079989 1.388326
S 0.942126 -1.079824 -1.388499
O 0.081845 -2.493836 1.966786
O -0.081620 2.493970 -1.966937
C -3.307647 0.548813 -0.814909
C 3.307619 -0.548892 0.814858
P -2.888523 0.976033 0.826525
P 2.888541 -0.975957 -0.826628
C 0.268267 -2.162981 3.347234
C 0.672659 -3.759718 1.664891
C -0.268588 2.162981 -3.347289
C -0.672074 3.760001 -1.664975
C -3.749149 -0.155515 1.977148
C -3.687952 2.591983 1.108913
C 3.749239 0.155681 -1.977108
C 3.687935 -2.591908 -1.109114
C -4.284062 -1.988981 -1.646962
H -0.642957 -1.659239 3.699048
H 1.116389 -1.462802 3.434845
C 0.532392 -3.494045 4.023858
C 1.334098 -4.228876 2.954129
H 1.389319 -3.632985 0.839080
H -0.124952 -4.445968 1.332673
C 4.284093 1.988802 1.647125
H 0.642519 1.659256 -3.699435
H -1.116703 1.462744 -3.434485
C -0.533056 3.493964 -4.023949
C -1.334216 4.228948 -2.953918
H -1.388238 3.633544 -0.838693
H 0.125825 4.446233 -1.333406
C -4.945865 -0.765804 1.590339
C -3.236393 -0.378557 3.258336
C -5.068632 2.726352 0.912030
C -2.939539 3.681167 1.558060
C 4.945998 0.765837 -1.590222
C 3.236493 0.378928 -3.258263
C 5.068604 -2.726334 -0.912197
C 2.939498 -3.681042 -1.558342
C -4.555145 -3.062034 -0.800664
C -5.149447 -1.651387 -2.690255
H 1.073382 -3.383770 4.974295
H -0.416653 -4.016372 4.227418
H 2.382541 -3.897658 2.976596
H 1.313976 -5.322439 3.060794
C 4.555271 3.061883 0.800895
C 5.149413 1.651107 2.690441
H -1.074528 3.383557 -4.974096
H 0.415881 4.016271 -4.228065
H -2.382660 3.897698 -2.975771
H -1.314170 5.322496 -3.060761
H -5.335525 -0.600745 0.583338
C -5.618333 -1.604591 2.477303
C -3.912495 -1.216200 4.143258
H -2.297920 0.101573 3.546595
H -5.658516 1.877416 0.559639

C -5.688841 3.947217 1.157757
C -3.565054 4.904890 1.799383
H -1.865921 3.556902 1.722222
H 5.335644 0.600630 -0.583239
C 5.618523 1.604691 -2.477080
C 3.912649 1.216642 -4.143078
H 2.297984 -0.101098 -3.546579
H 5.658504 -1.877440 -0.559729
C 5.688782 -3.947201 -1.157988
C 3.564982 -4.904768 -1.799729
H 1.865885 -3.556736 -1.722506
H -3.856777 -3.295760 0.003845
C -5.722204 -3.800467 -0.994789
C -6.314873 -2.391755 -2.870851
H -4.907605 -0.812676 -3.346547
H 3.856955 3.295686 -0.003635
C 5.722360 3.800244 0.995112
C 6.314869 2.391404 2.871128
H 4.907499 0.812372 3.346675
H -6.545361 -2.090513 2.163711
C -5.102183 -1.832847 3.752938
H -3.505068 -1.391740 5.141871
H -6.765395 4.049057 1.002596
C -4.937409 5.038837 1.599384
H -2.974742 5.755205 2.148722
H 6.545587 2.090507 -2.163429
C 5.102384 1.833150 -3.752683
H 3.505227 1.392344 -5.141664
H 6.765328 -4.049085 -1.002803
C 4.937329 -5.038768 -1.599708
H 2.974652 -5.755044 -2.149134
H -5.945332 -4.642757 -0.335555
C -6.602060 -3.464667 -2.023874
H -7.001202 -2.133439 -3.680623
H 5.945563 4.642556 0.335931
C 6.602151 3.464345 2.024220
H 7.001147 2.133010 3.680918
H -5.627287 -2.495611 4.445039
H -5.427359 5.996662 1.790362
H 5.627533 2.495967 -4.444700
H 5.427254 -5.996596 -1.790736
H -7.516176 -4.044687 -2.170910
H 7.516291 4.044309 2.171328
S -2.826126 -0.965096 -1.396834
O -2.159811 -1.635654 -0.217167
S 2.826124 0.964993 1.396871
O 2.159879 1.635662 0.217225
N 2.114294 0.770664 2.740946
C 1.834683 1.919763 3.574156
H 1.657143 1.569021 4.602310
H 2.659838 2.656818 3.617102
H 0.922916 2.452958 3.250327
N -2.114359 -0.770836 -2.740954
C -1.834724 -1.919991 -3.574081
H -2.659888 -2.657035 -3.617020
H -1.657136 -1.569310 -4.602248
H -0.922978 -2.453180 -3.250186
Li 0.506665 1.273983 -0.639876
Li -0.506536 -1.273850 0.639767

κ*N*,κ*S*-$Li_2(THF)_2$

E = –5479.14937283

Cl -2.350006 -0.655674 2.040689
Cl 2.350152 0.655766 -2.040698
S -0.908357 -0.876635 -1.515934
S 0.908327 0.876611 1.515873
O -0.466811 2.603675 -1.952099
O 0.466803 -2.603661 1.952031
C -3.305662 -0.280646 0.598348
C 3.305729 0.280633 -0.598328
P -2.773935 -1.199576 -0.802527
P 2.773939 1.199542 0.802549
C -1.532182 2.525843 -2.903255
C 0.363952 3.733905 -2.210827
C 1.532148 -2.525772 2.903217
C -0.363925 -3.733915 2.210765
C -4.008350 -0.991101 -2.121219
C -2.926283 -2.946357 -0.296180
C 4.008300 0.991080 2.121290
C 2.926316 2.946326 0.296221
H -2.469672 2.317329 -2.365356
H -1.334132 1.684396 -3.589193
C -1.514116 3.858785 -3.631051
C -0.030082 4.208526 -3.598231
H 1.415312 3.426009 -2.122732
H 0.167583 4.509595 -1.450097
H 2.469651 -2.317261 2.365340
H 1.334058 -1.684301 3.589112
C 1.514085 -3.858684 3.631069
C 0.030061 -4.208462 3.598208
H -1.415291 -3.426067 2.122602
H -0.167485 -4.509633 1.450079
C -5.366525 -1.151022 -1.822259
C -3.602917 -0.731561 -3.430101
C -3.871724 -3.329009 0.661846
C -2.121104 -3.910586 -0.909143
C 5.366489 1.150996 1.822391
C 3.602803 0.731609 3.430166
C 3.871793 3.328966 -0.661774
C 2.121144 3.910566 0.909174
H -1.932275 3.790055 -4.644962
H -2.096676 4.609611 -3.072889
H 0.515284 3.638572 -4.367805
H 0.178104 5.276484 -3.753432
H 1.932209 -3.789901 4.644991
H 2.096682 -4.609521 3.072959
H -0.515347 -3.638478 4.367729
H -0.178111 -5.276416 3.753453
H -5.678401 -1.339615 -0.792958
C -6.312732 -1.044539 -2.835894
C -4.556517 -0.621505 -4.442423
H -2.537554 -0.613447 -3.643640
H -4.484777 -2.565521 1.146329
C -3.998273 -4.672102 1.010478
C -2.261172 -5.253958 -0.566175
H -1.375420 -3.596174 -1.644124
H 5.678412 1.339546 0.793097
C 6.312646 1.044560 2.836078
C 4.556354 0.621594 4.442539
H 2.537431 0.613508 3.643661
H 4.484841 2.565466 -1.146247
C 3.998384 4.672059 -1.010388

C 2.261259 5.253940 0.566231
H 1.375432 3.596162 1.644130
H -7.373379 -1.164280 -2.602722
C -5.909393 -0.777571 -4.146697
H -4.238479 -0.412564 -5.466585
H -4.726424 -4.968394 1.769160
C -3.195856 -5.635180 0.396519
H -1.629650 -6.004880 -1.046803
H 7.373304 1.164293 2.602951
C 5.909244 0.777646 4.146872
H 4.238265 0.412701 5.466695
H 4.726562 4.968342 -1.769047
C 3.195978 5.635150 -0.396434
H 1.629745 6.004873 1.046852
H -6.655960 -0.691105 -4.939948
H -3.297929 -6.687369 0.672864
H 6.655773 0.691217 4.940163
H 3.298085 6.687341 -0.672760
S -3.586344 1.400707 0.394219
S 3.586351 -1.400747 -0.394259
N -2.227139 2.165173 0.478595
C -2.289712 3.616221 0.492760
H -1.284952 4.002753 0.721020
H -2.596722 4.046258 -0.478067
H -2.974991 4.015796 1.263479
N 2.227135 -2.165183 -0.478683
C 2.289675 -3.616231 -0.492885
H 2.596658 -4.046302 0.477935
H 1.284912 -4.002737 -0.721176
H 2.974962 -4.015797 -1.263603
Li 0.593480 -1.414846 0.433001
Li -0.593487 1.414827 -0.433105
O -4.443645 1.621411 -0.796605
O 4.443642 -1.621544 0.796555
C -4.583661 1.864138 1.811615
C -3.969167 2.113221 3.038579
C -5.965791 1.949619 1.655916
C -4.763698 2.444000 4.134771
H -2.882730 2.049384 3.121128
C -6.749705 2.281925 2.758623
H -6.404431 1.760143 0.674643
C -6.149834 2.525580 3.995625
H -4.296838 2.641933 5.102355
H -7.834544 2.353969 2.651877
H -6.768191 2.786006 4.857924
C 4.583659 -1.864160 -1.811666
C 3.969148 -2.113250 -3.038619
C 5.965791 -1.949640 -1.655985
C 4.763664 -2.444016 -4.134825
H 2.882707 -2.049432 -3.121148
C 6.749691 -2.281935 -2.758706
H 6.404445 -1.760170 -0.674717
C 6.149804 -2.525585 -3.995701
H 4.296792 -2.641953 -5.102402
H 7.834532 -2.353975 -2.651975
H 6.768150 -2.786004 -4.858010

κ*O*,κ*N*,κ*Cl*-$Li_2(THF)_2$

E = –5479.15020428

Cl -1.444965 1.365199 -2.156734
Cl 1.591586 -1.537747 2.103500

S -5.045054 1.571508 -1.478483
S 5.183609 -1.638022 1.552207
C -2.087768 0.558976 -0.709108
C 2.238303 -0.697821 0.682990
P -3.789405 0.112959 -1.014793
P 3.921227 -0.210208 1.012159
C -4.397566 -0.868480 0.403801
C -3.610909 -1.115151 -2.359069
C 4.571399 0.745034 -0.407026
C 3.699470 1.047628 2.323001
C -2.570724 2.722023 1.151577
C 2.454605 -2.757247 -1.405480
C -3.958984 -2.179367 0.615653
C -5.375279 -0.321176 1.239062
C -2.429315 -1.852458 -2.508097
C -4.690450 -1.339230 -3.217359
C 4.043757 1.996024 -0.739043
C 5.652287 0.238028 -1.133030
C 2.500251 1.760538 2.436376
C 4.761651 1.321787 3.188899
C -2.334119 3.946835 0.527258
C -3.684211 2.529706 1.968380
C 1.801864 -3.988576 -1.418808
C 3.774663 -2.633129 -1.837766
H -3.228703 -2.631089 -0.058568
C -4.473289 -2.923624 1.674410
C -5.877113 -1.062656 2.306828
H -5.746984 0.683728 1.022391
H -1.577081 -1.659826 -1.849362
C -2.341606 -2.819019 -3.509167
C -4.597354 -2.310459 -4.212878
H -5.594217 -0.734314 -3.102416
H 3.237317 2.427902 -0.144935
C 4.558149 2.708490 -1.818157
C 6.166334 0.951483 -2.215078
H 6.091534 -0.714428 -0.822802
H 1.662264 1.526698 1.773981
C 2.375313 2.754478 3.406158
C 4.632254 2.319274 4.153661
H 5.680526 0.734937 3.104678
H -1.460908 4.067215 -0.113024
C -3.227550 4.995155 0.730673
C -4.565421 3.587886 2.173327
H -3.860049 1.557565 2.431229
H 0.764784 -4.047943 -1.088499
C 2.492074 -5.116411 -1.861955
C 4.452619 -3.763636 -2.283151
H 4.261553 -1.657211 -1.834512
H -4.131809 -3.949294 1.829211
C -5.424206 -2.363844 2.527908
H -6.636860 -0.627496 2.960520
H -1.416032 -3.388576 -3.627496
C -3.423973 -3.051990 -4.358761
H -5.443039 -2.484910 -4.882473
H 4.131198 3.679720 -2.076521
C 5.614127 2.183244 -2.564466
H 7.007770 0.545629 -2.781617
H 1.434871 3.304612 3.494468
C 3.440289 3.037864 4.261964
H 5.464236 2.532944 4.829050
H -3.053206 5.956437 0.241930
C -4.341198 4.817115 1.552763

H -5.436705 3.448329 2.817143
H 1.990856 -6.086975 -1.873391
C 3.814471 -5.005502 -2.290662
H 5.486404 -3.674016 -2.623990
H -5.825933 -2.949869 3.358130
H -3.350684 -3.809036 -5.143538
H 6.016887 2.743839 -3.411500
H 3.339257 3.816116 5.022523
H -5.039878 5.642011 1.709572
H 4.351747 -5.891690 -2.636207
S -1.543712 1.325400 0.714568
O -0.212521 1.958836 0.420464
S 1.612741 -1.304958 -0.772156
O 0.235593 -1.839288 -0.489566
N 1.793344 -0.133693 -1.780202
C 1.669842 -0.392481 -3.204433
H 2.118959 0.457480 -3.739294
H 2.198901 -1.302181 -3.537063
H 0.616345 -0.472721 -3.525424
N -1.627698 0.234519 1.815342
C -1.358460 0.630508 3.191258
H -1.499957 1.707130 3.386188
H -2.037979 0.083654 3.862437
H -0.322698 0.377615 3.474632
Li -0.896141 -1.445496 0.979603
Li 0.992239 1.548383 -1.002164
O -1.232213 -3.043849 1.986588
C -0.814837 -4.326054 1.520174
C -1.380897 -3.045124 3.407864
C -1.120629 -5.278528 2.660887
H -1.357728 -4.552695 0.589956
H 0.265734 -4.294733 1.297586
C -0.850688 -4.393082 3.872701
H -0.822859 -2.194339 3.826886
H -2.449389 -2.910864 3.647738
H -0.499785 -6.184458 2.631414
H -2.178074 -5.587441 2.633441
H 0.232269 -4.332439 4.063758
H -1.339559 -4.741998 4.792664
O 1.356520 3.319869 -1.731297
C 1.299307 4.474370 -0.888141
C 1.025166 3.694495 -3.062479
C 0.557884 5.554561 -1.687128
H 0.780814 4.175983 0.034571
H 2.323224 4.787937 -0.623514
C -0.006125 4.791082 -2.886416
H 1.927124 4.061120 -3.587329
H 0.656366 2.802677 -3.588467
H 1.259377 6.332150 -2.024036
H -0.220980 6.052137 -1.092240
H -0.126243 5.416961 -3.781338
H -0.981511 4.338962 -2.647544

κ*O*,κ*N*-$Li_2(THF)_4$

E = –5943.74225062

C -3.756808 0.073296 -0.577335
S -2.182556 0.668452 -0.402962
P -5.087192 0.117379 0.577651
O -1.396811 0.119752 -1.558497
S -4.722508 0.865530 2.375659
Li 0.161823 1.349813 1.594188

Li -0.161771 -1.349956 -1.593967
O 1.396875 -0.119870 1.558640
S 2.182622 -0.668510 0.403079
C 3.756844 -0.073258 0.577344
P 5.087106 -0.117341 -0.577776
S 4.722210 -0.865646 -2.375676
Cl 4.224556 0.352679 2.223259
Cl -4.224426 -0.352511 -2.223313
C 6.446837 -1.047377 0.208020
C 6.152363 -2.098751 1.081851
C 7.774417 -0.794888 -0.154096
C 7.179704 -2.890383 1.591156
H 5.114154 -2.290380 1.364090
C 8.799850 -1.585400 0.360823
H 8.006589 0.020731 -0.843978
C 8.503741 -2.633766 1.233438
H 6.944694 -3.710196 2.274228
H 9.835792 -1.383126 0.078287
H 9.308934 -3.252853 1.636487
C 5.700548 1.606168 -0.689217
C 5.457207 2.343056 -1.852397
C 6.341949 2.219025 0.395878
C 5.831867 3.683336 -1.926298
H 4.981599 1.843336 -2.701089
C 6.717685 3.558895 0.316942
H 6.550984 1.648576 1.302706
C 6.459856 4.294020 -0.840870
H 5.637555 4.251737 -2.839043
H 7.219726 4.030561 1.165142
H 6.756972 5.343977 -0.899337
C -6.446792 1.047527 -0.208242
C -6.152180 2.098927 -1.081994
C -7.774421 0.795080 0.153724
C -7.179432 2.890632 -1.591366
H -5.113934 2.290521 -1.364121
C -8.799764 1.585666 -0.361262
H -8.006702 -0.020560 0.843542
C -8.503517 2.634061 -1.233795
H -6.944312 3.710466 -2.274375
H -9.835745 1.383427 -0.078840
H -9.308640 3.253204 -1.636897
C -5.700738 -1.606104 0.688873
C -5.457658 -2.343078 1.852052
C -6.341952 -2.218861 -0.396389
C -5.832393 -3.683346 1.925791
H -4.982194 -1.843434 2.700870
C -6.717764 -3.558719 -0.317615
H -6.550777 -1.648344 -1.303224
C -6.460194 -4.293931 0.840200
H -5.638287 -4.251815 2.838538
H -7.219656 -4.030308 -1.165945
H -6.757366 -5.343880 0.898539
C 2.148259 -2.438983 0.650438
C 1.462120 -2.965894 1.740566
C 2.908150 -3.246791 -0.198049
C 1.530217 -4.339521 1.980791
H 0.887889 -2.298439 2.383758
C 2.971805 -4.613793 0.056167
H 3.454782 -2.794673 -1.032305
C 2.283762 -5.160867 1.143546
H 0.992488 -4.766932 2.830272
H 3.564000 -5.257527 -0.598173

H 2.337902 -6.234494 1.338139
C -2.148047 2.438924 -0.650334
C -1.461790 2.965778 -1.740417
C -2.907909 3.246801 0.198111
C -1.529736 4.339413 -1.980636
H -0.887594 2.298272 -2.383586
C -2.971421 4.613811 -0.056104
H -3.454618 2.794738 1.032346
C -2.283257 5.160826 -1.143436
H -0.991911 4.766777 -2.830080
H -3.563594 5.257598 0.598204
H -2.337283 6.234460 -1.338026
N -1.622180 0.512315 1.035293
N 1.622210 -0.512422 -1.035167
C -1.698680 -0.807848 1.646672
H -2.736019 -1.115562 1.851083
H -1.200666 -1.597070 1.060211
H -1.183415 -0.747206 2.614725
C 1.698663 0.807722 -1.646586
H 2.735991 1.115483 -1.850982
H 1.183427 0.747025 -2.614650
H 1.200594 1.596938 -1.060164
O 1.118504 2.956100 0.987068
C 2.523209 3.160724 0.909170
C 0.541845 4.242578 1.156275
C 2.662153 4.368737 -0.002025
H 2.921977 3.368290 1.920190
H 2.990984 2.240460 0.536590
C 1.409403 5.200578 0.326765
H -0.503193 4.191480 0.829268
H 0.560935 4.508855 2.228768
H 2.651331 4.040626 -1.052275
H 3.605248 4.907134 0.163795
H 0.887494 5.520074 -0.585902
H 1.652061 6.105219 0.902426
O -0.138413 1.725663 3.471771
C 0.766501 1.262448 4.459103
C -1.427308 1.809615 4.069605
C -0.018040 0.190886 5.201495
H 1.666208 0.895076 3.946806
H 1.046677 2.095471 5.132315
C -1.458797 0.728644 5.163938
H -1.562805 2.817951 4.500028
H -2.171402 1.655698 3.274595
H 0.355001 0.023767 6.221648
H 0.059939 -0.763134 4.657592
H -1.752184 1.161511 6.131064
H -2.184689 -0.061942 4.926644
O 0.138348 -1.725859 -3.471559
C -0.766609 -1.262698 -4.458878
C 1.427242 -1.809771 -4.069407
C 0.017884 -0.191144 -5.201327
H -1.046785 -2.095749 -5.132057
H -1.666307 -0.895332 -3.946563
C 1.458636 -0.728909 -5.163852
H 2.171335 -1.655708 -3.274422
H 1.562819 -2.818141 -4.499723
H -0.060061 0.762877 -4.657421
H -0.355217 -0.024027 -6.221459
H 2.184564 0.061692 -4.926719
H 1.751913 -1.161882 -6.130964
O -1.118418 -2.956183 -0.986632

C -2.523166 -3.160663 -0.909107
C -0.541939 -4.242755 -1.155616
C -2.662576 -4.368652 0.002094
H -2.990946 -2.240340 -0.536677
H -2.921650 -3.368199 -1.920242
C -1.409400 -5.200338 -0.325572
H -0.561402 -4.509370 -2.228024
H 0.503202 -4.191696 -0.828934
H -3.605437 -4.907226 -0.164483
H -2.652718 -4.040501 1.052335
H -1.651453 -6.105640 -0.900444
H -0.887651 -5.518713 0.587577

κ*O*,*κN*,κ*S*,*κCl*-Li$_2$

E = –5014.57443118

Cl 2.197995 0.756872 -2.059982
Cl -2.198778 -0.757548 2.060209
S 3.004376 1.004370 2.629309
S -3.003687 -1.003948 -2.629469
C 2.159025 0.287518 -0.358590
C -2.159389 -0.287658 0.359022
P 3.368532 1.078522 0.666307
P -3.368407 -1.078524 -0.666583
C 3.331247 2.798256 0.071500
C 5.052197 0.452376 0.324757
C -3.331138 -2.798424 -0.072277
C -5.052281 -0.452645 -0.325479
C 2.989530 -2.460562 -0.282100
C -2.989614 2.460699 0.283324
C 4.487047 3.456569 -0.353116
C 2.105274 3.476589 0.105262
C 5.375383 -0.015356 -0.956287
C 6.001218 0.381438 1.348719
C -4.487068 -3.457160 0.351322
C -2.105017 -3.476509 -0.105652
C -5.376410 0.013182 0.956034
C -6.000452 -0.379895 -1.350085
C 3.193717 -3.150946 -1.474503
C 3.909613 -2.525012 0.767507
C -3.193445 3.151324 1.475643
C -3.909698 2.525455 -0.766263
H 5.442143 2.927006 -0.379609
C 4.417061 4.793110 -0.749504
C 2.042885 4.807322 -0.295466
H 1.200982 2.955399 0.433253
H 4.634866 0.022989 -1.757314
C 6.635692 -0.552873 -1.202560
C 7.263469 -0.152887 1.095690
H 5.730797 0.727905 2.349278
H -5.442281 -2.927787 0.377470
C -4.417060 -4.793864 0.747154
C -2.042611 -4.807411 0.294521
H -1.200654 -2.955023 -0.432977
H -4.636563 -0.026620 1.757610
C -6.636799 0.550595 1.202133
C -7.262759 0.154409 -1.097278
H -5.729269 -0.724895 -2.350945
H 2.451836 -3.077204 -2.270852
C 4.346948 -3.922594 -1.616167
C 5.053361 -3.301397 0.614688
H 3.733047 -1.954835 1.683522

H -2.451586 3.077318 2.271985
C -4.346273 3.923600 1.617210
C -5.053077 3.302397 -0.613526
H -3.733355 1.955221 -1.682278
H 5.321656 5.306908 -1.083133
C 3.197946 5.467500 -0.722909
H 1.085144 5.333128 -0.277052
H 6.877084 -0.929260 -2.199208
C 7.580599 -0.623251 -0.178296
H 7.999811 -0.209461 1.900921
H -5.321756 -5.307985 1.080008
C -3.197795 -5.468001 0.720997
H -1.084759 -5.333026 0.276417
H -6.878963 0.925412 2.199184
C -7.580819 0.622875 0.177171
H -7.998416 0.212436 -1.903032
H 4.521204 -4.469307 -2.545773
C 5.273452 -3.996635 -0.576226
H 5.782461 -3.354988 1.425825
H -4.520219 4.470553 2.546733
C -5.272760 3.997969 0.577278
H -5.782198 3.356169 -1.424629
H 3.145131 6.512825 -1.036693
H 8.566874 -1.051406 -0.373187
H -3.144965 -6.513454 1.034355
H -8.567143 1.050982 0.371918
H 6.176587 -4.599819 -0.694341
H -6.175565 4.601665 0.695311
S 1.624182 -1.313905 -0.128070
O 0.725765 -1.680156 -1.277144
S -1.624487 1.313774 0.129109
O -0.726174 1.679698 1.278394
N -1.097573 1.342897 -1.322871
C -0.867674 2.610221 -1.989147
H -1.232265 2.540780 -3.026611
H -1.382590 3.464253 -1.516262
H 0.209159 2.840087 -2.023966
N 1.097542 -1.342590 1.324028
C 0.867482 -2.609731 1.990570
H 1.384648 -3.463442 1.519546
H 1.229553 -2.539011 3.028813
H -0.209132 -2.840970 2.023116
Li 0.620114 0.495813 1.952281
Li -0.619636 -0.495055 -1.952190

κ*N*,κ*S*,κ*Cl*-Li$_2$

E = –5014.55088939

Cl -1.731140 0.593419 -2.591268
Cl 1.731266 -0.593490 2.591386
S -1.248907 0.742870 1.321988
S 1.248841 -0.742879 -1.321812
C -2.981221 0.172818 -1.400012
C 2.981288 -0.172838 1.400079
P -2.820137 1.100768 0.081931
P 2.820136 -1.100790 -0.081859
C -4.375863 0.979084 1.015816
C -2.742516 2.836726 -0.480535
C 4.375820 -0.979077 -1.015812
C 2.742614 -2.836757 0.480601
C -4.852738 -1.802189 -0.697066
C 4.852677 1.802221 0.696953

C -5.593540 1.150793 0.344922
C -4.357128 0.784121 2.397940
C -3.466323 3.243758 -1.608165
C -1.996467 3.769397 0.245301
C 5.593541 -1.150612 -0.344953
C 4.357007 -0.784242 -2.397953
C 3.466476 -3.243755 1.608207
C 1.996593 -3.769464 -0.245217
C -4.956765 -2.102996 0.658545
C -5.972539 -1.745429 -1.524835
C 4.956543 2.103231 -0.658627
C 5.972580 1.745299 1.524571
H -5.604260 1.289356 -0.737749
C -6.785526 1.115197 1.059959
C -5.555896 0.752560 3.109349
H -3.398534 0.652944 2.906015
H -4.029842 2.506165 -2.183972
C -3.437841 4.578379 -2.003864
C -1.971851 5.104812 -0.156135
H -1.430292 3.440104 1.120514
H 5.604319 -1.289062 0.737731
C 6.785493 -1.114975 -1.060045
C 5.555741 -0.752645 -3.109417
H 3.398379 -0.653190 -2.905995
H 4.029962 -2.506133 2.184009
C 3.438077 -4.578379 2.003902
C 1.972062 -5.104883 0.156213
H 1.430378 -3.440192 -1.120412
H -4.054856 -2.144972 1.272303
C -6.218452 -2.337838 1.200553
C -7.228548 -1.979235 -0.971964
H -5.845758 -1.527265 -2.586516
H 4.054557 2.145358 -1.272262
C 6.218169 2.338102 -1.200760
C 7.228528 1.979138 0.971575
H 5.845921 1.526976 2.586234
H -7.735399 1.235327 0.534601
C -6.768032 0.916980 2.442114
H -5.540249 0.596569 4.190440
H -3.996169 4.892140 -2.888917
C -2.691532 5.509861 -1.279598
H -1.385111 5.829887 0.412689
H 7.735401 -1.234964 -0.534717
C 6.767921 -0.916891 -2.442219
H 5.540034 -0.596759 -4.190522
H 3.996444 -4.892114 2.888940
C 2.691798 -5.509898 1.279652
H 1.385344 -5.829987 -0.412597
H -6.317573 -2.567037 2.263490
C -7.349915 -2.273404 0.387605
H -8.117546 -1.936363 -1.605260
H 6.317164 2.567457 -2.263675
C 7.349734 2.273500 -0.387966
H 8.117605 1.936142 1.604752
H -7.707142 0.887204 2.999809
H -2.668663 6.555390 -1.596057
H 7.707004 -0.887081 -2.999957
H 2.668997 -6.555430 1.596106
H -8.337748 -2.455163 0.817395
H 8.337519 2.455285 -0.817855
S -3.241076 -1.532019 -1.426726
S 3.241094 1.532003 1.426769

N -2.171063 -2.262695 -0.534921
O -3.356053 -1.987812 -2.827968
C -2.202394 -3.718242 -0.597977
H -1.390037 -4.110623 0.032363
H -3.147976 -4.151741 -0.220464
H -2.044796 -4.101554 -1.620889
N 2.170989 2.262628 0.535035
O 3.356164 1.987804 2.828003
C 2.202264 3.718176 0.598071
H 3.147794 4.151706 0.220460
H 1.389826 4.110515 -0.032192
H 2.044752 4.101498 1.620992
Li -0.605600 -1.372502 0.209895
Li 0.605525 1.372384 -0.209676

κ*O*,κ*S*,κ*Cl*-Li$_2$

E = –5014.54493149

Cl 1.687089 -0.684620 -2.542398
Cl -1.665168 0.756919 2.499473
S 0.918041 -1.339170 1.094720
S -0.948205 1.343624 -1.154886
C 2.798511 -0.180265 -1.267644
C -2.784192 0.212346 1.248644
P 2.700037 -1.234419 0.135051
P -2.718119 1.241390 -0.173915
C 4.024791 -0.727692 1.272984
C 3.159032 -2.888702 -0.467337
C -4.053725 0.696391 -1.280902
C -3.186833 2.901794 0.403039
C 4.527012 1.912659 -0.587815
C -4.497341 -1.920312 0.656879
C 5.359248 -0.826829 0.859320
C 3.719177 -0.243945 2.545611
C 3.997408 -3.031571 -1.578988
C 2.704083 -4.019136 0.218635
C -5.381661 0.778079 -0.843221
C -3.763404 0.199156 -2.551849
C -4.016711 3.056241 1.519562
C -2.748587 4.024184 -0.306507
C 4.881040 2.569659 0.585410
C 5.467287 1.622333 -1.576364
C -4.875682 -2.603915 -0.493358
C -5.412999 -1.623405 1.666320
H 5.599004 -1.199830 -0.138354
C 6.380282 -0.435715 1.718699
C 4.746200 0.151244 3.401403
H 2.673478 -0.184735 2.856583
H 4.327924 -2.141224 -2.119367
C 4.383193 -4.304260 -1.994720
C 3.097832 -5.288648 -0.199092
H 2.031541 -3.894422 1.071175
H -5.609203 1.161169 0.153479
C -6.411565 0.355630 -1.676877
C -4.799074 -0.227433 -3.381733
H -2.722689 0.154090 -2.881518
H -4.333923 2.172592 2.078616
C -4.410966 4.332289 1.916461
C -3.150594 5.297085 0.092700
H -2.082389 3.891071 -1.162744
H 4.115903 2.774498 1.334434
C 6.216530 2.920679 0.783788

C 6.796660 1.973487 -1.366825
H 5.149755 1.111832 -2.488321
H -4.129715 -2.812063 -1.260592
C -6.211358 -2.976155 -0.646714
C -6.742967 -1.995595 1.501571
H -5.076479 -1.091154 2.558904
H 7.420021 -0.506634 1.392534
C 6.074288 0.055814 2.989036
H 4.505970 0.532951 4.396393
H 5.031799 -4.416085 -2.866681
C 3.937589 -5.431885 -1.304194
H 2.742484 -6.170769 0.338553
H -7.446011 0.412622 -1.331706
C -6.120718 -0.149822 -2.945253
H -4.570907 -0.619791 -4.375420
H -5.052912 4.453283 2.792112
C -3.981978 5.451781 1.202533
H -2.808128 6.172945 -0.463214
H 6.511636 3.423718 1.707409
C 7.171413 2.620460 -0.185627
H 7.546314 1.742218 -2.127043
H -6.525788 -3.500439 -1.551974
C -7.142338 -2.669791 0.343830
H -7.473834 -1.759488 2.278411
H 6.878002 0.366769 3.660785
H 4.241044 -6.428594 -1.633493
H -6.931128 -0.485653 -3.596679
H -4.291876 6.451228 1.517201
H 8.217328 2.890905 -0.022503
H -8.188736 -2.956763 0.215803
S 2.810076 1.511710 -0.932212
S -2.777631 -1.485464 0.941923
O 2.141561 1.861103 0.383232
N 2.338246 2.200858 -2.220807
N -2.252480 -2.142280 2.226472
O -2.146527 -1.843532 -0.389408
C -2.222930 -3.589678 2.237171
H -1.567577 -4.018305 1.456250
H -1.825586 -3.916117 3.209412
H -3.223359 -4.053553 2.120812
C 2.343155 3.648947 -2.204219
H 1.998735 4.003352 -3.186767
H 1.662648 4.078158 -1.445351
H 3.347524 4.086641 -2.033094
Li 0.477521 1.169935 0.949891
Li -0.495544 -1.160905 -1.000048

κ*O*,κ*N*-Li_2

E = –5014.54769161
C -3.854938 -0.821620 -0.620586
S -2.269042 -0.244514 -0.428446
P -5.109875 -0.460811 0.586464
O -1.561936 -0.532465 -1.724170
S -4.646061 -0.849735 2.473458
Li -0.347295 0.402468 1.901249
Li 0.281922 -0.587365 -2.047543
O 1.489172 0.558365 1.581917
S 2.232941 0.188899 0.327189
C 3.791700 0.845508 0.494722
P 5.088713 0.422085 -0.644679
S 4.653803 0.606654 -2.568729

Cl 4.312745 1.128401 2.159494
Cl -4.420696 -0.913212 -2.292740
C 5.711724 -1.269709 -0.301253
C 5.550784 -1.851444 0.962491
C 6.312961 -2.004112 -1.328209
C 5.979694 -3.157511 1.188330
H 5.078369 -1.287100 1.768438
C 6.748645 -3.307073 -1.096044
H 6.407324 -1.548179 -2.317075
C 6.578730 -3.886910 0.161377
H 5.836356 -3.610563 2.172053
H 7.214255 -3.876204 -1.904408
H 6.909808 -4.912870 0.340286
C 6.448494 1.536437 -0.155040
C 6.308000 2.900440 -0.436669
C 7.611085 1.068510 0.461375
C 7.322110 3.789743 -0.095004
H 5.400535 3.257855 -0.930104
C 8.628048 1.963045 0.797672
H 7.723975 0.004878 0.682112
C 8.484412 3.321637 0.522754
H 7.208375 4.854242 -0.313509
H 9.536854 1.593579 1.278675
H 9.281413 4.020500 0.788056
C -5.667918 1.280191 0.424804
C -5.524023 1.969649 -0.785922
C -6.192057 1.943276 1.538807
C -5.890556 3.310754 -0.872051
H -5.111462 1.461612 -1.659334
C -6.566950 3.282155 1.445958
H -6.274687 1.401493 2.484378
C -6.411786 3.969054 0.241874
H -5.759454 3.846921 -1.814842
H -6.972494 3.794529 2.321822
H -6.693795 5.022531 0.172320
C -6.518963 -1.471551 0.022940
C -6.366869 -2.863438 0.025297
C -7.735673 -0.903678 -0.360427
C -7.425500 -3.679228 -0.360553
H -5.412443 -3.300367 0.330282
C -8.796487 -1.726176 -0.743020
H -7.856321 0.181872 -0.364498
C -8.642812 -3.111066 -0.745045
H -7.303160 -4.764991 -0.361775
H -9.747305 -1.279091 -1.042755
H -9.474048 -3.752994 -1.046643
C 2.270787 -1.594950 0.308123
C 2.040141 -2.278090 1.501107
C 2.700117 -2.263415 -0.841362
C 2.204234 -3.661905 1.530997
H 1.738296 -1.724222 2.391098
C 2.857990 -3.645753 -0.797544
H 2.937979 -1.694559 -1.743835
C 2.607492 -4.345204 0.384177
H 2.016411 -4.208208 2.458034
H 3.195470 -4.177830 -1.689537
H 2.738475 -5.429184 0.413612
C -2.225629 1.531099 -0.238844
C -1.958930 2.314810 -1.359866
C -2.625317 2.106273 0.971884
C -2.052927 3.701487 -1.254737
H -1.680364 1.835631 -2.299075

C -2.711663 3.492665 1.063028
H -2.899258 1.467625 1.816442
C -2.422966 4.289882 -0.045904
H -1.834774 4.324882 -2.124711
H -3.024461 3.951670 2.003244
H -2.497920 5.376849 0.030717
N -1.600032 -0.731041 0.890200
N 1.576590 0.533894 -1.041748
C -1.646363 -2.165144 1.151946
H -2.605092 -2.456454 1.610444
H -1.480339 -2.779804 0.250655
H -0.838802 -2.406115 1.859035
C 1.591096 1.941490 -1.425513
H 2.586918 2.255642 -1.775987
H 0.885771 2.067260 -2.260207
H 1.258796 2.617729 -0.619230

κ*O*,κ*S*-Li$_2$

E = –5014.54537659

Cl -2.415761 -0.624826 2.788636
Cl 2.417420 0.628673 -2.787575
S -0.835572 -1.514350 -0.947856
S 0.835353 1.513656 0.949595
C -2.893837 -0.140338 1.180542
C 2.893696 0.141751 -1.179689
P -2.678771 -1.284081 -0.120320
P 2.678780 1.283943 0.122553
C -3.894783 -0.855318 -1.412671
C -3.237762 -2.891176 0.535523
C 3.894195 0.853697 1.414980
C 3.238316 2.891620 -0.531425
C -4.539348 2.079559 0.513492
C 4.539343 -2.079872 -0.518634
C -5.236016 -0.679430 -1.053073
C -3.509223 -0.731018 -2.748296
C -4.303112 -2.953538 1.443091
C -2.643811 -4.069797 0.076309
C 5.235191 0.675816 1.055532
C 3.508412 0.730650 2.750676
C 4.304701 2.954720 -1.437734
C 2.643700 4.069842 -0.072093
C -4.927943 2.651306 -0.692921
C -5.434038 1.938664 1.575211
C 4.929747 -2.653406 0.686339
C 5.432290 -1.937980 -1.581708
H -5.536265 -0.756448 -0.006549
C -6.183087 -0.377500 -2.027199
C -4.459402 -0.420185 -3.719930
H -2.459757 -0.878275 -3.015930
H -4.750182 -2.031682 1.820875
C -4.771188 -4.190304 1.878807
C -3.117202 -5.305577 0.515301
H -1.804978 -4.007351 -0.621779
H 5.535617 0.751843 0.008980
C 6.181812 0.373077 2.029870
C 4.458123 0.419033 3.722502
H 2.459139 0.879506 3.018193
H 4.752287 2.033187 -1.815688
C 4.773155 4.191828 -1.872041
C 3.117467 5.305983 -0.509684
H 1.804092 4.006801 0.625010

H -4.201243 2.736155 -1.501613
C -6.248740 3.074805 -0.845629
C -6.749948 2.359286 1.411853
H -5.094802 1.492330 2.513472
H 4.204389 -2.739053 1.496143
C 6.250609 -3.077755 0.836198
C 6.748278 -2.359430 -1.421178
H 5.091658 -1.490286 -2.518820
H -7.226660 -0.231986 -1.739631
C -5.795673 -0.243639 -3.360988
H -4.153211 -0.317365 -4.763635
H -5.598391 -4.235668 2.590976
C -4.180912 -5.367054 1.414525
H -2.649545 -6.224228 0.153513
H 7.225189 0.225975 1.742398
C 5.794168 0.240432 3.363702
H 4.151759 0.317209 4.766254
H 5.601176 4.237782 -2.583220
C 4.182210 5.368196 -1.407622
H 2.649285 6.224333 -0.147809
H -6.569464 3.516080 -1.792159
C -7.157794 2.925910 0.200291
H -7.462590 2.246823 2.232205
H 6.572743 -3.520497 1.781563
C 7.157932 -2.927899 -0.211090
H 7.459557 -2.246225 -2.242609
H -6.538838 0.000986 -4.123770
H -4.549384 -6.335806 1.760526
H 6.536962 -0.004834 4.126640
H 4.550984 6.337226 -1.752522
H -8.192422 3.253729 0.074808
H 8.192607 -3.256416 -0.087840
S -2.852581 1.517410 0.782002
O -2.263980 1.656834 -0.607822
S 2.852442 -1.516460 -0.783634
O 2.265716 -1.657205 0.606854
N 2.248982 -2.253140 -1.984478
C 2.298825 -3.698776 -1.983476
H 1.959219 -4.056102 -2.966924
H 3.314916 -4.109816 -1.819455
H 1.633144 -4.152213 -1.225764
N -2.251288 2.255955 1.982816
C -2.301232 3.701593 1.979185
H -3.317205 4.112260 1.813481
H -1.962600 4.060673 2.962329
H -1.634892 4.153791 1.221310
Li 0.552373 -1.005657 1.088194
Li -0.550837 1.004559 -1.087498

κ*S*,κ*Cl*-Li$_2$

E = –5014.55089132

Cl 1.731505 0.594659 2.590935
Cl -1.731199 -0.595370 -2.590556
S 1.248339 0.745082 -1.321327
S -1.248215 -0.744801 1.321575
C 2.981476 0.173306 1.399790
C -2.981325 -0.173705 -1.399690
P 2.820599 1.101346 -0.082125
P -2.820522 -1.101295 0.082511
C 4.375656 0.977812 -1.016824
C 2.745051 2.837385 0.480308

C -4.375544 -0.977306 1.017206
C -2.745105 -2.837493 -0.479444
C 4.852099 -1.802692 0.697221
C -4.852239 1.802425 -0.698242
C 5.593808 1.149264 -0.346743
C 4.356020 0.781548 -2.398755
C 3.469320 3.243611 1.607922
C 1.999887 3.770844 -0.245428
C -5.593738 -1.148941 0.347243
C -4.355841 -0.780495 2.399058
C -3.469247 -3.243915 -1.607069
C -2.000127 -3.770848 0.246612
C 4.955954 -2.104709 -0.658134
C 5.971926 -1.745538 1.524909
C -4.956545 2.105144 0.656921
C -5.971806 1.744731 -1.526243
H 5.605266 1.288853 0.735788
C 6.785386 1.112161 -1.062395
C 5.554378 0.748495 -3.110777
H 3.397063 0.650572 -2.906196
H 4.032088 2.505381 2.183651
C 3.442289 4.578253 2.003660
C 1.976800 5.106289 0.155992
H 1.433088 3.442165 -1.120466
H -5.605261 -1.288929 -0.735235
C -6.785281 -1.111486 1.062934
C -5.554164 -0.747101 3.111123
H -3.396851 -0.649401 2.906403
H -4.031831 -2.505760 -2.183075
C -3.442291 -4.578654 -2.002488
C -1.977117 -5.106391 -0.154486
H -1.433414 -3.442010 1.121644
H 4.053954 -2.146980 -1.271742
C 6.217532 -2.340413 -1.200000
C 7.227841 -1.980181 0.972161
H 5.845284 -1.526453 2.586416
H -4.054737 2.147824 1.270780
C -6.218314 2.341003 1.198274
C -7.227916 1.979531 -0.974007
H -5.844818 1.525114 -2.587599
H 7.735617 1.232094 -0.537640
C 6.766996 0.912693 -2.444353
H 5.538030 0.591531 -4.191717
H 4.000949 4.891390 2.888726
C 2.696985 5.510555 1.279422
H 1.390836 5.832006 -0.412811
H -7.735542 -1.231568 0.538270
C -6.766818 -0.911493 2.444816
H -5.537762 -0.589717 4.192001
H -4.000844 -4.891943 -2.887568
C -2.697185 -5.510856 -1.277920
H -1.391301 -5.832029 0.414571
H 6.316532 -2.570605 -2.262733
C 7.349049 -2.275593 -0.387152
H 8.116899 -1.937033 1.605354
H -6.317664 2.571741 2.260856
C -7.349575 2.275643 0.385113
H -8.116773 1.935960 -1.607453
H 7.705778 0.881753 -3.002536
H 2.675250 6.556094 1.595928
H -7.705572 -0.880286 3.003029
H -2.675506 -6.556473 -1.594173

H 8.336814 -2.458046 -0.816802
H -8.337493 2.458220 0.814361
S 3.240497 -1.531723 1.426762
S -3.240384 1.531305 -1.427172
N 2.170154 -2.262320 0.535182
O 3.355260 -1.987224 2.828122
C 2.200872 -3.717848 0.599339
H 1.387525 -4.110300 -0.029667
H 3.145798 -4.152057 0.221001
H 2.044406 -4.100234 1.622759
N -2.170337 2.262159 -0.535459
O -3.354731 1.986443 -2.828685
C -2.200986 3.717673 -0.600088
H -3.145896 4.152056 -0.221909
H -1.387627 4.110295 0.028802
H -2.044481 4.099719 -1.623629
Li -0.606153 1.370738 0.210383
Li 0.606192 -1.370181 -0.210154

κ*O*,κ*N*,κ*Cl*-Li$_2$

E = –5014.55606304

Cl 1.921440 0.071309 -2.546321
Cl -1.921525 -0.070102 2.546259
S 5.353018 -0.011028 -0.829781
S -5.352780 0.010706 0.830311
C 2.120394 -0.023350 -0.779888
C -2.120193 0.023676 0.779668
P 3.631420 0.781147 -0.265743
P -3.631171 -0.781218 0.265980
C 3.542412 0.974258 1.555745
C 3.395005 2.473000 -0.915005
C -3.542616 -0.974627 -1.555492
C -3.394248 -2.472915 0.915412
C 2.666591 -2.892400 -0.494323
C -2.666913 2.892459 0.494016
C 2.697387 1.923756 2.146450
C 4.347943 0.170621 2.365587
C 2.116888 3.040224 -0.992621
C 4.514785 3.211612 -1.303014
C -2.697896 -1.924364 -2.146259
C -4.348237 -0.171010 -2.365268
C -2.115979 -3.039790 0.992971
C -4.513815 -3.211817 1.303505
C 2.391347 -3.769220 -1.542870
C 3.828787 -3.011937 0.268621
C -2.391624 3.769389 1.542451
C -3.829272 3.011739 -0.268721
H 2.090995 2.588866 1.527302
C 2.626074 2.025961 3.536443
C 4.269833 0.270976 3.753484
H 5.049648 -0.519769 1.890119
H 1.245270 2.454515 -0.690837
C 1.969070 4.348575 -1.447854
C 4.360171 4.520492 -1.757757
H 5.501272 2.742201 -1.254430
H -2.091434 -2.589442 -1.527138
C -2.627017 -2.026881 -3.536245
C -4.270541 -0.271665 -3.753167
H -5.049696 0.519596 -1.889756
H -1.244556 -2.453825 0.691132
C -1.967789 -4.348107 1.448193

C -4.358825 -4.520646 1.758247
H -5.500423 -2.742654 1.254972
H 1.470230 -3.651057 -2.114165
C 3.300271 -4.786808 -1.827247
C 4.723969 -4.037192 -0.019096
H 4.023956 -2.314681 1.083814
H -1.470376 3.651432 2.113580
C -3.300702 4.786802 1.826960
C -4.724604 4.036832 0.019112
H -4.024439 2.314391 -1.083836
H 1.965535 2.769078 3.989628
C 3.402049 1.190769 4.341938
H 4.896901 -0.368601 4.379031
H 0.970996 4.789615 -1.508284
C 3.088639 5.089803 -1.829522
H 5.237245 5.096637 -2.061982
H -1.966734 -2.770198 -3.989470
C -3.403098 -1.191736 -4.341685
H -4.897681 0.367897 -4.378657
H -0.969591 -4.788876 1.508549
C -3.087136 -5.089629 1.829923
H -5.235723 -5.097020 2.062552
H 3.096246 -5.481013 -2.645435
C 4.462423 -4.920931 -1.067422
H 5.633993 -4.142323 0.575324
H -3.096662 5.481081 2.645083
C -4.463023 4.920669 1.067350
H -5.634754 4.141765 -0.575149
H 3.343249 1.271397 5.429790
H 2.968521 6.114517 -2.189648
H -3.344626 -1.272601 -5.429538
H -2.966736 -6.114314 2.190034
H 5.170626 -5.721634 -1.292611
H -5.171327 5.721255 1.292636
S 1.568633 -1.517724 -0.182616
O 0.346688 -1.881671 -0.987866
S -1.568715 1.518024 0.182025
O -0.346564 1.882332 0.986816
N -1.440155 1.294088 -1.349189
C -1.354759 2.425625 -2.258138
H -2.132727 2.337656 -3.034258
H -1.484264 3.405395 -1.769616
H -0.373932 2.432965 -2.760699
N 1.439590 -1.294024 1.348620
C 1.354440 -2.425672 2.257461
H 1.481471 -3.405525 1.768453
H 2.133956 -2.339123 3.032204
H 0.374532 -2.431649 2.761816
Li 0.612825 0.448864 1.786211
Li -0.613464 -0.448874 -1.786650

6.4.3 Coordinates of the monomeric structures of 1-Na

κ*O*-Na(THF)$_3$

E = –3358.82170887

O 0.556258 0.193141 0.155741
O 2.683118 2.571931 1.493066
O 4.128284 -0.481801 -0.171687
O 2.005202 2.195660 -2.181737
S 0.264817 -1.093700 0.879601
C 1.662285 3.556623 1.643864
C 3.020284 2.000525 2.760203
C 4.823006 -1.023535 -1.293784
C 4.276458 -1.329895 0.971690
C 2.094572 3.607461 -2.332028
C 0.865302 1.692664 -2.896308
N 0.873730 -1.366206 2.273852
C -1.433687 -1.205411 0.840699
C 1.007850 -2.385528 -0.102286
H 0.928119 3.437180 0.832067
H 2.109340 4.564429 1.565388
C 1.072870 3.317622 3.021763
C 2.292350 2.838447 3.800997
H 4.114907 2.013024 2.878593
H 2.682092 0.949075 2.777107
H 5.425431 -0.228726 -1.761226
H 4.084887 -1.382126 -2.032479
C 5.657163 -2.171950 -0.747591
C 4.804287 -2.643461 0.424987
H 3.299882 -1.418233 1.474262
H 4.996095 -0.866396 1.671730
H 2.805654 3.857205 -3.141787
H 2.480129 4.036235 -1.394116
C 0.687087 4.050899 -2.681350
C 0.228595 2.901201 -3.571796
H 0.183423 1.209065 -2.178336
H 1.200452 0.929410 -3.616894
C 0.241156 -0.739849 3.415500
Cl -2.031869 -2.742842 1.467284
P -2.330495 -0.472258 -0.494967
C 1.267791 -2.134704 -1.449033
C 1.205380 -3.651658 0.450127
H 0.312551 2.522676 2.970852
H 0.601424 4.215236 3.445393
H 2.910834 3.695990 4.109706
H 2.039017 2.260910 4.701034
H 6.633544 -1.805317 -0.391966
H 5.840185 -2.951577 -1.500414
H 3.968879 -3.263954 0.066967
H 5.364666 -3.219345 1.174784
H 0.656277 5.032379 -3.175136
H 0.067092 4.099611 -1.771703
H 0.620829 3.031434 -4.592855
H -0.863665 2.807911 -3.638405
H -0.837781 -0.967671 3.488967
H 0.347482 0.361032 3.438663
H 0.722927 -1.124813 4.327032
S -1.959883 -0.974783 -2.385718
C -4.072200 -0.842017 -0.051512
C -2.257188 1.346282 -0.272346
H 1.071408 -1.144263 -1.859347
C 1.761753 -3.165016 -2.247007

C 1.688810 -4.676893 -0.360315
H 0.996576 -3.807245 1.508777
C -4.564006 -0.489816 1.211599
C -4.914417 -1.455709 -0.977789
C -2.587579 2.165254 -1.355237
C -1.973572 1.921319 0.969965
H 1.976469 -2.975782 -3.301392
C 1.970208 -4.434322 -1.706132
H 1.852629 -5.670324 0.063689
H -3.902456 -0.016643 1.941083
C -5.891598 -0.750301 1.538536
C -6.244561 -1.718239 -0.647050
H -4.507657 -1.719858 -1.958310
H -2.794836 1.699097 -2.322417
C -2.637248 3.549499 -1.198207
C -2.022654 3.305881 1.124086
H -1.704541 1.270561 1.805102
H 2.353327 -5.239892 -2.337018
H -6.272520 -0.474463 2.524907
C -6.734183 -1.365396 0.609213
H -6.900362 -2.200978 -1.375853
H -2.895260 4.183854 -2.049873
C -2.354334 4.122500 0.042009
H -1.799435 3.751702 2.096311
H -7.776073 -1.569667 0.868270
H -2.391880 5.207605 0.165997
Na 2.534666 1.117881 -0.255583

κ*S*-Na(THF)$_5$

E = –3823.38603574

Cl 2.361663 1.063208 -2.373846
S -0.170011 0.605228 1.406888
C 2.208608 0.251013 -0.834168
P 1.620813 1.040505 0.616610
C 2.900560 0.875695 1.921060
C 1.681377 2.828184 0.196723
C 4.321484 -1.628995 -0.933969
C 4.251554 0.814990 1.564499
C 2.540930 0.860815 3.270812
C 2.872111 3.423221 -0.239243
C 0.537070 3.611483 0.352187
C 5.053701 -2.071558 0.165662
C 4.949757 -1.328861 -2.145158
H 4.530880 0.794077 0.508706
C 5.233590 0.745393 2.550655
C 3.524608 0.789851 4.255900
H 1.480135 0.891953 3.532541
H 3.772432 2.820142 -0.369901
C 2.909509 4.786566 -0.518128
C 0.574563 4.976666 0.066885
H -0.377008 3.127804 0.708281
H 4.524439 -2.300300 1.091830
C 6.438705 -2.193836 0.058692
C 6.334653 -1.447390 -2.240269
H 4.352218 -1.001440 -2.998205
H 6.286026 0.686684 2.262430
C 4.872214 0.733937 3.898096
H 3.236488 0.773859 5.309990
H 3.841782 5.244453 -0.857282
C 1.760163 5.565735 -0.368335
H -0.326458 5.582872 0.190584

H 7.020830 -2.536131 0.917931
C 7.079576 -1.877338 -1.139654
H 6.836221 -1.208909 -3.181342
H 5.642320 0.673812 4.671186
H 1.792435 6.635429 -0.589519
H 8.165256 -1.971382 -1.219723
S 2.534898 -1.407896 -0.790733
Na -2.596314 -0.341350 0.143060
N 1.878147 -2.069390 -2.025345
O 2.230009 -1.864670 0.595741
C 2.261530 -3.432436 -2.327524
H 2.100037 -4.138010 -1.490381
H 1.645050 -3.785529 -3.168479
H 3.320637 -3.536794 -2.636449
O -4.632565 -1.377950 -0.423698
C -4.675463 -2.345375 -1.468436
C -5.938127 -0.898107 -0.148046
C -6.135927 -2.439228 -1.907612
H -4.020779 -1.998930 -2.286070
H -4.277876 -3.304056 -1.098322
C -6.705545 -1.102699 -1.440606
H -6.393475 -1.480823 0.675574
H -5.851929 0.148009 0.176794
H -6.642498 -3.267367 -1.387492
H -6.239977 -2.609382 -2.988289
H -7.795409 -1.114371 -1.298921
H -6.459125 -0.302956 -2.157662
O -1.766770 -2.521669 0.453731
C -1.292233 -2.930460 1.735519
C -1.117814 -3.275975 -0.567638
C -0.202072 -3.961413 1.474486
H -0.922677 -2.045861 2.276759
H -2.136096 -3.366206 2.299871
C -0.625471 -4.532668 0.125736
H -1.843601 -3.465937 -1.373042
H -0.271174 -2.700129 -0.979185
H -0.132819 -4.712463 2.274634
H 0.769417 -3.455344 1.369085
H -1.446220 -5.260279 0.242781
H 0.193051 -5.022959 -0.420339
O -3.380463 -0.235931 2.379996
C -4.219675 -1.254369 2.894391
C -2.973686 0.545678 3.486233
C -3.666157 -1.602901 4.286687
H -5.256027 -0.875391 2.969903
H -4.211820 -2.086537 2.177434
C -2.659334 -0.474631 4.571582
H -2.109346 1.150695 3.178689
H -3.796618 1.220548 3.795071
H -3.177953 -2.587318 4.297120
H -4.474430 -1.631176 5.031410
H -1.626394 -0.832718 4.448491
H -2.752474 -0.055748 5.583342
O -1.810151 0.139306 -2.026428
C -1.170282 1.399027 -2.140481
C -1.202498 -0.701615 -3.002496
C -1.064019 1.630360 -3.637840
H -1.769778 2.137177 -1.596114
H -0.171365 1.355207 -1.678455
C -0.812877 0.213406 -4.177088
H -0.305206 -1.182140 -2.576607
H -1.932109 -1.479211 -3.273336

H -0.260299 2.336012 -3.890481
H -2.010722 2.037820 -4.026921
H 0.246583 0.065868 -4.428354
H -1.405951 0.010589 -5.080368
O -3.796890 1.703899 -0.207974
C -4.465396 2.086954 -1.395973
C -3.796768 2.825571 0.650800
C -4.161890 3.581471 -1.598781
H -4.099309 1.437368 -2.203267
H -5.552902 1.922471 -1.281399
C -3.520449 4.011055 -0.267081
H -4.781050 2.924188 1.149240
H -3.030884 2.665173 1.421715
H -5.083299 4.142062 -1.810645
H -3.479203 3.746944 -2.444302
H -3.931701 4.950695 0.127168
H -2.435330 4.147862 -0.385614

κ*O*-Na(THF)$_4$

E = –3591.10906578

O 0.003687 0.168436 0.129064
O -2.581856 -0.848872 2.023296
O -3.420934 1.801500 0.003838
O -1.729642 -1.806483 -1.777028
S 0.683766 1.463123 0.477303
C -1.965059 -2.036010 2.503300
C -2.564832 0.169059 3.024854
C -2.869735 3.123536 0.045300
C -4.733591 1.797350 0.550371
C -2.118892 -3.145878 -1.505179
C -0.517409 -1.786451 -2.538650
N 0.236220 2.282537 1.709976
C 2.347123 1.089258 0.503261
C 0.297042 2.577431 -0.862331
H -1.349790 -2.466946 1.697735
H -2.741745 -2.773587 2.780096
C -1.152621 -1.608002 3.711918
C -2.018024 -0.491023 4.284579
H -3.586303 0.558992 3.159278
H -1.921510 0.994738 2.675836
H -2.504883 3.392680 -0.957914
H -2.006314 3.117767 0.730537
C -3.990694 4.028437 0.536864
C -4.827748 3.068604 1.374285
H -4.868425 0.874955 1.132196
H -5.481241 1.796353 -0.265234
H -2.920032 -3.454705 -2.203934
H -2.526303 -3.196314 -0.484560
C -0.863600 -3.973462 -1.706453
C -0.203704 -3.239171 -2.867612
H 0.276467 -1.322402 -1.932735
H -0.669011 -1.167720 -3.437907
C 0.749797 1.885417 3.004196
Cl 3.346162 2.529901 0.736941
P 3.030660 -0.160483 -0.542757
C -0.069777 2.035464 -2.092936
C 0.431865 3.954610 -0.688360
H -0.179793 -1.210082 3.384514
H -0.967133 -2.430062 4.417288
H -2.836653 -0.914716 4.887638
H -1.460975 0.213261 4.918209

H -4.584534 4.409799 -0.309404
H -3.613574 4.893122 1.100864
H -4.364259 2.916447 2.362664
H -5.863879 3.401270 1.529330
H -1.077041 -5.031864 -1.913507
H -0.225082 -3.917823 -0.811080
H -0.668428 -3.536310 -3.821577
H 0.877341 -3.418544 -2.945219
H 1.838951 2.048832 3.096494
H 0.553672 0.830641 3.271824
H 0.257565 2.504544 3.769956
S 2.852897 -0.064235 -2.523485
C 4.797645 -0.160301 -0.045784
C 2.478964 -1.789937 0.090554
H -0.136848 0.953927 -2.205705
C -0.341890 2.892537 -3.157654
C 0.162834 4.801889 -1.760621
H 0.713685 4.339463 0.291940
C 5.142874 -0.301880 1.304072
C 5.797237 -0.046851 -1.010638
C 2.703464 -2.917821 -0.704738
C 1.957887 -1.943259 1.378025
H -0.640742 2.477178 -4.122933
C -0.229480 4.273481 -2.992305
H 0.254317 5.883117 -1.632770
H 4.357844 -0.383148 2.060081
C 6.482774 -0.331158 1.679180
C 7.140284 -0.074929 -0.632009
H 5.501507 0.061802 -2.058303
H 3.101240 -2.781460 -1.714045
C 2.416576 -4.189908 -0.213509
C 1.661534 -3.216261 1.863447
H 1.786379 -1.053072 1.986198
H -0.445092 4.942504 -3.828857
H 6.749932 -0.441492 2.733048
C 7.483949 -0.218013 0.711062
H 7.920897 0.015658 -1.391443
H 2.596050 -5.067095 -0.839951
C 1.893505 -4.341321 1.071410
H 1.250332 -3.331200 2.869345
H 8.535487 -0.241316 1.007915
H 1.664526 -5.338341 1.455749
Na -2.249221 -0.202150 -0.173221
O -4.377933 -1.147817 -0.540025
C -5.181079 -1.925090 0.337909
C -4.962044 -1.084699 -1.836345
C -6.505675 -2.116997 -0.383365
H -4.684231 -2.894733 0.528357
H -5.267241 -1.401136 1.301799
C -6.059078 -2.137451 -1.841487
H -5.376576 -0.072873 -2.000218
H -4.171261 -1.258722 -2.581887
H -7.169893 -1.257008 -0.199438
H -7.033738 -3.027940 -0.068382
H -6.863594 -1.910646 -2.554924
H -5.642550 -3.124501 -2.099931

κ*S*,κ*Cl*-Na(THF)$_4$

E = –3591.10150089

Cl 0.103141 -0.080683 1.997141
S -0.352419 -0.666349 -1.694672

O -2.908743 -1.795161 1.324029
C 1.455159 0.101357 0.886099
P 1.283694 -0.881324 -0.555229
C -3.397992 -1.852959 2.649723
C -2.149061 -2.974832 1.113684
C 2.804914 -0.645515 -1.536287
C 1.403553 -2.605955 0.043285
H -4.270922 -2.533586 2.699031
H -3.726815 -0.843670 2.933084
C -2.233166 -2.400581 3.464798
C -1.510541 -3.322196 2.466846
H -1.416924 -2.761066 0.323388
H -2.816505 -3.785433 0.765958
C 3.922896 1.385440 0.713125
C 4.002691 -1.254795 -1.144557
C 2.769804 0.147556 -2.684955
C 2.039066 -2.888979 1.256824
C 0.900056 -3.649661 -0.739061
H -1.572139 -1.578264 3.776064
H -2.569004 -2.922063 4.372197
H -0.430952 -3.124979 2.452561
H -1.652441 -4.385759 2.706169
C 4.709687 2.005564 -0.250633
C 4.491249 0.624504 1.734357
H 4.035741 -1.874757 -0.246719
C 5.157399 -1.067758 -1.898916
C 3.928909 0.336868 -3.434279
H 1.825441 0.609986 -2.978984
H 2.415342 -2.058407 1.859592
C 2.167320 -4.210055 1.684370
C 1.032259 -4.968500 -0.310190
H 0.389811 -3.411131 -1.676088
H 4.224288 2.588320 -1.034457
C 6.094305 1.836143 -0.207423
C 5.871818 0.457471 1.768527
H 3.840593 0.160687 2.479323
H 6.090697 -1.541931 -1.587400
C 5.121995 -0.270559 -3.043252
H 3.899501 0.961267 -4.330496
H 2.656329 -4.427352 2.637010
C 1.664954 -5.249854 0.902192
H 0.636121 -5.781759 -0.923113
H 6.721240 2.305650 -0.969251
C 6.674063 1.061433 0.795101
H 6.327938 -0.146735 2.556531
H 6.029826 -0.122053 -3.633271
H 1.762820 -6.284506 1.239939
H 7.757866 0.924692 0.821601
S 2.140483 1.669293 0.736627
Na -2.426053 0.153028 0.168048
N 1.775748 2.395519 2.056486
O 1.888336 2.344095 -0.570116
C 2.341000 3.720048 2.196716
H 2.071076 4.411960 1.375155
H 1.957851 4.161231 3.129659
H 3.448745 3.723826 2.269985
O -4.573578 0.834878 1.035850
C -4.963769 2.203378 1.000160
C -5.685588 -0.002303 0.710260
C -6.231717 2.236651 0.167017
H -5.155749 2.565740 2.027954
H -4.132808 2.781338 0.573413

C -6.888287 0.923925 0.577613
H -5.460593 -0.514935 -0.238381
H -5.813816 -0.761576 1.497118
H -5.980537 2.223475 -0.906285
H -6.850875 3.123496 0.362809
H -7.626353 0.552094 -0.147140
H -7.397615 1.043224 1.547387
O -1.865056 2.383338 0.071626
C -1.593913 3.019405 -1.176289
C -1.445938 3.212156 1.159860
C -0.769633 4.252677 -0.840882
H -1.061486 2.303763 -1.822386
H -2.554305 3.283867 -1.657232
C -1.269194 4.595829 0.558871
H -2.217496 3.167680 1.945397
H -0.495493 2.827405 1.566118
H -0.905355 5.063494 -1.570979
H 0.293335 3.971865 -0.802896
H -2.233326 5.129747 0.512040
H -0.564417 5.212793 1.134812
O -3.629378 -0.439925 -1.775191
C -3.664125 0.421493 -2.907833
C -3.640116 -1.806073 -2.185280
C -4.083919 -0.456472 -4.074420
H -4.360516 1.249376 -2.701098
H -2.658061 0.847465 -3.072379
C -3.440747 -1.785124 -3.692381
H -2.837963 -2.340561 -1.655351
H -4.609692 -2.256738 -1.904950
H -3.745169 -0.063806 -5.043415
H -5.181174 -0.554045 -4.108737
H -2.364029 -1.767777 -3.925130
H -3.888501 -2.655281 -4.192642

κ*O*,κ*N*-Na(THF)$_3$

E = –3358.81612601

O -1.542569 2.232693 -2.281515
O -4.301498 -0.229438 -0.733299
O -3.156829 2.703886 0.855542
S -0.271316 -0.964881 -0.073907
C -0.142091 2.358945 -2.028773
C -1.766311 1.697177 -3.585884
C -5.360656 -0.411822 0.206429
C -4.555477 -0.965151 -1.930057
C -2.394533 3.898363 0.735535
C -3.271729 2.358495 2.236950
C 1.377723 -1.118947 -0.422990
C -0.885734 -2.522250 0.588683
H 0.071800 1.964678 -1.023805
H 0.135518 3.429125 -2.052542
C 0.548053 1.570592 -3.128483
C -0.420656 1.750630 -4.292540
H -2.548412 2.290656 -4.084426
H -2.122223 0.656025 -3.491402
H -5.702096 0.573528 0.559926
H -4.970387 -0.972061 1.074887
C -6.438054 -1.195392 -0.530754
C -5.619470 -1.980105 -1.551319
H -3.606603 -1.409349 -2.266794
H -4.918682 -0.276752 -2.714790
H -3.053552 4.783013 0.827408

H -1.938760 3.910157 -0.264968
C -1.407741 3.824141 1.883982
C -2.291975 3.261765 2.995621
H -3.024833 1.289850 2.340854
H -4.313195 2.501569 2.570431
Cl 1.702604 -2.499932 -1.485481
P 2.706337 -0.685481 0.656021
C -0.038706 -3.387092 1.275961
C -2.256395 -2.767938 0.492994
H 0.608260 0.512941 -2.830674
H 1.561277 1.939333 -3.343202
H -0.274059 2.732997 -4.769775
H -0.323331 0.977708 -5.067763
H -7.128862 -0.508607 -1.044949
H -7.030820 -1.830675 0.141885
H -5.156761 -2.863679 -1.082223
H -6.205148 -2.323319 -2.415255
H -0.958484 4.795442 2.134138
H -0.595232 3.124182 1.633785
H -2.832969 4.076185 3.501251
H -1.726430 2.714699 3.762835
S 3.134626 -1.839535 2.224863
C 4.142040 -0.573403 -0.477975
C 2.474795 1.058078 1.177241
H 1.030243 -3.162953 1.358429
C -0.578262 -4.530824 1.865703
C -2.784669 -3.909129 1.091467
H -2.895433 -2.059350 -0.037247
C 3.981464 -0.181280 -1.812640
C 5.422643 -0.812440 0.025636
C 2.485844 1.387971 2.532732
C 2.386763 2.074004 0.219064
H 0.077532 -5.219967 2.403038
C -1.944965 -4.793009 1.773462
H -3.856574 -4.111528 1.024083
H 2.976709 -0.023178 -2.210186
C 5.098322 -0.019063 -2.629083
C 6.538469 -0.647488 -0.794112
H 5.527555 -1.136492 1.064697
H 2.592870 0.585148 3.267162
C 2.372707 2.720527 2.930351
C 2.281660 3.403389 0.616230
H 2.408502 1.822950 -0.841928
H -2.360658 -5.690309 2.238172
H 4.967995 0.281301 -3.671619
C 6.377980 -0.248296 -2.120702
H 7.537958 -0.835241 -0.394443
H 2.374129 2.970682 3.994026
C 2.268004 3.728908 1.974297
H 2.211776 4.190909 -0.138020
H 7.252316 -0.122238 -2.764036
H 2.182255 4.772875 2.285494
N -0.588934 0.178775 0.937730
C -0.459811 -0.077466 2.360132
H 0.469099 -0.601138 2.646311
H -0.448230 0.890599 2.880762
H -1.306345 -0.666574 2.762663
O -1.050466 -0.756064 -1.329448
Na -2.394721 1.009747 -0.516536

κ*N*-Na(THF)$_3$

E = –3358.81680171

O 3.932603 -2.588153 -0.539881
O 3.995524 0.966938 -0.642634
O 2.991560 -0.217246 2.771552
S 0.094940 -0.520340 -0.970295
C 4.190998 -3.839882 0.083535
C 3.455685 -2.788386 -1.878395
C 4.470284 2.225610 -0.170876
C 3.711718 1.027918 -2.042949
C 3.772181 0.732098 3.473851
C 1.756867 -0.347899 3.474366
C -1.583466 -0.849425 -0.893308
C 0.370390 1.238787 -0.799328
H 3.937525 -3.757148 1.152161
H 5.266159 -4.087724 0.003568
C 3.340040 -4.840457 -0.675946
C 3.439125 -4.297924 -2.098403
H 4.125378 -2.265844 -2.580315
H 2.452796 -2.338261 -1.961230
H 5.375160 2.063964 0.436160
H 3.696081 2.683192 0.469969
C 4.722408 3.065151 -1.413980
C 3.683867 2.507024 -2.380456
H 2.756917 0.514032 -2.233061
H 4.511163 0.501737 -2.595964
H 4.582088 1.071824 2.812401
H 4.224676 0.264811 4.369791
C 2.778792 1.816912 3.860030
C 1.492360 1.018938 4.128249
H 1.853482 -1.143626 4.234226
H 0.984471 -0.636519 2.746659
Cl -2.413093 -0.772040 -2.456732
P -2.586971 -0.262867 0.441861
C 0.789177 1.807504 0.401291
C 0.016201 2.035226 -1.891437
H 2.299647 -4.799853 -0.317772
H 3.700253 -5.873562 -0.572764
H 4.377775 -4.633342 -2.566997
H 2.608700 -4.614621 -2.744562
H 5.740277 2.890344 -1.797809
H 4.611495 4.141679 -1.222255
H 2.689717 2.923243 -2.158843
H 3.912765 2.704135 -3.437037
H 2.643169 2.509195 3.015130
H 3.112127 2.407482 4.724688
H 0.607320 1.504227 3.692645
H 1.305605 0.906002 5.205561
S -1.720924 -0.141862 2.218520
C -3.309503 1.352123 -0.039126
C -4.016134 -1.395569 0.495367
H 1.029208 1.153501 1.238349
C 0.867594 3.196447 0.502454
C 0.082899 3.420827 -1.772842
H -0.312576 1.563209 -2.818819
C -4.354519 1.439762 -0.968771
C -2.732493 2.523804 0.460160
C -5.167758 -1.014194 1.193928
C -3.933368 -2.671843 -0.066175
H 1.195238 3.652810 1.439860
C 0.512072 4.003194 -0.578461
H -0.199853 4.050091 -2.619690

H -4.817111 0.530991 -1.358765
C -4.810448 2.687453 -1.391549
C -3.183676 3.768804 0.028360
H -1.927286 2.442413 1.194195
H -5.227161 -0.019492 1.644148
C -6.232705 -1.902470 1.319429
C -5.001186 -3.559672 0.062364
H -3.025222 -2.952508 -0.605753
H 0.565295 5.090717 -0.489574
H -5.630016 2.748945 -2.111822
C -4.222866 3.853087 -0.898620
H -2.720226 4.677811 0.419565
H -7.131301 -1.601131 1.863247
C -6.150774 -3.176337 0.752658
H -4.935238 -4.556426 -0.380713
H -4.580520 4.829686 -1.234534
H -6.987340 -3.872503 0.851385
N 0.856129 -1.123903 0.242059
C 0.517124 -2.484224 0.621356
H -0.412098 -2.522666 1.215184
H 0.409151 -3.177398 -0.232473
H 1.325799 -2.874589 1.259949
O 0.537070 -0.861399 -2.350658
Na 3.143374 -0.753095 0.566794

κ*S*,κ*Cl*-Na(THF)$_3$

E = –3358.81120355

Cl -0.349078 0.298503 -1.823836
S -0.773358 0.359534 2.131482
O -3.423767 1.757857 -0.893308
C 0.917361 0.005358 -0.644473
P 0.737259 0.833033 0.886151
C -4.397759 1.578728 -1.904884
C -2.711775 2.934546 -1.243612
C 2.344461 0.683493 1.736988
C 0.607675 2.602195 0.436556
H -5.229277 2.294235 -1.753418
H -4.800284 0.560333 -1.817298
C -3.652111 1.871266 -3.203024
C -2.604199 2.917692 -2.774961
H -1.743816 2.914092 -0.727858
H -3.276111 3.818818 -0.894057
C 3.493748 -1.079672 -0.848180
C 3.456253 1.381829 1.251649
C 2.468070 -0.126104 2.867211
C 1.136422 3.071501 -0.771620
C -0.014981 3.493613 1.315698
H -3.161320 0.956449 -3.568158
H -4.322406 2.228803 -3.997151
H -1.594653 2.623478 -3.093812
H -2.809676 3.911794 -3.196580
C 4.421028 -1.662454 0.007533
C 3.890447 -0.217471 -1.870311
H 3.365597 2.011193 0.364406
C 4.684089 1.269211 1.896994
C 3.701324 -0.243118 3.505430
H 1.588663 -0.657625 3.237535
H 1.610449 2.364668 -1.456722
C 1.030020 4.421938 -1.099786
C -0.109082 4.844965 0.989311
H -0.444271 3.107799 2.243836

H 4.067613 -2.325435 0.798290
C 5.773592 -1.354980 -0.147065
C 5.240150 0.086472 -2.016221
H 3.135111 0.215542 -2.531015
H 5.549654 1.813385 1.512972
C 4.808515 0.454251 3.022591
H 3.796913 -0.881195 4.387236
H 1.431402 4.782285 -2.049949
C 0.407931 5.309369 -0.220810
H -0.597897 5.537319 1.678814
H 6.509885 -1.794946 0.529824
C 6.182351 -0.480357 -1.151822
H 5.562554 0.768949 -2.806353
H 5.774149 0.362414 3.525973
H 0.322494 6.367006 -0.481939
H 7.241289 -0.236198 -1.265893
S 1.743124 -1.496366 -0.707541
Na -2.500101 -0.187119 -0.003932
N 1.290103 -2.155283 -2.031516
O 1.678075 -2.246434 0.579052
C 2.014177 -3.341673 -2.430777
H 1.909845 -4.189135 -1.725131
H 1.612595 -3.680951 -3.397952
H 3.101282 -3.173003 -2.574254
O -4.675108 -0.568603 0.669828
C -5.162774 -1.881026 0.917578
C -5.155997 0.326076 1.675621
C -5.356301 -1.933573 2.420458
H -6.122339 -2.036303 0.388043
H -4.421573 -2.589826 0.523039
C -5.905839 -0.535609 2.693019
H -4.289005 0.837973 2.125682
H -5.797428 1.093124 1.212303
H -4.383054 -2.075461 2.918196
H -6.029607 -2.740134 2.743121
H -5.747052 -0.196072 3.725981
H -6.988964 -0.511624 2.495506
O -2.130733 -2.428891 -0.260422
C -1.545516 -3.218318 0.780116
C -1.951813 -3.062366 -1.529352
C -0.856339 -4.380327 0.081169
H -0.842341 -2.591587 1.347918
H -2.352304 -3.557987 1.455349
C -1.661725 -4.517001 -1.206228
H -2.868790 -2.912197 -2.121144
H -1.092685 -2.597664 -2.042218
H -0.843137 -5.293009 0.693876
H 0.180144 -4.091303 -0.142742
H -2.597108 -5.073162 -1.027713
H -1.111121 -5.022448 -2.012777

κ*O*-Na(THF)$_2$

E = –3126.52827985

O -0.729526 0.076672 -0.716263
O -1.954229 3.041072 0.132853
O -3.995902 0.341754 -1.199551
S -0.355390 -1.375008 -0.832878
C -2.080259 4.460016 0.157780
C -1.554111 2.548769 1.417433
C -4.691660 0.478269 0.036966
C -4.151990 -0.985250 -1.717606

N -0.719568 -2.180619 -2.099539
C 1.304880 -1.385450 -0.463538
C -1.316235 -2.206653 0.418078
H -3.002137 4.746697 -0.372062
H -1.221024 4.911418 -0.370736
C -2.082353 4.843337 1.628532
C -1.166615 3.777777 2.220712
H -0.732830 1.828468 1.289473
H -2.411322 2.025926 1.879474
H -5.184244 1.462527 0.058915
H -3.970366 0.432621 0.873184
C -5.656004 -0.693320 0.090482
C -4.853511 -1.775487 -0.623226
H -3.160308 -1.393893 -1.971037
H -4.763489 -0.933799 -2.636171
C 0.087497 -1.956454 -3.279841
Cl 1.965066 -3.007701 -0.273715
P 1.997572 -0.089538 0.512574
C -1.713214 -1.468609 1.533069
C -1.599360 -3.567180 0.307901
H -3.097765 4.754212 2.047265
H -1.733470 5.871737 1.797710
H -0.111586 4.035393 2.038820
H -1.297598 3.631791 3.301747
H -6.577448 -0.458859 -0.466668
H -5.936213 -0.961527 1.118927
H -4.113659 -2.215644 0.062222
H -5.472146 -2.590105 -1.024916
H 1.172960 -2.030640 -3.081555
H -0.092542 -0.974868 -3.760190
H -0.166011 -2.726806 -4.023796
S 1.412196 0.206250 2.394419
C 3.797982 -0.431637 0.436087
C 1.841348 1.452321 -0.473422
H -1.449058 -0.413412 1.600577
C -2.436788 -2.103400 2.540347
C -2.321615 -4.191437 1.323246
H -1.272499 -4.105424 -0.582109
C 4.402020 -0.752786 -0.786279
C 4.577636 -0.326206 1.587904
C 1.804743 2.680342 0.188205
C 1.797337 1.417627 -1.871506
H -2.761331 -1.533126 3.413850
C -2.743923 -3.461020 2.435385
H -2.559517 -5.254723 1.243327
H 3.789059 -0.850068 -1.685214
C 5.776964 -0.960564 -0.849713
C 5.955623 -0.535863 1.521982
H 4.083701 -0.080101 2.532387
H 1.833130 2.686575 1.281396
C 1.707774 3.867277 -0.538341
C 1.699944 2.603591 -2.596108
H 1.809904 0.451948 -2.381416
H -3.313756 -3.953851 3.226707
H 6.244530 -1.213397 -1.804471
C 6.556300 -0.851255 0.304429
H 6.561428 -0.453407 2.427752
H 1.673921 4.824804 -0.012343
C 1.651333 3.831541 -1.931216
H 1.658818 2.570209 -3.687910
H 7.635422 -1.015989 0.252304
H 1.572121 4.760619 -2.501287

Na -2.091046 1.552489 -1.568205

κ*S*,κ*Cl*-Na(THF)$_2$

E = –3126.52185991

O 0.133909 3.404879 -1.152821
O 3.907815 1.838707 0.342756
S 0.654852 -0.902872 -1.159903
C -0.648474 3.158319 -2.323226
C -0.682452 3.927526 -0.103556
C 5.152890 2.229819 -0.222467
C 4.126481 0.889341 1.394912
C -0.345802 -0.344876 0.089831
C 2.098321 -1.733772 -0.464038
H -0.419601 2.145609 -2.690497
H -0.365017 3.887331 -3.103136
C -2.096463 3.337190 -1.896152
C -1.973582 4.362670 -0.774910
H -0.141943 4.752273 0.385863
H -0.861424 3.135027 0.644272
H 5.534534 3.135358 0.285929
H 4.990924 2.473554 -1.283816
C 6.068558 1.044378 0.009687
C 5.625573 0.592449 1.398501
H 3.523095 -0.004625 1.177681
H 3.777428 1.314978 2.349402
Cl 0.519500 0.668225 1.248903
P -1.750875 -1.244692 0.670352
C 1.934777 -2.522472 0.673936
C 3.332623 -1.634954 -1.106396
H -2.496850 2.389910 -1.505433
H -2.743911 3.660488 -2.722939
H -1.871281 5.378233 -1.189633
H -2.828458 4.362303 -0.083977
H 7.134039 1.307457 -0.046636
H 5.865043 0.261475 -0.737160
H 6.138223 1.185629 2.171499
H 5.832872 -0.468766 1.593906
S -1.571510 -3.055276 1.480931
C -2.432779 -0.062737 1.900684
C -3.025457 -1.228722 -0.640557
H 0.948834 -2.602962 1.146995
C 3.038595 -3.196644 1.192356
C 4.425466 -2.330924 -0.590425
H 3.429919 -1.005706 -1.992674
C -2.981133 1.155084 1.482600
C -2.381685 -0.368655 3.260701
C -4.149781 -2.043639 -0.482580
C -2.936411 -0.374983 -1.743181
H 2.925484 -3.803567 2.093835
C 4.281521 -3.103228 0.562483
H 5.395861 -2.265458 -1.088471
H -3.032363 1.392571 0.417922
C -3.468026 2.060791 2.421868
C -2.865727 0.541179 4.200751
H -1.963247 -1.332561 3.563771
H -4.195199 -2.727677 0.369013
C -5.188310 -1.988770 -1.411242
C -3.975180 -0.322067 -2.670181
H -2.033911 0.223145 -1.873960
H 5.142328 -3.637673 0.971448

H -3.901097 3.007899 2.090384
C -3.407053 1.756537 3.783403
H -2.821690 0.297485 5.265117
H -6.065985 -2.626797 -1.281959
C -5.104497 -1.125109 -2.503690
H -3.898124 0.344965 -3.532540
H -3.789400 2.467702 4.519793
H -5.917853 -1.084027 -3.232473
N -0.235755 -1.894356 -1.924043
C 0.357402 -2.672098 -2.984959
H 1.196515 -3.314154 -2.648627
H -0.414811 -3.341535 -3.392223
H 0.731210 -2.057463 -3.826039
O 1.293764 0.249338 -1.892141
Na 1.962183 2.099800 -0.806973

κ*O*,κ*S*-Na(THF)$_2$

E = –3126.52723608

Cl 1.664747 -0.274585 -1.952130
S 0.189335 -1.762467 1.644784
O -0.836189 -3.639971 -1.507270
C 0.828114 0.533172 -0.621995
P 1.123599 -0.103990 0.993591
C -0.315106 -3.246446 -2.783143
C 0.106728 -4.472387 -0.842810
C 0.907842 1.267537 2.181307
C 2.934042 -0.387358 0.993408
C -0.599320 2.144030 -2.376359
H -0.940995 -3.676803 -3.582699
H -0.369932 -2.148454 -2.841941
C 1.127434 -3.752488 -2.836569
C 1.451342 -4.013957 -1.368389
H -0.011157 -4.327381 0.240967
H -0.085413 -5.535011 -1.086865
C 1.214954 2.580241 1.807817
C 0.497685 0.992866 3.486335
C 3.797180 0.588925 0.479534
C 3.458954 -1.557536 1.543133
C -0.230818 3.437709 -2.006804
C -0.831701 1.804023 -3.705694
H 1.802118 -3.019232 -3.299931
H 1.192450 -4.686916 -3.415814
H 1.736718 -3.078426 -0.861748
H 2.249840 -4.754706 -1.219646
H 1.508466 2.786941 0.776621
C 1.114627 3.609097 2.739776
C 0.396580 2.026872 4.417712
H 0.247523 -0.036279 3.757400
H 3.386455 1.495871 0.030781
C 5.174297 0.391733 0.523447
C 4.839942 -1.752139 1.585316
H 2.768286 -2.311028 1.931450
H -0.067265 3.668423 -0.951419
C -0.088445 4.408045 -2.994459
C -0.679767 2.782206 -4.688823
H -1.131507 0.784473 -3.951954
H 1.348677 4.634511 2.443128
C 0.705565 3.334595 4.046844
H 0.071794 1.807855 5.437872
H 5.844227 1.153814 0.118168
C 5.698105 -0.778503 1.077666

H 5.245235 -2.670889 2.016152
H 0.195224 5.426518 -2.719470
C -0.308958 4.079080 -4.334493
H -0.853482 2.529219 -5.737357
H 0.623912 4.144654 4.775810
H 6.779716 -0.931132 1.109399
H -0.193071 4.842387 -5.107564
S -0.775088 0.923110 -1.070345
O -3.584769 -1.390390 1.048212
C -4.975506 -1.488105 0.754098
C -3.377704 -0.686618 2.277908
C -5.691004 -1.116960 2.042262
H -5.228537 -0.784800 -0.060595
H -5.196063 -2.509304 0.406335
C -4.730065 -0.093606 2.637369
H -3.031549 -1.401303 3.044719
H -2.586562 0.061092 2.123981
H -5.773975 -1.995642 2.701923
H -6.702872 -0.725496 1.866962
H -4.852480 0.049324 3.720084
H -4.862765 0.884410 2.148165
O -1.519092 -0.195154 -1.751077
N -1.392591 1.542258 0.201561
C -2.756129 2.013536 0.089471
H -3.055757 2.423972 1.065284
H -2.880656 2.826480 -0.653713
H -3.479759 1.217615 -0.167851
Na -1.838559 -1.942211 -0.312576

κ*O*,κ*N*-Na(THF)

E = –2894.22919197

O -0.797856 1.388843 -0.274606
O -4.113224 0.901925 0.273409
S 0.413914 1.567674 0.579262
C -5.393689 0.437191 0.682015
C -3.865415 0.545797 -1.094749
N -0.057946 2.015937 2.002279
C 1.361404 0.158486 0.505597
C 1.371924 2.886926 -0.172135
H -5.839506 1.182932 1.357694
H -5.286489 -0.514663 1.235071
C -6.169880 0.233648 -0.606302
C -5.071999 -0.270079 -1.537420
H -2.915019 -0.004313 -1.156134
H -3.763327 1.475225 -1.680330
C 0.824529 1.813212 3.131668
Cl 3.051742 0.393107 0.933489
P 0.962310 -1.109417 -0.650796
C 1.991951 2.642374 -1.397959
C 1.437246 4.130679 0.448825
H -6.574286 1.194381 -0.963763
H -7.007475 -0.468766 -0.492113
H -4.889752 -1.341646 -1.366049
H -5.301298 -0.131854 -2.603133
H 1.704310 2.485323 3.136646
H 1.205016 0.776511 3.196271
H 0.255823 2.015800 4.051443
S 1.102143 -0.807372 -2.613910
C 2.061777 -2.476529 -0.116363
C -0.701423 -1.727008 -0.191114
H 1.923936 1.648125 -1.856195

C 2.688972 3.678766 -2.012185
C 2.143934 5.159952 -0.174156
H 0.932713 4.277239 1.406399
C 2.087010 -2.868480 1.227905
C 2.853961 -3.143245 -1.050179
C -1.428214 -2.464242 -1.130076
C -1.220687 -1.537801 1.093922
H 3.179072 3.506616 -2.973354
C 2.766260 4.933667 -1.401485
H 2.207962 6.141433 0.301387
H 1.474851 -2.336399 1.959894
C 2.898130 -3.926188 1.628192
C 3.668556 -4.201723 -0.645760
H 2.818025 -2.816490 -2.093450
H -1.020256 -2.584822 -2.137259
C -2.658108 -3.020624 -0.782616
C -2.459465 -2.079971 1.432268
H -0.651160 -0.947863 1.816386
H 3.320036 5.740866 -1.887007
H 2.916423 -4.230196 2.677622
C 3.690291 -4.594524 0.691319
H 4.289191 -4.720791 -1.380266
H -3.218068 -3.601456 -1.519623
C -3.176717 -2.827923 0.498294
H -2.866567 -1.919171 2.433832
H 4.327700 -5.423777 1.008130
H -4.145167 -3.256811 0.767490
Na -2.377790 1.846936 1.366907

6.4.4 Coordinates of the dimeric structures of 1-Na

***κS*,*κCl*-Na$_2$(THF)$_2$**

E = –5788.49203279

Cl 1.476145 1.515779 1.699285
Cl -1.483862 -1.405375 -1.770364
S 1.346507 0.974221 -1.888525
S -1.224298 -0.976798 1.818187
C 3.475709 -2.168549 1.699761
C 2.589557 -2.918383 2.475411
C 2.432961 -4.276524 2.206188
C 3.163157 -4.876419 1.179250
C 4.061645 -4.121904 0.425194
C 4.225452 -2.762022 0.684280
O 0.770085 -3.548715 -0.706280
C -3.648613 2.174500 -1.632098
C -2.863168 2.963044 -2.473493
C -2.744883 4.326557 -2.212619
C -3.415894 4.893235 -1.128348
C -4.220240 4.099803 -0.310256
C -4.344524 2.734401 -0.560941
O -0.769125 3.577669 0.467190
C 2.569203 0.369816 0.894019
C -2.594327 -0.332168 -0.888842
P 2.987262 0.962526 -0.718991
P -2.918621 -0.968396 0.729050
H 2.038665 -2.427330 3.279685
H 1.735666 -4.868899 2.803202
H 3.033589 -5.940612 0.968294
H 4.643901 -4.592080 -0.370762

H 4.931359 -2.162685 0.107236
C 1.159126 -4.011961 -1.997420
C -0.281009 -4.358696 -0.180276
H -2.358878 2.496356 -3.321530
H -2.125741 4.949826 -2.862095
H -3.316644 5.961982 -0.923903
H -4.760165 4.544009 0.529355
H -4.978975 2.105815 0.065419
C -1.096941 4.086059 1.758286
C 0.240907 4.379698 -0.144883
C 4.240740 -0.122131 -1.474084
C 3.709405 2.634925 -0.638131
C -4.141300 0.085901 1.570287
C -3.614485 -2.652585 0.659151
H 2.250308 -4.161903 -2.010833
H 0.907512 -3.237552 -2.744462
C 0.370652 -5.293072 -2.236974
C -0.873216 -5.064021 -1.384260
H -0.990998 -3.706849 0.348475
H 0.140507 -5.077751 0.545117
H -2.188712 4.213490 1.826055
H -0.787290 3.349164 2.521581
C -0.325175 5.391171 1.901257
C 0.878446 5.147784 0.996252
H 0.937322 3.715215 -0.676847
H -0.228893 5.058151 -0.879844
C 5.605606 0.091297 -1.245844
C 3.828753 -1.178226 -2.293032
C 4.395460 3.037475 0.513099
C 3.550451 3.521111 -1.709092
C -5.513178 -0.161915 1.443933
C -3.694886 1.156009 2.352413
C -4.304964 -3.074998 -0.481736
C -3.418455 -3.533313 1.729215
H 0.153807 -5.459287 -3.301391
H 0.932606 -6.164550 -1.864492
H -1.580640 -4.393467 -1.897282
H -1.405163 -5.987761 -1.117065
H -0.058228 5.608845 2.944824
H -0.924018 6.234021 1.520287
H 1.624134 4.513163 1.500735
H 1.377294 6.068659 0.663479
H 5.928895 0.916402 -0.611422
C 6.543794 -0.770626 -1.808405
C 4.770993 -2.042227 -2.848356
H 2.765580 -1.311688 -2.505468
H 4.527549 2.325145 1.332806
C 4.918035 4.328071 0.587372
C 4.079770 4.807096 -1.629778
H 2.992240 3.200569 -2.592381
H -5.860773 -0.997157 0.835723
C -6.428099 0.679069 2.072838
C -4.614511 1.999309 2.973452
H -2.622631 1.316607 2.485133
H -4.469856 -2.367124 -1.298354
C -4.793104 -4.379645 -0.548412
C -3.915195 -4.832377 1.658822
H -2.854628 -3.198637 2.603592
H 7.607866 -0.604052 -1.626006
C 6.128469 -1.843655 -2.600311
H 4.441110 -2.869736 -3.480699
H 5.449439 4.645232 1.487856

C 4.760329 5.212258 -0.480024
H 3.954171 5.499037 -2.465955
H -7.498425 0.485976 1.970768
C -5.981433 1.765716 2.828372
H -4.259207 2.838656 3.575654
H -5.327010 -4.711989 -1.441898
C -4.598628 -5.257628 0.517740
H -3.760990 -5.519287 2.494355
H 6.868869 -2.519386 -3.035196
H 5.167324 6.224404 -0.415487
H -6.703929 2.425231 3.315176
H -4.979124 -6.280417 0.459487
S -3.760738 0.397654 -1.948138
O -3.290568 0.254949 -3.345537
N -5.153673 -0.109250 -1.515748
C -6.320828 0.500777 -2.116192
H -6.500672 0.148643 -3.148277
H -7.202492 0.225482 -1.516518
H -6.285247 1.607145 -2.150880
S 3.640598 -0.396431 2.024388
O 3.085947 -0.233058 3.387809
N 5.078813 0.053370 1.687258
C 6.174877 -0.594772 2.376131
H 7.108724 -0.353133 1.844951
H 6.287815 -0.245630 3.418673
H 6.097328 -1.699100 2.409087
Na -0.817493 1.393176 -0.112044
Na 0.871027 -1.406401 0.008896

κ*N*,κ*S*,κ*Cl*-$Na_2(THF)_2$

E = –5788.48094950

Cl -1.675277 1.505043 -1.918606
Cl 1.676549 -1.502744 1.920478
S -1.430462 0.161517 1.637814
S 1.430158 -0.160042 -1.635880
O -0.909680 -3.620635 0.583844
O 0.909487 3.620390 -0.581749
C -3.091662 0.785157 -1.119493
C 3.093221 -0.784639 1.120068
P -2.971820 0.979869 0.617143
P 2.971582 -0.979727 -0.616449
C -2.045555 -3.601239 1.430299
C 0.012383 -4.529962 1.161497
C 2.043521 3.600562 -1.430676
C -0.012677 4.531743 -1.156040
C -4.550803 0.482625 1.373262
C -2.902620 2.798523 0.829334
C 4.550475 -0.484621 -1.374176
C 2.900202 -2.798408 -0.827904
C -5.246100 -0.964239 -1.522090
H -2.658300 -4.507669 1.253736
H -2.641849 -2.715100 1.171868
C -1.459838 -3.583369 2.834917
C -0.168516 -4.408698 2.683667
H 1.016999 -4.261398 0.811277
H -0.216631 -5.553061 0.809775
C 5.248720 0.964209 1.519359
H 2.658016 4.505804 -1.254087
H 2.639021 2.713100 -1.174983
C 1.454671 3.585636 -2.833991
C 0.165220 4.413100 -2.678782

H -1.016929 4.263566 -0.804460
H 0.218028 5.553963 -0.802874
C -5.746429 1.058184 0.926409
C -4.563509 -0.423222 2.434866
C -3.563309 3.634803 -0.078609
C -2.215979 3.350896 1.913885
C 5.745974 -1.060580 -0.927491
C 4.563122 0.419926 -2.436886
C 3.561511 -3.635019 0.079294
C 2.211337 -3.350501 -1.911185
C -5.568147 -1.889241 -0.533992
C -6.230410 -0.305387 -2.255688
H -1.229823 -2.544169 3.115519
H -2.150153 -3.992426 3.586527
H 0.688380 -3.900049 3.146515
H -0.255738 -5.401191 3.149364
C 5.569654 1.888246 0.529980
C 6.233837 0.305911 2.252357
H 1.222193 2.547213 -3.115447
H 2.144047 3.994413 -3.586612
H -0.693466 3.907004 -3.141104
H 0.253572 5.406345 -3.142665
H -5.734715 1.760382 0.091162
C -6.947483 0.718262 1.537991
C -5.771982 -0.764561 3.040854
H -3.615945 -0.843769 2.782238
H -4.061617 3.189286 -0.943490
C -3.541446 5.014887 0.107513
C -2.193103 4.733600 2.092004
H -1.692925 2.686105 2.607371
H 5.734305 -1.761723 -0.091362
C 6.946850 -0.722322 -1.540338
C 5.771422 0.759571 -3.044173
H 3.615635 0.840825 -2.784055
H 4.061521 -3.189706 0.943296
C 3.538091 -5.015135 -0.106355
C 2.186831 -4.733250 -2.088773
H 1.687877 -2.685452 -2.604127
H -4.765524 -2.388155 0.011668
C -6.909759 -2.149929 -0.263877
C -7.568122 -0.563907 -1.970446
H -5.930212 0.386632 -3.044806
H 4.766402 2.386784 -0.015110
C 6.910961 2.148463 0.257936
C 7.571233 0.563983 1.965210
H 5.934505 -0.385315 3.042498
H -7.880105 1.161510 1.182674
C -6.961699 -0.193714 2.594817
H -5.780388 -1.474443 3.871384
H -4.051074 5.665892 -0.606875
C -2.858423 5.565643 1.192845
H -1.649946 5.160891 2.938138
H 7.879385 -1.165849 -1.185142
C 6.961016 0.188352 -2.598290
H 5.779781 1.468432 -3.875575
H 4.048242 -5.666383 0.607438
C 2.852820 -5.565614 -1.190416
H 1.641888 -5.160300 -2.933879
H -7.176825 -2.869694 0.512738
C -7.906245 -1.485828 -0.977707
H -8.353020 -0.053673 -2.533931
H 7.177172 2.867443 -0.519700

C 7.908240 1.484892 0.971162
H 8.356761 0.054182 2.528210
H -7.908082 -0.459086 3.072448
H -2.837781 6.649190 1.332659
H 7.907263 0.452414 -3.076913
H 2.830946 -6.649187 -1.329837
H -8.957666 -1.690156 -0.761306
H 8.959419 1.688857 0.753247
S -3.526329 -0.666289 -1.947513
S 3.529435 0.666955 1.947113
N -2.673788 -1.867239 -1.415066
O -3.529384 -0.410672 -3.398665
C -2.837419 -3.115534 -2.147404
H -2.184618 -3.866735 -1.677717
H -3.871288 -3.511789 -2.118168
H -2.552696 -3.024163 -3.210273
N 2.676608 1.867931 1.415272
O 3.534197 0.411812 3.398344
C 2.841643 3.116605 2.146604
H 3.875505 3.512725 2.115285
H 2.188123 3.867616 1.677616
H 2.558804 3.025907 3.210036
Na 0.620744 1.569178 0.395566
Na -0.619506 -1.568492 -0.391993

κ*O*,κ*N*,κ*S*-$Na_2(THF)_2$

E = –5788.51446562

Cl -4.372690 0.861597 -1.960439
Cl 4.373044 -0.861747 1.960266
S -1.448361 1.712795 0.985619
S 1.448430 -1.713059 -0.984561
O 0.408950 -2.674913 2.678363
O -0.409639 2.675426 -2.677266
C -3.561432 -0.057573 -0.706918
C 3.562058 0.057768 0.706760
P -3.199908 0.744015 0.810877
P 3.199949 -0.744172 -0.810747
C 1.420419 -2.505071 3.679834
C 0.641757 -3.876664 1.954264
C -1.420377 2.505424 -3.679433
C -0.643462 3.876826 -1.952902
C -3.371785 -0.479588 2.158986
C -4.597591 1.889482 1.062116
C 3.371172 0.479289 -2.159073
C 4.597665 -1.889512 -1.062400
C -3.392358 -2.678475 -1.724452
H 0.951080 -2.526497 4.677556
H 1.880377 -1.516106 3.521075
C 2.420222 -3.644131 3.478985
C 2.139422 -4.090231 2.046980
H 0.276971 -3.732327 0.926300
H 0.082070 -4.712806 2.415793
C 3.393212 2.678619 1.724269
H -0.950383 2.527154 -4.676838
H -1.880123 1.516286 -3.521127
C -2.420688 3.644104 -3.479025
C -2.141132 4.089842 -2.046662
H -0.279422 3.732260 -0.924706
H -0.083754 4.713349 -2.413711
C -4.157232 -1.623538 1.984993
C -2.725821 -0.256201 3.378545

C -5.910370 1.405649 0.995375
C -4.361903 3.233583 1.352345
C 4.156755 1.623237 -1.985670
C 2.724512 0.255767 -3.378240
C 5.910442 -1.405643 -0.995912
C 4.361945 -3.233578 -1.352771
C -3.292899 -3.844614 -0.968715
C -4.231126 -2.601057 -2.839013
H 3.456654 -3.310519 3.627133
H 2.222786 -4.468056 4.182885
H 2.645926 -3.426074 1.328931
H 2.442592 -5.126706 1.840953
C 3.293505 3.844808 0.968638
C 4.232419 2.601092 2.838483
H -3.456903 3.310228 -3.628099
H -2.222948 4.468319 -4.182501
H -2.647935 3.425262 -1.329220
H -2.444858 5.126131 -1.840514
H -4.640500 -1.799857 1.021097
C -4.286092 -2.541570 3.024581
C -2.857281 -1.178316 4.416224
H -2.099767 0.632303 3.498665
H -6.093652 0.354521 0.759867
C -6.978427 2.269072 1.218581
C -5.436025 4.096401 1.573080
H -3.329554 3.590427 1.402485
H 4.640581 1.799654 -1.022073
C 4.285012 2.541152 -3.025432
C 2.855397 1.177755 -4.416109
H 2.098350 -0.632724 -3.497894
H 6.093738 -0.354550 -0.760260
C 6.978468 -2.268995 -1.219545
C 5.436038 -4.096322 -1.573942
H 3.329591 -3.590450 -1.402652
H -2.623871 -3.865130 -0.106872
C -4.059322 -4.952146 -1.330527
C -4.993327 -3.712944 -3.187574
H -4.290480 -1.676483 -3.416595
H 2.624139 3.865423 0.107059
C 4.060126 4.952278 1.330212
C 4.994807 3.712927 3.186816
H 4.291973 1.676477 3.415980
H -4.889683 -3.440916 2.880174
C -3.636146 -2.321968 4.240225
H -2.343027 -1.003921 5.364406
H -8.002177 1.891331 1.164953
C -6.742350 3.615572 1.507069
H -5.248931 5.149057 1.798125
H 4.888688 3.440513 -2.881473
C 3.634365 2.321423 -4.240681
H 2.340597 1.003253 -5.363976
H 8.002221 -1.891234 -1.166112
C 6.742362 -3.615454 -1.508206
H 5.248923 -5.148950 -1.799100
H -3.991612 -5.871839 -0.744718
C -4.908399 -4.886552 -2.435160
H -5.656139 -3.664364 -4.054551
H 3.992227 5.872008 0.744484
C 4.909637 4.886582 2.434510
H 5.657955 3.664253 4.053530
H -3.732437 -3.048204 5.050909
H -7.583344 4.291397 1.680485

H 3.730195 3.047572 -5.051497
H 7.583337 -4.291215 -1.681964
H -5.507481 -5.756324 -2.714666
H 5.508873 5.756308 2.713826
S -2.452860 -1.232637 -1.236011
O -1.713730 -1.670417 -0.004355
S 2.453519 1.232821 1.236087
O 1.714021 1.670627 0.004669
N 1.676444 0.682755 2.456183
C 1.072843 1.574065 3.428802
H 0.827229 0.980010 4.323089
H 1.746874 2.387388 3.752636
H 0.135939 2.030986 3.066204
N -1.675554 -0.682521 -2.455921
C -1.071836 -1.573701 -3.428569
H -1.745898 -2.386874 -3.752718
H -0.825952 -0.979490 -4.322675
H -0.135089 -2.030817 -3.065816
Na 0.034304 0.852824 -1.382109
Na -0.034155 -0.852271 1.383109

κ*O*,κ*S*,κ*Cl*-$Na_2(THF)_2$

E = –5788.50372250

Cl 1.847915 1.787980 1.844655
Cl -1.848076 -1.788312 -1.844643
S 1.135029 0.366063 -1.738780
S -1.135021 -0.366148 1.738730
O 0.709298 -4.029897 -0.486474
O -0.709234 4.029531 0.486951
C 2.978155 0.803354 0.925046
C -2.978194 -0.803508 -0.925072
P 2.824415 0.979404 -0.814328
P -2.824417 -0.979473 0.814307
C 1.729277 -4.277107 -1.439539
C -0.376186 -4.876353 -0.835430
C -1.729541 4.277152 1.439540
C 0.375961 4.876364 0.835769
C 4.275908 0.150392 -1.540439
C 3.051808 2.767877 -1.101746
C -4.275877 -0.150340 1.540341
C -3.051885 -2.767905 1.101882
C 5.059250 -1.008983 1.442779
H 2.228840 -5.238838 -1.212765
H 2.473409 -3.472394 -1.359895
C 0.984588 -4.341213 -2.766452
C -0.367738 -4.963613 -2.370914
H -1.293876 -4.440970 -0.418388
H -0.223924 -5.871188 -0.378843
C -5.059067 1.008998 -1.443027
H -2.229215 5.238638 1.211981
H -2.473497 3.472252 1.360172
C -0.985327 4.342219 2.766705
C 0.367413 4.963816 2.371221
H 1.293806 4.441210 0.418824
H 0.223391 5.871092 0.379039
C 5.561429 0.626281 -1.255065
C 4.110945 -0.957988 -2.372434
C 3.851745 3.525525 -0.236873
C 2.433323 3.380546 -2.195530
C -5.561412 -0.626127 1.254855
C -4.110892 0.958012 2.372366

C -3.851861 -3.525606 0.237093
C -2.433471 -3.380475 2.195761
C 5.589629 -2.124099 0.803866
C 5.874651 -0.086193 2.098009
H 0.844007 -3.323352 -3.161914
H 1.524492 -4.923829 -3.525918
H -1.209317 -4.407823 -2.807160
H -0.452760 -6.007730 -2.704295
C -5.589392 2.124201 -0.804219
C -5.874511 0.086210 -2.098206
H -0.845262 3.324690 3.163195
H -1.525378 4.925725 3.525382
H 1.208592 4.407471 2.807535
H 0.453129 6.007894 2.704536
H 5.695130 1.487600 -0.598213
C 6.673036 -0.009129 -1.798958
C 5.227807 -1.598150 -2.907152
H 3.100350 -1.307460 -2.596570
H 4.303917 3.043911 0.633323
C 4.033403 4.886417 -0.473040
C 2.619934 4.742295 -2.428216
H 1.792503 2.782429 -2.848591
H -5.695115 -1.487419 0.597965
C -6.673014 0.009350 1.798677
C -5.227751 1.598252 2.907002
H -3.100284 1.307415 2.596563
H -4.304010 -3.044076 -0.633160
C -4.033600 -4.886460 0.473418
C -2.620169 -4.742185 2.428611
H -1.792628 -2.782315 2.848760
H 4.917012 -2.820277 0.302041
C 6.973086 -2.304791 0.799243
C 7.252832 -0.273845 2.085731
H 5.421373 0.773894 2.596635
H -4.916747 2.820381 -0.302430
C -6.972836 2.304990 -0.799653
C -7.252680 0.273955 -2.085977
H -5.421278 -0.773952 -2.596741
H 7.674455 0.361364 -1.569976
C 6.507547 -1.124263 -2.621913
H 5.095539 -2.469250 -3.553247
H 4.650123 5.476997 0.208419
C 3.419895 5.495422 -1.568956
H 2.131637 5.217758 -3.281976
H -7.674442 -0.361065 1.569610
C -6.507503 1.124461 2.621661
H -5.095468 2.469336 3.553116
H -4.650346 -5.477087 -0.207977
C -3.420151 -5.495370 1.569422
H -2.131922 -5.217567 3.282444
H 7.404131 -3.168627 0.287970
C 7.802240 -1.381916 1.433675
H 7.904360 0.446295 2.586236
H -7.403838 3.168897 -0.288465
C -7.802035 1.382114 -1.434026
H -7.904242 -0.446186 -2.586437
H 7.382370 -1.626363 -3.042140
H 3.559424 6.564145 -1.748508
H -7.382322 1.626620 3.041826
H -3.559752 -6.564063 1.749099
H 8.885501 -1.523934 1.422190
H -8.885287 1.524201 -1.422576

S 3.279430 -0.774497 1.539754
S -3.279250 0.774366 -1.539836
Na -0.575031 1.801037 -0.038063
Na 0.575249 -1.801195 0.038116
O 2.758278 -1.858820 0.632711
N 2.831890 -0.745682 3.012876
N -2.831541 0.745488 -3.012907
O -2.758128 1.858647 -0.632730
C -3.128605 1.933765 -3.785700
H -2.654099 2.850536 -3.387049
H -2.740498 1.785820 -4.804398
H -4.214326 2.140520 -3.878578
C 3.129045 -1.934026 3.785546
H 2.740855 -1.786253 4.804236
H 2.654675 -2.850799 3.386742
H 4.214784 -2.140655 3.878462

κ*O*,κ*N*-$Na_2(THF)_4$

E = –6253.09435252

C 3.828451 0.031806 -0.572054
S 2.309951 -0.670931 -0.278888
P 5.214774 0.020291 0.518595
O 1.490068 -0.383168 -1.499382
S 4.862434 -0.349086 2.432822
O -1.489834 0.383219 1.499105
S -2.309814 0.671039 0.278693
C -3.828281 -0.031783 0.571962
P -5.214687 -0.020145 -0.518591
S -4.862406 0.348814 -2.432901
Cl -4.253746 -0.237755 2.275619
Cl 4.253986 0.237958 -2.275662
C -6.436133 1.179192 0.121794
C -6.014747 2.270300 0.887388
C -7.782181 1.067313 -0.245739
C -6.932821 3.242437 1.281937
H -4.965153 2.355707 1.177089
C -8.698326 2.036778 0.155022
H -8.112729 0.219403 -0.851778
C -8.274263 3.126404 0.918550
H -6.596531 4.093664 1.878829
H -9.748695 1.943715 -0.131465
H -8.993773 3.887094 1.231234
C -6.021576 -1.650542 -0.295036
C -5.883560 -2.615795 -1.296274
C -6.723779 -1.963405 0.876061
C -6.423162 -3.889031 -1.123212
H -5.356326 -2.347156 -2.216140
C -7.262681 -3.237506 1.045743
H -6.851635 -1.209910 1.655507
C -7.109295 -4.203052 0.049889
H -6.309631 -4.638518 -1.910214
H -7.809978 -3.476251 1.960863
H -7.534249 -5.200630 0.185778
C 6.436401 -1.178763 -0.121955
C 6.015087 -2.270033 -0.887359
C 7.782503 -1.066548 0.245278
C 6.933290 -3.242001 -1.282018
H 4.965444 -2.355685 -1.176821
C 8.698776 -2.035850 -0.155594
H 8.112994 -0.218506 0.851161
C 8.274787 -3.125637 -0.918931

H 6.597065 -4.093355 -1.878765
H 9.749188 -1.942530 0.130651
H 8.994396 -3.886195 -1.231704
C 6.021413 1.650863 0.295429
C 5.883395 2.615796 1.296978
C 6.723420 1.964178 -0.875667
C 6.422785 3.889163 1.124221
H 5.356320 2.346798 2.216831
C 7.262097 3.238414 -1.045046
H 6.851315 1.210928 -1.655341
C 7.108699 4.203642 -0.048884
H 6.309259 4.638397 1.911465
H 7.809237 3.477515 -1.960167
H 7.533472 5.201329 -0.184541
C -2.410354 2.454781 0.231346
C -2.079044 3.167338 1.382881
C -2.940789 3.090739 -0.893816
C -2.277193 4.548424 1.404699
H -1.668025 2.631930 2.240117
C -3.130891 4.469564 -0.858485
H -3.225094 2.492867 -1.762952
C -2.802208 5.198453 0.287858
H -2.015427 5.118733 2.298936
H -3.545125 4.979486 -1.731237
H -2.956466 6.279732 0.308925
C 2.410427 -2.454702 -0.231501
C 2.078872 -3.167283 -1.382949
C 2.941049 -3.090653 0.893576
C 2.276952 -4.548380 -1.404762
H 1.667716 -2.631885 -2.240125
C 3.131098 -4.469487 0.858249
H 3.225557 -2.492776 1.762641
C 2.802160 -5.198399 -0.288006
H 2.014993 -5.118701 -2.298935
H 3.545480 -4.979399 1.730937
H 2.956367 -6.279685 -0.309070
N 1.746390 -0.337290 1.122523
N -1.746365 0.337452 -1.122778
C 1.736262 1.055845 1.537472
H 2.706804 1.351889 1.969777
H 1.479572 1.767279 0.735932
H 0.966896 1.168991 2.313822
C -1.736039 -1.055724 -1.537629
H -2.706410 -1.351769 -1.970320
H -0.966315 -1.168937 -2.313611
H -1.479741 -1.767117 -0.735927
O -1.406724 -3.270747 1.295366
C -2.795158 -3.462744 1.042897
C -0.753376 -4.464128 0.898762
C -2.871456 -4.336655 -0.218115
H -3.254402 -3.972143 1.909271
H -3.258822 -2.472901 0.927709
C -1.432571 -4.849392 -0.407688
H 0.320887 -4.257580 0.803378
H -0.895393 -5.244448 1.671231
H -3.206597 -3.751412 -1.084568
H -3.592526 -5.155079 -0.083470
H -0.939473 -4.333883 -1.245387
H -1.377586 -5.929564 -0.602861
O 0.298187 -1.174001 4.095105
C -0.546944 -0.443369 4.966056
C 1.650276 -0.940267 4.484615

C 0.196250 0.862043 5.194383
H -1.524987 -0.327131 4.477295
H -0.688791 -0.996070 5.915153
C 1.660985 0.406507 5.228855
H 1.992041 -1.762276 5.138041
H 2.265169 -0.935866 3.571867
H -0.118840 1.378048 6.112158
H 0.013917 1.540769 4.346686
H 2.006741 0.274500 6.264474
H 2.333325 1.130650 4.748156
O -0.298004 1.172750 -4.095411
C 0.546817 0.441415 -4.966074
C -1.650208 0.939562 -4.484865
C -0.197060 -0.863653 -5.194129
H 0.689065 0.993774 -5.915311
H 1.524739 0.324772 -4.477168
C -1.661537 -0.407325 -5.228889
H -2.265091 0.935586 -3.572107
H -1.991613 1.761620 -5.138417
H -0.015198 -1.542266 -4.346242
H 0.117849 -1.380065 -6.111737
H -2.334350 -1.131028 -4.748189
H -2.007050 -0.275291 -6.264586
O 1.406720 3.270762 -1.295354
C 2.795129 3.462920 -1.042825
C 0.753141 4.463938 -0.898494
C 2.871312 4.336913 0.218154
H 3.258871 2.473121 -0.927563
H 3.254369 3.972299 -1.909210
C 1.432245 4.848974 0.408052
H 0.895047 5.244477 -1.670764
H -0.321088 4.257172 -0.803189
H 3.591922 5.155705 0.083271
H 3.206993 3.751900 1.084551
H 1.376812 5.929036 0.603705
H 0.939411 4.332884 1.245551
Na 0.208804 1.412134 -1.887427
Na -0.208653 -1.412138 1.887030

6.4.5 Coordinates of the monomeric structures of 1-K

κ*O*-K(THF)$_3$

E = –3224.93559097

O 0.494338 0.319543 0.099420
O 2.409912 2.806809 1.456226
O 4.487364 -0.412954 -0.174579
O 1.117257 3.228826 -2.162012
S 0.448202 -1.073668 0.653949
C 1.097242 3.322053 1.680835
C 2.802790 2.006892 2.563599
C 5.144671 -1.233466 -1.130358
C 4.558905 -1.012964 1.122866
C 0.953746 4.612354 -1.877065
C -0.095638 2.685002 -2.692288
N 1.159516 -1.429963 1.979543
C -1.202560 -1.498353 0.651394
C 1.334493 -2.096260 -0.517886
H 0.403587 2.920607 0.924350

H 1.134643 4.419685 1.574666
C 0.701438 2.897061 3.094570
C 2.045344 2.584222 3.743870
H 3.897624 2.062380 2.660847
H 2.516381 0.951729 2.391467
H 5.641563 -0.585519 -1.869716
H 4.404867 -1.861833 -1.659622
C 6.107937 -2.089346 -0.327845
C 5.297343 -2.334786 0.940332
H 3.538500 -1.147044 1.518696
H 5.112428 -0.329258 1.791142
H 1.463701 5.216666 -2.650876
H 1.425519 4.836103 -0.907253
C -0.545520 4.856876 -1.892997
C -0.993110 3.879050 -2.972765
H -0.545697 2.004028 -1.949725
H 0.137128 2.096863 -3.594150
C 0.491711 -1.132542 3.225684
Cl -1.467634 -3.197288 1.050386
P -2.334662 -0.746883 -0.474421
C 1.315093 -1.725268 -1.863496
C 1.901160 -3.303907 -0.112737
H 0.085475 1.986802 3.053218
H 0.125662 3.669771 3.622608
H 2.535956 3.504687 4.099218
H 1.970629 1.886032 4.589888
H 7.025917 -1.522841 -0.101281
H 6.395532 -3.012294 -0.851407
H 4.579115 -3.151877 0.774442
H 5.913255 -2.596645 1.812231
H -0.806025 5.903699 -2.104061
H -0.990318 4.577759 -0.923930
H -0.789525 4.293602 -3.973433
H -2.059575 3.622473 -2.921240
H -0.609481 -1.209582 3.158340
H 0.731453 -0.124947 3.613180
H 0.825859 -1.853512 3.988553
S -2.077692 -0.852634 -2.449331
C -3.934633 -1.508585 0.005719
C -2.579283 0.988838 0.061363
H 0.809885 -0.805140 -2.162459
C 1.899884 -2.565427 -2.809132
C 2.481596 -4.138262 -1.067188
H 1.886519 -3.560323 0.947105
C -4.357366 -1.465346 1.340294
C -4.744288 -2.105817 -0.959335
C -3.186349 1.883314 -0.824774
C -2.276291 1.401107 1.361721
H 1.889163 -2.281589 -3.864196
C 2.483803 -3.770289 -2.413245
H 2.933605 -5.083337 -0.756634
H -3.719568 -1.004352 2.098177
C -5.584048 -2.015724 1.699762
C -5.972913 -2.659565 -0.596363
H -4.393813 -2.124085 -1.995385
H -3.404972 1.548439 -1.842590
C -3.494943 3.178114 -0.411670
C -2.579717 2.698920 1.770348
H -1.794564 0.694935 2.041275
H 2.937962 -4.427612 -3.158473
H -5.911048 -1.980329 2.741852
C -6.394012 -2.614346 0.731376

H -6.603363 -3.127870 -1.356307
H -3.973915 3.871494 -1.107463
C -3.190463 3.589020 0.886635
H -2.337588 3.017748 2.786710
H -7.356738 -3.046326 1.015887
H -3.429953 4.604923 1.210392
K 2.702213 1.480021 -0.881829

κ*O*-K(THF)$_4$

E = –3457.23124949

O 0.054487 -0.830675 1.163115
O 2.020833 2.043168 2.445604
O 3.569331 -0.931539 0.927114
O 1.970800 3.445681 -0.839793
S -0.726821 -2.107770 1.048327
C 0.788176 2.230440 3.117470
C 2.918897 1.401241 3.342473
C 4.585456 -1.351510 0.018141
C 3.215138 -1.998189 1.802806
C 2.252492 4.443019 0.127630
C 1.007856 3.947497 -1.762484
N -0.929796 -3.009261 2.281013
C -2.155249 -1.612663 0.267464
C 0.207689 -3.190704 -0.030102
H 0.002596 2.382354 2.364448
H 0.830628 3.133003 3.760083
C 0.617426 0.965023 3.939549
C 2.053798 0.679404 4.392883
H 3.576045 2.153349 3.815747
H 3.544390 0.720132 2.746753
H 5.413362 -0.623358 0.039236
H 4.165637 -1.355151 -1.002422
C 5.008994 -2.743117 0.469938
C 3.737185 -3.257273 1.135173
H 2.124553 -1.986924 1.941100
H 3.695209 -1.843364 2.788474
H 3.034491 5.134435 -0.243994
H 2.614598 3.938744 1.034489
C 0.928723 5.160839 0.313094
C 0.394111 5.200760 -1.119079
H 0.269442 3.149127 -1.943210
H 1.496689 4.182291 -2.724661
C -1.874132 -2.575179 3.284256
Cl -3.193714 -2.938468 -0.231381
P -2.255265 -0.045345 -0.518829
C 0.748237 -2.631002 -1.187877
C 0.343551 -4.548766 0.247746
H 0.246156 0.165195 3.281610
H -0.085589 1.088830 4.775345
H 2.243217 1.090144 5.395932
H 2.266016 -0.398313 4.433650
H 5.827650 -2.679704 1.204973
H 5.352734 -3.369502 -0.365616
H 3.020681 -3.607349 0.377503
H 3.910157 -4.076947 1.846833
H 1.033364 6.156507 0.767091
H 0.272776 4.556155 0.958409
H 0.742513 6.109430 -1.632961
H -0.704414 5.198710 -1.166571
H -2.870555 -2.337148 2.863828
H -1.536323 -1.691258 3.859125

H -2.014143 -3.392667 4.007743
S -0.964445 0.514770 -1.957818
C -3.962709 -0.028566 -1.180313
C -2.324801 1.260684 0.767763
H 0.570098 -1.576150 -1.415350
C 1.463412 -3.443248 -2.064442
C 1.059410 -5.353731 -0.638044
H -0.106858 -4.941401 1.159836
C -5.050466 -0.092234 -0.300342
C -4.185071 0.044214 -2.555467
C -2.187776 2.600377 0.386679
C -2.627983 0.948092 2.095387
H 1.892970 -3.012652 -2.971656
C 1.623970 -4.802974 -1.788932
H 1.177185 -6.418984 -0.425772
H -4.880052 -0.151721 0.777271
C -6.349930 -0.080842 -0.798896
C -5.488720 0.051089 -3.052455
H -3.323716 0.102627 -3.226269
H -1.941388 2.835157 -0.652202
C -2.370459 3.616201 1.323496
C -2.797533 1.966032 3.033411
H -2.727273 -0.099853 2.382472
H 2.186053 -5.436864 -2.478813
H -7.196252 -0.127727 -0.109410
C -6.570895 -0.010422 -2.176224
H -5.658057 0.107174 -4.130521
H -2.276010 4.660842 1.017221
C -2.675551 3.300927 2.648826
H -3.026174 1.712758 4.071434
H -7.591942 -0.001380 -2.565583
H -2.813906 4.098190 3.383038
K 1.685884 0.846629 -0.025288
O 2.955649 0.351186 -2.439720
C 3.904276 1.378693 -2.731517
C 2.284235 -0.035546 -3.628570
C 3.738612 1.713659 -4.214889
H 3.675198 2.244409 -2.088610
H 4.920693 1.023130 -2.491504
C 2.337125 1.191569 -4.518342
H 2.804782 -0.890438 -4.103511
H 1.263093 -0.347934 -3.363874
H 4.482125 1.173044 -4.821480
H 3.859674 2.787430 -4.416746
H 2.172622 0.963326 -5.580850
H 1.570640 1.915970 -4.198736

κ*Ph*,κ*S*-K(THF)$_3$

E = –3224.93366894

K -1.588434 -0.061148 0.317991
Cl 0.521440 -0.825218 -2.003475
S 1.057144 0.999996 1.769935
C 0.449683 -2.972834 0.665417
C -0.566847 -3.574061 -0.080284
C -1.789907 -3.843346 0.533232
C -1.989231 -3.520052 1.877441
C -0.961122 -2.928403 2.615123
C 0.264734 -2.647509 2.010916
O -2.936257 0.509655 2.634382
C 1.798550 -0.917121 -0.780128
P 2.150553 0.532249 0.147352

H -0.375659 -3.832796 -1.122421
H -2.590093 -4.316441 -0.040301
H -2.948894 -3.734532 2.353828
H -1.113297 -2.685172 3.669049
H 1.078917 -2.172305 2.560268
C -2.130278 0.406404 3.810761
C -3.411289 1.842325 2.493767
C 3.931249 0.512509 0.557146
C 2.030220 1.878222 -1.093820
H -2.574752 -0.329720 4.500668
H -1.132013 0.040705 3.515863
C -2.059346 1.809052 4.412886
C -2.386131 2.696794 3.215980
H -3.499165 2.061474 1.419340
H -4.412928 1.946281 2.953209
C 4.845649 -0.129714 -0.284508
C 4.387259 1.214823 1.674106
C 2.639296 1.728575 -2.346753
C 1.377952 3.070194 -0.778695
H -1.077887 2.021667 4.859097
H -2.821894 1.934512 5.197630
H -1.492939 2.827723 2.582991
H -2.766837 3.690261 3.492106
H 4.477421 -0.698863 -1.140391
C 6.208676 -0.062267 -0.008705
C 5.753333 1.279557 1.947357
H 3.659134 1.698857 2.330251
H 3.142780 0.792169 -2.597394
C 2.590456 2.766811 -3.272414
C 1.333137 4.110699 -1.707577
H 0.909030 3.166254 0.204576
H 6.919705 -0.571861 -0.663585
C 6.665350 0.643365 1.106219
H 6.105476 1.827280 2.824910
H 3.063750 2.644891 -4.249639
C 1.937684 3.960215 -2.954196
H 0.824238 5.043760 -1.453405
H 7.735482 0.691230 1.322559
H 1.902541 4.774424 -3.682213
O -3.505250 -1.288903 -1.119910
C -4.767951 -1.725668 -0.627205
C -3.225319 -1.888783 -2.383801
C -5.466728 -2.371403 -1.812344
H -4.611292 -2.451267 0.192035
H -5.307676 -0.858230 -0.215442
C -4.286381 -2.960914 -2.577369
H -3.285687 -1.115022 -3.170839
H -2.197164 -2.282206 -2.372447
H -5.974518 -1.606146 -2.421257
H -6.214756 -3.117429 -1.509936
H -4.500348 -3.157305 -3.637106
H -3.966245 -3.909175 -2.116619
O -2.473245 2.203111 -0.862506
C -3.545485 2.068062 -1.778136
C -2.279716 3.595667 -0.697152
C -4.563553 3.116236 -1.335608
H -3.187918 2.271639 -2.806122
H -3.899825 1.029115 -1.729782
C -3.685803 4.213224 -0.699746
H -1.718010 3.755314 0.233906
H -1.673343 3.986104 -1.534733
H -5.252225 2.686890 -0.592562

H -5.170034 3.483722 -2.175120
H -4.020831 4.448670 0.320654
H -3.704867 5.150514 -1.273331
S 1.988849 -2.484925 -0.101670
O 2.973575 -2.300501 0.996729
N 2.155377 -3.579425 -1.175543
C 3.218579 -3.382439 -2.137693
H 4.230369 -3.482456 -1.699912
H 3.128965 -4.151444 -2.920150
H 3.176315 -2.395682 -2.639568

κ*N*-K(THF)$_3$

E = –3224.93163716

O 1.502702 -4.048887 -0.704662
O 4.350580 -0.122348 -1.678606
O 4.511083 -0.630188 1.879782
S 0.267545 -0.123928 0.039798
C 1.061469 -5.192430 0.005248
C 0.526832 -3.801525 -1.705849
C 4.019204 1.208581 -1.282347
C 4.014947 -0.319380 -3.049275
C 5.799106 -0.067097 1.659769
C 3.780931 0.184076 2.788956
C -1.395095 -0.473323 -0.212925
C 0.536150 1.642721 0.037211
H 1.536133 -5.183242 0.997584
H 1.388673 -6.108022 -0.522797
C -0.473506 -5.105657 0.046413
C -0.803925 -4.028230 -1.001111
H 0.666924 -4.506675 -2.548845
H 0.655040 -2.769969 -2.059149
H 4.869332 1.643989 -0.734547
H 3.146435 1.179371 -0.607636
C 3.683551 1.957466 -2.561453
C 3.105722 0.839392 -3.420797
H 3.529654 -1.302446 -3.162733
H 4.937276 -0.323253 -3.659697
H 5.974148 -0.037466 0.571672
H 6.576091 -0.710233 2.108616
C 5.782687 1.330890 2.292946
C 4.295558 1.588065 2.533628
H 3.983864 -0.128313 3.832334
H 2.709755 0.052177 2.568942
Cl -1.849847 -0.602965 -1.924232
P -2.638710 0.311401 0.776808
C 0.825557 2.334359 1.210510
C 0.366702 2.312435 -1.177113
H -0.834830 -4.814904 1.043366
H -0.927107 -6.077536 -0.195465
H -1.601060 -4.327084 -1.696071
H -1.108882 -3.087514 -0.518542
H 4.596358 2.369098 -3.022288
H 2.982795 2.785044 -2.385249
H 2.077555 0.612588 -3.100513
H 3.109254 1.057446 -4.498079
H 6.254656 2.087312 1.649594
H 6.326640 1.325403 3.250003
H 3.812411 2.002698 1.635357
H 4.102597 2.276263 3.368910
S -2.197768 0.528507 2.691835
C -3.077729 1.922396 0.018385

C -4.130910 -0.714285 0.556015
H 0.918007 1.770244 2.138411
C 0.980582 3.719422 1.158608
C 0.507176 3.696546 -1.212801
H 0.120946 1.749410 -2.079018
C -3.779491 1.992071 -1.193464
C -2.614031 3.099696 0.613049
C -5.391976 -0.165623 0.815440
C -4.015277 -2.071162 0.242491
H 1.219463 4.270422 2.071448
C 0.824587 4.399891 -0.048821
H 0.369196 4.229884 -2.156111
H -4.156690 1.080438 -1.660440
C -4.003248 3.226615 -1.800723
C -2.834840 4.331131 0.000754
H -2.078046 3.031579 1.562920
H -5.483678 0.893016 1.072513
C -6.528842 -0.967875 0.747910
C -5.154421 -2.871671 0.176567
H -3.024589 -2.486554 0.046153
H 0.940926 5.485710 -0.082745
H -4.555230 3.274411 -2.742725
C -3.527881 4.397071 -1.207861
H -2.461257 5.243892 0.471092
H -7.512006 -0.534884 0.947628
C -6.411730 -2.321568 0.426863
H -5.059177 -3.931220 -0.073487
H -3.704046 5.363279 -1.687341
H -7.304590 -2.949335 0.372886
N 0.800660 -0.558877 1.422861
C 0.380900 -1.827239 1.982121
H -0.680250 -1.818678 2.283655
H 0.538618 -2.690434 1.310447
H 0.977067 -2.003772 2.891930
O 0.993843 -0.617141 -1.175626
K 3.132652 -1.955784 -0.042393

κ*O*,κ*S*-K(THF)$_3$

E = –3224.93442813

Cl 1.983905 2.056997 1.127585
S -1.117913 0.733236 -1.471419
O -2.331290 2.434846 1.850012
C 1.620750 0.523597 0.342991
P 0.867672 0.552744 -1.244812
C -1.257057 2.869518 2.689892
C -2.470830 3.315188 0.741132
C 1.489008 -0.890141 -2.183237
C 1.703646 1.973786 -2.051839
C 2.811642 -0.990316 2.342583
H -1.625585 2.976693 3.723588
H -0.466933 2.099798 2.676702
C -0.757810 4.182706 2.099726
C -1.141195 4.034737 0.632056
H -2.704766 2.717844 -0.152865
H -3.301200 4.024554 0.925268
C 2.773411 -1.390061 -1.945483
C 0.697598 -1.458255 -3.181971
C 3.097708 2.097261 -1.990932
C 0.956260 2.914396 -2.761009
C 3.748848 -1.825035 1.733455
C 3.066173 -0.390038 3.573169

H 0.321549 4.319673 2.254513
H -1.282160 5.040881 2.550567
H -0.418173 3.387401 0.111300
H -1.210814 4.989779 0.091896
H 3.377821 -0.949858 -1.149871
C 3.256937 -2.455806 -2.699378
C 1.183085 -2.527874 -3.934972
H -0.307104 -1.060805 -3.347849
H 3.683713 1.374441 -1.419295
C 3.732510 3.151431 -2.641230
C 1.594833 3.971857 -3.409994
H -0.131188 2.803341 -2.791216
H 3.509668 -2.286397 0.772554
C 4.964960 -2.058019 2.370029
C 4.292212 -0.620335 4.197382
H 2.304768 0.246028 4.026709
H 4.258269 -2.848052 -2.504647
C 2.461603 -3.028933 -3.694358
H 0.556241 -2.972930 -4.711738
H 4.819909 3.244834 -2.588701
C 2.982126 4.090502 -3.352737
H 1.003147 4.706121 -3.962162
H 5.703567 -2.716320 1.906688
C 5.238519 -1.452027 3.598671
H 4.507744 -0.149274 5.159375
H 2.840582 -3.869175 -4.281431
H 3.482973 4.918455 -3.860604
H 6.195453 -1.633308 4.093789
S 1.260106 -0.689200 1.483317
O -2.561215 -2.669360 0.086447
C -3.194944 -3.867448 0.508169
C -1.887821 -2.869725 -1.160140
C -3.241716 -4.760445 -0.721292
H -2.603215 -4.336912 1.317124
H -4.190083 -3.622004 0.912740
C -1.948259 -4.366075 -1.426562
H -2.416577 -2.298842 -1.944061
H -0.869116 -2.464668 -1.076587
H -4.113049 -4.503736 -1.345677
H -3.304860 -5.828890 -0.470814
H -1.939310 -4.603206 -2.499786
H -1.089859 -4.878428 -0.962986
O 0.341057 -0.255920 2.585893
N 0.852410 -1.908522 0.624130
C 0.546188 -3.128005 1.339813
H -0.347188 -3.053466 1.988076
H 0.338080 -3.914088 0.599737
H 1.380630 -3.494360 1.970446
K -2.150308 -0.295899 1.392087
O -4.880110 -0.121403 0.981273
C -5.448616 1.174655 1.146840
C -5.186941 -0.582976 -0.323531
C -5.745907 1.710829 -0.264705
H -6.368664 1.107484 1.753997
H -4.718306 1.787905 1.696545
C -5.109769 0.665592 -1.183817
H -4.457862 -1.364715 -0.580483
H -6.203956 -1.022702 -0.351833
H -5.338950 2.719438 -0.422547
H -6.831601 1.763355 -0.436418
H -4.054117 0.906855 -1.389150
H -5.627488 0.561757 -2.147908

κ*O*,κ*N*-K(THF)$_2$

E = –2992.64304011

O -0.759698 0.136482 -0.079947
O -2.083466 3.354930 -0.408902
O -4.475404 -0.143911 -0.352061
S -0.304535 -1.086692 -0.817843
C -2.308340 4.740161 -0.606985
C -1.723328 3.103444 0.953696
C -4.143481 -0.187776 1.037640
C -5.098640 -1.360503 -0.749060
N -0.805075 -1.300813 -2.274757
C 1.386547 -1.104425 -0.667080
C -1.023004 -2.476025 0.044682
H -3.121404 4.865448 -1.339331
H -1.398522 5.219383 -1.018023
C -2.632840 5.292484 0.769764
C -1.697465 4.462584 1.642884
H -0.758285 2.577041 0.984609
H -2.484794 2.438511 1.397195
H -4.491115 0.740541 1.519072
H -3.045694 -0.241089 1.136734
C -4.817625 -1.434015 1.590845
C -4.826267 -2.349106 0.371987
H -4.679749 -1.678342 -1.717421
H -6.183909 -1.194509 -0.884217
C -0.033906 -0.701348 -3.345262
Cl 2.120693 -2.599784 -1.238561
P 2.169879 -0.231293 0.650934
C -1.190775 -2.365792 1.425773
C -1.298051 -3.665048 -0.628489
H -3.686235 5.088689 1.022593
H -2.465548 6.376134 0.848353
H -0.680768 4.883027 1.615859
H -2.010791 4.406097 2.694836
H -5.847021 -1.208433 1.913167
H -4.275170 -1.857515 2.447648
H -3.837866 -2.813738 0.240023
H -5.577691 -3.149479 0.425350
H 1.052715 -0.872599 -3.244038
H -0.174868 0.394173 -3.455281
H -0.352475 -1.153077 -4.297171
S 1.815362 -0.686434 2.557000
C 3.949620 -0.435067 0.252326
C 1.900590 1.557076 0.341721
H -0.924177 -1.434342 1.928288
C -1.669403 -3.463912 2.136564
C -1.778286 -4.756602 0.093655
H -1.145938 -3.706629 -1.707584
C 4.423620 -0.106673 -1.024106
C 4.837413 -0.891893 1.225616
C 2.077473 2.455465 1.397252
C 1.587581 2.039493 -0.932605
H -1.808199 -3.388791 3.217630
C -1.966638 -4.655884 1.472842
H -2.006918 -5.691061 -0.424177
H 3.726933 0.243546 -1.789406
C 5.778250 -0.233795 -1.317374
C 6.194888 -1.021086 0.928710
H 4.443754 -1.139920 2.215639
H 2.299763 2.061253 2.392541

C 1.954465 3.826742 1.177770
C 1.447061 3.409312 -1.145426
H 1.438048 1.328134 -1.747388
H -2.343854 -5.513242 2.035354
H 6.145059 0.023286 -2.314113
C 6.666271 -0.691749 -0.340855
H 6.886499 -1.380839 1.694535
H 2.098761 4.523875 2.006917
C 1.636812 4.305928 -0.093336
H 1.188260 3.780734 -2.140273
H 7.729437 -0.791625 -0.573397
H 1.529789 5.380243 -0.263238
K -2.611705 1.131295 -1.830769

κ*O*-K(THF)$_2$

E = –2992.64651467

O -0.759516 -0.125480 -0.699322
O -2.191738 3.019263 0.467396
O -4.016975 0.217562 -1.395074
S -0.244620 -1.530655 -0.783014
C -2.462083 4.386559 0.751585
C -1.710496 2.354205 1.635620
C -4.248259 0.252568 0.003947
C -4.018012 -1.145786 -1.821617
N -0.508504 -2.386668 -2.043210
C 1.408661 -1.402735 -0.388123
C -1.132002 -2.442806 0.467880
H -3.465116 4.647245 0.375813
H -1.725273 5.020148 0.223425
C -2.331680 4.537239 2.261215
C -1.302704 3.461320 2.589502
H -0.885884 1.682326 1.356381
H -2.522947 1.742189 2.070203
H -4.648510 1.243455 0.264563
H -3.297844 0.104602 0.548218
C -5.195807 -0.902872 0.260684
C -4.624169 -1.967678 -0.674511
H -2.988203 -1.474415 -2.043126
H -4.613623 -1.206606 -2.747005
C 0.282957 -2.084703 -3.215081
Cl 2.184569 -2.966370 -0.145018
P 2.004709 -0.028666 0.536227
C -1.521732 -1.753332 1.616144
C -1.377956 -3.806461 0.320466
H -3.290558 4.310308 2.754469
H -2.028546 5.550434 2.560655
H -0.286081 3.814608 2.353771
H -1.313478 3.141437 3.640570
H -6.220693 -0.623275 -0.031962
H -5.211065 -1.218077 1.313923
H -3.838414 -2.539219 -0.159727
H -5.381438 -2.682890 -1.025302
H 1.369599 -2.057679 -3.007450
H 0.018059 -1.121580 -3.695047
H 0.109631 -2.871818 -3.964640
S 1.424455 0.304206 2.414612
C 3.828014 -0.221007 0.449866
C 1.716719 1.457475 -0.509806
H -1.275225 -0.695760 1.718995
C -2.203722 -2.440438 2.617707
C -2.061028 -4.483498 1.329435

H -1.052785 -4.303377 -0.593904
C 4.445466 -0.596409 -0.750221
C 4.609005 0.059207 1.571571
C 1.496198 2.692559 0.101155
C 1.765301 1.375242 -1.907133
H -2.520450 -1.908982 3.518223
C -2.479425 -3.801421 2.473042
H -2.270330 -5.550256 1.220228
H 3.833553 -0.835287 -1.622754
C 5.833142 -0.681743 -0.822514
C 5.999536 -0.027817 1.497019
H 4.105484 0.341890 2.500546
H 1.460211 2.733537 1.193326
C 1.305347 3.837786 -0.673488
C 1.583750 2.521008 -2.680555
H 1.918084 0.402738 -2.380015
H -3.018111 -4.335120 3.259732
H 6.310560 -0.978369 -1.759632
C 6.612725 -0.396020 0.300653
H 6.605383 0.191715 2.379606
H 1.123765 4.799130 -0.186159
C 1.349859 3.755371 -2.065480
H 1.624098 2.451643 -3.770850
H 7.701796 -0.465352 0.241687
H 1.206561 4.652002 -2.673861
K -1.879902 1.848483 -1.927994

κ*O*,κ*Cl*-K(THF)$_2$

E = –2992.63903055

O -1.588665 3.277379 -0.410908
O 3.528624 3.073284 0.399639
S 1.233739 -0.823885 -0.953860
C -2.592879 3.712112 -1.318459
C -2.151359 3.069139 0.887532
C 4.633001 3.447420 -0.414084
C 3.854245 1.910333 1.167512
C 0.017390 -0.567501 0.188261
C 2.612817 -1.720003 -0.210261
H -2.419485 3.229258 -2.292767
H -2.522492 4.806953 -1.463398
C -3.913498 3.332581 -0.672282
C -3.593849 3.546599 0.803194
H -1.558983 3.623289 1.633694
H -2.087903 1.996762 1.133066
H 5.144788 4.323598 0.026587
H 4.262021 3.738919 -1.410154
C 5.550496 2.236670 -0.443976
C 5.340709 1.669153 0.955201
H 3.252650 1.064361 0.796898
H 3.589648 2.087984 2.221945
Cl 0.433094 0.517384 1.509155
P -1.369719 -1.655614 0.244094
C 2.344780 -2.766035 0.670946
C 3.919834 -1.391722 -0.566935
H -4.141115 2.272951 -0.870092
H -4.756056 3.937832 -1.034857
H -3.666512 4.616015 1.057981
H -4.255108 2.991384 1.482624
H 6.594837 2.496975 -0.666448
H 5.206221 1.518896 -1.205647
H 5.937793 2.233784 1.689233

H 5.606254 0.606473 1.044323
S -1.144337 -3.619620 0.520000
C -2.355845 -0.999383 1.647786
C -2.421612 -1.311174 -1.212886
H 1.305864 -3.019969 0.912907
C 3.410500 -3.478749 1.217906
C 4.978963 -2.114646 -0.019166
H 4.090942 -0.568069 -1.261563
C -3.519636 -0.247561 1.464372
C -1.942174 -1.315837 2.948723
C -3.260603 -2.306708 -1.715396
C -2.421369 -0.039835 -1.795730
H 3.211928 -4.295590 1.916057
C 4.724960 -3.153880 0.876760
H 6.006963 -1.863054 -0.291754
H -3.865454 -0.010751 0.456262
C -4.254567 0.191300 2.568164
C -2.665525 -0.862682 4.047029
H -1.052407 -1.933731 3.091869
H -3.234146 -3.297761 -1.254585
C -4.098496 -2.034777 -2.797101
C -3.261458 0.229305 -2.874326
H -1.753009 0.728914 -1.398383
H 5.556071 -3.715593 1.310092
H -5.172150 0.764411 2.413367
C -3.825358 -0.106556 3.859142
H -2.329385 -1.108548 5.057305
H -4.749940 -2.819167 -3.190362
C -4.101049 -0.766902 -3.377258
H -3.258244 1.220751 -3.334322
H -4.399659 0.239723 4.721897
H -4.754093 -0.554045 -4.227246
N 0.514976 -1.699558 -1.998551
C 1.249055 -2.628889 -2.818395
H 1.942422 -3.277817 -2.248196
H 0.523600 -3.295259 -3.310040
H 1.837190 -2.143999 -3.621333
O 1.881049 0.466378 -1.362038
K 1.049577 2.879412 -0.720083

κ*O*,κ*S*-K(THF)$_2$

E = –2992.64457655

Cl 2.530646 0.091045 1.164802
S 0.007944 1.771712 -1.767765
O 0.648841 4.085757 1.479877
C 1.018098 -0.442587 0.439937
P 0.715058 -0.030817 -1.240502
C 1.563781 3.481459 2.401185
C 1.353646 4.568067 0.342062
C -0.310166 -1.359824 -1.971487
C 2.354809 -0.293327 -2.022110
C 0.173322 -2.037033 2.561390
H 1.453749 3.959193 3.388973
H 1.308522 2.413094 2.499535
C 2.956068 3.667569 1.808600
C 2.654368 3.788946 0.319639
H 0.736449 4.394952 -0.553186
H 1.535459 5.655765 0.441286
C -0.135135 -2.688072 -1.569896
C -1.225788 -1.055288 -2.978744
C 3.118145 -1.422279 -1.698016

C 2.820752 0.604095 -2.984107
C -0.188162 -3.287851 2.058897
C 0.858845 -1.910859 3.767708
H 3.621294 2.826431 2.048741
H 3.422885 4.592742 2.183784
H 2.475342 2.793856 -0.117354
H 3.446938 4.288938 -0.255236
H 0.577543 -2.917600 -0.775477
C -0.885074 -3.700314 -2.162066
C -1.977838 -2.071118 -3.570176
H -1.349712 -0.011846 -3.280068
H 2.767188 -2.111698 -0.927195
C 4.331440 -1.650691 -2.340878
C 4.037971 0.373171 -3.625975
H 2.216661 1.485814 -3.215110
H -0.733448 -3.351972 1.114794
C 0.149702 -4.430933 2.778402
C 1.204506 -3.063233 4.473387
H 1.110575 -0.916892 4.140195
H -0.750799 -4.735472 -1.838154
C -1.811434 -3.393381 -3.161552
H -2.699249 -1.825436 -4.353513
H 4.925008 -2.530677 -2.081578
C 4.792685 -0.754371 -3.307565
H 4.397489 1.080239 -4.377571
H -0.136326 -5.414816 2.399258
C 0.851016 -4.319571 3.981365
H 1.749925 -2.977894 5.416235
H -2.402650 -4.188443 -3.622569
H 5.746473 -0.935196 -3.809421
H 1.119332 -5.219063 4.540420
S -0.224106 -0.573486 1.596590
O -3.864850 1.728004 -0.131278
C -5.220900 1.654685 0.281206
C -3.653840 0.924739 -1.296755
C -5.982513 1.105378 -0.913981
H -5.312143 0.976880 1.152002
H -5.556353 2.656048 0.594105
C -4.949761 0.157965 -1.514901
H -3.429804 1.587680 -2.151572
H -2.777868 0.280595 -1.128333
H -6.218070 1.916147 -1.622478
H -6.924812 0.615027 -0.631263
H -5.126742 -0.076585 -2.574021
H -4.935709 -0.793751 -0.959614
O -0.221588 0.523109 2.616687
N -1.503519 -0.784002 0.751709
C -2.727183 -1.074792 1.468571
H -3.124028 -0.215014 2.041590
H -3.496476 -1.348172 0.731975
H -2.642544 -1.924522 2.173636
K -1.488690 2.436014 0.978041

κ*O*,κ*N*-K(THF)

E = –2760.34854473

O 0.729144 1.373703 0.021389
O 4.108743 0.638114 0.075590
S -0.579650 1.546145 -0.667590
C 5.368056 -0.020289 0.079001
C 3.585983 0.723572 1.403048
N -0.339862 1.961107 -2.149606

C -1.523389 0.144821 -0.419858
C -1.418275 2.876213 0.206455
H 6.074943 0.544213 -0.550978
H 5.255475 -1.030869 -0.355053
C 5.796171 -0.096485 1.537073
C 4.449377 -0.200855 2.243941
H 2.521696 0.449994 1.386320
H 3.664898 1.768827 1.756496
C -1.377950 1.692633 -3.120529
Cl -3.261747 0.378924 -0.537725
P -0.903003 -1.119386 0.630712
C -1.825288 2.659651 1.523549
C -1.594600 4.104269 -0.422423
H 6.314816 0.829124 1.835140
H 6.469610 -0.941869 1.737680
H 4.067152 -1.232226 2.191595
H 4.481273 0.095837 3.301658
H -2.255786 2.363270 -3.036296
H -1.759142 0.654267 -3.066610
H -0.957465 1.838196 -4.127583
S -0.662174 -0.845165 2.591408
C -2.053696 -2.513113 0.313017
C 0.654764 -1.691107 -0.158494
H -1.675869 1.676708 1.985986
C -2.420381 3.707273 2.220370
C -2.196581 5.146446 0.284341
H -1.254791 4.226463 -1.453569
C -2.420896 -2.840931 -0.998397
C -2.535409 -3.274321 1.378164
C 1.643108 -2.291630 0.623664
C 0.828413 -1.601251 -1.545172
H -2.744741 3.556725 3.252845
C -2.606481 4.947440 1.602261
H -2.345086 6.116290 -0.196462
H -2.056900 -2.234507 -1.830591
C -3.258890 -3.926755 -1.235803
C -3.377049 -4.361019 1.137798
H -2.239877 -2.996288 2.393908
H 1.496771 -2.342594 1.706349
C 2.798106 -2.796402 0.027026
C 1.986631 -2.102192 -2.138587
H 0.059474 -1.109328 -2.145918
H -3.078501 5.764016 2.154057
H -3.545129 -4.177983 -2.260023
C -3.738063 -4.689023 -0.167803
H -3.752755 -4.952510 1.976341
H 3.567973 -3.265267 0.644836
C 2.973457 -2.701936 -1.353183
H 2.117511 -2.028340 -3.221726
H -4.397787 -5.539602 -0.356576
H 3.879544 -3.097244 -1.819110
K 2.537361 1.526619 -1.939397

6.4.6 Coordinates of the dimeric structures of 1-K

κ*O*,κ*N*,κ*S*-$K_2(THF)_2$

E = –5520.75133972

Cl 4.814649 0.902496 1.696501
Cl -4.814297 -0.902754 -1.696605
S 1.700897 1.489447 -1.399194
S -1.700539 -1.489138 1.398850
O -1.355486 -3.608698 -2.260305
O 1.356179 3.608766 2.260178
C 3.731955 -0.005653 0.668843
C -3.732035 0.005807 -0.668842
P 3.395930 0.494614 -0.978041
P -3.395788 -0.494578 0.977961
C -2.421641 -3.326740 -3.172283
C -1.826570 -4.422129 -1.189980
C 2.422135 3.326604 3.172330
C 1.827555 4.422227 1.190010
C 3.530610 -0.972069 -2.070871
C 4.854045 1.495204 -1.443565
C -3.530660 0.971971 2.070943
C -4.853692 -1.495465 1.443509
C 3.642583 -2.532565 1.931760
H -2.095165 -3.572791 -4.195814
H -2.653151 -2.247422 -3.127921
C -3.607359 -4.161695 -2.708180
C -3.336991 -4.282646 -1.213441
H -1.378656 -4.064520 -0.249305
H -1.514485 -5.471269 -1.351604
C -3.643328 2.532714 -1.931746
H 2.095512 3.572650 4.195817
H 2.653486 2.247256 3.127944
C 3.608051 4.161426 2.708487
C 3.337953 4.282532 1.213712
H 1.379749 4.064766 0.249228
H 1.515593 5.471399 1.351673
C 4.293212 -2.077400 -1.680921
C 2.899686 -0.970945 -3.319212
C 6.140812 0.959497 -1.303193
C 4.686254 2.772922 -1.977672
C -4.293637 2.077134 1.681247
C -2.899526 0.970879 3.319176
C -6.140582 -0.960087 1.303012
C -4.685614 -2.773066 1.977807
C 3.485798 -3.742231 1.257508
C 4.560620 -2.397774 2.975844
H -4.568079 -3.680169 -2.937577
H -3.596939 -5.155593 -3.184319
H -3.634843 -3.355927 -0.699357
H -3.854629 -5.125189 -0.733095
C -3.487020 3.742402 -1.257423
C -4.561224 2.397657 -2.975920
H 4.568663 3.679741 2.938005
H 3.597700 5.155290 3.184699
H 3.635768 3.355811 0.699610
H 3.855788 5.125041 0.733518
H 4.775734 -2.078141 -0.700926
C 4.408511 -3.179281 -2.526135
C 3.018078 -2.074690 -4.163777
H 2.302610 -0.103070 -3.613546
H 6.275497 -0.038914 -0.880738

C 7.248919 1.705089 -1.693408
C 5.800005 3.519244 -2.364366
H 3.673007 3.169080 -2.086987
H -4.776330 2.077847 0.701336
C -4.409094 3.178888 2.526604
C -3.018074 2.074500 4.163883
H -2.302163 0.103134 3.613309
H -6.275486 0.038228 0.880398
C -7.248523 -1.705887 1.693301
C -5.799198 -3.519596 2.364578
H -3.672275 -3.168968 2.087198
H 2.752481 -3.811309 0.452573
C 4.277113 -4.830578 1.623033
C 5.348451 -3.490612 3.329397
H 4.654092 -1.444236 3.499105
H -2.753798 3.811698 -0.452420
C -4.278679 4.830492 -1.622966
C -5.349401 3.490239 -3.329491
H -4.654317 1.444111 -3.499233
H 4.994228 -4.045236 -2.208105
C 3.769094 -3.182011 -3.766627
H 2.518614 -2.069797 -5.135727
H 8.251542 1.285560 -1.582195
C 7.079988 2.986952 -2.222660
H 5.664203 4.520627 -2.779832
H -4.995108 4.044715 2.208773
C -3.769464 3.181656 3.766987
H -2.518441 2.069636 5.135747
H -8.251243 -1.286617 1.581988
C -7.079304 -2.987630 2.222753
H -5.663171 -4.520886 2.780195
H 4.163057 -5.782969 1.099803
C 5.209249 -4.704585 2.653426
H 6.072814 -3.395709 4.141678
H -4.165000 5.782898 -1.099681
C -5.210680 4.704228 -2.653448
H -6.073658 3.395125 -4.141842
H 3.855292 -4.049432 -4.425425
H 7.952294 3.570720 -2.526655
H -3.855792 4.048979 4.425897
H -7.951480 -3.571561 2.526806
H 5.828402 -5.559179 2.936142
H -5.830102 5.558622 -2.936179
S 2.684116 -1.099144 1.423266
O 1.790501 -1.615483 0.339347
S -2.684445 1.099581 -1.423217
O -1.791014 1.616190 -0.339274
N -2.077832 0.431552 -2.681009
C -1.417627 1.269773 -3.665856
H -1.312643 0.691833 -4.597035
H -1.981165 2.188215 -3.916862
H -0.403007 1.579463 -3.353631
N 2.077744 -0.430939 2.681080
C 1.417420 -1.268954 3.666020
H 1.980882 -2.187394 3.917207
H 1.312433 -0.690851 4.597096
H 0.402796 -1.578627 3.353791
K 0.134877 1.330551 1.463047
K -0.134853 -1.330011 -1.463522

κ*N*,κ*S*,κ*Cl*-K_2(THF)$_2$

E = –5520.74331077

Cl 2.414896 0.976694 1.653604
Cl -2.409098 -0.976869 -1.650284
S 1.503008 -0.059922 -2.104532
S -1.508530 0.056649 2.112121
O 0.419798 -3.986256 -1.018115
O -0.420991 3.990233 1.025463
C 3.422650 0.127455 0.489302
C -3.419149 -0.126923 -0.488715
P 3.182790 0.562813 -1.186840
P -3.185790 -0.563743 1.187849
C 1.119018 -3.847829 -2.245636
C -0.899170 -4.451821 -1.290454
C -1.119389 3.847475 2.253022
C 0.899197 4.452372 1.298464
C 4.670484 0.086671 -2.127809
C 3.250239 2.395393 -1.194399
C -4.676094 -0.086588 2.124153
C -3.255357 -2.396224 1.194369
C 5.634821 -1.100339 1.738515
H 1.560948 -4.816626 -2.550653
H 1.931382 -3.120645 -2.098508
C 0.055745 -3.390839 -3.226325
C -1.151514 -4.221862 -2.788315
H -1.598943 -3.889022 -0.654719
H -0.986348 -5.518361 -1.019009
C -5.627750 1.101632 -1.743918
H -1.560668 4.815261 2.562093
H -1.932330 3.121317 2.103826
C -0.055605 3.386311 3.231183
C 1.151058 4.219445 2.795848
H 1.597795 3.888892 0.661965
H 0.988857 5.519023 1.028415
C 5.937590 0.403546 -1.625989
C 4.544658 -0.542493 -3.365873
C 4.086450 3.071795 -0.296971
C 2.524284 3.119107 -2.144825
C -5.941816 -0.402041 1.617925
C -4.553850 0.541623 3.363034
C -4.089473 -3.071110 0.293863
C -2.533526 -3.121275 2.146886
C 6.813794 -1.509696 1.121071
C 5.649795 -0.435246 2.966474
H -0.127419 -2.314154 -3.081562
H 0.337707 -3.549549 -4.276866
H -2.107519 -3.710652 -2.969611
H -1.176585 -5.180279 -3.328668
C -6.808276 1.512486 -1.130477
C -5.639459 0.435795 -2.971516
H 0.127722 2.310286 3.081632
H -0.336953 3.540437 4.282565
H 2.107601 3.709035 2.976565
H 1.174547 5.176701 3.338352
H 6.036587 0.884159 -0.650534
C 7.073931 0.078622 -2.360571
C 5.686318 -0.865010 -4.099238
H 3.545811 -0.783866 -3.739038
H 4.635094 2.505876 0.459620
C 4.194563 4.459259 -0.356844
C 2.632232 4.508929 -2.198894
H 1.872518 2.579902 -2.838263

H -6.038001 -0.881916 0.641828
C -7.080309 -0.076619 2.348920
C -5.697681 0.864736 4.092770
H -3.556051 0.781828 3.739743
H -4.634809 -2.504151 -0.464340
C -4.199675 -4.458447 0.352791
C -2.643623 -4.510961 2.200076
H -1.883185 -2.583226 2.842561
H 6.758083 -2.029032 0.163141
C 8.033711 -1.231824 1.738461
C 6.872912 -0.162223 3.573225
H 4.709139 -0.133355 3.432657
H -6.755123 2.032306 -0.172665
C -8.026504 1.235398 -1.751566
C -6.860903 0.163511 -3.581947
H -4.697623 0.132739 -3.434545
H 8.062748 0.317468 -1.961728
C 6.950257 -0.557381 -3.597700
H 5.586354 -1.363069 -5.066598
H 4.842729 4.982568 0.349968
C 3.468812 5.179550 -1.307274
H 2.059564 5.068494 -2.942304
H -8.068000 -0.314351 1.946635
C -6.960205 0.558514 3.586857
H -5.600487 1.362098 5.060772
H -4.846156 -4.980596 -0.356418
C -3.478135 -5.180089 1.305403
H -2.074226 -5.071615 2.945177
H 8.965745 -1.544984 1.261811
C 8.064010 -0.558228 2.959381
H 6.898163 0.359893 4.532587
H -8.959741 1.549764 -1.278074
C -8.053571 0.561061 -2.972143
H -6.883613 -0.359225 -4.541034
H 7.843989 -0.813930 -4.171701
H 3.550986 6.268254 -1.347677
H -7.855623 0.815468 4.158043
H -3.561961 -6.268693 1.345126
H 9.021370 -0.342685 3.439668
H -9.009604 0.346136 -3.455340
S 4.052104 -1.391591 0.933308
S -4.047208 1.392233 -0.934167
O 4.395840 -2.133032 -0.309186
N 3.072611 -2.002172 1.967370
N -3.064592 2.002564 -1.965389
O -4.394225 2.133461 0.307541
C -3.512808 3.156241 -2.721139
H -3.633312 4.066052 -2.103567
H -2.756381 3.382269 -3.487747
H -4.470615 2.990866 -3.251250
C 3.523546 -3.155405 2.722201
H 2.768319 -3.382911 3.489555
H 3.644862 -4.064860 2.104255
H 4.481641 -2.988621 3.251329
K 0.632874 -1.829235 0.581041
K -0.630740 1.828478 -0.567893

κ*Ph*,κ*S*,κ*Cl*-$K_2(THF)_2$

E = –5520.73154420

K -0.815455 1.892088 0.327708
K 0.851963 -1.927614 -0.271178

Cl -1.765212 -0.879417 1.643642
Cl 1.762926 0.840762 -1.623516
S -1.548227 -0.533290 -1.872623
S 1.580963 0.499653 1.907212
C -3.992839 2.162722 2.088710
C -3.436817 2.575224 3.297220
C -3.050150 3.908933 3.448842
C -3.235598 4.815821 2.405553
C -3.818289 4.395283 1.205505
C -4.198133 3.065419 1.041656
O 0.016546 3.911648 -1.241483
C 4.026338 -2.187679 -2.046267
C 3.471508 -2.628081 -3.245360
C 3.105124 -3.969888 -3.373964
C 3.309264 -4.856850 -2.317174
C 3.890380 -4.407979 -1.126630
C 4.250137 -3.069940 -0.985796
O -0.096825 -3.958340 1.211589
C -3.108031 -0.124593 0.775514
C 3.120395 0.107043 -0.759741
P -3.298315 -0.644589 -0.889596
P 3.312974 0.651618 0.896922
H -3.307146 1.844545 4.097499
H -2.601558 4.239373 4.388744
H -2.931436 5.858036 2.527381
H -3.975307 5.108208 0.392320
H -4.636059 2.714623 0.103505
C -0.237328 3.834743 -2.642355
C 1.308039 4.472880 -1.000653
H 3.326957 -1.912094 -4.056306
H 2.657647 -4.322276 -4.306389
H 3.020925 -5.905500 -2.420817
H 4.062035 -5.105312 -0.302935
H 4.687275 -2.697091 -0.055768
C 0.117541 -3.927489 2.620325
C -1.391824 -4.485430 0.917404
C -4.509687 0.405911 -1.757425
C -3.936950 -2.355361 -0.959384
C 4.567857 -0.346525 1.763638
C 3.895445 2.383387 0.928515
H -1.256751 4.201478 -2.843146
H -0.185744 2.779264 -2.968889
C 0.847149 4.667649 -3.307522
C 2.014343 4.474758 -2.345634
H 1.830343 3.867458 -0.243857
H 1.191484 5.497350 -0.602498
H 1.127933 -4.309039 2.837902
H 0.063436 -2.882705 2.979307
C -0.992690 -4.770475 3.227058
C -2.132031 -4.526719 2.243623
H -1.884117 -3.841204 0.172819
H -1.282710 -5.495369 0.481971
C -5.823124 -0.020239 -1.975763
C -4.092392 1.647233 -2.246067
C -4.654883 -2.866689 0.126638
C -3.668041 -3.169097 -2.065995
C 5.871143 0.122409 1.953086
C 4.194895 -1.590458 2.280673
C 4.590183 2.893158 -0.173392
C 3.607650 3.212301 2.018874
H 1.062175 4.337164 -4.333375
H 0.548733 5.727834 -3.345408

H 2.495309 3.499224 -2.516332
H 2.783678 5.255972 -2.417764
H -1.230172 -4.475275 4.258722
H -0.707720 -5.835054 3.232928
H -2.604618 -3.551593 2.438314
H -2.913176 -5.299072 2.267449
H -6.150683 -0.988169 -1.593316
C -6.715115 0.801064 -2.660342
C -4.992111 2.474731 -2.916541
H -3.053415 1.958342 -2.115733
H -4.860537 -2.222357 0.984341
C -5.097600 -4.188792 0.104705
C -4.114439 -4.488416 -2.084020
H -3.087664 -2.765031 -2.899352
H 6.163701 1.092305 1.547966
C 6.797701 -0.659365 2.637881
C 5.129484 -2.378341 2.951411
H 3.163527 -1.934330 2.171866
H 4.808626 2.237628 -1.019465
C 4.991498 4.228512 -0.183108
C 4.012455 4.545121 2.005135
H 3.045683 2.808448 2.864844
H -7.740232 0.463222 -2.829760
C -6.305185 2.053120 -3.123053
H -4.660768 3.447401 -3.288205
H -5.650811 -4.588203 0.957961
C -4.826147 -5.000057 -0.996821
H -3.899382 -5.123682 -2.946632
H 7.814759 -0.288322 2.784875
C 6.432338 -1.914248 3.129408
H 4.833519 -3.353485 3.345677
H 5.526445 4.626330 -1.048652
C 4.701427 5.054748 0.902504
H 3.782673 5.192129 2.855127
H -7.009613 2.697127 -3.654987
H -5.167501 -6.038107 -1.008070
H 7.163726 -2.527280 3.661559
H 5.010074 6.102949 0.888961
S 4.366015 -0.428030 -1.804090
O 4.226794 0.151220 -3.161494
N 5.650465 -0.252719 -0.974734
C 6.861588 -0.927726 -1.372781
H 7.362341 -0.442949 -2.231196
H 7.563307 -0.898050 -0.523829
H 6.719984 -1.994507 -1.636291
S -4.356801 0.412550 1.815118
O -4.243792 -0.190553 3.164577
N -5.633234 0.270917 0.966908
C -6.839758 0.955574 1.362393
H -7.528748 0.957194 0.502555
H -7.361123 0.458550 2.201285
H -6.685894 2.013432 1.653479

κ*O*,κ*N*,κ*S*-$K_2(THF)_4$

E = –5985.32646643

Cl 4.659622 2.154968 1.219149
Cl -4.643748 -2.154364 -1.237084
S 1.595233 0.898307 -1.830508
S -1.598876 -0.889438 1.834979
O -1.217216 -4.253096 -0.425467
O 1.203794 4.266293 0.430560

C 3.732211 0.794329 0.631158
C -3.719310 -0.795011 -0.641623
P 3.422034 0.538008 -1.074891
P -3.422295 -0.538086 1.066616
C -2.045688 -4.593427 -1.535261
C -1.773248 -4.747737 0.782454
C 2.051963 4.587860 1.531306
C 1.754542 4.754095 -0.782882
C 3.968193 -1.145579 -1.544593
C 4.668869 1.584572 -1.912763
C -3.981747 1.141581 1.534202
C -4.668945 -1.592037 1.895775
C 3.949465 -0.801695 2.921897
H -1.435008 -5.102739 -2.300169
H -2.447237 -3.662746 -1.969703
C -3.153449 -5.482890 -0.982057
C -3.232160 -5.031925 0.472544
H -1.633567 -3.985963 1.565244
H -1.242250 -5.669453 1.091975
C -3.934362 0.810594 -2.927286
H 1.457316 5.096800 2.308926
H 2.450281 3.649558 1.952394
C 3.160819 5.469785 0.969315
C 3.219553 5.021627 -0.486951
H 1.599842 3.993837 -1.564436
H 1.230829 5.681458 -1.087601
C 4.878225 -1.839559 -0.742516
C 3.562909 -1.688861 -2.767200
C 6.030326 1.410151 -1.632585
C 4.270347 2.523161 -2.864318
C -4.889595 1.830771 0.725506
C -3.590300 1.685593 2.760934
C -6.029595 -1.424090 1.607770
C -4.271585 -2.529520 2.848888
C 4.232162 -2.162176 2.812682
C 4.590551 -0.007141 3.874914
H -4.099451 -5.355823 -1.527657
H -2.866360 -6.545411 -1.040990
H -3.818413 -4.104847 0.550476
H -3.676232 -5.779394 1.145620
C -4.214362 2.171523 -2.817098
C -4.576105 0.018291 -3.881790
H 4.111539 5.332949 1.504205
H 2.883152 6.534487 1.034155
H 3.794928 4.088396 -0.572404
H 3.665173 5.765436 -1.163050
H 5.181134 -1.410082 0.215516
C 5.388057 -3.064576 -1.168625
C 4.080540 -2.909926 -3.193295
H 2.836981 -1.145387 -3.377905
H 6.347113 0.673240 -0.890916
C 6.979998 2.181823 -2.294987
C 5.224921 3.296204 -3.526364
H 3.204075 2.634280 -3.079230
H -5.181675 1.400821 -0.235678
C -5.411374 3.051546 1.149272
C -4.120025 2.902200 3.184751
H -2.865474 1.146276 3.376589
H -6.345735 -0.687852 0.865173
C -6.979334 -2.201399 2.263438
C -5.226174 -3.308261 3.504186
H -3.206186 -2.635178 3.070643

H 3.709325 -2.759033 2.065706
C 5.180473 -2.732522 3.659998
C 5.539281 -0.586115 4.715242
H 4.340807 1.052454 3.952970
H -3.690777 2.766401 -2.069091
C -5.161068 2.744552 -3.664442
C -5.523181 0.599902 -4.722114
H -4.328177 -1.041635 -3.960932
H 6.099628 -3.603465 -0.538007
C 4.995819 -3.597493 -2.396691
H 3.765257 -3.327801 -4.152571
H 8.040910 2.044981 -2.072621
C 6.578324 3.128058 -3.241192
H 4.906831 4.031943 -4.268931
H -6.121043 3.586757 0.513392
C -5.033272 3.584837 2.381634
H -3.815400 3.320879 4.147103
H -8.039555 -2.069582 2.034827
C -6.578640 -3.146811 3.210885
H -4.908812 -4.043146 4.247909
H 5.405883 -3.798686 3.580430
C 5.836541 -1.945606 4.607457
H 6.046594 0.028055 5.462915
H -5.384477 3.811065 -3.583973
C -5.818026 1.959846 -4.613080
H -6.031081 -0.012507 -5.470836
H 5.404482 -4.553414 -2.733329
H 7.326512 3.732636 -3.759728
H -5.451344 4.537372 2.716343
H -7.326881 -3.755852 3.724095
H 6.580052 -2.395227 5.269776
H -6.560271 2.411524 -5.275421
S 2.795540 -0.029165 1.774167
O 2.153325 -1.166854 1.043794
S -2.782296 0.034955 -1.779683
O -2.143564 1.171145 -1.043843
N -1.911704 -0.976598 -2.565380
C -1.270298 -0.538956 -3.792204
H -1.018601 -1.429353 -4.389830
H -1.908207 0.106166 -4.425902
H -0.327636 0.007947 -3.607301
N 1.926773 0.985587 2.557967
C 1.284174 0.549496 3.784803
H 1.923782 -0.089526 4.422972
H 1.026043 1.441028 4.377933
H 0.344845 -0.003236 3.599984
K -0.180360 1.902008 0.742370
K 0.180941 -1.898127 -0.736960
O -1.830222 4.085123 1.068825
C -1.718768 5.268639 1.824841
C -2.082747 4.498141 -0.256467
C -2.783281 6.222055 1.263886
H -1.862842 5.014514 2.884771
H -0.705597 5.693989 1.697316
C -3.084911 5.644092 -0.132660
H -1.142552 4.847648 -0.725612
H -2.444128 3.623059 -0.811799
H -2.411732 7.256015 1.222523
H -3.684860 6.220395 1.892201
H -2.965540 6.382197 -0.938456
H -4.112824 5.257491 -0.179048
O 1.822169 -4.092877 -1.026442

C 1.726542 -5.286723 -1.768015
C 2.061980 -4.487059 0.306832
C 2.787677 -6.228268 -1.180089
H 0.713584 -5.715165 -1.649047
H 1.884008 -5.045973 -2.829140
C 3.076570 -5.624050 0.207970
H 2.407458 -3.601825 0.856185
H 1.119696 -4.840816 0.768711
H 3.694614 -6.238272 -1.800551
H 2.416343 -7.261512 -1.121981
H 4.099929 -5.225055 0.252323
H 2.961717 -6.349733 1.025620

7. References

[1] K.-S. Feichtner, S. Englert, V. H. Gessner, *Chem Eur. J.* **2016**, *22*, 506-510.

[2] a) G. M. Sheldrick, *Acta Cryst.* **2008**, *A64*, 112; b) A. Thorn, B. Dittrich, G. M. Sheldrick, *Acta Cryst.* **2012**, *A68*, 448; c) G. M. Sheldrick, *Acta Cryst*. **2008**, *A64*, 112; d) G. M. Sheldrick, *Acta Cryst.* **2015**, *C71*, 3.

[3] R. Dennington, T. A. Keith, J. M. Millam, *GaussView*, Semichem Inc. Shawnee Mission KS, **2016**.

[4] M. J. Frisch, G. W. Trucks, H. B. Schlegel, G. E. Scuseria, M. A. Robb, J. R. Cheeseman, G. Scalmani, V. Barone, G. A. Petersson, H. Nakatsuji et al., *Gaussian 16 Revision C.01*, **2016**.

[5] a) W. Kohn, L. J. Sham, *Phys. Rev.* **1965**, *140*, A1133-A1138; b) P. Hohenberg, W. Kohn, *Phys. Rev.* **1964**, *136*, B864-B871; c) J. Reinhold, *Cryst. Res. Technol.* **1990**, *25*, 624; d) R. G. Parr, W. Yang, *Density-Functional Theory of Atoms and Molecules (International Series of Monographs on Chemistry)*, Oxford University Press, USA, **1994**.

[6] C. Adamo, V. Barone, *J. Chem. Phys.* **1999**, *110*, 6158.

[7] A. Bergner, M. Dolg, W. Kuechle, H. Stoll, H. Preuss *Mol. Phys.* **1993**, *80*, 1431.

[8] A. Schäfer, H. Horn, R. Ahlrichs, *J. Chem. Phys.* **1992**, *97*, 2571.

[9] a) S. Grimme, S. Ehrlich, L. Goerigk, *J. Comput. Chem.* **2011**, *32*, 1456; b) S. Grimme, J. Antony, S. Ehrlich, H. Krieg, *J. Chem. Phys.* **2010**, *132*, 154104.

[10] P. Deglmann, F. Furche, R. Ahlrichs, *Chem. Phys. Lett.* **2002**, *362*, 511.

[11] A. Schäfer, C. Huber, R. Ahlrichs, *J. Chem. Phys.* **1994**, *100*, 5829.
